# Mitochondrial protein import stress causes lysosomal damage and progressive tissue atrophy

**DOI:** 10.1038/s44319-026-00774-9

**Published:** 2026-04-27

**Authors:** Nicholas A Brennan, Xiaowen Wang, Arnav Rana, Sanaea Z Bhagwagar, Jason Horton, Patricia M Kane, Xin Jie Chen

**Affiliations:** 1 Department of Biochemistry and Molecular Biology, State University of New York Upstate Medical University, Syracuse, NY 13210 USA; 2 Neuroscience and Physiology, State University of New York Upstate Medical University, Syracuse, NY 13210 USA

**Keywords:** Molecular Biology of Disease, Musculoskeletal System, Organelles

## Abstract

Mitochondrial and lysosomal abnormalities co-occur in aging-related diseases with progressive tissue atrophy. It remains unclear whether these two pathogenic pathways affect tissue homeostasis independently, convergently or epistatically. We show that mitochondrial protein import stress causes vacuolar damage in yeast, manifested by V-ATPase disassembly, and vacuolar deacidification and fragmentation. In a mouse model of mitochondrial protein import stress induced by overloading of the nuclear-encoded ANT1 protein, we observe progressive muscle atrophy independent of bioenergetic defects. Like in yeast mutants with severe vacuolar damage, genes involved in amino acid uptake/biosynthesis, one-carbon metabolism, lysosomal biogenesis and iron homeostasis are activated in the skeletal muscle of *Ant1*-transgenic mice. The affected muscles accumulate glycogen, lipofuscin and poorly processed multivesicular bodies. Despite activation of lysosomal repair and lysophagic pathways, autophagic flux is severely stalled. During aging, various proteolytic cathepsins are increasingly released from the lysosomal lumen into the cytosol. Together with proteasomal activation, this may contribute to unbalanced proteostasis, reduced myofiber size and skeletal muscle atrophy. Our study therefore discovered an evolutionarily conserved mitochondria-to-lysosome proteotoxic axis that affects tissue mass homeostasis during aging.

## Synopsis

In response to mitochondrial stress, unimported mitochondrial proteins cause vacuolar dysfunction in yeast and lysosomal damage and progressive muscle atrophy in mice.

- In yeast, mitochondrial protein import stress disrupts vacuolar integrity, causing V-ATPase disassembly and loss of cellular pH homeostasis.
- In mice, mitochondrial protein import stress causes lysosomal damage independent of bioenergetic deficiency, leading to the release of cathepsins into the cytosol and progressive atrophy of the skeletal muscle.
- An evolutionarily conserved mitochondria-to-vacuole/lysosome proteotoxic axis is therefore defined, which may have implications for understanding the mechanism of aging-associated tissue atrophy.

## Abstract (web summary)

In response to mitochondrial stress, unimported mitochondrial proteins cause vacuolar dysfunction in yeast and lysosomal damage and progressive muscle atrophy in mice.

## Graphical abstract

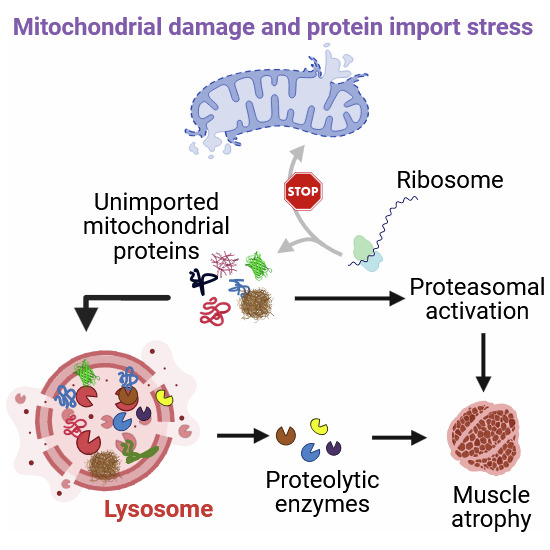

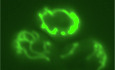

## Introduction

Severe mitochondrial dysfunction typically affects children and young adults due to bioenergetic deficits (Calvo et al, 2006; Nunnari and Suomalainen, 2012; Wallace, 2005). Nuclear mutations affecting mitochondrial functions also cause late-onset degenerative diseases, including Parkinson’s disease (PD), amyotrophic lateral sclerosis and frontotemporal dementia. However, because many of these genes are not directly involved in oxidative phosphorylation (OXPHOS), whether these diseases are solely caused by bioenergetic impairment has long been questioned (Fukui and Moraes, 2008; Schon and Przedborski, 2011). On the other hand, dysfunction of the lysosome, an intracellular organelle critical for protein degradation, nutrient sensing, pH control, ionic and amino acid homeostasis and vesicular trafficking, is also associated with these aging-related degenerative diseases (Udayar et al, 2022). These findings invited the question of whether and how the mitochondrial and lysosomal pathogenic pathways interact to affect cell fitness and tissue homeostasis.

The yeast vacuole, functional counterpart of the mammalian lysosome, has been shown to directly interact with mitochondria to facilitate ion and lipid exchange (Cisneros et al, 2022), in addition to its role in mitochondrial quality control via mitophagy (Pickles et al, 2018). Vacuolar dysfunction also disrupts cellular processes such as amino acid homeostasis, which is toxic to mitochondria (Hughes et al, 2020). Does mitochondrial stress reciprocally affect vacuolar/lysosomal function? Previous studies have shown that severe mitochondrial respiratory chain deficiency causes lysosomal damage in cultured mammalian cells, but the underlying mechanism(s) is poorly understood (Baixauli et al, 2015; Demers-Lamarche et al, 2016; Fernandez-Mosquera et al, 2017; Raimundo et al, 2016; Yagi et al, 2021).

The mitochondrial proteome consists of ~1000–1500 proteins. Most of these proteins are nuclear-encoded and transported into mitochondria via dedicated protein translocation machineries, including the translocase of outer membrane (TOM) and translocases of inner membrane 22 (TIM22) and 23 (TIM23) (Chacinska et al, 2009). Protein translocation through TIM22 and TIM23 is driven by the membrane potential on the inner mitochondrial membrane (IMM). The membrane potential is vulnerable and can be affected by various mitochondrial stressors. Recent studies have shown that many types of mitochondrial damage can compromise the efficiency of protein import and induce cell degeneration via mitochondrial Precursor Overaccumulation Stress (mPOS). The phenomenon of mPOS is characterized by the toxic accumulation of unimported preproteins in the cytosol, followed by activation of proteasomal function as an anti-mPOS response (Wang and Chen, 2015; Wrobel et al, 2015). However, how mPOS in the cytosol affects cell fitness and survival is unknown. In this report, we found that mitochondrial protein import stress can cause severe vacuolar/lysosomal damage. This leads to the inhibition of cell growth in yeast and progressive atrophy of skeletal muscle in mice, in a manner independent of bioenergetic deficiency and oxidative stress.

## Results

### Mitochondrial protein import stress synergizes with vacuolar mutants to inhibit cell growth in yeast

**Figure 1:**
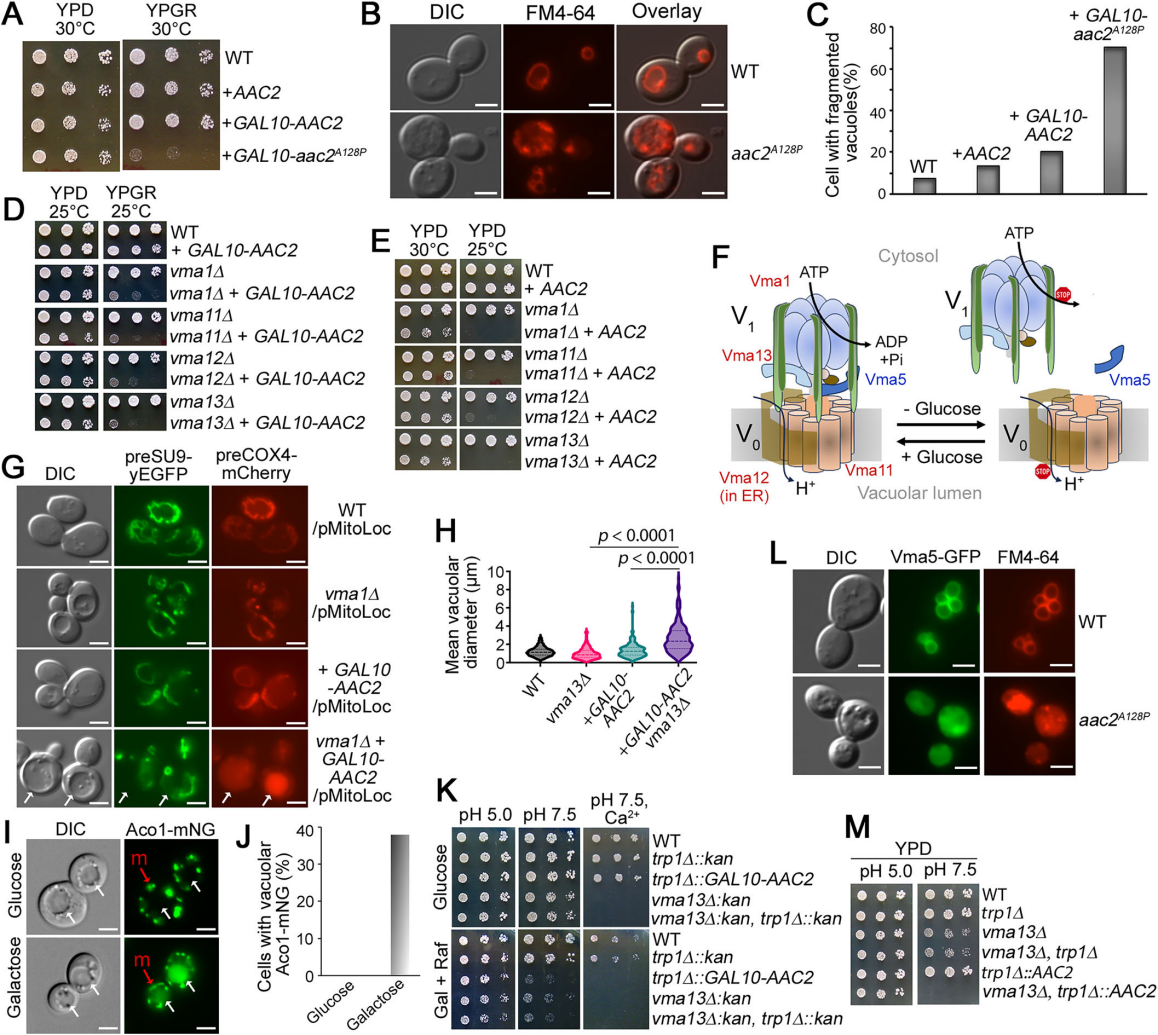
Mitochondrial protein import stress causes vacuolar damage in yeast. (**A**) Expression of the mitochondrial protein import clogger Aac2^A128P^ from the *GAL10* promoter inhibits cell growth on complete galactose plus raffinose (YPGR) but not glucose (YPD) medium. (**B**) FM 4-64 staining and fluorescence microscopy showing severe fragmentation of the vacuole in cells expressing *GAL10*-*aac2*^*A128P*^. (**C**) Quantification of vacuolar fragmentation in cells expressing the chromosomally integrated *GAL10-aac2*^*A128P*^, *GAL10-AAC2*, and *AAC2*. (**D**) Expression of the chromosomally integrated *GAL10-AAC2* is synthetically lethal with *vma1Δ*, *vma11Δ*, *vma12Δ*, and *vma13Δ* on YPGR medium. (**E**) Expression of an extra copy of *AAC2* from the chromosome is synthetically lethal with the *vma* mutants on YPD medium. (**F**) Schematics of V-ATPase structure, and its assembly and disassembly in response to glucose availability. Vma1, 5, 11, and 13 are subunits of V-ATPase in the V_1_ or V_o_ subdomain. Note that Vma12 is an assembly factor of V_o_ localized in the endoplasmic reticulum (ER). (**G**) Aac2 overloading induces the vacuolar overaccumulation of preCOX4-mCherry but not preSU9-yEGFP in *vma1Δ* cells. White arrows demark the position of vacuoles. (**H**) Vacuolar diameter. *P* values were calculated using an unpaired two-tailed Student’s *t*-test. (**I**) Expression of *GAL10-AAC2* in galactose medium induces the accumulation of Aco1-mNG in the vacuole of *vma1Δ* cells. Cells (*GAL10-AAC2 ACO1-mNG vma1Δ*) were grown in complete galactose or glucose medium at 30 °C overnight before being examined with fluorescence microscopy. M, mitochondria. White arrows denote the position of the vacuole. (**J**) Quantification of (**I**). > 500 cells were examined. (**K**) Expression of *GAL10-AAC2* causes hypersensitivity to high extracellular pH and Ca^2+^ loading (60 mM CaCl_2_) like a *vma* mutant. *GAL10-AAC2* was expressed from the *TRP1* locus in the chromosome by replacing the *TRP1* gene. A *TRP1* knockout strain was therefore used as an additional control for *trp1Δ::GAL10-AAC2*. (**L**) Expression of *aac2*^*A128P*^ induces the dissociation of Vma5-GFP from the vacuolar membrane. (**M**) Expression of one extra copy of *AAC2* sensitizes *vma13Δ* cells to high extracellular pH. Scale bars, 2.5 μm. Source data are available online for this figure.

Adenine nucleotide translocase 1 (ANT1) is an abundant nuclear-encoded protein involved in ATP/ADP exchange across the IMM. The A114P mutation in the human *ANT1* gene causes autosomal dominant progressive external ophthalmoplegia, manifested by symptoms including muscle weakness and neurological disorders (Kaukonen et al, 2000; Kaukonen et al, 1999; Simoncini et al, 2017). Previous studies have shown that the mutant ANT1(p.A114P) protein and its yeast equivalent, Aac2^A128P^, have increased retention to the TOM and TIM22 mitochondrial protein translocases, which clogs the protein import process (Coyne et al, 2023). This strongly inhibits the growth of yeast cells when *aac2*^*A128P*^ expression is induced by the *GAL10* promoter on galactose medium (Fig. 1A). To learn how Aac2^A128P^-induced protein import clogging affects cell growth, we examined vacuolar morphology and function in the mutant cells. We found that Aac2^A128P^ causes extensive vacuolar fragmentation (Fig. 1B). To a lesser extent, vacuolar fragmentation was also observed in cells harboring an extra chromosomally integrated copy of the wild-type *AAC2* expressed from its native or the galactose inducible *GAL10* promoter (Fig. 1C). Mitochondrial inner membrane protein overloading is known to cause protein import clogging (Weidberg and Amon, 2018). Thus, like Aac2^A128P^, overloading of Aac2 can also cause vacuolar stress via mitochondrial protein import clogging.

**Figure EV1:**
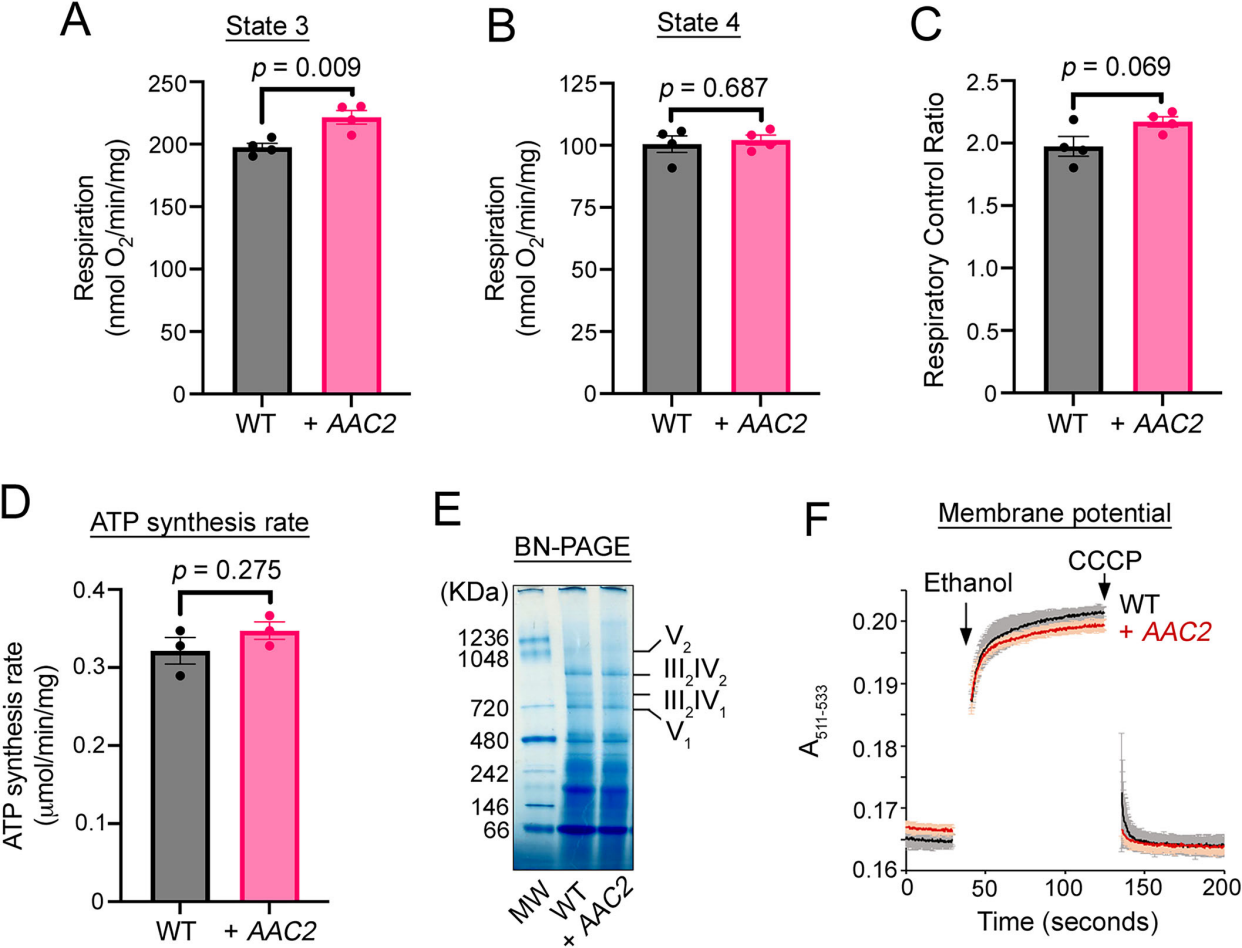
Expression of an extra copy of *AAC2* integrated into the chromosome has little effect on mitochondrial function. Mitochondria were isolated from BY4741/AN1 (+*AAC2*) (*α, lys2, his3, leu2, ura3, trp1Δ::AAC2-HIS3*) and the control strain BY4741 (WT) (*α, lys2, his3, leu2, ura3*) grown in complete galactose (YPGal) medium. (**A**, **B**) Ethanol-stimulated state 3 and state 4 respiration. (**C**) Respiratory control ratio. (**D**) ATP synthesis rate. (**E**) Respiratory complex assembly. V_1_, complex V or ATP synthetase; V_2_, dimer of complex V; III_2_IV_2_, supercomplex with the denoted stoichiometry; III_2_IV_1_, supercomplex with the denoted stoichiometry. (**F**) Membrane potential after the energization of mitochondria with ethanol. The error bars for three biological replicates are shown. For (**A**–**D**), data were means ± SEM of 3–4 biological replicates. *P* values were calculated by a two-tailed Student’s *t*-test).

We subsequently found that *AAC2* overexpression is synthetically lethal with mutations in the V-ATPase, an evolutionarily conserved proton pump critical for vacuolar (or lysosomal in mammalian cells) acidification. Expression of *GAL10-AAC2* (Fig. 1D) or one extra copy of chromosomally integrated *AAC2* (Fig. 1E) strongly inhibited the growth of cells lacking *VMA1*, *11*, *12*, and *13*. *VMA11* and *13* encode integral subunits of the V-ATPase, whereas *VMA12* encodes a V-ATPase assembly factor located at the endoplasmic reticulum (Fig. 1F). Importantly, expression of an extra copy of *AAC2* has little effect on mitochondrial respiration, membrane coupling efficiency, ATP synthesis rate, respiratory complex biogenesis/assembly and membrane potential (Fig. EV1A–F). Moderate overloading of mitochondria by Aac2 seems to have a rather subtle effect on the import of proteins involved in the limiting step(s) of the OXPHOS pathway. We thus concluded that the synergistic effect of Aac2 overloading and *vma* mutations in inhibiting cell growth is independent of mitochondrial bioenergetics. We speculated that unimported mitochondrial proteins are trafficked to the vacuole for degradation, and that mitochondrial protein import stress results in the overaccumulation of unimported mitochondrial proteins in the vacuole. Combined with the loss of V-ATPase activity and reduced degradative capacity, this may lead to severe vacuolar damage incompatible with cell growth. Vacuolar damage may, in turn, slow down protein degradation, thereby further heightening proteostatic burden in the cytosol and contributing to the inhibition of cell growth. Supporting this, we found that mCherry fused to the weak preCOX4 mitochondrial targeting sequence accumulates in the vacuole of *GAL10-AAC2 vma1Δ* but not *GAL10-AAC2* or *vma1Δ* cells (Fig. 1G). The *GAL10-AAC2 vma1Δ* cells also have significantly enlarged vacuolar size (Fig. 1H), consistent with cargo protein overaccumulation under the deacidified conditions. We also detected vacuolar accumulation of the mitochondrial matrix protein Aco1 in *vma1Δ* cells following induction of *GAL10-AAC2* in galactose medium (Fig. 1I,J).

Next, we found that induction of *GAL10*-*AAC2* on galactose plus raffinose medium is sufficient to sensitize cells to elevated extracellular pH (pH 7.5) (Fig. 1K). The sensitivity to high pH is exacerbated by Ca^2+^. These are characteristic phenotypes of *vma* mutants defective in V-ATPase (Kane, 2007). The data therefore suggest that *AAC2* overexpression-induced stress may impair vacuolar acidification. Supporting this, we found that the vacuolar localization of Vma5, a cytosolically oriented peripheral membrane subunit of V-ATPase (Fig. 1F), is disrupted in cells expressing the clogger protein Aac2^A128P^ (Fig. 1L). Mitochondrial protein import stress, therefore, affects V-ATPase biogenesis and/or assembly. Moreover, we found that expression of an extra copy of *AAC2* enhances the growth defect of *vma13Δ* cells on medium buffered to pH 7.5 (Fig. 1M). This suggests that mitochondrial protein import stress can promote additional vacuolar damage independent of V-ATPase activity.

**Figure 2:**
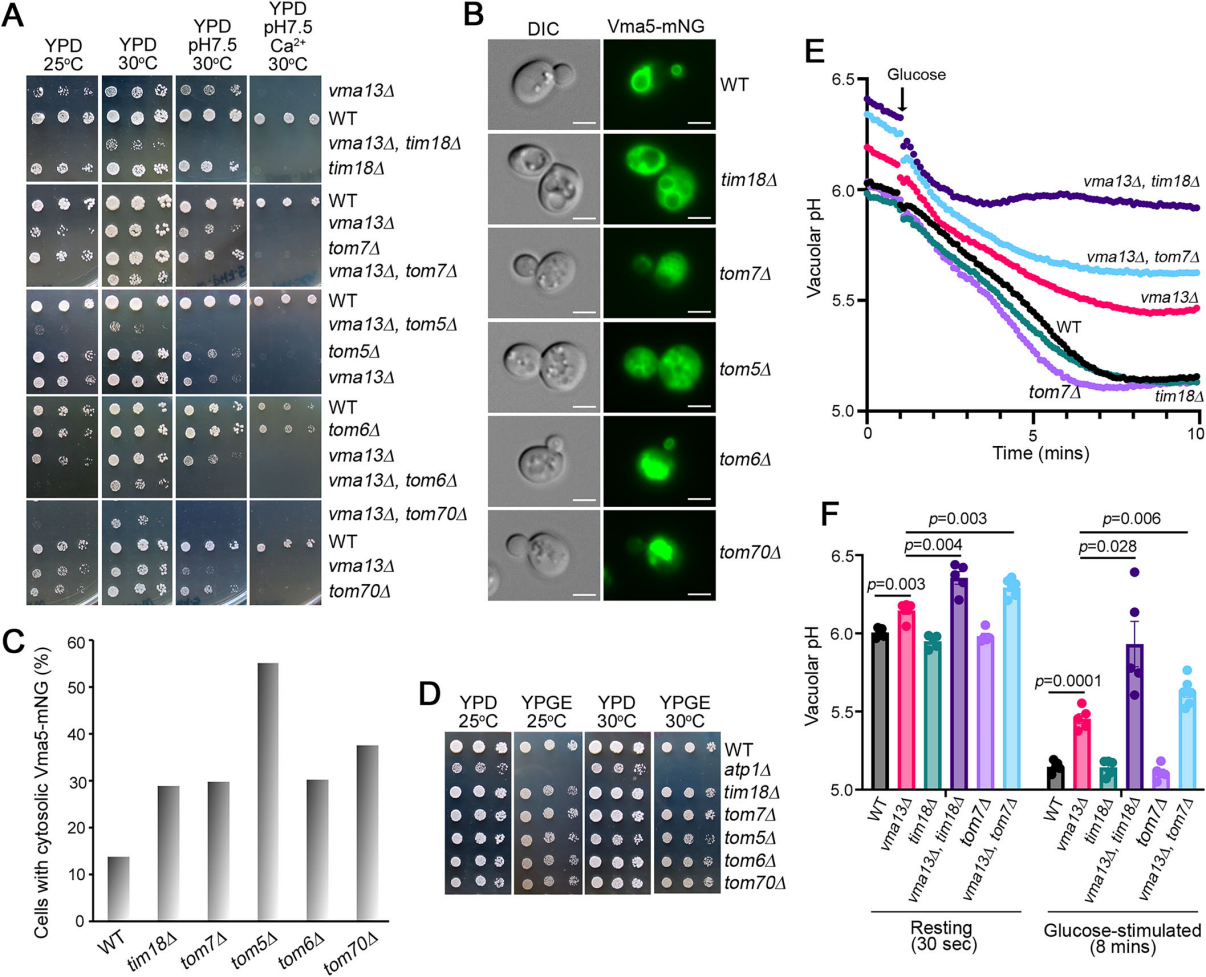
Mitochondrial protein import mutants have a *vma*-like phenotype, and affect V-ATPase assembly and vacuolar acidification. (**A**) The *tim18Δ*, *tom7Δ*, *tom5Δ*, *tom6Δ*, and *tom70Δ* cells are synthetically lethal with *vma13Δ* on glucose medium at 25 °C or at 30 °C (pH 7.5). Cells with *tim18Δ*, *tom7Δ*, *tom5Δ*, and *tom70Δ*, but not *tom6Δ*, have a *vma*-like phenotype on pH 7.5 medium supplemented with 60 mM CaCl_2_. (**B**) Fluorescence microscopy showing increased cytosolic localization of Vma5-mNG in mitochondrial protein import mutants. Cells were grown in YPD at 25 C° to the stationary phase before being imaged. Scale bars, 2.5 μm. (**C**) Quantification of (**B**). More than 500 cells were counted for each strain. (**D**) Growth of mitochondrial protein import mutants on glucose (YPD) and the non-fermentable glycerol plus ethanol (YPGE) medium. The respiratory deficient *atp1Δ* mutant was included as a negative control. (**E**) BCECF (2’,7’-Bis-(2-Carboxyethyl)-5-(6)-Carboxyfluorescein)-based ratiometric measurements of vacuolar pH. Cells were first grown in complete glucose medium at 25 °C to an OD_600_ = ~1.0, before being depleted of glucose and loaded with BCECF. The vacuolar acidification assay is initiated by glucose re-addition. (**F**) Quantification of vacuolar pH measurements at resting (30 s) and glucose-stimulated state (8 min). Error bars represent mean ± SEM of a total of 5–6 repeats from two biological replicates. *P* values were calculated by a two-tailed Student’s *t*-test. Source data are available online for this figure.

Finally, we tested whether intrinsic damage to the mitochondrial protein import machinery causes vacuolar stress. *TIM18* encodes a component of the TIM22 complex on the IMM, whereas *TOM7, 5, 6*, and *70* encode non-essential components of the TOM complex on the OMM. Indeed, we found that disruption of all these genes creates a synthetic lethality with *vma13Δ* when grown at 25 °C or challenged by high pH at 30 °C on glucose medium (Fig. 2A). Furthermore, we found that *tim18Δ*, *tom7Δ*, *tom5Δ* and *tom70Δ* single mutants are hypersensitive to Ca^2+^ at pH 7.5, a stringent condition non-permissible for the growth of cells with vacuolar dysfunction. While synthetically lethal with *vma13Δ, tom6Δ* single mutant cells can still grow at high pH with or without Ca^2+^ supplementation. Taken together, these data further support the idea that mitochondrial protein import stress can intrinsically cause vacuolar damage and affect pH homeostasis. Similar to *aac2*^*A128P*^ cells, Vma5-GFP is increasingly mislocalized to the cytosol and large clumps in *tim18Δ*, *tom7Δ*, *tom5Δ*, *tom6Δ*, and *tom70Δ* mutants, when cells were grown to the stationary phase to derepress mitochondrial respiration (Fig. 2B,C). The data provide further support for the idea that mitochondrial protein import stress affects V-ATPase assembly. Although disruption of *TIM18, TOM7, TOM5, TOM6*, and *TOM70* is expected to reduce protein import efficiency, the mutant cells maintain respiratory growth capacity on non-fermentable carbon sources at both 25 and 30 °C (Fig. 2D). It is therefore unlikely that vacuolar stress is caused by OXPHOS deficiency.

### Mitochondrial protein import stress disrupts vacuolar and cytosolic pH homeostasis

**Figure 3:**
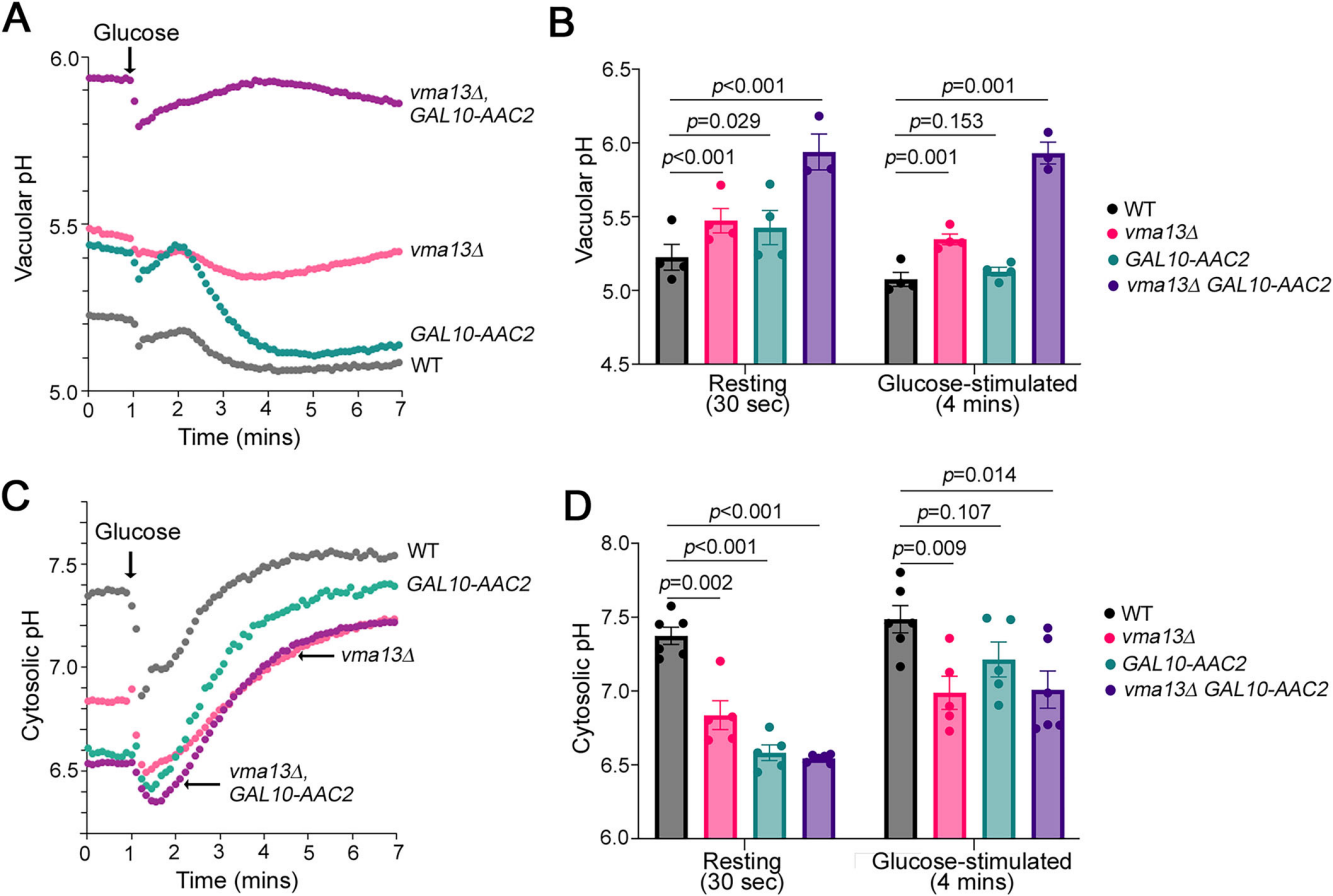
Mitochondrial protein import stress causes vacuolar deacidification in yeast. (**A**) BCECF (2’,7’-Bis-(2-carboxyethyl)-5-(6)-carboxyfluorescein)-based ratiometric measurements of vacuolar pH. Cells were first grown in YPGR to mid-exponential phase to allow the expression of *GAL10-AAC2*, before being loaded with BCECF and glucose. (**B**) Quantification of vacuolar pH measurements at resting (30 s) and glucose-stimulated state (4 min). (**C**) pHluorin-based ratiometric measurements of cytosolic pH. (**D**) Quantification of cytosolic pH measurements at resting (30 s) and glucose-stimulated state (4 min). For (**B**, **D**), the data were given as mean ± SEM of 3–6 independent experiments and *p* values were calculated by two-tailed Student’s *t*-test. Source data are available online for this figure.

The catalytic V_1_ sector of the V-ATPase is dissociated from the holoenzyme upon glucose depletion and reassembled with the V_o_ sector on the membrane in response to glucose addition to stimulate vacuolar acidification (Fig. 1F) (Kane, 1995). To provide support for mitochondria-induced vacuolar deacidification, we used the ratiometric fluorescent indicator BCECF-AM to directly measure vacuolar pH in cell suspensions before and after the addition of glucose. We found that *GAL10-AAC2* cells, like *vma* mutants, have significantly higher resting vacuolar pH compared with the wildtype (Fig. 3A,B). Interestingly, glucose stimulates vacuolar acidification in these cells, ultimately attaining a pH close to that in the wildtype. The data suggest that *AAC2* overexpression disassembles the V-ATPase, which is reversed by glucose. We also found that, consistent with their severe growth defect, the *GAL10-AAC2 vma13Δ* cells have a much higher resting vacuolar pH compared with either *vma13Δ* or *GAL10-AAC2* cells. As expected, due to the loss of the V-ATPase, glucose failed to stimulate vacuolar acidification. The data suggest that additional vacuolar damage in *vma13Δ* cells is caused by mitochondrial protein import stress. Using the ratiometric pHluorin as a sensor, we found that cells expressing *GAL10-AAC2* have lower cytosolic pH compared with the wildtype (Fig. 3C,D). Cytosolic pH is partially recovered in response to glucose in *GAL10-AAC2* but much less in *GAL10-AAC2 vma13Δ* cells. The data provide direct evidence for an effect of mitochondrial protein import stress on vacuolar acidification and cytosolic pH homeostasis.

We found that *tim18Δ* and *tom7Δ* single mutants grown under glucose-repressed conditions do not affect glucose-induced vacuolar acidification (Fig. 2E,F). It is possible that protein import stress is relatively low under glucose-repressed conditions, or that a compensatory mechanism is induced to maintain vacuolar acidity. Importantly, we found that in the *vma13Δ* background, *tim18Δ* and *tom7Δ* resulted in significantly higher resting and glucose-stimulated vacuolar pH compared with *vma13Δ* single mutant. These data are consistent with the conclusion that mitochondrial protein import stress causes vacuolar damage independent of V-ATPase under these conditions.

### Synergistic transcriptional response between mitochondrial protein import stress and loss of V-ATPase activity provides further support for vacuolar damage

**Figure 4:**
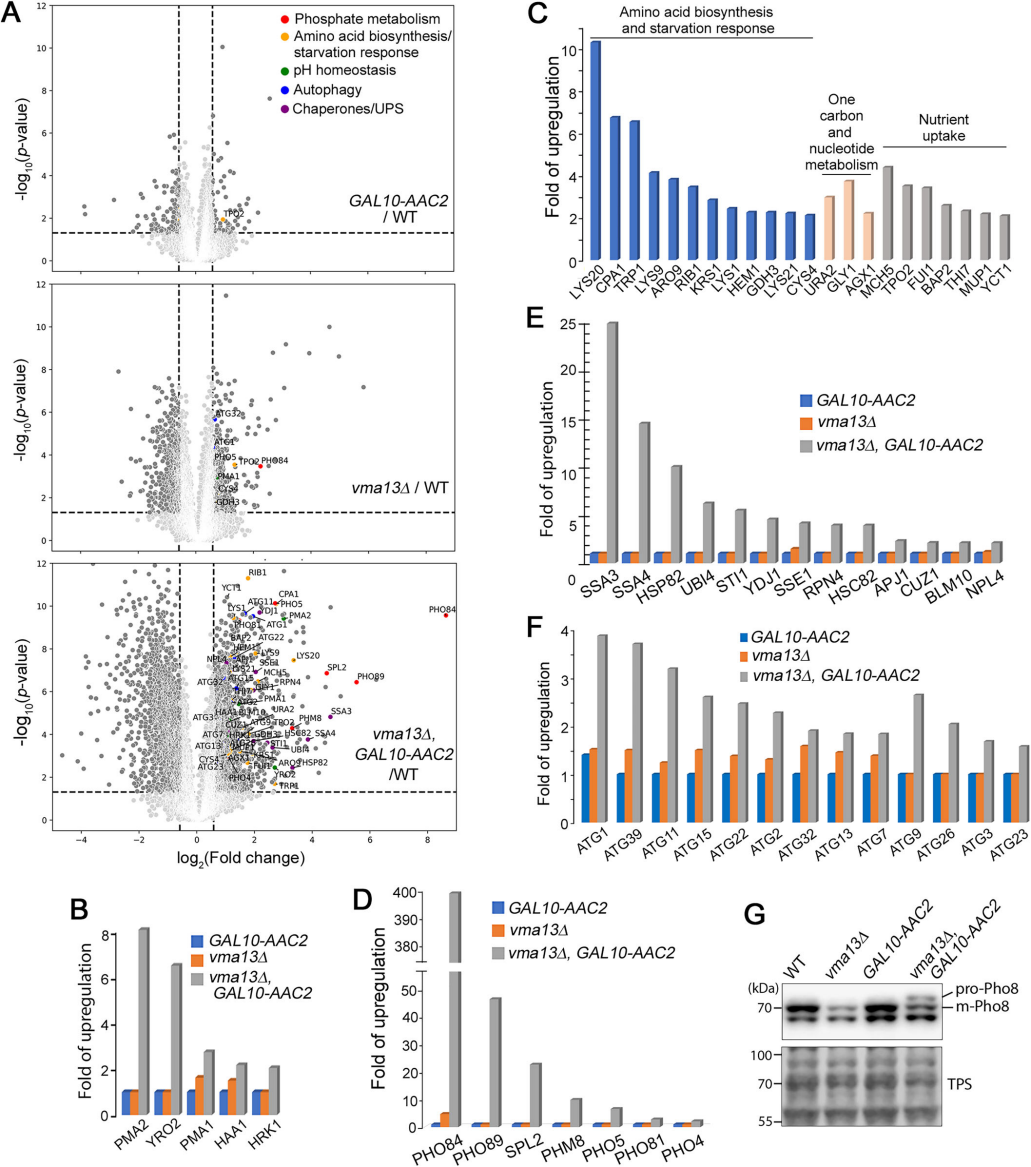
RNA-seq analysis showing that mitochondrial protein import stress synergizes with *vma13Δ* to activate adaptive genes (*n*-3). (**A**) Volcano plot showing activation of a large number of genes in *vma13Δ GAL10-AAC2* relative to *vma13Δ* or *GAL10-AAC2* cells. X-axis denotes adjusted *p* values from ANOVA tests. (**B**) Synergistic activation of genes in pH homeostasis. (**C**) Synergistic activation of genes involved in amino acid biosynthesis and starvation response, which was not observed in *vma13Δ* or *GAL10-AAC2* cells. (**D**) Synergistic activation of genes involved in phosphate homeostasis. (**E**) Synergistic activation of genes involved in chaperoning and proteasomal activities. (**F**) Synergistic activation of genes involved in autophagy. (**G**) Immunoblotting showing the accumulation of the Pho8 precursor (pro-Pho8) in *vma13Δ GAL10-AAC2* cells grown in YPGR medium. TPS total protein staining. Source data are available online for this figure.

Further support for the role of mitochondrial protein import stress in synergizing with *vma13Δ* to cause severe vacuolar damage came from transcriptomic analysis. The yeast vacuole plays a role not only in cellular pH control, protein degradation and autophagy, but also in iron, phosphate and amino acid homeostasis (Kane, 2007; Milgrom et al, 2007; Okreglak et al, 2023; Shirahama et al, 1996). In *GAL10-AAC2 vma13Δ* cells, we found that *AAC2* is overexpressed by only 2.4-fold in galactose medium, and this strongly activates many genes whose expression is either unchanged or only moderately activated in *GAL10-AAC2* or *vma13Δ* strains (Fig. 4A; Dataset EV1–3). These include (1) genes involved in cellular pH control (Fig. 4B), amino acid biosynthesis and starvation response, one-carbon metabolism and nutrient uptake (Fig. 4C), and phosphate homeostasis (Fig. 4D); and (2) those involved in cytosolic proteostasis (Fig. 4E) and autophagy (Fig. 4F), which supports mPOS in the cytosol and a potential role of autophagy in triaging the unimported mitochondrial proteins. Severe vacuolar damage in *GAL10-AAC2 vma13Δ* cells was further supported by the accumulation of the Pho8 precursor protein (Fig. 4G), an alkaline phosphatase that is proteolytically processed in the lumen of a functional vacuole. Defective Pho8 processing provides strong evidence for functional damage to the vacuole. Finally, we found that one extra copy of chromosomally integrated *AAC2* is sufficient to sensitize cells to hydroxyurea, an inhibitor of ribonucleotide reductase involved in dNTP biosynthesis (Appendix Fig. S1A). *AAC2* overexpression also stimulates the formation of an Ade4-based purinosome that facilitates purine biosynthesis (Takaine et al, 2025) (Appendix Fig. S1B). These observations support nucleotide dyshomeostasis as a consequence of vacuolar damage and dysfunction. In summary, the data provide further support for the idea that overloading by unimported mitochondrial proteins causes functional damage to the vacuole.

### Mitochondrial protein import stress in mice induces mPOS and muscle atrophy independent of bioenergetic deficiency and oxidative stress

**Figure EV2:**
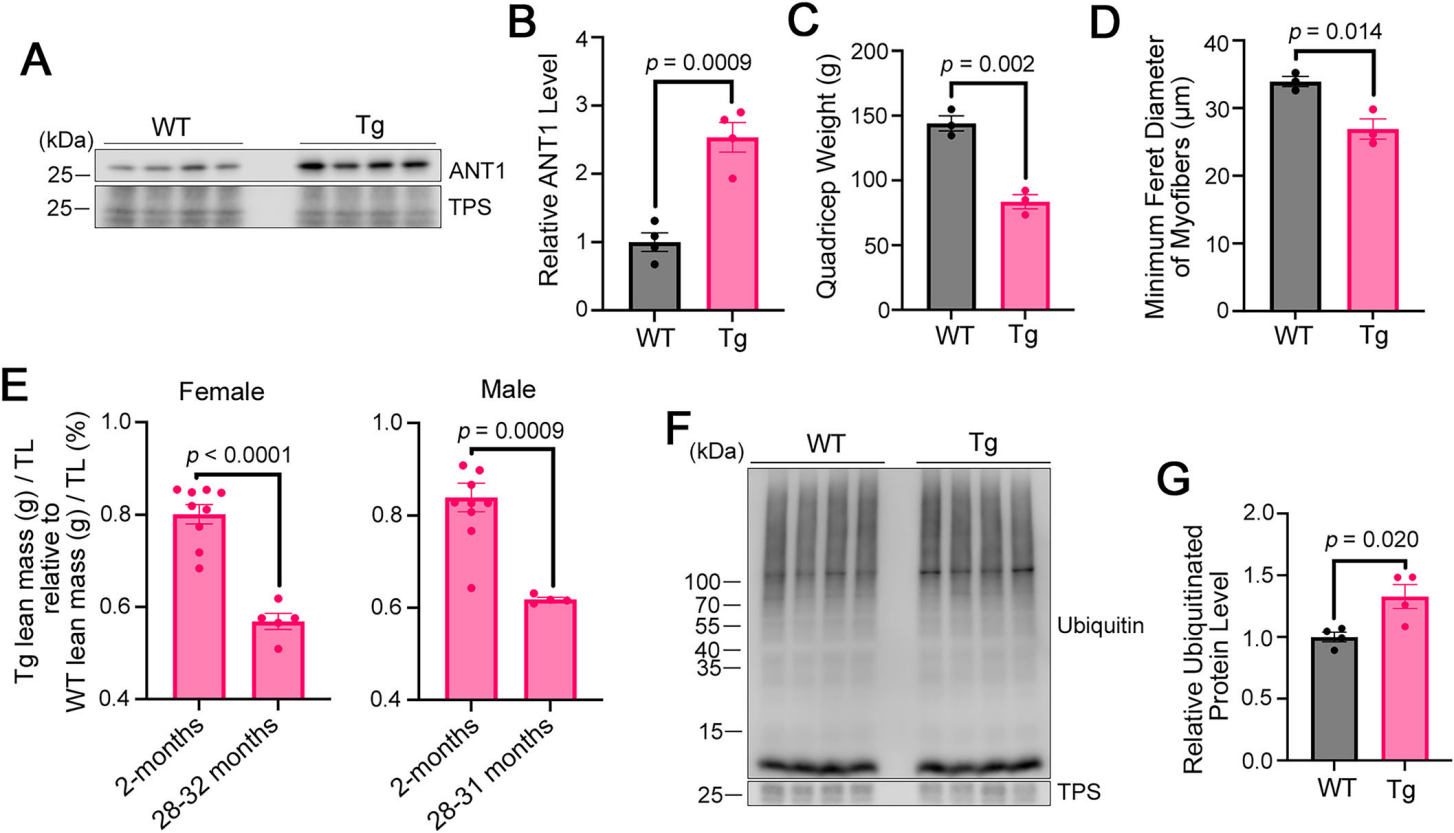
*Ant1* overexpression in *Ant1*^*Tg*^/+ mice causes progressive muscle atrophy during aging and affects quadriceps muscle weight, myofiber size, and protein ubiquitination levels at 2 months of age. (**A**) Immunoblotting showing ANT1 protein levels in *Ant1*^*Tg*^/+ (Tg) quadriceps relative to age-matched wild type controls (WT) at 2 months of age (*n* = 4/genotype; female). (**B**) Quantification of (**A**). (**C**) Quadriceps muscle weight at 2 months of age (*n* = 3/genotype; female). (**D**) Minimum Feret diameter of myofibers at 2 months of age (*n* = 3/genotype; female). (**E**) DXA scanning showing lean mass of *Ant1*^*Tg*^/+ mice relative to wild-type controls at different ages (*n* = 4–10/genotype/sex) normalized by tibia length (TL). (**F**) Immunoblotting of total cell lysates using an anti-ubiquitin antibody (*n* = 4/genotype; female). (**G**) Quantification of (**F**). Data in (**A**–**D**, **G**) are means ± SEM. The “*n*” denotes the number of animals. *P* values were calculated by a two-tailed Student’s *t-*test. TPS total protein stain.

**Figure 5:**
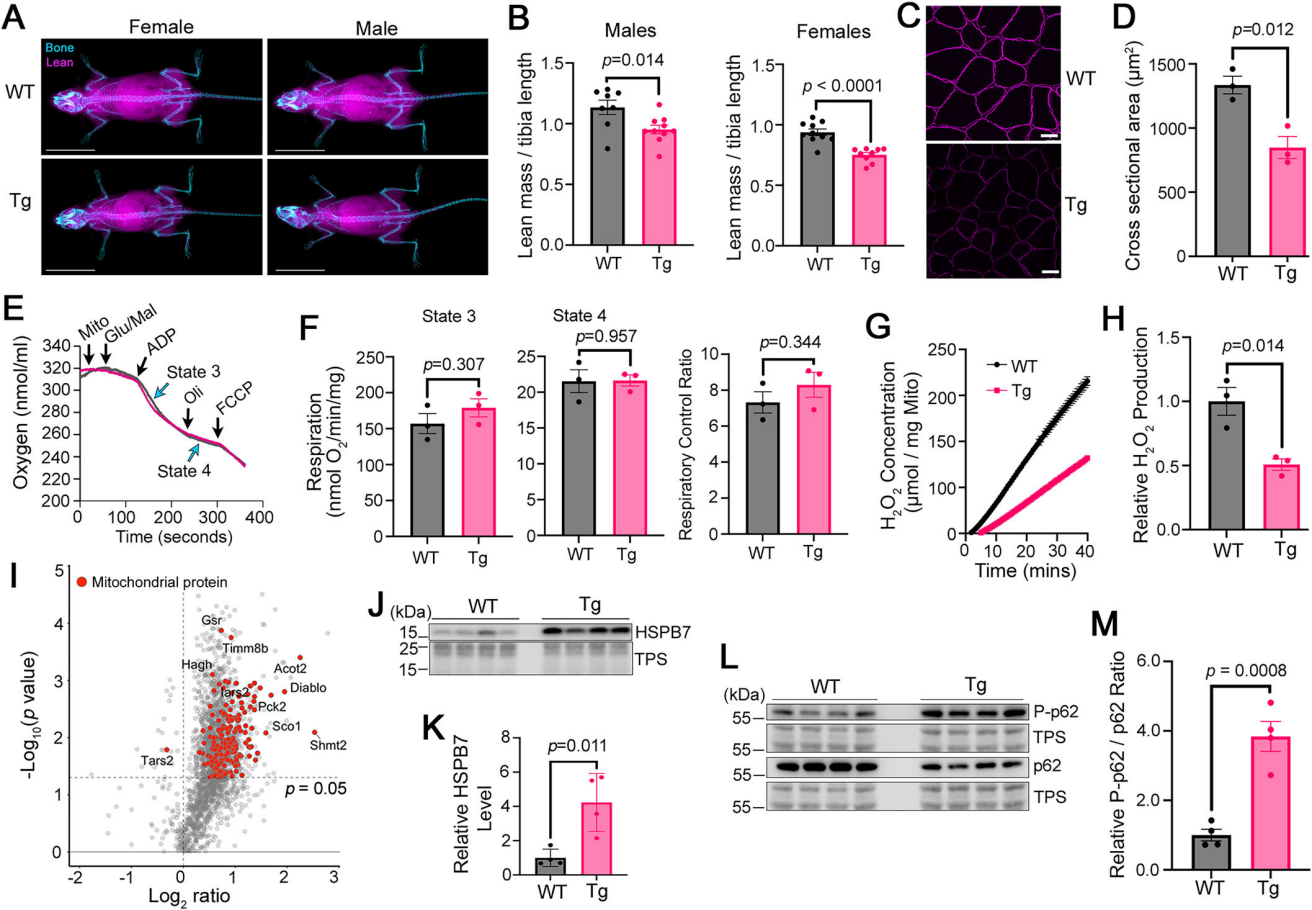
Mitochondrial protein import stress in *Ant1*^*Tg*^/+ muscle induces mPOS and muscle atrophy independent of bioenergetics. Quadriceps muscle was analyzed in two-month-old mice. (**A**) Body composition analysis by Dual-energy X-ray absorptiometry (DXA). Scale bar, 2.5 cm. (**B**) Lean mass quantification by DXA (*n* = 8–10/genotype/sex). (**C**) Immunofluorescence staining with anti-dystrophin antibody. Scale bar, 20 μm. (**D**) Cross-sectional area of myofibers (*n* = 3/genotype; female). (**E**) Representative traces of oxygraph analysis of isolated mitochondria. Mito mitochondria, Glu glutamate, Mal malate, Oli oligomycin, FCCP carbonyl cyanide-*p*-trifluoromethoxyphenylhydrazone. (**F**) Respiratory rates (*n* = 3/genotype; female). (**G**) Representative traces of H_2_O_2_ production from isolated mitochondria. (**H**) Quantification of H_2_O_2_ production (*n* = 3/genotype; female). (**I**) TMT mass spectrometry analysis of cytosolic fractions (*n* = 3/genotype; female). (**J**) Immunoblotting of HSPB7 (*n* = 4/genotype; female). (**K**) Quantification of (**J**). (**L**) Immunoblotting of P-p62 and p62 (*n* = 4/genotype; male). (**M**) P-p62/p62 ratio. Error bars represent mean ± SEM, and *p* values were calculated using unpaired two-tailed Student’s *t*-test in (**B**, **D**, **F**, **H**, **K**, **M**). Source data are available online for this figure.

To determine whether mPOS causes damage to the lysosome in mammals, we analyzed transgenic mice overexpressing ANT1, the functional homolog of yeast Aac2. These mice develop a muscle atrophy phenotype (Wang et al, 2022). To determine the primary stress that causes muscle atrophy, we analyzed young *Ant1*-transgenic (*Ant1*^*Tg*^/+) mice at the age of only 2 months. In these mice, the level of ANT1 is increased by only 2.5-fold in the skeletal muscle (Fig. EV2A,B). We found that these young mice already have significantly reduced lean mass (Figs. 5A,B and EV2C), myofiber cross-sectional area (Fig. 5C,D) and diameter (Fig. EV2D). Loss of lean mass progresses with age (Fig. EV2E). Interestingly, mitochondrial respiration (Fig. 5E,F) is little affected, like in yeast cells moderately overexpressing *AAC2* (Fig. EV1). Moreover, production of reactive oxygen species (ROS) is instead reduced in *Ant1*^*Tg*^/+ muscles (Fig. 5G,H). Muscle atrophy is therefore independent of bioenergetic deficiency and oxidative stress in *Ant1*^*Tg*^/+ mice.

We then analyzed the cytosolic proteome of *Ant1*^*Tg*^/+ muscle using Tandem Mass Tag (TMT)-based mass spectrometry. We found that the abundance of 1511 proteins is significantly elevated (ANOVA test, *p* < 0.05; Dataset EV4). Among them are 191 mitochondrial proteins (Fig. 5I), including ANT1 that is enriched by twofold (Dataset EV4). The retention of these proteins in the cytosol is consistent with mitochondrial protein import stress. We also observed an increase in the expression of *Hspb7*, based on both the proteomic data (Dataset EV4) and Western blot analysis (Fig. 5J,K). HSPB7 is a small heat shock family B chaperone that is known to provide the first line defense against proteostatic stress in the cytosol in an ATP-independent manner (Mogk et al, 2019). Also consistent with mPOS in the cytosol, the overall protein ubiquitination is increased in *Ant1*^*Tg*^/+ muscle (Fig. EV2F,G). Phosphorylation of p62, which enhances its activity as an autophagic receptor for protein aggregates, is also increased (Fig. 5L,M). Taken together, the data strongly support mPOS in the cytosol of *Ant1*^*Tg*^/+ muscle in the absence of significant changes to bioenergetics.

### Like in yeast, transcriptomic analysis reveals metabolic and lysosomal stress in the *Ant1*^*Tg*^/+ muscle

**Figure 6:**
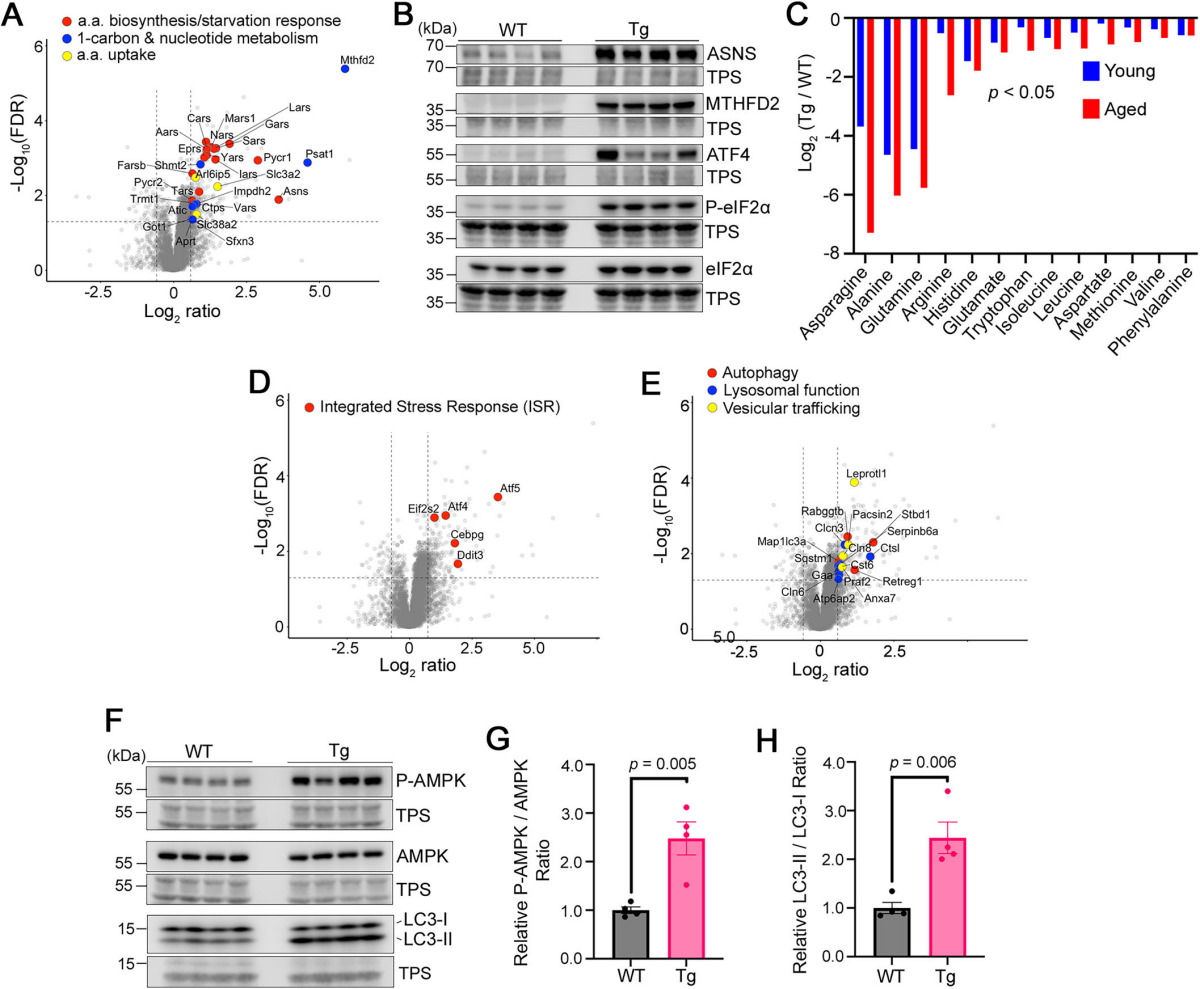
Mitochondrial protein import stress in *Ant1*^*Tg*^/+ muscle activates genes involved in ISR, metabolic adaptation and lysosome-related functions. Quadriceps muscle from two-month-old mice was analysed except for the inclusion of aged mice in (**C**). (**A**) RNA-seq analysis showing activation of metabolic genes in *Ant1*^*Tg*^/+ muscle. (**B**) Western blot analysis validating the activation of ISR and selected target genes. (**C**) Targeted metabolomic analysis showing depletion of amino acids in young (2 months) and old (27–30 months) *Ant1*^*Tg*^/+ muscle (*n* = 3-5/genotype; female). (**D**) Volcano plot showing the activation of ISR genes in *Ant1*^*Tg*^/+ muscle (*n* = 4). (**E**) RNA-seq analysis showing the activation of genes related to lysosomal functions in *Ant1*^*Tg*^/+ muscle (*n* = 4). (**F**) Immunoblotting showing increased AMPK phosphorylation and LC3-II accumulation in *Ant1*^*Tg*^/+ muscle (*n* = 4/genotype; male). (**G**) Quantification of relative P-AMPK/AMPK ratio (*n* = 4). (**H**) Quantification of relative LC3-II/LC3-I ratio (*n* = 4). The data were given as mean ± SEM. *P* values were calculated by a two-tailed Student’s *t*-test. TPS, total protein staining. Source data are available online for this figure.

**Figure EV3:**
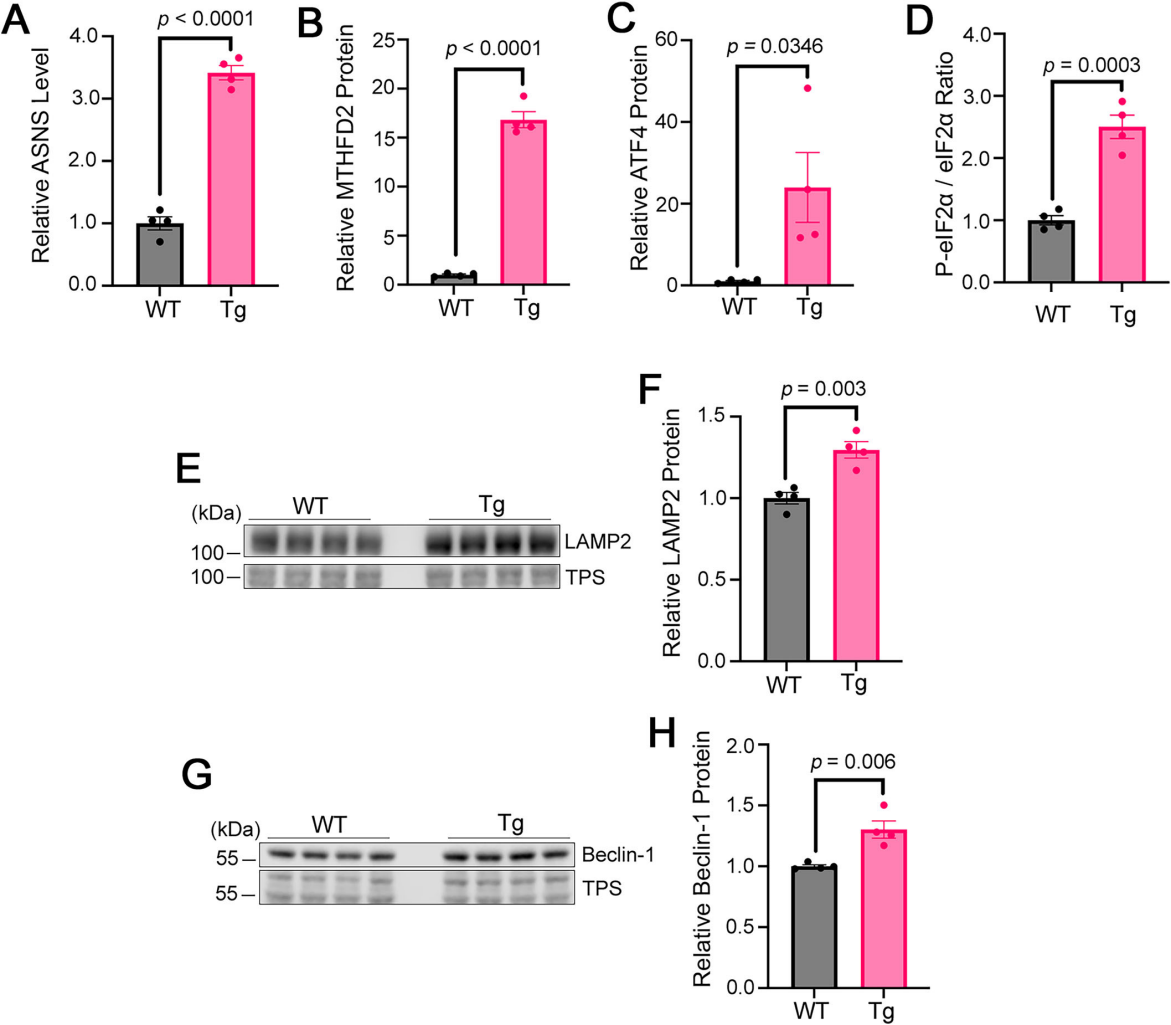
Immunoblot analysis validating transcriptional activation of ISR and autophagy-related genes in 2-month-old *Ant1*^*Tg*^/+ muscle. (**A**–**C**) Quantification of relative protein levels of ASNS (**A**), MTHFD2 (**B**) and ATF4 (**C**) (Fig. 6B). (**D**) Quantification of the relative P-eIF2α/eIF2α ratio (Fig. 6B). (**E**) Immunoblotting of LAMP2 (*n* = 4/genotype; male). (**F**) Quantification of (**E**). (**G**) Immunoblotting of Beclin-1 (*n* = 4/genotype; male). (**H**) Quantification of (**G**). TPS, total protein stain. Error bars are means ± SEM. *P* values were calculated by a two-tailed Student’s *t*-test. The “*n*” denotes the number of animals.

To determine the mechanism of muscle atrophy in *Ant1*^*Tg*^/+ mice, we compared the transcriptome of *Ant1*^*Tg*^/+ muscle and wild-type controls at 2 months of age. We found that 277 genes are upregulated by >1.5-fold (one-way ANOVA, *q* < 0.05; Dataset EV5). Among these genes are those involved in amino acid (a.a.) biosynthesis/starvation response, one-carbon and nucleotide metabolism, a.a. uptake (Fig. 6A; Appendix Fig. S2A) and iron homeostasis (Appendix Fig. S2B). Activation of representative genes in these functional groups, such as *Asns* and *Mthfd2* were validated by western blotting (Figs. 6b and EV3A,B). Supporting amino acid starvation response, numerous amino acids in the cytosolic fraction are increasingly depleted in aging *Ant1*^*Tg*^/+ muscle (Fig. 6C). We also found that genes encoding several key components of the Integrated Stress Response (ISR), including *Atf4, Atf5*, and *Ddit3*, are activated (Fig. 6D). These transcription factors including ATF4 (Figs. 6B and EV3C) are known to activate metabolic genes including *Asns* and *Mthfd2*. Activation of ISR was further confirmed by increased phosphorylation of eIF2α (Figs. 6B and EV3D), which increases the translation of ATF4, ATF5, and DDIT3 in a cap-independent manner (Pakos-Zebrucka et al, 2016). ISR activation is a hallmark of mitochondrial disease that directly affects oxidative phosphorylation (Dogan et al, 2014; Han et al, 2023; Hunt et al, 2019; Khan et al, 2017; Silva et al, 2009; Zurita Rendon and Shoubridge, 2018).

**Figure EV4:**
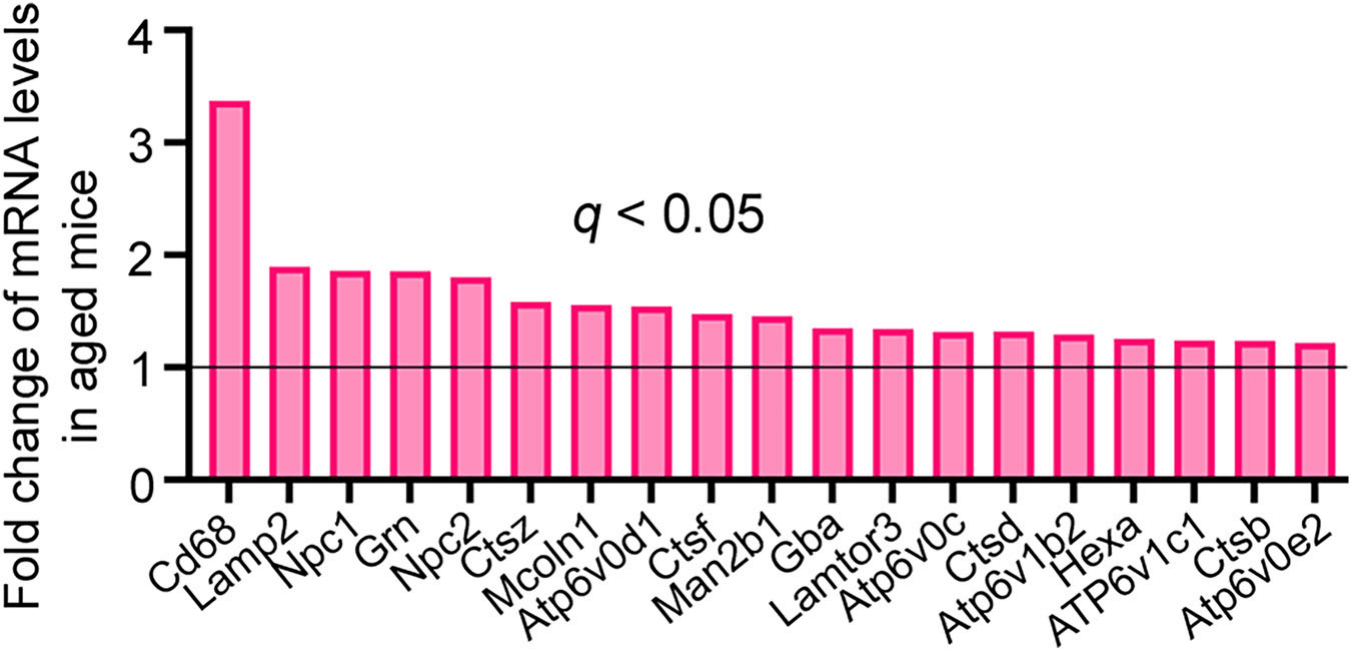
RNA-seq data showing the genes involved in lysosomal and autophagic functions that are activated in aged (27–30 months) *Ant1*^*Tg*^/+ quadriceps muscle relative to age-matched wild type controls (*n* = 4/genotype).

Finally, we found that genes involved in autophagy, lysosomal function and vesicular trafficking are also activated in *Ant1*-transgenic muscle (Fig. 6E). The number of activated genes involved in lysosomal function is increased in aged *Ant1*^*Tg*^/+ muscle (Fig. EV4). These include (1) five genes in the *Atp6* family encoding subunits of the V-ATPase, and (2) *Npc1*, *Npc2* and *Gba* known to be associated with lysosome storage disease. These observations suggest a cumulative increase in the severity of lysosomal damage. Further supporting lysosomal damage, the level of LAMP2, a commonly used marker of lysosomes and late endosomes, is increased (Fig. EV3E–F). Phosphorylation of AMP-activated protein kinase (AMPK) is increased (Fig. 6F,G), which is known to stimulate autophagy. The LC3-II/LC3-I ratio, which is commonly used as a quantitative measure of macroautophagy, is also increased in the *Ant1*^*Tg*^/+ muscle (Fig. 6F,H). The level of Beclin-1, another key regulator of autophagy, is also increased (Fig. EV3G,H). In summary, the data support activation of genes involved in lysosomal function and autophagy. These gene expression patterns remarkably resemble what was found in yeast mutants with severe vacuolar damage (see Fig. 3). *Ant1*-induced mitochondrial protein import stress also activates genes involved in a.a. biosynthesis/starvation response, one-carbon and nucleotide metabolism and a.a. uptake. This suggests metabolic stress, and is consistent with a role of the lysosome in nutrient recycling (Xu and Ren, 2015). Overall, the data strongly suggest that, like in yeast, mitochondrial protein import stress induces lysosomal stress in *Ant1*^*Tg*^/+ mice.

### Lysosomal damage and activation of repair pathways in *Ant1*^*Tg*^/+ muscle

**Figure 7:**
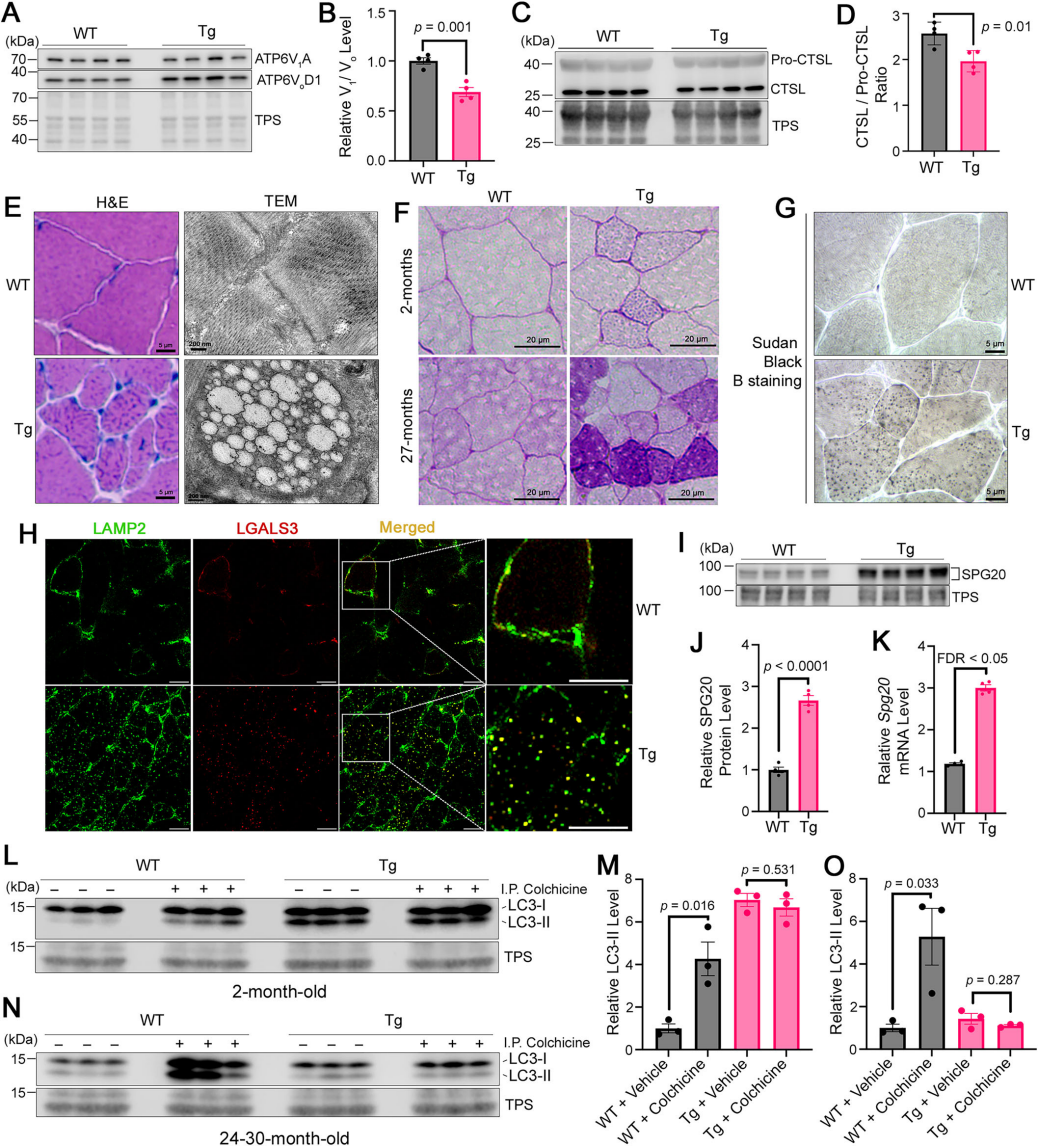
Mitochondrial protein import stress in *Ant1*^*Tg*^/+ muscle induces lysosomal damage, activates lysosomal repair pathways and stalls autophagic flux. Quadriceps muscle was analyzed in all the experiments. (**A**) Immunoblotting of V-ATPase subunits (*n* = 4/genotype; female). (**B**) Quantification of the relative V_1_/V_o_ ratio. (**C**) Immunoblotting of pro- and mature forms of CTSL (*n* = 4/genotype; female). (**D**) Quantification of relative CTSL/Pro-CTSL ratio. (**E**) H&E staining and transmission electron microscopy images showing the accumulation of basophilic vesicles and multivesicular body-like structures. (**F**) PAS staining showing glycogen accumulation in *Ant1*^*Tg*^/+ muscle. (**G**) Sudan Black B staining showing accumulation of lipofuscin in *Ant1*^*Tg*^/+ muscle from 30- to 32-month-old mice. (**H**) Immunofluorescence showing partial colocalization of LAMP2 and LGALS3 in 24-month-old *Ant1*^*Tg*^/+ muscle. Scale bar, 20 μm. (**I**) Immunoblotting of SPG20 (*n* = 4/genotype; female). (**J**) Quantification of (**I**). (**K**) RNA-seq analysis showing transcriptional activation of *Spg20* in *Ant1*^*Tg*^/+ muscle. (**L**) Western blot analysis showing the levels of LC3-I and LC3-II in the skeletal muscle of 2-month-old mice with or without colchicine treatment, establishing the autophagic flux in these animals. (**M**) Quantification of (**L**). (**N**) Western blot analysis showing the levels of LC3-I and LC3-II in the skeletal muscle of 24–30-month-old mice with or without colchicine treatment. (**O**) Quantification of (**N**). The data were given as mean ± SEM. *P* values were calculated by a two-tailed Student’s *t*-test. The “*n*” denotes the number of animals. TPS total protein staining. A 2-month-old quadriceps muscle was used for immunoblotting. Source data are available online for this figure.

**Figure EV5:**
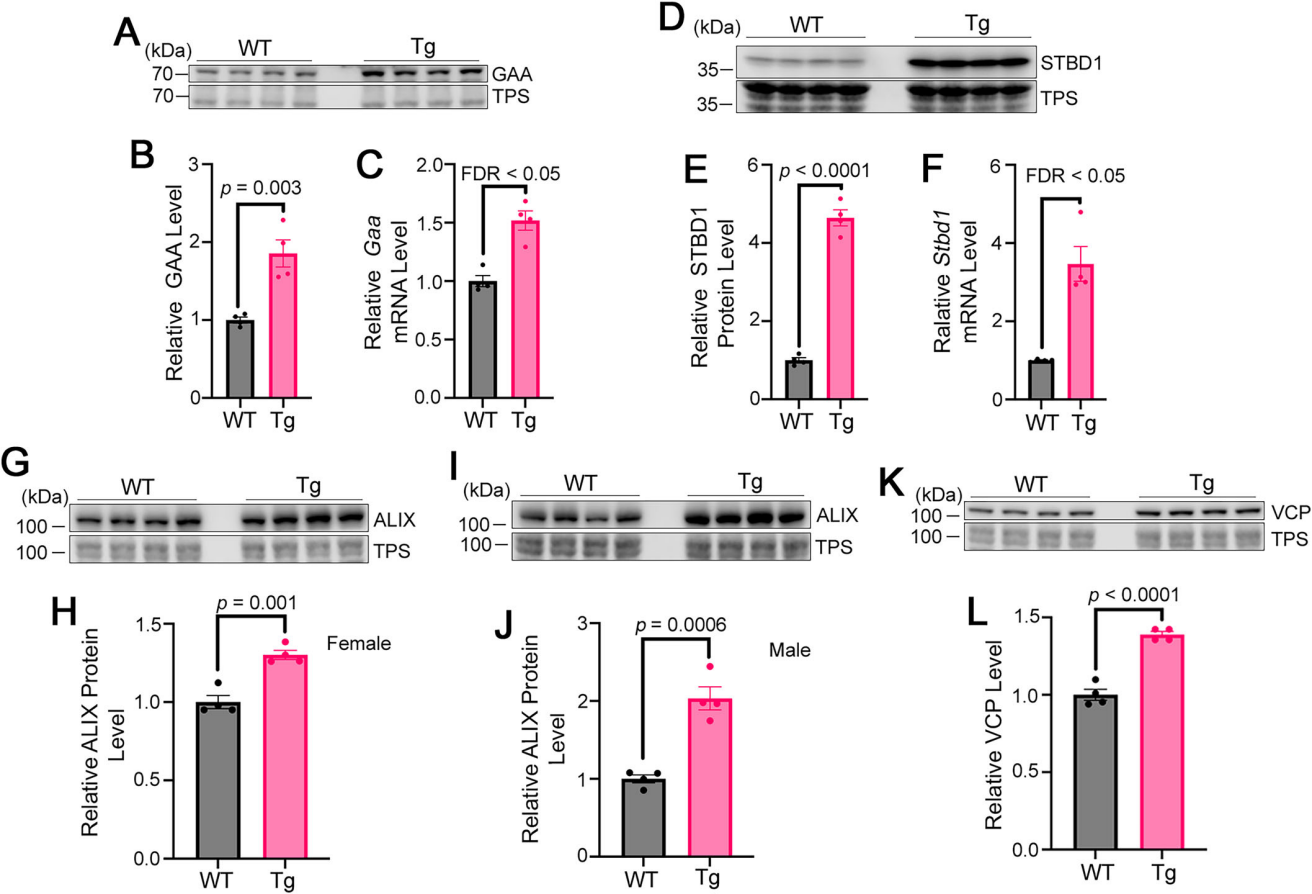
Immunoblotting showing the increased levels of lysosomal repair-related proteins in 2-month-old *Ant1*^*Tg*^/+ quadriceps muscle. (**A**) Immunoblotting of GAA (acid α-glucosidase) (*n* = 4/genotype; male). (**B**) Quantification of (**A**). (**C**) RNA-seq analysis showing transcriptional activation of *Gaa* in *Ant1*^*Tg*^/+ muscle (*n* = 4). (**D**) Immunoblotting of STBD1 (*n* = 4/genotype; male). (**E**) Quantification of (**D**). (**F**) RNA-seq analysis showing transcriptional activation of Stbd1 in *Ant1*^*Tg*^/+ muscle. (**G**) Immunoblotting of ALIX in female mice (*n* = 4/genotype). (**H**) Quantification of (**G**). (**I**) Immunoblotting of ALIX in male mice (*n* = 4/genotype). (**J**) Quantification of (**I**). (**K**) Immunoblotting of VCP (*n* = 4/genotype). (**L**) Quantification of (K). TPS total protein stain. Error bars are means ± SEM. *P* values were calculated by a two-tailed Student’s *t*-test. The “*n*” denotes the number of animals.

Multiple lines of evidence supported lysosomal damage in the *Ant1*^*Tg*^/+ muscle. First, we determined the abundance of the cytosolically faced V_1_ sector of the V-ATPase relative to the membrane-associated Vo (Fig. 1F) in lysosome-enriched subcellular fractions (Appendix Fig. S3). Reduced V_1_/V_o_ ratio in the lysosome-enriched pellet is commonly used as an indicator for the disassembly of the V-ATPase. We found that the V_1_/V_o_ ratio is moderately but significantly reduced in *Ant1*^*Tg*^/+ muscle compared with that of the wildtype (Fig. 7A,B), suggesting partial V_1_ dissociation or a defect in V-ATPase assembly, both of which would reduce V-ATPase activity. Second, the ratio between the mature and precursor (pro-) form of the lysosomal protease cathepsin L (CTSL) is reduced (Fig. 7C,D), supporting impaired lysosomal maturation. Third, H&E staining revealed the presence of basophilic vesicles in *Ant1*^*Tg*^/+ muscle (Fig. 7E), reminiscent of those seen in lysosomal myopathy (Malicdan et al, 2008). These profiles have approximately the same size as structures resembling poorly processed multivesicular bodies that may accumulate because of endolysosomal dysfunction, as revealed by transmission electron microscopy (TEM) (Fig. 7E). Fourth, we observed progressive overaccumulation of glycogen in *Ant1*^*Tg*^/+ muscle (Fig. 7F). Expression of α-glucosidase (GAA), the only glycogen-hydrolyzing enzyme in the lysosome is increased (Fig. EV5A–C), likely as a stress response. Along this line, the transcript and protein levels of STBD1, an autophagic receptor for glycophagy, are increased (Fig. EV5D–F). These data were consistent with lysosomal damage. Fifth, lipofuscin over-accumulates in *Ant1*^*Tg*^/+ muscle (Fig. 7G). Lipofuscin accumulation is not only a biomarker for reduced degradative capacity of lysosomes, but is also known to directly cause lysosomal membrane permeabilization (Pan et al, 2021).

Sixth, we found that lysosomal repair and turnover pathways are activated in *Ant1*^*Tg*^/+ muscle. Galectin-3, a glycan-recognizing protein involved in lysosomal repair (Jia et al, 2020), forms intracellular puncta that considerably colocalize with LAMP2, resembling lysosomal repair foci (Fig. 7H). The protein (Fig. 7I,J) and transcript (Fig. 7K) levels of SPG20 are both elevated. SPG20 is a recently identified protein involved in the initiation of lysophagy and lysosomal quality control (Gahlot et al, 2024). The levels of Alix and VCP, two additional proteins that participate in lysosomal repair (Papadopoulos et al, 2020; Radulovic et al, 2018; Skowyra et al, 2018), are also elevated (Fig. EV5G–L). The data provide further support for lysosomal damage and activation of repair pathways in *Ant1*^*Tg*^/+ muscle.

Despite the activation of genes involved in autophagy (see Fig. 6E), lysosomal damage would be expected to reduce the overall autophagic flux due to reduced trafficking and processing of autophagosomes. To test this, we determined autophagic flux in *Ant1*^*Tg*^/+ and wild-type muscle using colchicine to inhibit autophagosome processing. In the wild-type skeletal muscle, we found that injection of colchicine leads to the accumulation of the lipidated form of LC3, LC3-II, suggesting effective inhibition of autophagosome degradation by the lysosome. Indeed, in both young (Fig. 7L,M) and aged (Fig. 7N,O) *Ant1*^*Tg*^/+ muscle, LC3-II accumulated at the same level regardless of whether colchicine was delivered. The high basal level of LC3-II strongly suggests that autophagic flux is severely stalled in the *Ant1*^*Tg*^/+ muscle, likely due to lysosomal damage.

### Release of lysosomal hydrolytic enzymes and activation of proteasomal activities in *Ant1*^*Tg*^/+ muscle

**Figure 8:**
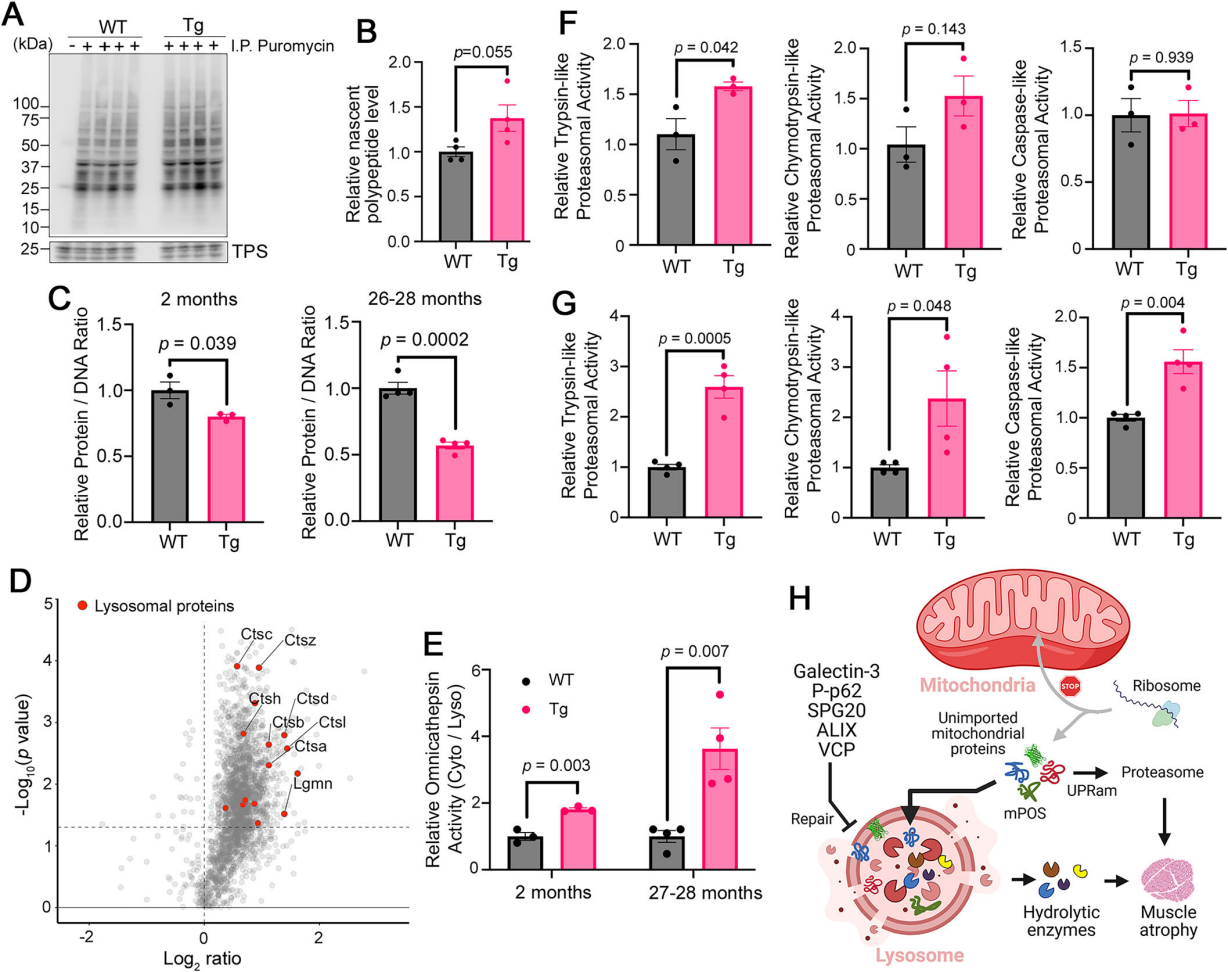
Mitochondrial protein import stress causes the release of cathepsins from lysosomes and stimulates proteasomal activity in *Ant1*^*Tg*^/+ quadriceps muscle. (**A**) In vivo protein synthesis measurement by SUnSET analysis of 2-month-old mice (*n* = 4/genotype; female). (**B**) Quantification of (**A**). (**C**) Relative protein/DNA ratio in *Ant1*^*Tg*^/+ and wild type muscles (*n* = 3–4/genotype; female). (**D**) TMT mass spectrometry analysis showing the increased levels of lysosomal hydrolytic proteases, including cathepsins, in the cytosolic fraction of *Ant1*^*Tg*^/+ muscle (*n* = 3/genotype; female). (**E**) Relative cytosolic/lysosomal omnicathepsin activity ratio in *Ant1*^*Tg*^/+ and wild type muscles (*n* = 3–4/genotype). (**F**) Proteasome-associated proteolytic activities in 2-month-old mice. (**G**) Proteasome-associated proteolytic activities in 27/28-month-old mice. (**H**) Model of mitochondria-induced progressive muscle atrophy. The release of hydrolytic enzymes from damaged lysosomes may cause excessive protein degradation and muscle atrophy in *Ant1*^*Tg*^/+ mice. Proteasomal activation may also play a role in muscle atrophy, especially in aged animals. Error bars are means ± SEM. *P* values were calculated by a two-tailed Student’s *t*-test. Source data are available online for this figure.

Muscle atrophy generally results from an imbalance between protein synthesis and degradation, when protein synthesis is decreased, and/or protein degradation is over-activated (Bonaldo and Sandri, 2013). Using the in vivo Surface Sensing of Translation (SUnSET) assay, we found that protein synthesis rate is marginally increased instead of decreased in *Ant1*^*Tg*^/+ muscle compared to the wildtype (Fig. 8A,B). In contrast, the relative protein content in *Ant1*^*Tg*^/+ muscle is drastically reduced relative to wildtype, with the most pronounced effect observed in aged mice (Fig. 8C). This suggests that muscle atrophy is likely caused by increased proteolysis in *Ant1*^*Tg*^/+ mice. The stalling of autophagic flux described above excludes the role of autophagy as the main driver of muscle atrophy. We then examined whether lysosomal damage leads to the release of luminal hydrolytic enzymes into the cytosol to cause excessive protein degradation. Indeed, proteomic examination revealed that lysosomal luminal proteins, including numerous hydrolytic proteases, are significantly enriched in the cytosol of *Ant1*^*Tg*^/+ muscle relative to the wild type controls (Fig. 8D). Direct enzymatic assay revealed that omnicathepsin activity, which reflects total activity of various lysosomal cathepsins, is already significantly increased in the cytosol of young *Ant1*^*Tg*^/+ muscle (Fig. 8E). The cytosolic cathepsin activities are further increased in aged *Ant1*^*Tg*^/+ muscle. The data support lysosomal membrane damage and the release of luminal proteases into the cytosol, which may cause excessive protein degradation, thereby contributing to muscle atrophy.

Previous studies have shown that accumulation of unimported mitochondrial protein activates the proteasomal branch of proteolysis (Wrobel et al, 2015). As such, we determined proteasomal activities in *Ant1*^*Tg*^/+ muscles. We found that, at the age of 2 months, the trypsin-like activity was marginally increased, whereas the chymotrypsin-like and caspase-like activities were not increased in the *Ant1*^*Tg*^/+ muscle compared to wild type (Fig. 8F). At 27–28 months of age, all three proteasome-associated protease activities were significantly increased in *Ant1*^*Tg*^/+ muscle compared to wild type controls (Fig. 8G). Therefore, it is possible that proteasomal activation also contributes to reduced protein content and muscle atrophy, especially in aged *Ant1*^*Tg*^/+ mice.

## Discussion

Under conditions of mitochondrial damage, accumulation of unimported mitochondrial proteins causes mPOS, which activates proteasomal function and represses global protein synthesis to contain cytosolic proteotoxicity (Wang and Chen, 2015; Wrobel et al, 2015). Studies have shown that accumulation of unimported mitochondrial proteins also activates other stress response programs to improve mitochondrial protein import and cellular proteostasis (Martensson et al, 2019; Nargund et al, 2012; Sutandy et al, 2023; Weidberg and Amon, 2018). To alleviate cellular stress, some unimported mitochondrial proteins are shuttled to the endoplasmic reticulum before being recycled back for mitochondrial import (Hansen et al, 2018), or are trafficked to the nucleus for degradation (Shakya et al, 2021). An outstanding question has been which cellular processes are most vulnerable to mPOS, leading to reduced cell fitness and viability. In the current study, we show that mitochondrial protein import stress causes vacuolar/lysosomal damage, which leads to inhibition of cell growth in yeast and progressive muscle atrophy in mice. We discovered a mitochondria-to-lysosome axis of proteostatic relay that affects tissue mass homeostasis.

In yeast, we found that mild mitochondrial protein import stress, induced either by Aac2 overloading or by the loss of non-essential components of the mitochondrial protein import pathway, including Tim18, Tom7, Tom5, Tom6, and Tom70, is synthetically lethal with *vma* mutations that disrupt V-ATPase activity. Aac2 overloading and mutations in the protein import genes lead to partial disassembly of the V-ATPase, vacuolar deacidification, and hypersensitivity to a high pH environment. Mitochondrial protein import stress can therefore intrinsically affect vacuolar function. When combined with *vma13Δ*, mitochondrial protein import stress causes drastic deacidification of the vacuole. The luminal pH reaches a level much higher than that observed in *vma13Δ* single mutant. This strongly suggests that mitochondrial protein import stress can also induce vacuolar damage independent of V-ATPase activity. Severe vacuolar damage caused by *AAC2*-overexpression and *vma13Δ* results in strong activation of genes involved in cellular pH homeostasis, including *PMA2*, which mediates proton efflux at the plasma membrane. Many genes involved in cytosolic proteostasis and autophagy are also activated in these cells. These data suggest defective autophagy and heightened proteostatic stress in the cytosol, because of vacuolar damage. Finally, numerous genes involved in amino acid biosynthesis, one-carbon metabolism and phosphate homeostasis are activated in *vma13Δ* cells challenged by Aac2 overloading. Given the important role of the vacuole in these metabolic processes, the data therefore provide further support for mitochondria-induced vacuolar damage. Previous studies have shown that vacuolar damage causes mitochondrial dysfunction (Hughes and Gottschling, 2012; Hughes et al, 2020). In light of our study, mitochondrial dysfunction can, in turn, induce vacuolar damage through proteostatic signaling. It is possible that a vicious cycle may occur, which ultimately causes the loss of cell viability.

Like in yeast, ANT1 overloading in the skeletal muscle of *Ant1*^*Tg*^/+ mice strongly activates genes involved in amino acid starvation response, one-carbon metabolism and autophagy. We speculate that these transcriptional responses may also be triggered by damage to the mammalian vacuolar counterpart, the lysosome. Lysosomal dysfunction and damage in the *Ant1*^*Tg*^/+ muscle is strongly supported by increased accumulation of glycogen, lipofuscin and poorly processed multivesicular bodies, severely stalled autophagy, and activation of genes involved in lysosomal biogenesis and repair. These data therefore support an evolutionarily conserved mechanism by which mitochondrial protein import stress induces vacuolar/lysosomal damage.

We found that ANT1 overloading causes significant muscle atrophy in two-month-old *Ant1*^*Tg*^/+ mice. In these young animals, OXPHOS capacity is little affected, whereas ROS production is reduced in the skeletal muscle. The data supported lysosomal but not bioenergetic and oxidative stress as a driver of muscle atrophy. Muscle atrophy occurs under many clinical conditions and during aging. A prevailing mechanism maintains that excessive protein degradation relative to synthesis drives muscle atrophy, by reducing the overall protein content and therefore the size of myofibers (Bonaldo and Sandri, 2013). Mitochondrial dysfunction is known to cause muscle atrophy (Calvani et al, 2013; Chen et al, 2010; Romanello and Sandri, 2016). Bioenergetic deficiency and oxidative stress have long been proposed as key drivers of mitochondria-induced muscle atrophy, despite the underlying mechanisms remaining vague. Our study supports the role of mPOS and lysosomal damage in mitochondria-induced muscle atrophy. We found that the overall rate of protein synthesis is not reduced in *Ant1*^*Tg*^/+ muscle. Therefore, muscle atrophy in *Ant1*^*Tg*^/+ mice is likely a result of excessive protein degradation. As the autophagic flux is severely stalled, it is unlikely that increased autophagy accounts for the muscle atrophy phenotype. Interestingly, we found that numerous lysosomal proteolytic enzymes are released into the cytosol. Although some of these enzymes, such as the cathepsins, have optimal activity at the low pH of the lysosomal lumen, they often retain some levels of proteolytic activity in the cytosol to degrade proteins (Yadati et al, 2020). Long-term exposure to these limited levels of cathepsin activities may cause excessive protein degradation, reduced myofiber size and ultimately, muscle atrophy (Fig. 8H). Interestingly, we also found that proteasomal activity is mildly increased in young, but significantly increased in aged *Ant1*^*Tg*^/+ muscle. This observation is reminiscent of the UPRam (Unfolded Protein Response activated by mistargeting of proteins) mechanism in cells challenged by severe mitochondrial protein import stress (Topf et al, 2016; Wrobel et al, 2015). It is therefore possible that proteasomal activation also contributes to muscle atrophy, especially in aged *Ant1*^*Tg*^/+ mice. Overall, we favor the model that excessive protein degradation by lysosome-released proteolytic enzymes and proteasomal activation cause progressive muscle atrophy, although it cannot be completely excluded that lysosomal damage and proteasomal activation may contribute to muscle atrophy via mechanisms other than proteostatic imbalance.

We found that the nuclear transcriptome is drastically remodeled even in young *Ant1*^*Tg*^/+ mice. Many genes involved in ISR, amino acid starvation response and one-carbon metabolism are activated. Of note, the activation of these genes is a common hallmark observed in various mitochondrial disease models (Dogan et al, 2014; Han et al, 2023; Khan et al, 2017; Kuhl et al, 2017; Tyynismaa et al, 2010). Given that OXPHOS capacity is not significantly reduced and ROS production is instead reduced in young *Ant1*^*Tg*^/+ muscle compared to wild type, our data strongly support the conclusion that the transcriptional response and metabolic remodeling are independent of bioenergetic impairment. OXPHOS-independent activation of ISR and other adaptive transcriptional responses have previously been observed in a mouse model of respiratory deficiency in the heart (Dogan et al, 2014). In light of our data, lysosomal damage may trigger the activation of metabolic remodeling, given the critical role of the lysosome in nutrient recycling and homeostasis.

How the unimported mitochondrial proteins are targeted and cause damage to the vacuole/lysosome remains unsolved. Autophagy may be involved in the delivery of these proteins into the vacuole/lysosome. In a *vma* mutant with reduced degradative capacity, we detected vacuolar accumulation of unimported mitochondrial proteins and vacuolar enlargement in yeast. We showed the vacuolar targeting of the matrix Aco1 protein under mitochondrial protein import stress conditions. Accumulation of poorly processed multivesicular bodies was observed in *Ant1*^*Tg*^/+ muscle. These observations suggest that at least some of the unimported mitochondrial proteins are targeted to the vacuole/lysosome for proteolytic processing. The mitochondrial proteome comprises many highly hydrophobic membrane proteins. Whether these proteins are targeted and effectively degraded within the vacuolar/lysosomal lumen is yet to be determined. In classic lysosomal storage disease, specific substrates such as lipids and complex sugar molecules over-accumulate inside the lysosome, causing functional and structural damage to the organelle. By analogy, we speculate that increased trafficking of unimported mitochondrial proteins may overwhelm the degradative capacity, thereby leading to vacuolar/lysosomal dysfunction. Alternatively, specific mitochondrial proteins may cause vacuolar/lysosomal membrane damage. The pathogenic overaccumulation of undigested substrates and aggregated proteins such as α-synuclein and tau within the lysosomal lumen, followed by lysosomal damage and dysfunction, is commonly observed in classic lysosomal storage, Parkinson’s disease (PD) and Alzheimer’s disease (AD) (Kakuda et al, 2024; Rose et al, 2024; Zoncu and Perera, 2022). Lysosomal membrane permeabilization and cathepsin release have also been observed in AD (Lee et al, 2022). Biomarkers of lysosomal damage, including the release of luminal enzymes and activation of repair pathways, were observed in the *Ant1*^*Tg*^/+ muscle, supporting the presence of lysosomal membrane damage. Further studies will be required to elucidate the underlying mechanisms.

In summary, our study uncovered a mitochondria-to-lysosome axis of proteostatic relay that causes tissue atrophy. Defective mitochondrial protein import can be induced by many types of mitochondrial injuries (Coyne and Chen, 2026). It may accelerate aging (Fortney et al, 2015; Gottschalk et al, 2014; Shadyab et al, 2016) and play a role in brain atrophy (Burggren et al, 2017; Chiba-Falek et al, 2018; Mise et al, 2017; Roses et al, 2016; Roses et al, 2009; Schott et al, 2016; Valant et al, 2012; Yu et al, 2017a; Yu et al, 2017b; Zeitlow et al, 2017). Our study may help in the better understanding of tissue atrophy during aging and also many diseases in which mitochondrial and lysosomal damage are co-manifested.

## Methods

**Table:** Reagents and tools table

Reagent/resource	Reference or source	Identifier or catalog number
**Experimental models**		
Yeast strains	Table EV1	
*Ant1*^*Tg*^/+ mouse	Wang et al, 2022	
C57BL/6J	Jackson Lab	#000664
**Recombinant DNA**		
pHluorin	Diakov et al, 2013	N/A
pMitoLoc plasmid	Adagene	#58980
**Antibodies**		
Mouse monoclonal ALIX	Invitrogen	#MA1-83977
Mouse monoclonal anti-galectin-3	Santa Cruz Biotechnology	#32790
Mouse monoclonal Pho8 antibody	Kane lab.	N/A
Mouse monoclonal anti-LAMP2	DSHB	#GL2A7
Mouse monoclonal anti-puromycin	Millipore	#MABE343
Mouse monoclonal S6	CST	#2317
Rabbit polyclonal anti-AMPKα	CST	#2532
Rabbit anti Ant1	Chen Lab	N/A
Rabbit polyclonal anti-ASNS	Proteintech	#14681-1-AP
Rabbit monoclonal anti-ATP6V1A	Abcam	#ab199326
Rabbit polyclonal anti-ATP6V0D1	Proteintech	#18274-1-AP
rabbit monoclonal anti-Beclin-1	CST	#3495
Rabbit polyclonal anti-eIF2α	CST	#9722
Rabbit polyclonal anti-GAA	Invitrogen	#PA5-117464
Rabbit polyclonal anti-HSPB7	Invitrogen	#PA5-100825
Rabbit polyclonal anti-LC3B	Novus Biologicals	#NB100-2220
Rabbit polyclonal anti-MTHFD2	Proteintech	#12270-1-AP
Rabbit monoclonal anti-P-AMPKa	CST	#2535
Rabbit polyclonal anti-P-S6	CST	#2211
Rabbit monoclonal anti-P-SQSTM1/p62	CST	#39786
rabbit monoclonal SQSTM1/p62	CST	#23214
Rabbit polyclonal anti-SPG20	Proteintech	#13791-1-AP
Rabbit polyclonal anti-STBD1	Proteintech	#11842-1-AP
Rabbit monoclonal anti-VCP	CST	#2649
Rat monoclonal anti-LAMP2	DSHB	#ABL-93
**Oligonucleotides and other sequence-based reagents**		
PCR primers	This study	N/A
**Chemicals, enzymes and other reagents**		
AllPrep DNA/RNA/Protein Mini Kit	Qiagen	#80004
Amplex UltraRed	Invitrogen	#A36006
Fatty acid-free BSA	Roche	#03117057001
BCECF-AM	Invitrogen	#B1150
FM 4-64	Thermo Fisher Scientific	#T13320
Halt Protease and Phosphatase Inhibitor Cocktail	Thermo Fisher Scientific	#78440
Monensin	Sigma-Aldrich	#M5273
Nigericin	Sigma-Aldrich	#N7143
Nourseothricin sulfate	GoldBio	#N-500
PVDF membrane	Millipore	#IPVH00010
Revert™ 700 Total Protein Stain	LI-COR	#926-11021
NativePAGE 3–12% Bis-Tris Gel	Invitrogen	#BN1001BOX
ProLong Gold Antifade Mountant	Invitrogen	#P36934
Protein Assay Dye Reagent Concentrate	Bio-Rad	#5000006
Schiff’s reagent	Electron Microscopy Sciences	#26853
Sudan Black B	Sigma-Aldrich	#199664
Zymolyase-20T	Amsbio	#120491-1
**Software**		
ImageJ	https://imagej.nih.gov/ij/index.html	N/A
GraphPad Prism	https://www.graphpad.com/	N/A
**Other**		
LI-COR Odyssey XF imager	https://www.licorbio.com/odyssey-xf	N/A
Oxygraph System	Hansatech Instruments Ltd	DW1/AD Electrode Chamber

### Yeast strains, growth media, and genetic manipulation

All the strains are in the BY4741/4742 background. Genotypes of yeast strains used in this study are listed in Table EV1. Yeast cells were cultured using standard media. Complete medium (YP) containing 1% Bacto yeast extract and 2% Bacto peptone is supplemented with 2% glucose (YPD) or 2% galactose plus 2% raffinose (YPGR), and tryptophan (20 μg/ml). Yeast minimal medium (YNBD) contains 0.67% Difco yeast nitrogen base without amino acids supplemented with 2% glucose. Supplements essential for auxotrophic strains were added at 20 μg/ml for bases and amino acids except for leucine (30 μg/ml) and lysine (30 μg/ml). The pMitoLoc plasmid (#58980, Adagene) was transformed into yeast strains and selected on YPD supplemented with 200 mg/ml nourseothricin sulfate (#N-500, GoldBio) as previously described (Vowinckel et al, 2015). The *vma1Δ*, *vma11Δ*, *vma12Δ*, and* vma13Δ* mutants were from the BY4741 knockout library and introduced in AN1 and AG3 strains by genetic crosses. Standard methods were used for mating, sporulation and segregational phenotyping of yeast strains. At least two to three independent zygotes were analyzed for segregational studies.

### Yeast vacuole membrane staining with FM 4-64

Yeast cells were grown in YPGR supplemented with tryptophan (20 μg/ml) at 30 °C until mid-log phase. Cells were subcultured in YPGR supplemented with tryptophan (20 μg/ml) and grown for 4 h at 30 °C. Cells equivalent to 1.0 OD_600_ were pelleted and resuspended in 1 mL of YPGR supplemented with tryptophan (20 μg/ml) and FM 4-64 at a final concentration of 8 μM. Cells were then shaken gently in the dark at 30 °C for 30 min. Cells were washed with YPGR supplemented with tryptophan (20 μg/ml) to remove free FM4-64. Cells were resuspended in 1 ml of YPGR supplemented with tryptophan (20 μg/ml) and then shaken gently in the dark at 30 °C for 75 min. The cells were then pelleted and resuspended in 50 μL YNBGR (YNB plus 2% galactose and 2% raffinose) supplemented with tryptophan (20 μg/ml). Finally, cells were examined under a fluorescent microscope, and vacuolar diameter was measured using ImageJ/Fiji imaging software. At least two independent experiments were performed to ensure reproducibility.

### Measurement of yeast vacuolar pH

Yeast cells were grown in 5 ml of YPD supplemented with tryptophan (200 μg/ml) at 30 °C overnight. The cells were then subcultured into 50 ml of YPGR + tryptophan (200 μg/ml) and grown for ~24 h at 30 °C to OD_600_ = ~1.0. Cells were collected by centrifugation and washed twice with YP. The cell pellet was weighed and resuspended in YP to a final density of 0.5 g/ml (w/v). BCECF-AM (#B1150, Invitrogen) was added to a final concentration of 50 μM. After incubation with gentle shaking at 30 °C for 30 min, the cells were pelleted and washed twice with YP before being resuspended in YP to a final density of 0.5 g/ml (w/v). Cells were kept on ice for a calibration curve and measurement of vacuolar pH.

The calibration buffer contains 50 mM MES (2-(*N*-morpholino) ethanesulfonic acid), 50 mM HEPES (4-(2-hydroxyethyl)-1-piperazineethanesulfonic acid), 50 mM KCl, 50 mM NaCl, 200 mM ammonium acetate, 10 mM sodium azide, and 10 mM 2-deoxyglucose, and is adjusted to pH from 5 to 7 with HCl or NaOH. Monensin (#M5273, Sigma-Aldrich) and nigericin (#N7143, Sigma-Aldrich) was added to 2 ml calibration buffer of each pH point at a final concentration of 110 and 15 μM, respectively. About 15 μl of cell suspension was added to each pH calibration tube. The cell mix was shaken gently at 30 °C for at least 1 h.

For vacuolar pH measurement, 15 μl of cell suspension was added to 2 ml of 1 mM MES, pH 5.0, in a cuvette. The fluorimeter was set to measure alternately at excitation wavelength 450 and 490 nm, both with an emission wavelength of 535 nm. The measurements were performed at 30 °C with continuous stirring of the mixture in the cuvette. One min after initiation of fluorescence measurement, glucose was added to the stirring mix at a final concentration of 50 mM. For continuous kinetic data, fluorescence was measured every 6 s. For the calibration curve, the mix of each pH point was measured using the same settings.

The fluorescence ratio of 490 to 450 nm was calculated for each pH calibration mixture. A calibration curve was obtained by plotting the fluorescence ratio vs. pH. The pH of each strain was obtained by calculating the fluorescence ratio and using the pH calibration curve. Vacuolar pH was plotted against time (kinetics of pH changes) and then compared in different strains.

### Measurement of yeast cytosolic pH

Yeast strains were transformed with the pHluorin plasmid (Diakov et al, 2013) by selecting for Ura^+^ cells. Cells were grown in 5 ml of YNBDCas supplemented with tryptophan (200 μg/ml) at 30 °C overnight. The cells were then subcultured into 50 ml of YNBGRCas supplemented with tryptophan (200 μg/ml) and grown for ~24 h at 30 °C to OD_600_ = ~1.0. Cells were collected by centrifugation and washed twice with YNBCas. The cell pellet was weighed and resuspended in YNBCas to a final density of 0.5 g/ml (w/v). Cells were kept on ice for a calibration curve and measurement of cytosolic pH.

The calibration buffer contains 50 mM MES (2-(*N*-morpholino) ethanesulfonic acid), 50 mM HEPES (4-(2-hydroxyethyl)-1-piperazineethanesulfonic acid), 50 mM KCl, 50 mM NaCl, 200 mM ammonium acetate, 10 mM sodium azide, and 10 mM 2-deoxyglucose, and is adjusted to pH from 6 to 8 with HCl or NaOH. Monensin (#M5273, Sigma-Aldrich) and nigericin (#N7143, Sigma-Aldrich) was added to 2 ml calibration buffer of each pH point at a final concentration of 110 and 15 μM, respectively. About 15 μl of cell suspension was added to each pH calibration tube. The cell mix was shaken gently at 30 °C for at least 1 h. For cytosolic pH measurement, 15 μl of cell suspension was added to 2 ml of 1 mM MES, pH 5.0, in a cuvette. The fluorimeter was set to measure alternately at excitation wavelength 405 and 485 nm, both with an emission wavelength of 508 nm. The measurements were performed at 30 °C with continuous stirring of the mixture in the cuvette. One min after initiation of fluorescence measurement, glucose was added to the stirring mix at a final concentration of 50 mM. For kinetic data, fluorescence was measured every 6 s. For the calibration curve, the mix of each pH point was measured using the same settings.

The fluorescence ratio of 405 to 485 nm was calculated for each pH calibration mixture. A calibration curve was obtained by plotting the fluorescence ratio vs. pH. The pH of each strain was obtained by calculating the fluorescence ratio and using the pH calibration curve. Vacuolar pH was plotted against time (kinetics of pH changes) and then compared in different strains.

### Immunoblotting analysis

Cell lysates were subject to SDS-PAGE and then transferred to a PVDF membrane (#IPVH00010, Millipore). For protein loading control, total protein stain (#926-11021, LI-COR) was used according to the manufacturer’s protocol. Standard procedures were used for chemiluminescent Western blot detection of proteins using a LI-COR Odyssey XF imager. Signals were quantified using ImageStudio software.

For yeast, cell lysates were prepared as previously described (Chen, 2001). The mouse monoclonal Pho8 antibody (1:400) was from the Kane laboratory.

For mouse studies, 30–50 mg of snap-frozen quadriceps muscle tissues were minced and homogenized in 0.3 mL of ice-cold RIPA buffer (20 mM Tris-HCl, pH 7.4; 150 mM NaCl; 1 mM EDTA, pH 7.4; 1% Triton X-100; 1% sodium deoxycholate; 0.1% SDS) supplemented with 1x Halt Protease and Phosphatase Inhibitor Cocktail (#78440, Thermo Fisher Scientific). Cellular debris was removed by centrifugation at 16,000×*g* for 5 min at 4 °C. Muscle lysates were then mixed with Laemmli buffer and heated at 42 °C for 30 min prior to SDS-PAGE. The following antibodies were used in this study: rabbit polyclonal HSPB7 (1:1000; #PA5-100825, Invitrogen); mouse monoclonal puromycin (1:5000; #MABE343, Millipore); rabbit polyclonal ASNS (1:1000; #14681-1-AP, Proteintech); rabbit polyclonal MTHFD2 (1:1000; #12270-1-AP, Proteintech); rabbit monoclonal ATF4 (1:1000; #11815, CST); rabbit monoclonal P-eIF2α (1:1000; #3597, CST); rabbit polyclonal eIF2α (1:1000; #9722, CST); rabbit monoclonal P-AMPKα (1:1000; #2535, CST); rabbit polyclonal AMPKα (1:1000; #2532, CST); rabbit polyclonal LC3B (1:1000; #NB100-2220, Novus Biologicals); rat monoclonal LAMP2 (1:100; #ABL-93, DSHB); rabbit polyclonal GAA (1:1000; #PA5-117464, Invitrogen); rabbit polyclonal SPG20 (1:1000; #13791-1-AP, Proteintech); rabbit monoclonal Beclin-1 (1:1000; #3495, CST); rabbit polyclonal STBD1 (1:1000; #11842-1-AP, Proteintech); mouse monoclonal ALIX (1:500; #MA1-83977, Invitrogen); rabbit monoclonal VCP (1:1000; #2649, CST); rabbit monoclonal ATP6V1A (1:2000; #ab199326, Abcam); rabbit polyclonal ATP6V0D1 (1:5000; #18274-1-AP, Proteintech); rabbit polyclonal P-S6 (1:1000; #2211, CST); mouse monoclonal S6 (1:1000; #2317, CST); rabbit monoclonal P-SQSTM1/p62 (1:1000; #39786, CST); and rabbit monoclonal SQSTM1/p62 (1:1000; #23214, CST).

### BN-PAGE

Isolated mitochondria (30 μg) were dissociated with digitonin (0.375%) and separated by using NativePAGE 3–12% Bis-Tris Gel (#BN1001BOX, Invitrogen), according to the manufacturer’s instructions.

### Mitochondrial isolation and bioenergetic analysis

Following isolation, mitochondrial concentration was determined by Protein Assay Dye Reagent Concentrate (#5000006, Bio-Rad). Oxygen consumption rates were measured using an Oxygraph System (DW1/AD Electrode Chamber, Hansatech Instruments Ltd) in 0.5 mL experimental buffer at 30 and 37 °C for yeast and mouse mitochondria, respectively. ADP and oligomycin was added for state 3 and state 4 measurement, respectively. The respiratory control ratio was determined by the ratio of state 3 to state 4 respiratory rate.

For yeast, cells were grown in 100 ml of YPD supplemented with tryptophan (20 μg/ml) overnight at 30 °C. The cells were then subcultured into 500 ml of YPG supplemented with tryptophan (20 μg/ml) and grown at 30 °C until OD_600_ = 2–3. Cells were collected by centrifugation and washed with water. The cell pellet was weighed, resuspended in 40 ml Buffer TD (100 mM Tris-SO_4_, pH 9.4, and 10 mM DTT), and shaken gently at 30 °C for 5 min. The cells were washed twice with 20 ml Buffer I (1.2 M sorbitol, 20 mM potassium phosphate, pH 7.4), resuspended in 20 ml Buffer I with 5 mg Zymolyase-20T (#120491-1, Amsbio)/1 g cells, and incubated at 37 °C with gentle shaking for about 60 min. Spheroplasts were harvested and washed twice with 40 ml ice-cold Buffer I. All subsequent operations were conducted at 4 °C. The pellet was resuspended in 30 ml Buffer II (0.6 M mannitol, 10 mM Tris-HCl, pH 7.4, 0.1% fatty acid-free BSA (#03117057001, Roche), 1 mM PMSF), and homogenized with 15 strokes using a Dounce tissue grinder. The supernatant was collected by two rounds of centrifugation at 2500×*g* for 5 min. The mitochondria-enriched pellet was collected by centrifugation at 12,000×*g* for 10 min. The mitochondrial pellet was washed with Buffer II, followed by two rounds of centrifugation at 2500×*g* for 5 min. The mitochondrial pellet was collected by centrifugation at 12,000×*g* for 10 min. Mitochondria was finally resuspended in 0.5 ml Buffer III (0.6 M mannitol, 20 mM Hepes-KOH, pH 7.4, 0.05% fatty acid-free BSA). About 200 μg of mitochondria was used to measure respiratory rates with 1 mM NADH and 25 μM ADP. State 4 respiration was measured in the presence of oligomycin (10 μg/ml). ATP synthesis rates were measured using a standard hexokinase/G6PD-coupled enzyme assay. Membrane potential was measured using the *Δψ*_m_-sensitive fluorescent dye safranin O as an indicator, with a dual wavelength Aminco DW-2 UV/VIS spectrophotometer.

For mouse studies, unanesthetized mice were decapitated using a guillotine. Mitochondria were isolated from hindlimb skeletal muscle, and respiration from 150 μg mitochondria was measured as previously described (Garcia-Cazarin et al, 2011). For complex I measurements, 5 mM glutamate and 2.5 mM malate were used as substrates. 200 μM ADP and 3 μg/ml oligomycin was added for state 3 and state 4 measurement, respectively. For complex II measurements, 5 μM rotenone was added before the substrate of 10 mM succinate. About 300 μM ADP and 3 μg/ml oligomycin was added for state 3 and state 4 measurement, respectively. The data represent three biological replicates with four technical replicates for each mouse.

### Reactive oxygen species production

Mitochondria were isolated from quadriceps muscles using a protocol adapted from Garcia-Cazarin et al. (Garcia-Cazarin et al, 2011). Briefly, unanesthetized mice were euthanized via guillotine, and both quadriceps muscles were removed and combined via mincing into PBS with 10 mM EDTA. Minced tissue was incubated with 0.01% trypsin and homogenized with a Teflon pestle in a Potter-type homogenizer on ice. The homogenate was centrifuged to remove cellular debris, and mitochondria were isolated via differential centrifugation to yield a purified mitochondrial pellet that was resuspended and stored on ice until use. The protein concentration of this mitochondrial suspension was measured via the Bradford protein assay, and 180 mg of mitochondria were centrifuged to remove the isolation buffer and resuspended in a buffer containing 125 mM KCl, 5 mM HEPES-KOH, 1 mM EGTA, and 0.3% w/v fatty acid-free BSA adjusted to a pH of 7.4 with KOH at 37 °C (KHEB buffer). 30% H_2_O_2_ was serially diluted in KHEB to yield 12 standards with concentrations ranging from 0 to 500 mM of H_2_O_2_. A reaction buffer was prepared containing 10 U/mL horseradish peroxidase, 100 mM Amplex UltraRed (#A36006, Invitrogen), 18 mM succinate, 20 mM glutamate, 10 mM malate, 2 mg/mL oligomycin, and 50 U/mL superoxide dismutase in KHEB buffer. Reaction buffer was added in triplicate for each mitochondrial sample to individual wells of a clear 96-well plate. Mixtures of reaction buffer and H_2_O_2_ standards or reaction buffer alone (to serve as blanks) were added to separate wells. The plate was incubated at 37 °C prior to the addition of prewarmed mitochondrial samples in a 1:1 ratio with reaction buffer. The development of Amplex UltraRed fluorescence in relative fluorescent units (RFU) was measured immediately with a plate reader (Molecular Devices, California, USA), periodically over an hour to generate plots of RFU/well/ unit of time. Technical replicates were averaged together, and the measured RFU from blanks were subtracted from samples and standards. A standard curve was generated by plotting RFU versus H_2_O_2_ concentration. A linear regression was fit to this standard curve to allow conversion of sample RFU into H_2_O_2_ concentration. The H_2_O_2_ generation rate of samples was calculated from the slope of a linear regression applied to the plot of sample H_2_O_2_ concentration versus time. The data represent three biological replicates with four technical replicates for each mouse.

### Animal experiments

*Ant1*^*Tg*^/+ mice were generated as previously described (Wang et al, 2022), in which the *Ant1* transgene is expressed from its native promoter. All the mice used in the study were on a C57Bl/6 N background. The animal experiments have been approved by the Institutional Animal Care and Use Committee (IACUC # D16-00318) of the State University of New York Upstate Medical University. The experiments were performed using age-matched and, when possible, littermate controls. For most experiments, the animals were fed with a regular chow diet ad libitum and were housed at an ambient temperature. When investigating mTOR signaling, mice were starved for 24 h and then refed for 1.5 h prior to tissue collection. Food and water were placed at a low and reachable position for the *Ant1*^*Tg*^/+ mice over 1-year-old.

### Dual-energy X-ray absorptiometry (DXA)

Body composition and tibial length was assessed by dual-energy X-ray absorptiometry (DXA) imaging, using an UltraFocus-DXA instrument (Hologic-Faxitron Bioptics, LLC, Marlborough, MA). Prior to imaging, the instrument was calibrated using an acrylic and aluminum step-phantom of known tissue equivalent densities. Animals were briefly anesthetized by intraperitoneal injection of Ketamine-Xylazine cocktail (75 mg/kg) to facilitate prone positioning with limbs extended on the scanning bed. A series of digital radiographs were obtained at low (40kVP/0.28 mA, 3.53 s; 40kVp/0.97 mA, 0.57 s) and high (80kVp/0.14 mA, 3.23 s; 80kVp 0.44 mA, 0.94 s) beam energies. Automated image processing and data integration was performed using VisionDXA software (Hologic-Faxitron Bioptics). Measurements of bone mineral density, fat and lean tissue composition were obtained for the whole body excluding the head, and regions of interest were drawn over the right femur and L4 vertebrae. Tibia length was measured from the center of the right tibial plateau to the apex of the tibial plafond. All lean mass measurements were normalized to tibia length.

### Surface sensing of translation (SUnSET) assay

In vivo assessment of protein synthesis was performed using the antibiotic puromycin, a structural analog of tyrosyl-tRNA. When used at low concentrations, puromycin incorporates at the C-terminus of nascent polypeptides, and the accumulation of puromycin-conjugated peptides indicates the rate of protein synthesis (Goodman et al, 2011). Mice were given an intraperitoneal injection of 0.04 mmol/g puromycin dissolved in 100 μl of PBS. Thirty minutes following injection, mice were sacrificed via decapitation and quadriceps muscles were extracted and frozen in liquid N_2_. About 30–50 mg of snap-frozen quadricep muscle tissues were minced and homogenized in 0.3 mL of ice-cold lysis buffer (40 mM Tris-HCl, pH 7.4; 5 mM EGTA; 1 mM EDTA, pH 7.4; 0.5% Triton X-100, 100 mM β-glycerophosphate; 1 mM Na_3_VO_4_; 25 mM NaF; 1 mM PMSF; and 10 μg/mL Leupeptin). Cellular debris was removed by centrifugation at 16,000×*g* for 5 min at 4 °C. About 30–50 mg of quadriceps muscle lysates were prepared with Laemmli buffer, heated at 95 °C for 5 min, and used for SDS-page and Western blot analysis. Puromycin-conjugated nascent peptides were detected via immunoblotting using a mouse monoclonal anti-puromycin antibody (1:5000; #MABE343, Millipore).

### V_1_/V_o_ ratio

Muscle samples were fractionated to obtain the lysosome-enriched fractions using differential centrifugation, as follows. Quadriceps muscle was rapidly dissected and placed in 1 mL/100 mg tissue of cytosol lysis buffer (20 mM Tris-HCl, 1 mM EDTA, 1 mM EGTA, 1% glycerol, and 2 mM dithiothreitol, pH 7.8). Muscle was minced and then homogenized in a 2 mL Dounce homogenizer with eight strokes using a bench-top drill press set to 570 rotations per minute. Homogenates were then centrifuged for 5 min at 2000×*g* twice. Pellets were discarded after each spin. To obtain the lysosome-enriched fraction, the supernatant was centrifuged for 30 min at 13,000×*g*. The lysosomal pellet was resuspended in 200 ml of acid lysis buffer (200 mM Na-acetate, 50 mM NaCl, and 0.1% Triton X-100, pH 5.0). These samples were then used for western blot analysis (10 ug).

### Measurement of autophagic flux

To assess changes in autophagic flux, we administered intraperitoneal colchicine, a well-established autophagosome degradation inhibitor (Ju et al, 2010), at 0.4 mg/kg/day for 2 days. Mice were sacrificed via guillotine decapitation exactly 48 h after the initial injection, followed by rapid dissection and snap freezing of muscle tissue in liquid nitrogen. Muscle lysates were then prepared, and immunoblotting for LC3B was performed as described above.

### Enzymatic activity assay

To determine total cathepsin enzyme activity in subcellular fractions of mouse quadriceps muscles, we followed the procedure described by Gumpper et al. (Gumpper et al, 2019) with the following modifications: Muscle samples were fractionated to obtain cytosolic and lysosome-enriched fractions using differential centrifugation, as follows. Quadriceps muscle was rapidly dissected and placed in 1 mL/100 mg tissue of cytosol lysis buffer (20 mM Tris-HCl, 1 mM EDTA, 1 mM EGTA, 1% glycerol, and 2 mM dithiothreitol, pH 7.8). Muscle was minced and then homogenized in a 2 mL Dounce homogenizer with 8 strokes using a bench-top drill press set to 570 rotations per minute. Homogenates were then centrifuged for 5 min at 2000×*g* twice. Pellets were discarded after each spin. To obtain the lysosome-enriched fraction, the supernatant was centrifuged for 30 min at 13,000×*g*. The supernatant, containing the cytosolic fraction, was used for the omnicathepsin enzymatic activity assay. The lysosomal pellet was resuspended in 200 ml of acid lysis buffer (200 mM Na-acetate, 50 mM NaCl, and 0.1% Triton X-100, pH 5.0), and the muscle acid lysate was obtained through sonication on ice for three 5 s to 10 s “on-off” cycles. Following sonication, the muscle acid lysates were centrifuged for 30 min at 13,000×*g*. The supernatant, containing the lysosomal contents, were used for the omnicathepsin enzymatic activity assay.

To determine total cathepsin activity, 5 mg of cytosolic and lysosome-enriched fractions were used. The plate was incubated at 37 °C prior to the addition of prewarmed lysosomal and cytosolic samples in a 1:1 ratio with reaction buffer. The development of fluorescence in relative fluorescent units (RFU) was measured immediately with a plate reader (Molecular Devices, California, USA), periodically over an hour to generate *V*_max_. Technical replicates were averaged together, and the measured *V*_max_ from blanks were subtracted from the samples.

Proteasomal activity assay was performed as previously described by Wang and co-workers (Wang et al, 2022).

### Protein/DNA ratio

DNA and proteins were extracted from approximately 15 mg of quadriceps muscles using AllPrep DNA/RNA/Protein Mini kit (#80004, Qiagen). DNA concentrations were determined by the Thermo Scientific NanoDrop 2000c spectrophotometer. Protein concentrations were determined by Bradford protein assay. Relative protein contents in the muscles were calculated after normalizing by the total amount of DNA.

### Muscle histology sample preparation

Quadriceps muscle was rapidly dissected and frozen using 2-methylbutane pre-chilled with liquid nitrogen (Meng et al, 2014). Fresh frozen muscle was then sectioned at a thickness of 10 µm. Slides were thawed and dried prior to histological analysis.

### Immunofluorescence

Muscle sections were permeabilized and fixed using ice-cold 100% methanol at −20 °C for 15 min, washed in PBS for 5 min, and treated with blocking buffer (1X PBS, 5% Normal Goat Serum, and 0.3% Triton X-100) at room temperature for 1 h. Samples were then incubated with primary antibodies at 4 °C overnight. Sections were then washed three times with 1X PBS/0.05% Tween 20 prior to probing with Alexa Fluor-conjugated secondary antibodies for two hours at room temperature, followed by three more washes with 1X PBS/0.05% Tween 20, and mounting with ProLong Gold Antifade Mountant (#P36934, Invitrogen).

### Myofiber size quantification

Prepared samples were probed with anti-dystrophin (1:200; #PA5-32388, Invitrogen), followed by Alexa Fluor 633-conjugated anti-rabbit IgG (H + L) (1:500, #A-21071, Invitrogen). Three biological replicates and three sections from each mouse were scanned using the Leica SP8 confocal microscope. Myofiber minimum ferret diameter and cross-sectional area were quantified using MyoVision v2.0 (Viggars et al, 2022).

### Hematoxylin and eosin staining

Standard procedures were followed (#HHS32 and #HT110232, Sigma-Aldrich).

### Transmission electron microscopy

Approximately 1 mm^3^ fresh quadriceps muscle was fixed in 4% glutaraldehyde/0.1 M cacodylate buffer, pH 7.2, at room temperature for 2 h and postfixed with 1% osmium tetroxide/0.1 M cacodylate buffer, pH 7.2, at room temperature for one hour. The specimens were dehydrated in 50, 70, 90, and 100% ethanol and propylene oxide, before being embedded in Luft’s Araldite 502 embedding medium and cut into ultrathin (70–80 nm) sections. The ultrathin sections were then stained with ethanolic uranyl acetate and Reynold’s lead citrate. The samples were examined with a JOEL JEM1400 transmission electron microscope, and images were acquired with a Gatan DAT-832 Orius camera.

### Periodic acid-Schiff staining

Muscle sections were oxidized in 0.5% Periodic Acid for 5 min, rinsed three times in distilled water, and placed in Schiff’s reagent (#26853, Electron Microscopy Sciences) for 15 min. Specimens were then washed in warm tap water for 5 min and briefly rinsed in distilled water, followed by dehydration and mounting with Micromount (#3801730, Leica Biosystems). Myofiber glycogen accumulation was imaged using the Nikon Eclipse Ci-L microscope.

### Lipofuscin staining

Specimens were stained as previously described (Evangelou and Gorgoulis, 2017). Briefly, sections were dehydrated gradually until 70% ethanol, followed by treatment with filtered Sudan Black B (#199664, Sigma-Aldrich) for 4 min. Specimens were then rinsed three times with 50% ethanol and washed with distilled water. Finally, samples were mounted in 40% glycerol/1XTBS and myofiber lipofuscin accumulation was imaged using the Nikon Eclipse Ci-L microscope.

### Assessment of lysosomal damage

Prepared samples were probed with anti-LAMP2 (1:15; #GL2A7, DSHB) and anti-Galectin-3 antibodies (1:200, #32790, Santa Cruz Biotechnology) at 4 °C overnight. Sections were then washed three times with 1X PBS/0.05% Tween 20 prior to probing with Alexa Fluor 488-conjugated anti-rat IgG (H + L) (1:500, #4416, CST) and Alexa Fluor 546-conjugated anti-mouse IgG1 (1:250, #A-21123, Invitrogen) for two hours at room temperature, followed by three more washes with 1X PBS/0.05% Tween 20, and mounting with ProLong Gold Antifade Mountant (#P36934, Invitrogen).

### RNA extraction, sequencing, and analysis

RNA quantity and quality were validated using the Agilent 2100 Bioanalyzer. Sequencing libraries were generated using the Illumina TruSeq Stranded mRNA Library Prep kit (#20020594, Illumina) with either 1 μg (yeast) or 500 ng (mouse) of total RNA as input. Sequencing libraries were validated using the Agilent 2100 Bioanalyzer. Libraries were sequenced on the NextSeq 2000 instrument, with paired-end 2 × 100 bp reads. Sequencing depth was 50 million paired-end reads per sample. Raw FASTQ files were imported into Partek Flow, the adapters were trimmed, and either the Bowtie 2 (yeast) or STAR (mouse) aligner was used for alignment. Differential expression analysis was performed using a one-way analysis of variance (ANOVA).

For yeast, cells were inoculated in 2.5 ml of YPD supplemented with tryptophan (200 μg/ml) and grown overnight at 30 °C. The overnight culture was subcultured in 20 ml of YPGR supplemented with tryptophan (200 mg/ml) and grown at 30 °C until mid-log phase. The cells were washed in ice-cold water and pelleted. The cell pellet was resuspended in 300 ml of RNA buffer (0.5 M NaCl, 200 mM Tris-HCl, pH 7.5, 10 mM EDTA). Approximately 200 μl of chilled acid-washed glass beads were added to the cell resuspension. About 300 μl of phenol/chloroform/isoamyl alcohol (25:24:1, #AC327115000, Thermo Fisher Scientific) was added and mixed well with the cells and glass beads. The yeast cell wall was broken by vortex at the highest speed for 2 min. The cell debris and glass beads were pelleted by centrifugation at top speed for 5 min at room temperature. The aqueous top layer was collected, followed by the addition of an equal volume of phenol/chloroform/isoamyl alcohol (25:24:1). The mixture was then vortexed vigorously for 10 s and centrifuged at top speed for 5 min at room temperature. The aqueous top layer was collected, followed by the addition of two volumes of ice-cold 100% ethanol. The mixture was incubated at −20 °C for at least 30 min. The RNA was pelleted by centrifugation at top speed for 10 min at 4 °C, washed with ice-cold 70% ethanol and dried.

For mouse studies, total RNA was extracted from snap-frozen 2-month-old quadricep muscles using the miRNeasy Kit (#217084, Qiagen). ANOVA identified a total of 7910 differentially expressed genes. For proper comparison of wildtype and *Ant1*^*Tg*^/+ muscle, filters for significance and fold-change were applied: false discovery rate (FDR) <0.05 and fold-change >1.5 for upregulated genes. This yielded a total of 272 genes, which were used for gene set enrichment analysis (GSEA)(Subramanian et al, 2005). The top 15 enriched canonical pathways were displayed in graphical form. Differential expression analysis was also performed on aged quadriceps muscle from both wild-type and *Ant1*^*Tg*^/+ mice, as described above. Genes were filtered for significance (FDR <0.05) and then compared to the significantly differentially expressed genes at 2 months.

### Quantitative mass spectrometry subcellular fractionation

Mice were sacrificed via decapitation, and one quadriceps muscle was rapidly dissected and minced in 1.0 mL/100 mg tissue of ice-cold PBS. Tissues were then centrifuged at 800×*g* for 5 min. The pellet was resuspended in 1 mL/100 mg tissue of STM buffer (250 mM sucrose, 50 mM HEPES-KOH, pH 7.4, 5 mM MgCl_2_, and protease inhibitors). Samples were then homogenized in a 2 mL Dounce homogenizer (0.15–0.25 mm clearance) with eight strokes using a bench-top drill press set to 570 rotations per minute. Homogenates were centrifuged at 800×*g* for 15 min. To remove contaminating mitochondria, the supernatant was centrifuged at 11,000×*g* for 10 min. To further purify the cytosolic fraction, the supernatant was centrifuged twice at 21,000×*g* for 30 min and frozen for further analysis.

### Quantitative mass spectrometry sample processing and data analysis

Three biological replicates of 2-month-old wildtype and *Ant1*^*Tg*^/+ quadriceps were prepared for multiplexed quantitative mass spectrometry. Samples were buffer exchanged on a 3 kDa molecular weight cutoff filter (Amicon 3k Ultracel) using 50 mM triethylammonium bicarbonate, pH 8.0 (Thermo). About 50 µg was taken for digestion using an EasyPep Mini MS sample prep kit (Thermo, A40006). To each buffer-exchanged sample, 65 µL of lysis buffer was added, followed by 50 µL of reduction solution and 50 µL of alkylating solution. Samples were incubated at 95 °C for 10 min, then cooled to room temperature. To each sample, 4 µg of trypsin/Lys-C protease was added, and the reaction was incubated at 37 °C overnight. TMT reagents were reconstituted with 20 µL acetonitrile (ACN) and the contents of each label added to a digested sample. After 60 min, 50 µL of quenching solution was added, consisting of 20% formic acid and 5% hydroxylamine (v/v) in water. The labeled digests were cleaned up by a solid-phase extraction device contained in the EasyPep kit, and dried by speed-vac. The individually labeled samples were dissolved in 50 µL of 3% ACN and 0.2% trifluoroacetic acid (v/v) in water, and 20 µL of each was used to create a pooled sample consisting of 160 µg. To ensure digestion and labeling quality of the pooled samples, 100 µg of the pooled sample was fractionated using a High pH Reversed-Phase Peptide Fractionation Kit (#84868, Pierce), per the manufacturer’s instructions for TMT-labeled peptides. In brief, samples were dissolved in 300 µL of 0.1% trifluoroacetic acid in water, and applied to the conditioned resin. Samples were washed first with water and then with 300 µL of 5% ACN, 0.1% triethylamine (TEA) in water. The second wash was collected for analysis. Peptides were step eluted from the resin using 300 µL of solvent consisting of 5 to 50% ACN with 0.1% TEA in eight steps. All collected fractions were dried in a speed-vacuum. For LC-MS/MS, dried fractions were reconstituted in 25 µL of load solvent consisting of 3% ACN and 0.5% formic acid in water, and a 5 µL aliquot was diluted 1:3 with the same solvent. Of these 15 µL, 2 µL were injected onto a pulled tip nano-LC column (New Objective, FS360-75-10-N) with 75 µm inner diameter packed to 28 cm with 2.4 µm, 120 Å, C18AQ particles (Dr. Maisch). The column was maintained at 50 °C with a column oven (Sonation GmbH, PRSO-V2). The peptides were separated using a 135-minute gradient consisting of 3–12.5% ACN over 60 min, 12.5–28% over 60 min, 28–85% ACN over 7 min, a 3 min hold, and 5 min re-equilibration at 3% ACN. The column was connected in line with an Orbitrap Lumos (Thermo) via a nanoelectrospray source operating at 2.5 kV. The mass spectrometer was operated in data-dependent top speed mode with a cycle time of 3 s. MS1 scans were collected from 375 to 1500 m/z at 120,000 resolution and a maximum injection time of 50 ms. HCD fragmentation at 37% collision energy was used, followed by MS2 scans in the Orbitrap at 50,000 resolution with a 125 ms maximum injection time.

The MS data were searched using SequestHT in Proteome Discoverer (version 2.4, Thermo Scientific) against the *M. Musculus* proteome from Uniprot, containing 17,097 sequences and a list of common laboratory contaminant proteins. Enzyme specificity for fully tryptic with up to two missed cleavages. Precursor and product ion mass tolerances were 10 ppm and 0.02 Da, respectively. Cysteine carbamidomethylation, TMT 16-plex at any N-terminus and TMT 16-plex at lysine were set as a fixed modification. Methionine oxidation was set as a variable modification. The output was filtered using the Percolator algorithm with a strict FDR set to 0.01. Quantification parameters included the allowance of unique and razor peptides, reporter abundance based on intensity, lot-specific isotopic purity correction factors, normalization based on total peptide amount, protein ratio based on protein abundance, and background-based hypothesis testing (ANOVA).

### Liquid chromatography-mass spectrometry (LC-MS) metabolomics subcellular fractionation

Mice were sacrificed via decapitation, and one quadriceps muscle was rapidly dissected and minced in 0.5 mL/100 mg tissue of KClEM buffer (180 mM KCl, 1 mM EDTA, pH 7.4, 5 mM MOPS-KOH (pH 7.2), and protease inhibitors). Tissues were then homogenized in a 2 mL Dounce homogenizer (0.15–0.25 mm clearance) with eight strokes using a bench-top drill press set to 570 rotations per minute. Homogenates were centrifuged at 1000×*g* for 4 min and the supernatant was centrifuged at 1900×*g* for 4 min. To obtain the mitochondrial fraction, the supernatant was centrifuged at 11,000×*g* for 15 min. Mitochondrial fractions were then washed with KClEM buffer, resuspended in 80% methanol and frozen for future analysis. Meanwhile, the supernatant was centrifuged at 4000×*g* using a 100 kDa filter, to obtain the cytosolic fraction. Cytosolic fractions were stored in 80% methanol and frozen for future analysis.

### LC-MS metabolomic sample processing and data analysis

All solvents were HPLC-MS grade from Sigma-Aldrich. The extracted samples were dried under nitrogen flow and resuspended in 20 µL of 30% acetonitrile in water containing 10 µM fully labeled amino acids (Cambridge Isotopes lab, Cambridge, MA, USA). Five microliters of each cytosolic sample were pooled together. A 12-point standard curve was prepared in the same solution, with the highest concentration being 100 µM for all targets. The subsequent standards were prepared as a 1:3 dilution series. Samples were analyzed by LC-MS on a Vanquish LC coupled to an ID-X MS (Thermo Fisher Scientific). Five µL of the sample was injected on a ZIC-pHILIC peek-coated column (150 mm × 2.1 mm, 5 micron particles, maintained at 40 °C, Sigma-Aldrich). Buffer A was 20 mM ammonium carbonate, 0.1% ammonium hydroxide in water, and Buffer B was acetonitrile 97% in water. The LC program was as follows: starting at 93% B, to 40% B in 19 min, then to 0% B in 9 min, maintained at 0% B for 5 min, then back to 93% B in 3 min and re-equilibrated at 93% B for 9 min. The flow rate was maintained at 0.15 mL min^−1^, except for the first 30 s where the flow rate was uniformly ramped up from 0.05 to 0.15 mL min^−1^. Data were acquired on the ID-X in switching polarities at 120,000 resolution, with an AGC target of 1e5, and a m/z range of 65 to 1000. Data were acquired in switching polarities for all samples in MS1 mode. MS2 and MS3 data were acquired on the pooled samples using the AquirX DeepScan function, with four reinjections, separately in positive and negative ion mode.

For targeted quantification, the data were analyzed using Tracefinder Software (Thermo Fisher Scientific). The standard curve was used to calculate the concentration of all the targets, using the labeled amino acid with the closest retention time to each target as an internal standard. The targeted metabolomic data were normalized using median-centered normalization derived from an untargeted metabolomic screen that was analyzed in Compound Discoverer 3.3 (CD, Thermo Fisher Scientific).

### Statistical analysis

Quantitative results are given as mean ± SEM. *P* values were calculated by a two-tailed Student’s *t*- test. The “*n*” in these experiments represents the number of biological replicates used. In Fig. 8, significant changes in protein abundances were determined using background-based hypothesis testing (ANOVA) with a cutoff of *p* < 0.05. For RNA-seq analysis, one-way analysis of variance (ANOVA) was used to determine significantly differentially expressed genes. A *q* value cutoff of 0.05 was used to identify genes that are differentially expressed in transgenic and wild-type muscles. For metabolite analysis (Fig. 6C), two-way ANOVA was used to determine significant changes in metabolite concentration of wildtype and *Ant1*^*Tg*^/+ cytosolic muscle fractions with a cutoff of *p* < 0.05.

## Supplementary information

Appendix

Table EV1

Peer Review File

Dataset EV1

Dataset EV2

Dataset EV3

Dataset EV4

Dataset EV5

Source data Fig. 1

Source data Fig. 2

Source data Fig. 3

Source data Fig. 4

Source data Fig. 5

Source data Fig. 6

Source data Fig. 7

Source data Fig. 8

Expanded View Figures

## Data Availability

The raw and processed high-throughput sequencing datasets, including RNA-seq and proteomic data generated in this study, have been deposited to the Gene Expression Omnibus (GEO) database (accession number GSE282262; URL: https://www.ncbi.nlm.nih.gov/geo/query/acc.cgi?acc=GSE282262) and the Proteomics Identifications database (PRIDE) (accession number PXD058344), respectively. Source data have been deposited to the BioStudies database (URL: https://www.ebi.ac.uk/biostudies/studies/S-BSST2669?key=2b320a6e-da8b-4d55-8a8c-30a0609174ef; accession number: S-BSST2669). The source data of this paper are collected in the following database record: biostudies:S-SCDT-10_1038-S44319-026-00774-9.
