# Supplementary material for: Mitochondrial protein import stress causes lysosomal damage and progressive tissue atrophy
Source: EMBO Rep. 2026 Apr 27;27(11):2973–3000. doi: 10.1038/s44319-026-00774-9 (PMC13260833; doi:10.1038/s44319-026-00774-9)
Supplement: Supplementary file 1 — Appendix [file 44319_2026_774_MOESM1_ESM.pdf]

Appendix Figures

Mitochondrial Protein Import Stress Causes Lysosomal Damage and Progressive Tissue Atrophy

Nicholas A. Brennan, Xiaowen Wang, Arnav Rana, Sanaea Z. Bhagwagar, Jason Horton, Patricia M. Kane & Xin Jie Chen

Corresponding author: Chenx@upstate.edu

Table of content

Appendix Figure S1. Overexpression of *AAC2* increases sensitivity to hydroxyurea and promotes the formation of Ade4-GFP foci.

Page 2

Appendix Figure S2. Upregulated pathways identified by RNA-seq analysis of *Ant1*^{Tg}/+ quadricep muscle at 2 months of age.

Page 3

Appendix Figure S3. Immunoblotting showing the quality of cytosolic and lysosome-enriched subcellular fractionations from quadricep muscle.

Page 4

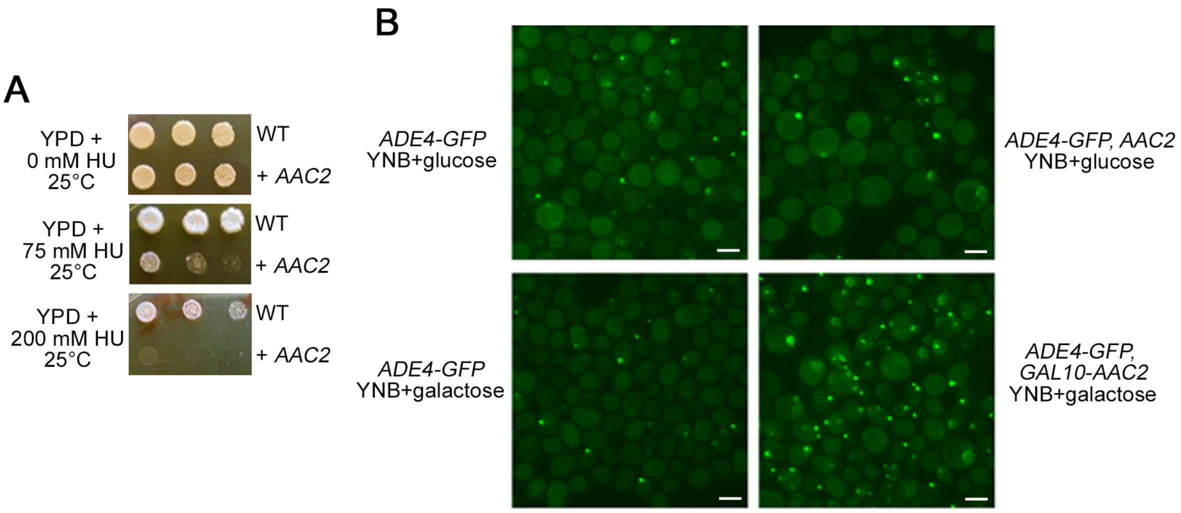

Appendix Figure S1. Overexpression of *AAC2* increases sensitivity to hydroxyurea and promotes the formation of Ade4-GFP foci. (**A**) Sensitivity of yeast cells expressing an extra copy of *AAC2* integrated into the chromosome (+ *AAC2*) to hydroxyurea (HU). (**B**) Overexpression of *AAC2* from the strong *GAL10* (*GAL10-AAC2*) but not its native (*AAC2*) promoter stimulates the formation of Ade4 "purinosome", which suggests nucleotide depletion. *ADE4-GFP*, *GAL10-AAC2* and *AAC2* are all chromosomally integrated. Scale bar, 5 μm.

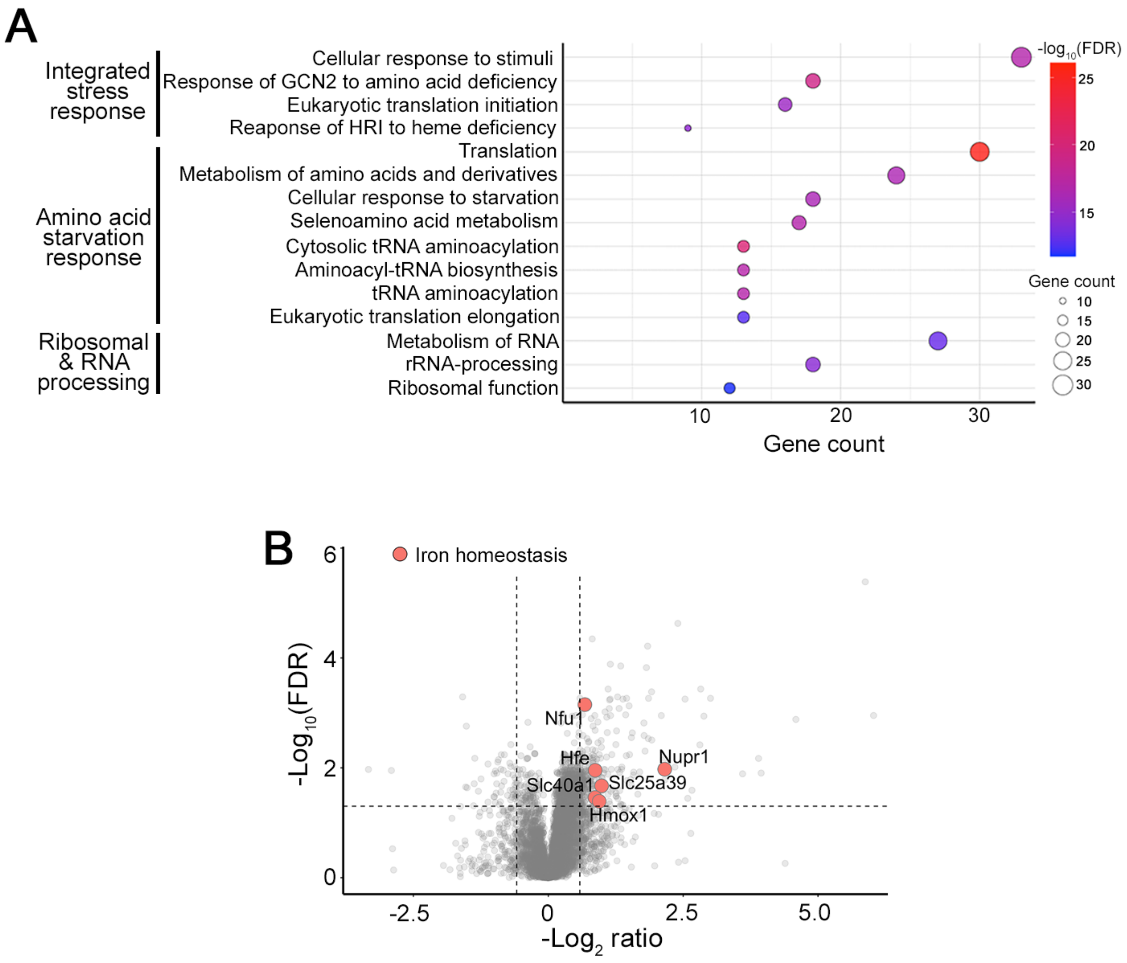

Appendix Figure S2. Upregulated pathways identified by RNA-seq analysis of *Ant1^{Tg}*/+ quadricep muscle at 2 months of age. (**A**) Dot plot showing the fifteen most upregulated pathways in *Ant1^{Tg}*/+ muscle. (**B**) Volcano plot showing the upregulation of genes involved in iron homeostasis in *Ant1^{Tg}*/+ muscle (n=4).

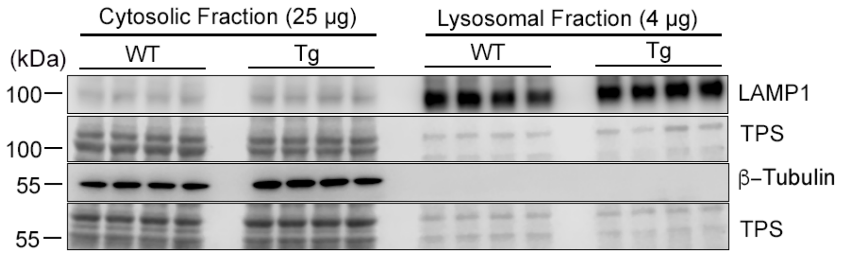

Appendix Figure S3. Immunoblotting showing the quality of cytosolic and lysosome-enriched subcellular fractionations from quadricep muscle. LAMP1 and β-tubulin were used as markers for the lysosomal and cytosolic subcellular fractions, respectively (n=4/genotype; female). Note that only 4 µg of proteins are loaded for the lysosomal fraction relative to 25 µg for the cytosolic fraction. TPS, total protein staining.
