# Supplementary material for: Mitochondrial protein import stress causes lysosomal damage and progressive tissue atrophy
Source: EMBO Rep. 2026 Apr 27;27(11):2973–3000. doi: 10.1038/s44319-026-00774-9 (PMC13260833; doi:10.1038/s44319-026-00774-9)
Supplement: Supplementary file 2 — Table EV1 [file 44319_2026_774_MOESM2_ESM.pdf]

Yeast strains used in this study

Strain name	Genotype	SOURCE
BY4741	*MAT***a**, *his3Δ1, leu2Δ0, met15Δ0, ura3Δ0*	Horizon
BY4742	*MAT*α, *his3Δ1, leu2Δ0, lys2Δ0, ura3Δ0*	Horizon
BY4742/AN1	as BY4742, *trp1Δ::AAC2-HIS3*	This lab
BY4742/AG3	as BY4742, *trp1Δ::GAL10-AAC2-HIS3*	This lab
BY4741/trp1	as BY4741, but *trp11Δ::kan*	Horizon
BY4741/vma1	as BY4741, but *vma1Δ::kan*	Horizon
BY4741/vma11	as BY4741, but *vma11Δ::kan*	Horizon
BY4741/vma12	as BY4741, but *vma12Δ::kan*	Horizon
BY4741/vma13	as BY4741, but *vma13Δ::kan*	Horizon
BY4741/tim18	as BY4741, but *tim18Δ::kan*	Horizon
BY4741/tom5	as BY4741, but *tom5Δ::kan*	Horizon
BY4741/tom6	as BY4741, but *tom6Δ::kan*	Horizon
BY4741/tom7	as BY4741, but *tom7Δ::kan*	Horizon
BY4741/tom70	as BY4741, but *tom70Δ::kan*	This study
CY3322	as BY4742, *trp1Δ::GAL10-aac2*^{A128P}*-HIS3*	This lab
CY5912	BY background, *vma13Δ::kan, trp1Δ::AAC2-HIS3*	This study
CY5919	BY background, *vma1Δ::kan, trp1Δ::GAL10-AAC2-HIS3*	This study
CY5922	BY background, *vma11Δ::kan, trp1Δ::GAL10-AAC2-HIS3*	This study
CY5924	BY background, *vma12Δ::kan, trp1Δ::GAL10-AAC2-HIS3*	This study
CY5926	BY background, *vma13Δ::kan, trp1Δ::GAL10-AAC2-HIS3*	This study
CY6126	BY background, *tim18Δ::kan, vma13Δ::kan*	This study
CY6606	BY background, *vma1Δ::kan, trp1Δ::AAC2-HIS3*	This study
CY6716	BY background, *vma13Δ::kan, trp1Δ::kan*	This study
CY6795	BY background, *VMA5-GFP, trp1Δ::GAL10-AAC2-HIS3*	This study
CY6946	BY background, *vma11Δ::kan, trp1Δ::AAC2-HIS3*	This study
CY6948	BY background, *vma12Δ::kan, trp1Δ::AAC2-HIS3*	This study
CY7108	BY background, *tom5Δ::kan, vma13Δ::kan*	This study
CY7112	BY background, *tom6Δ::kan, vma13Δ::kan*	This study
CY7147	BY background, *tim18Δ::kan, VMA5-mNG*	This study
CY7158	BY background, *tom7Δ::kan, vma13Δ::kan*	This study
CY7163	BY background, *tom70Δ::kan, vma13Δ::kan*	This study
CY7175	BY background, *tom7Δ::kan, VMA5-mNG*	This study
CY7181	BY background, *tom6Δ::kan, VMA5-mNG*	This study
CY7183	BY background, *tom70Δ::kan, VMA5-mNG*	This study
CY7187	BY background, *tom5Δ::kan, VMA5-mNG*	This study
YMR300C	as BY4741, *ADE4-GFP::His37158*	Invitrogen
