# Supplementary figures and images for: Mitochondrial protein import stress causes lysosomal damage and progressive tissue atrophy
Source: EMBO Rep. 2026 Apr 27;27(11):2973–3000. doi: 10.1038/s44319-026-00774-9 (PMC13260833; doi:10.1038/s44319-026-00774-9)

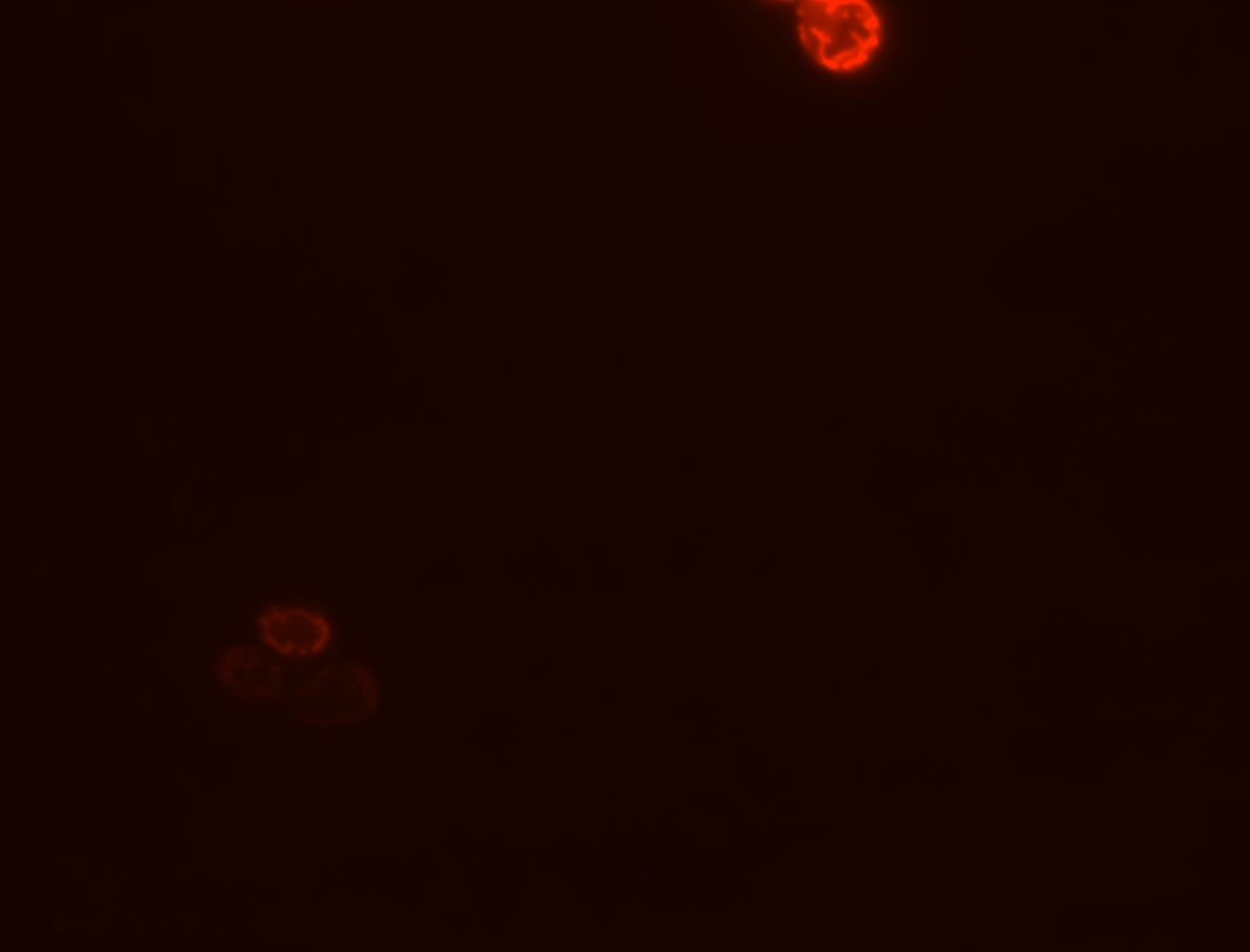

Supplement: Supplementary file 9 — Source data Fig. 1 [file 44319_2026_774_MOESM9_ESM.zip › Figure 1/1G/WT_mCherry.TIF]

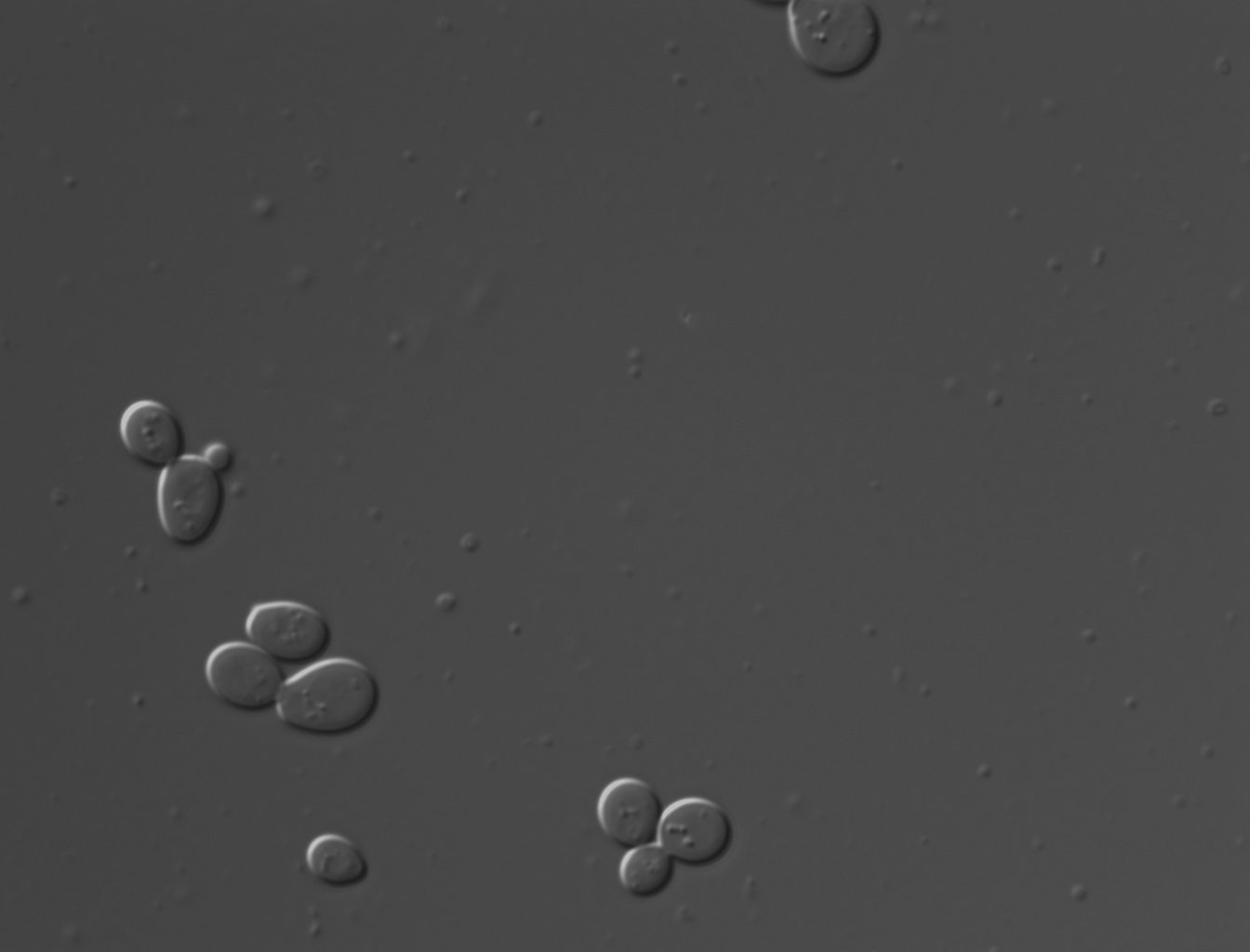

Supplement: Supplementary file 9 — Source data Fig. 1 [file 44319_2026_774_MOESM9_ESM.zip › Figure 1/1G/WT_DIC.TIF]

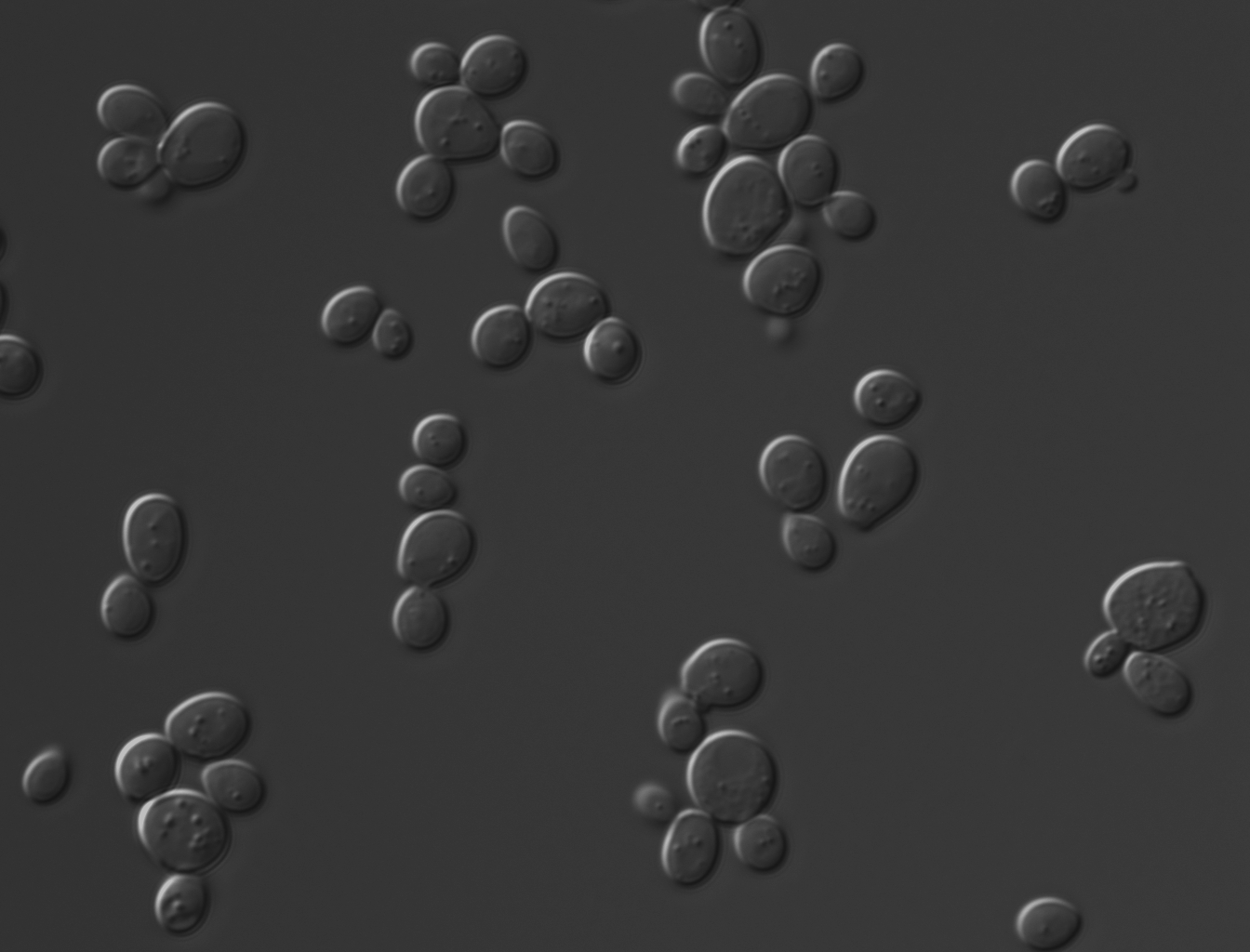

Supplement: Supplementary file 9 — Source data Fig. 1 [file 44319_2026_774_MOESM9_ESM.zip › Figure 1/1G/GAL10-AAC2_DIC.TIF]

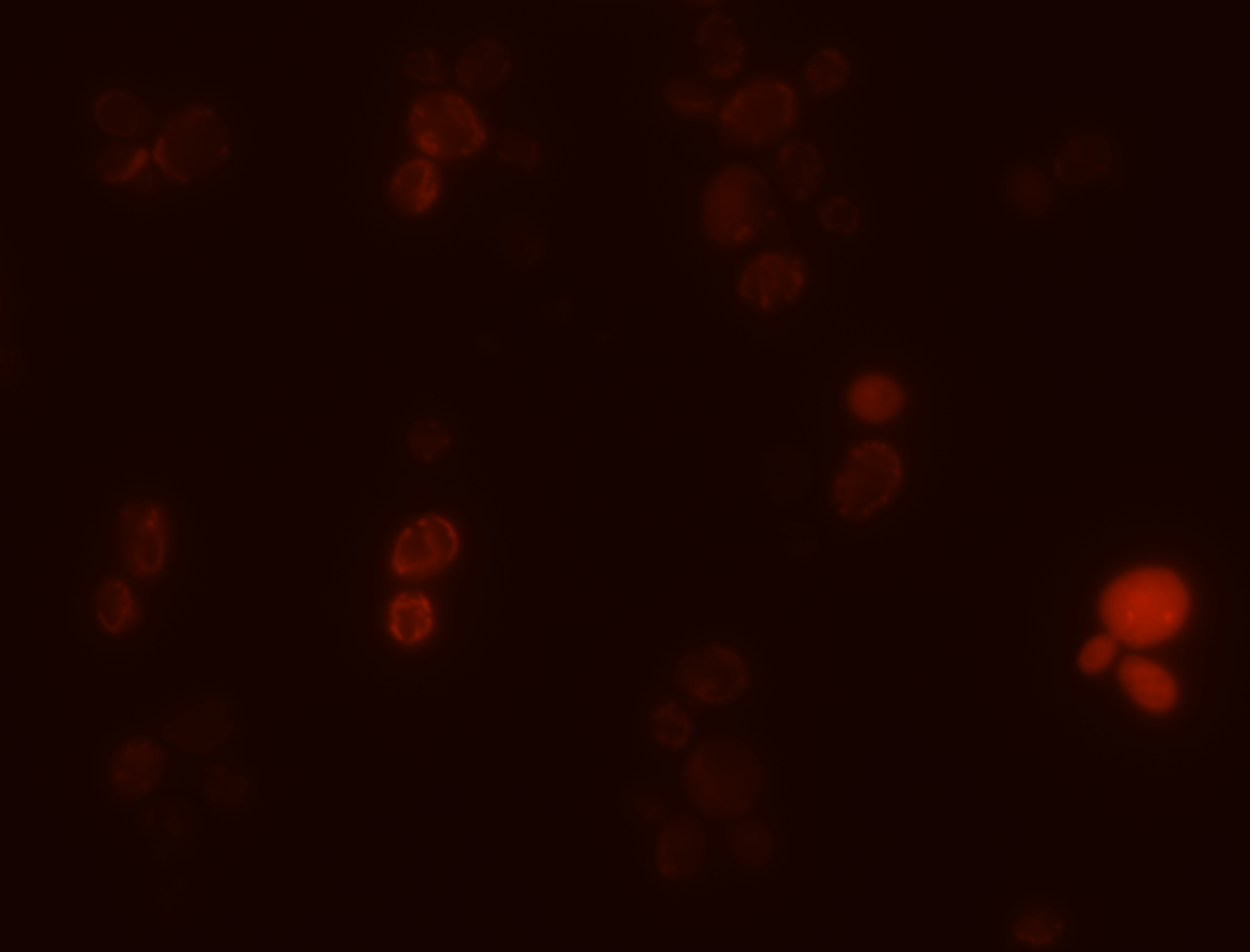

Supplement: Supplementary file 9 — Source data Fig. 1 [file 44319_2026_774_MOESM9_ESM.zip › Figure 1/1G/GAL10-AAC2_mCherry.TIF]

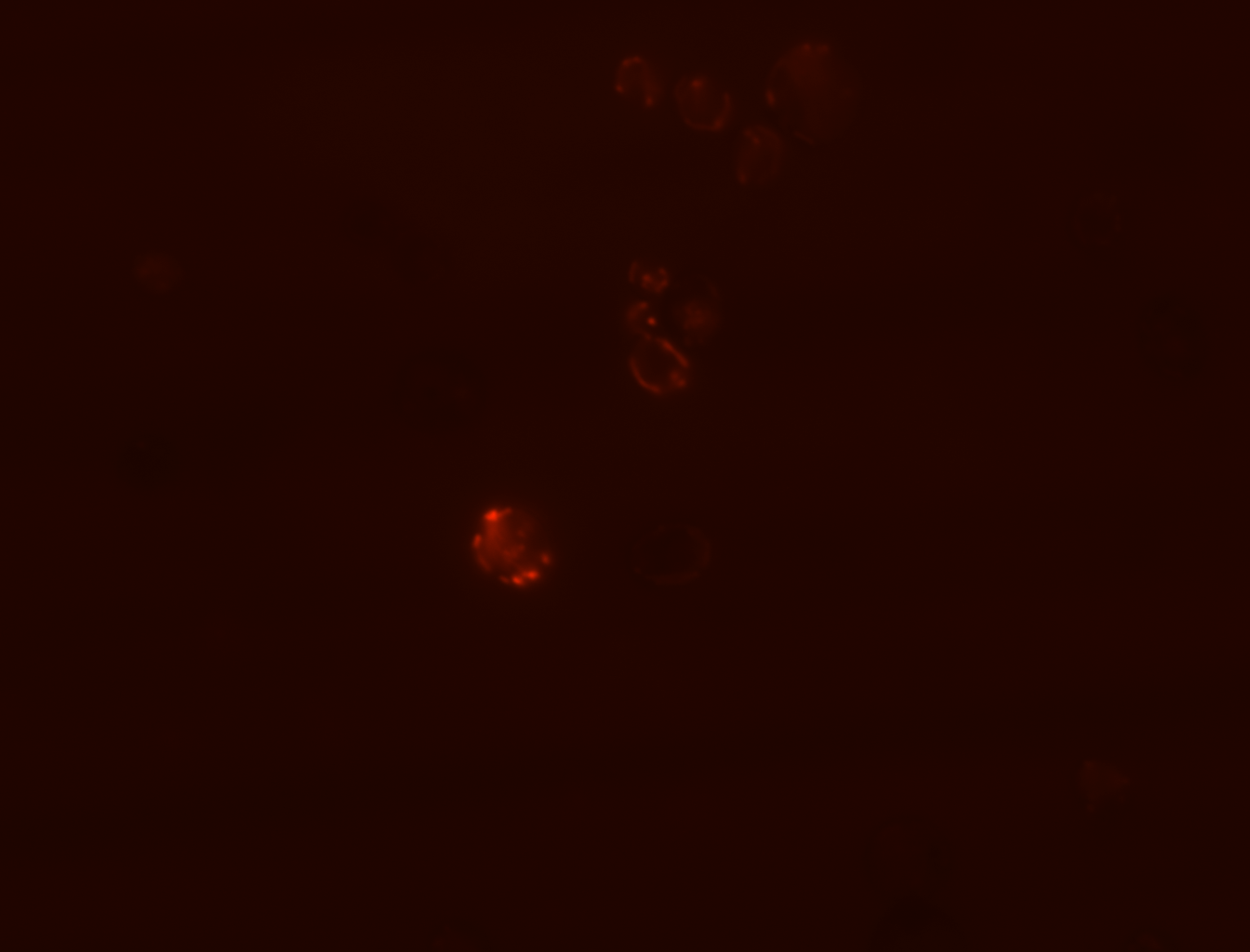

Supplement: Supplementary file 9 — Source data Fig. 1 [file 44319_2026_774_MOESM9_ESM.zip › Figure 1/1G/vma1_mCherry.TIF]

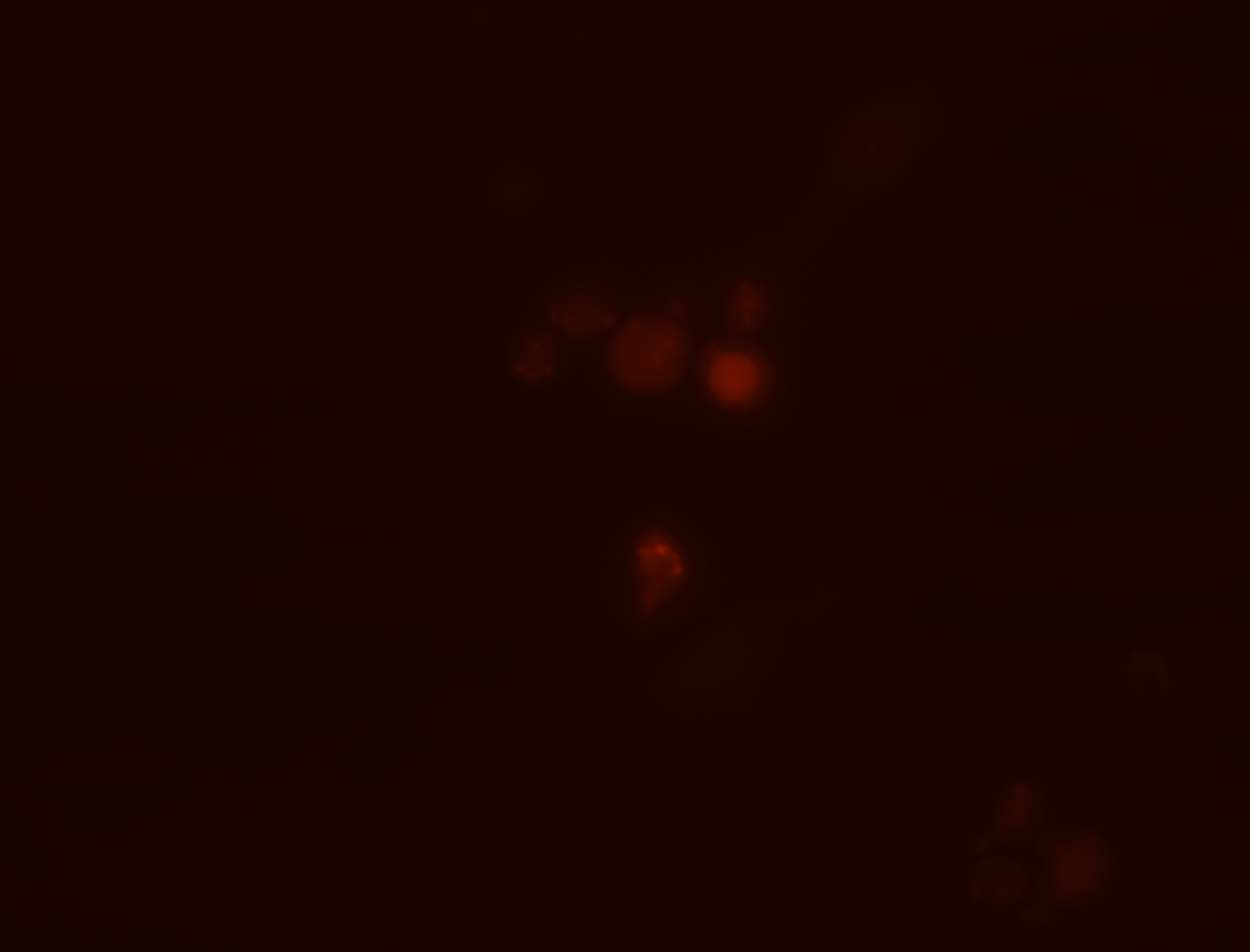

Supplement: Supplementary file 9 — Source data Fig. 1 [file 44319_2026_774_MOESM9_ESM.zip › Figure 1/1G/vma1 GAL10-AAC2_mCherry.TIF]

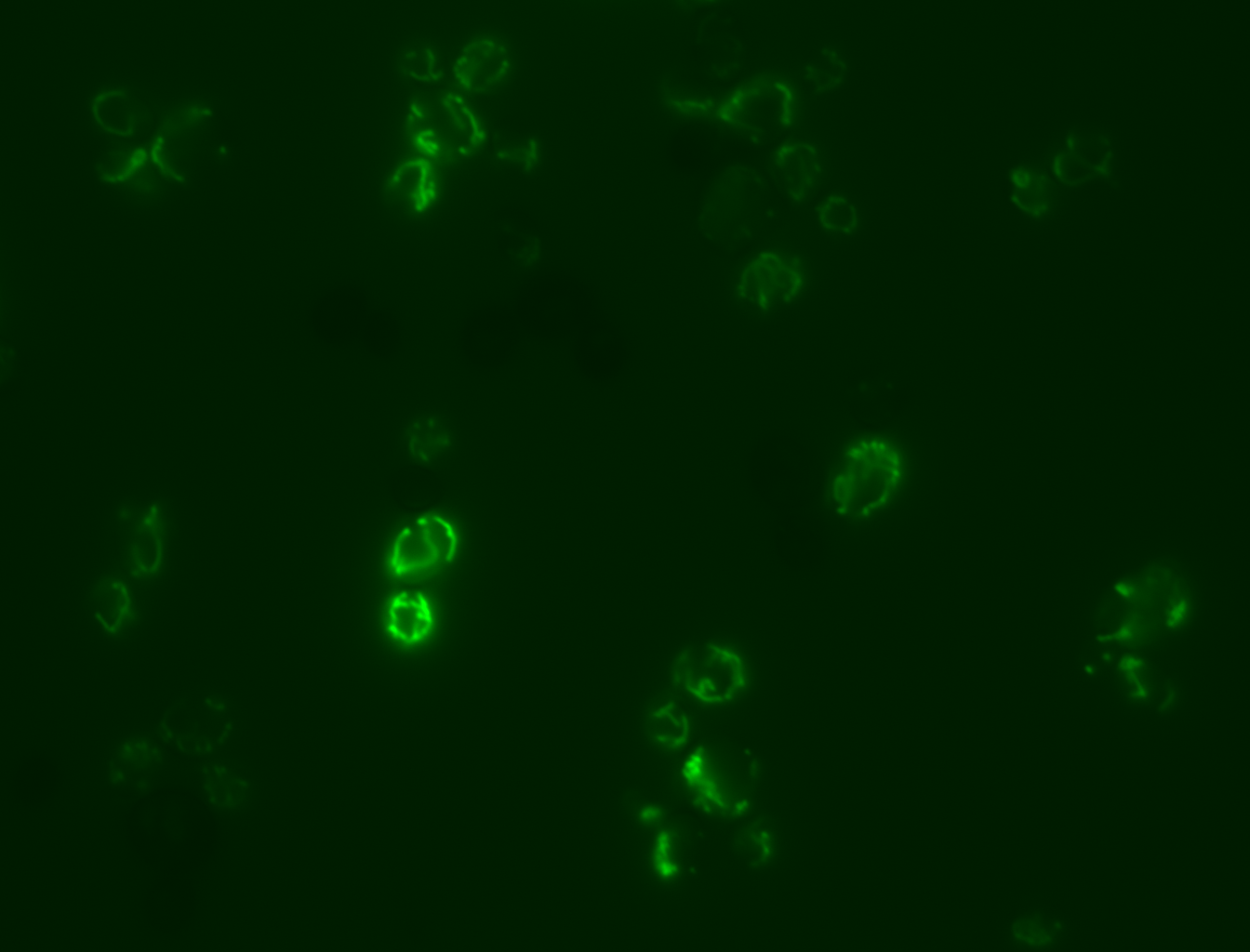

Supplement: Supplementary file 9 — Source data Fig. 1 [file 44319_2026_774_MOESM9_ESM.zip › Figure 1/1G/GAL10-AAC2_EGFP.TIF]

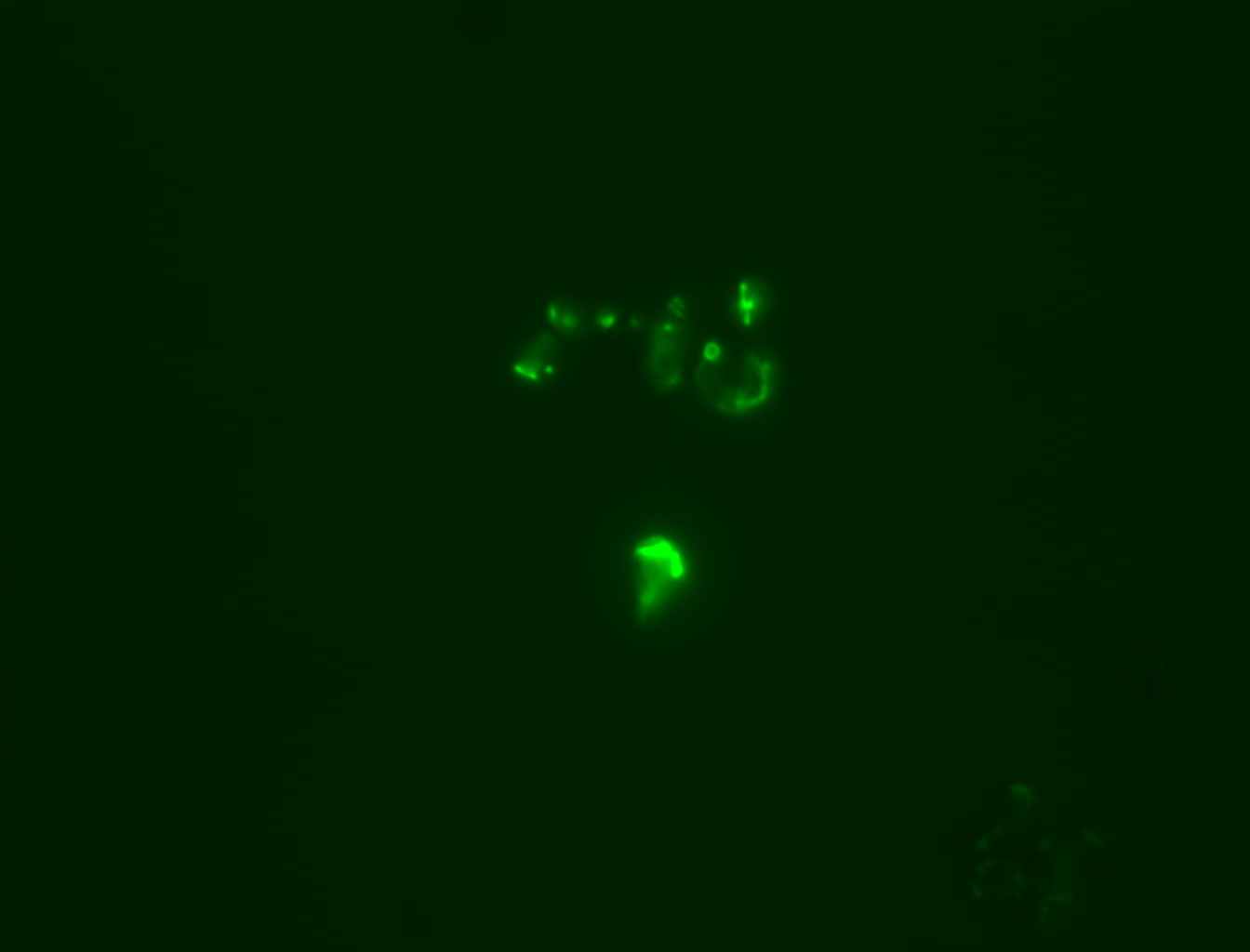

Supplement: Supplementary file 9 — Source data Fig. 1 [file 44319_2026_774_MOESM9_ESM.zip › Figure 1/1G/vma1 GAL10-AAC2_EGFP.TIF]

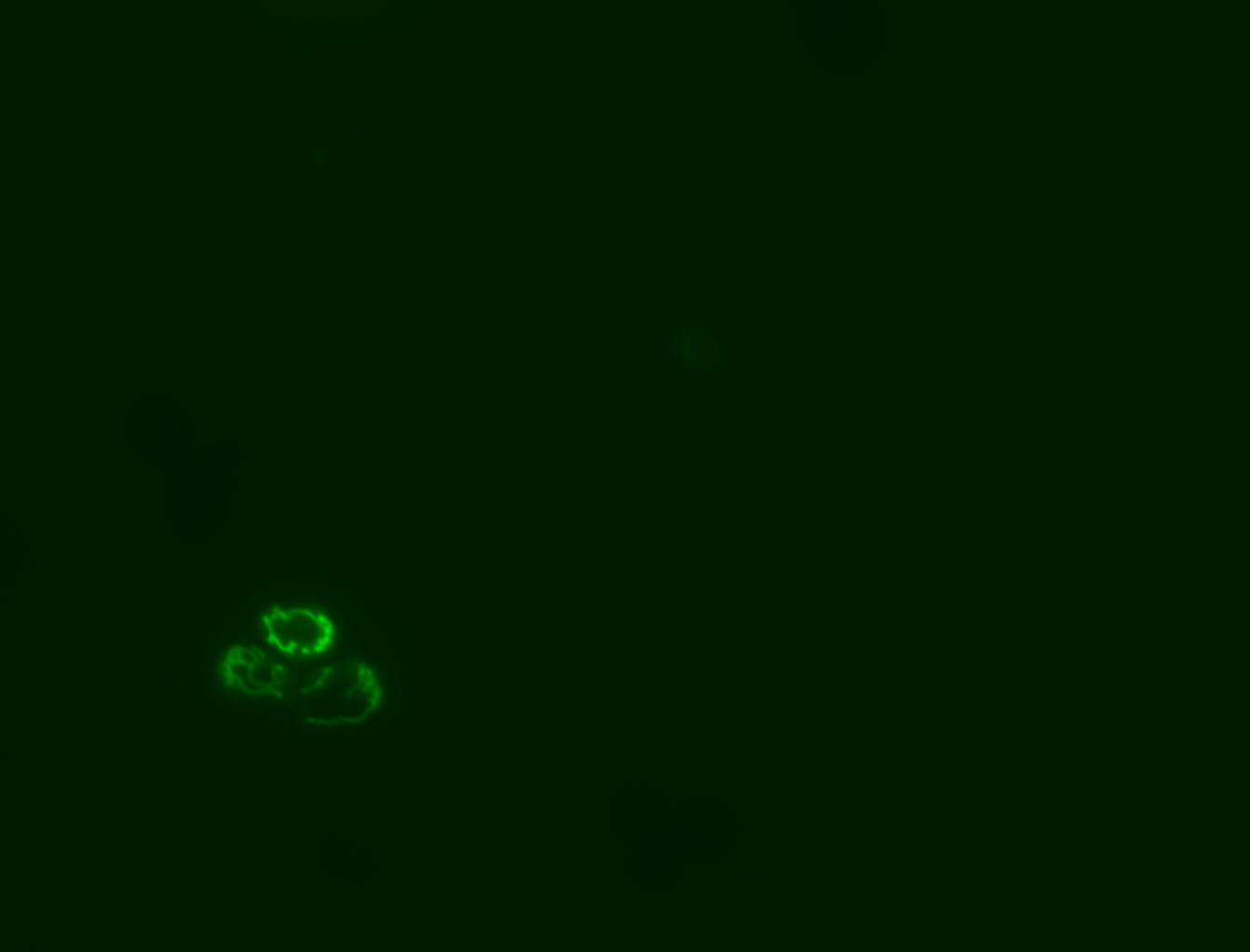

Supplement: Supplementary file 9 — Source data Fig. 1 [file 44319_2026_774_MOESM9_ESM.zip › Figure 1/1G/WT_EGFP.TIF]

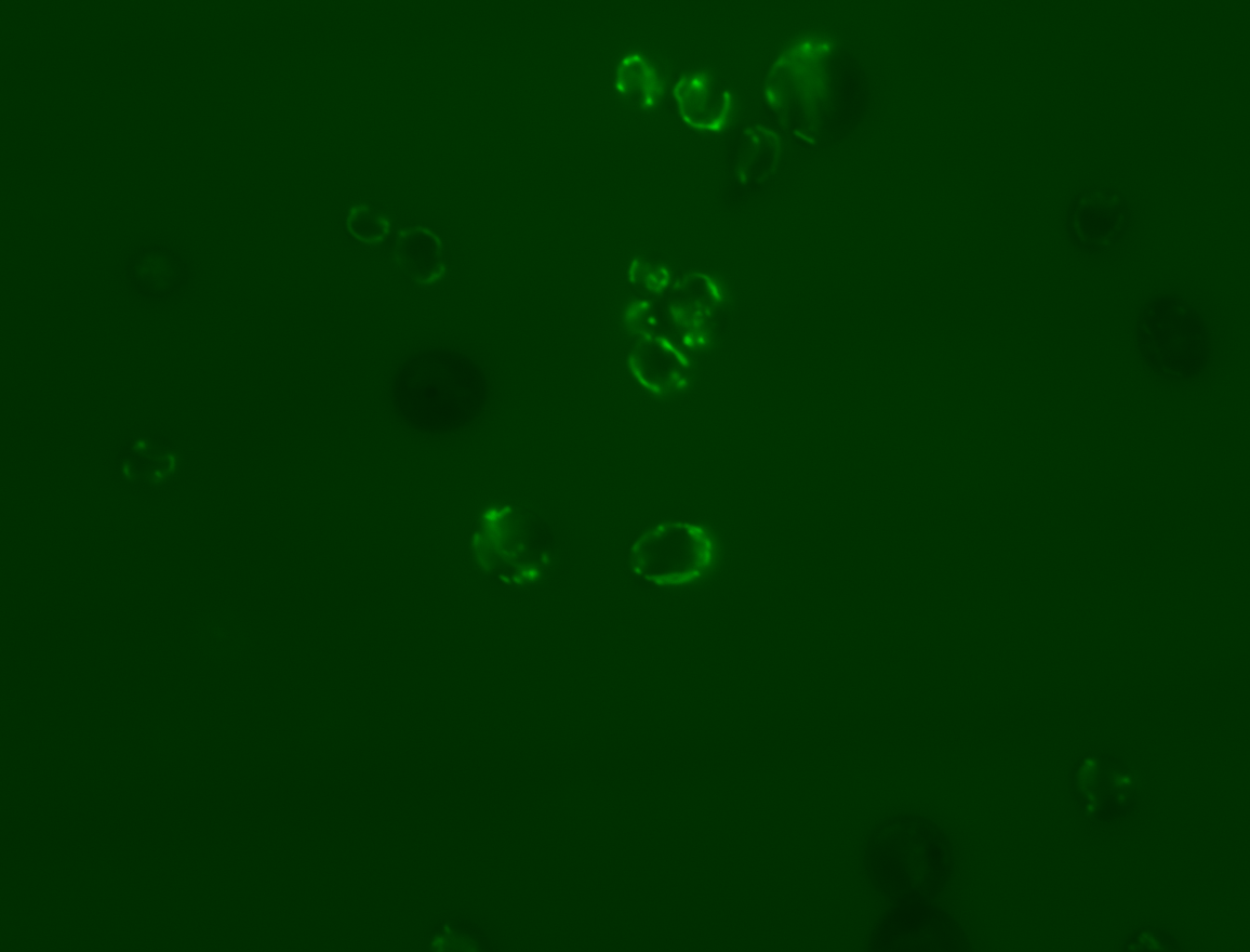

Supplement: Supplementary file 9 — Source data Fig. 1 [file 44319_2026_774_MOESM9_ESM.zip › Figure 1/1G/vma1_EGFP.TIF]

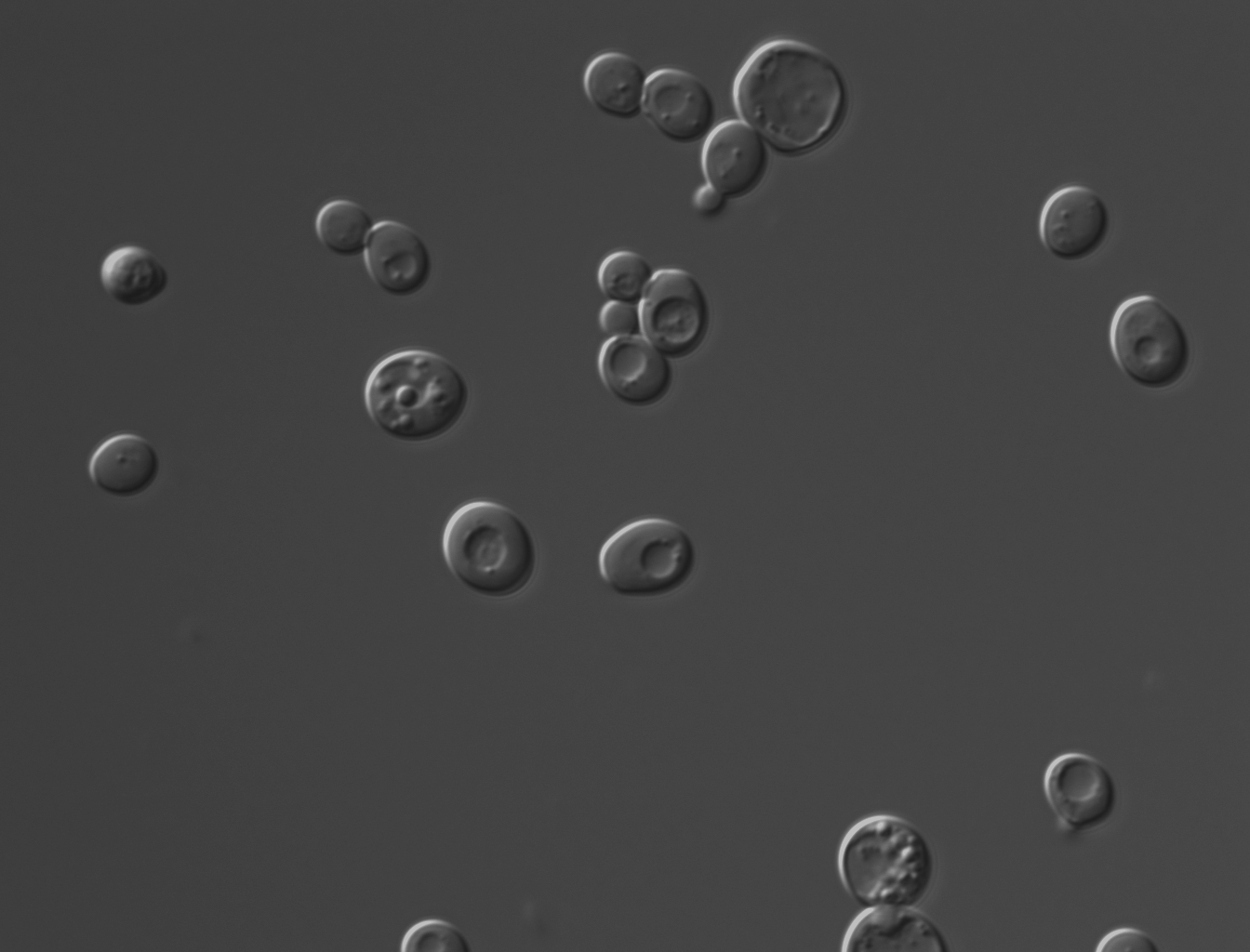

Supplement: Supplementary file 9 — Source data Fig. 1 [file 44319_2026_774_MOESM9_ESM.zip › Figure 1/1G/vma1_DIC.TIF]

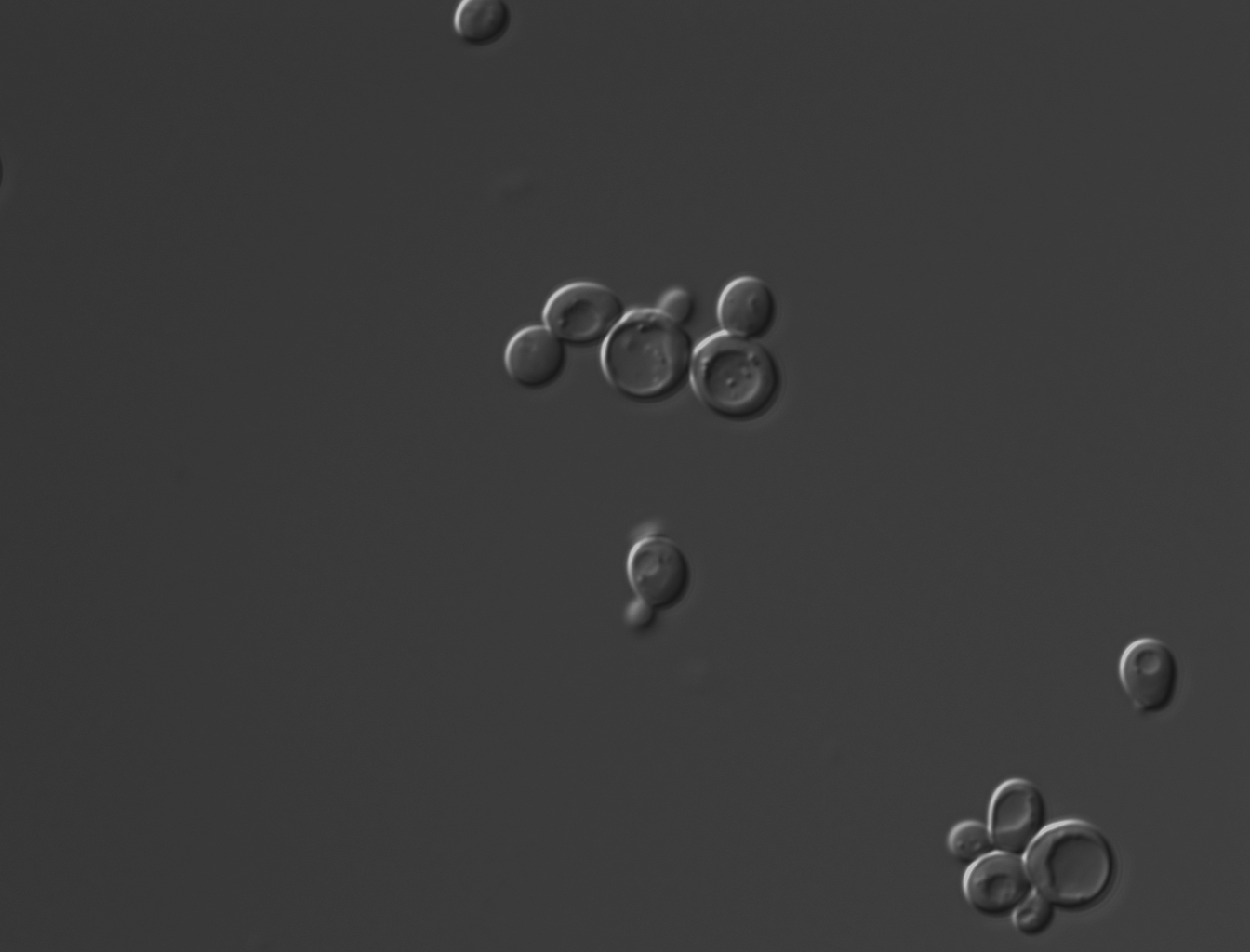

Supplement: Supplementary file 9 — Source data Fig. 1 [file 44319_2026_774_MOESM9_ESM.zip › Figure 1/1G/vma1 GAL10-AAC2_DIC.TIF]

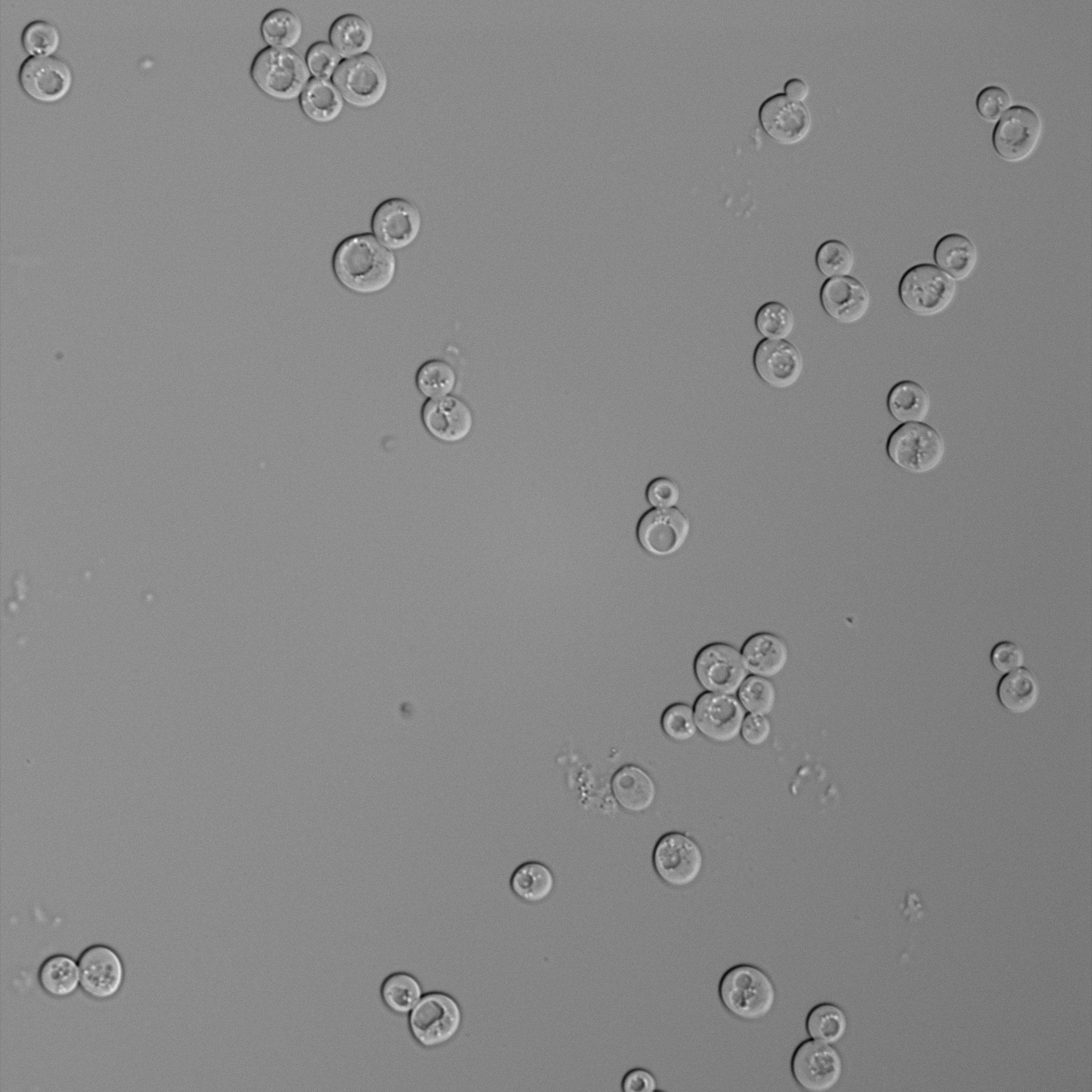

Supplement: Supplementary file 9 — Source data Fig. 1 [file 44319_2026_774_MOESM9_ESM.zip › Figure 1/1I/vma1_GAL10-AAC2_Aco1-mNG_YPD_30C_12-26-25_3.tif]

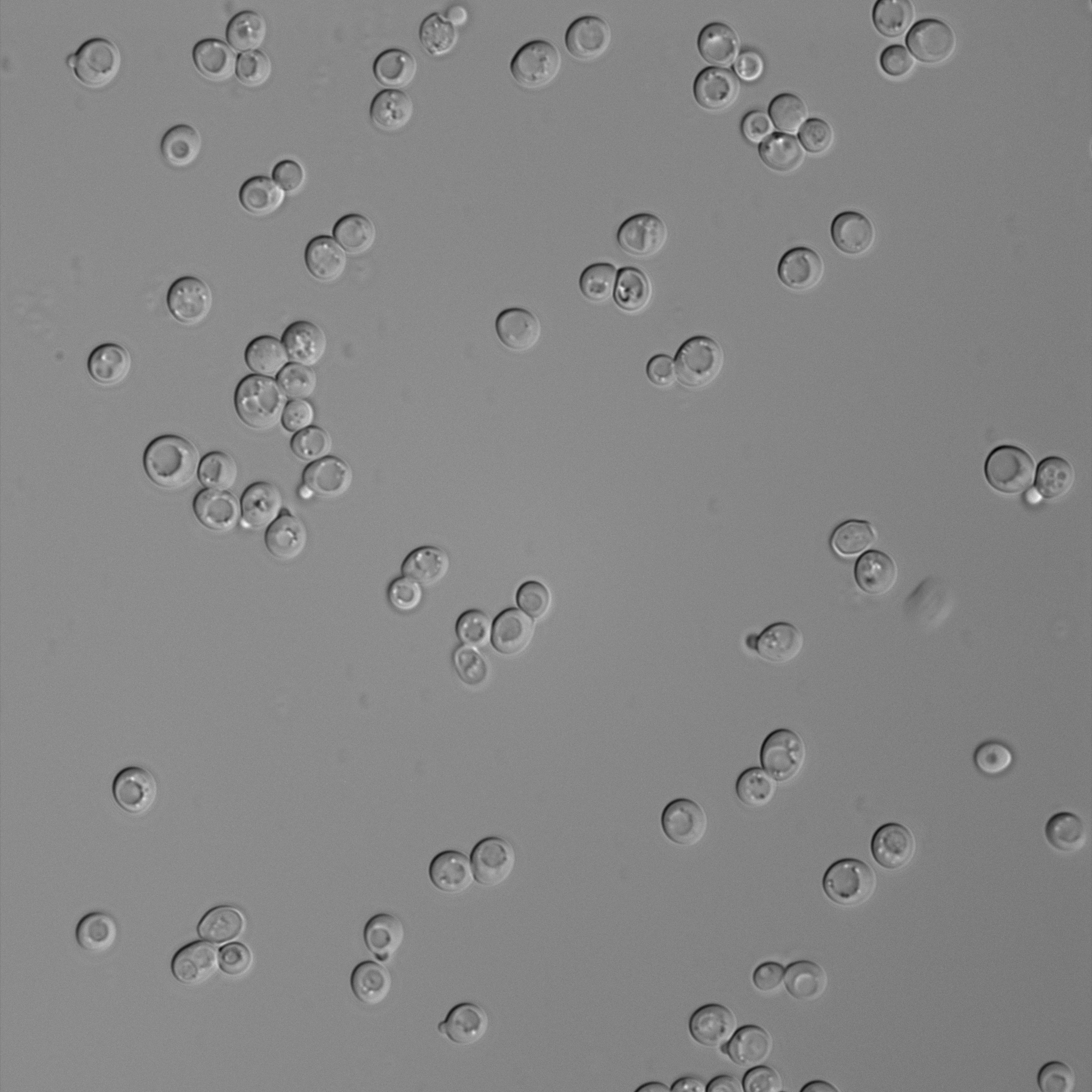

Supplement: Supplementary file 9 — Source data Fig. 1 [file 44319_2026_774_MOESM9_ESM.zip › Figure 1/1I/vma1_GAL10-AAC2_Aco1-mNG_YPGR_30C_12-27-25_33.tif]

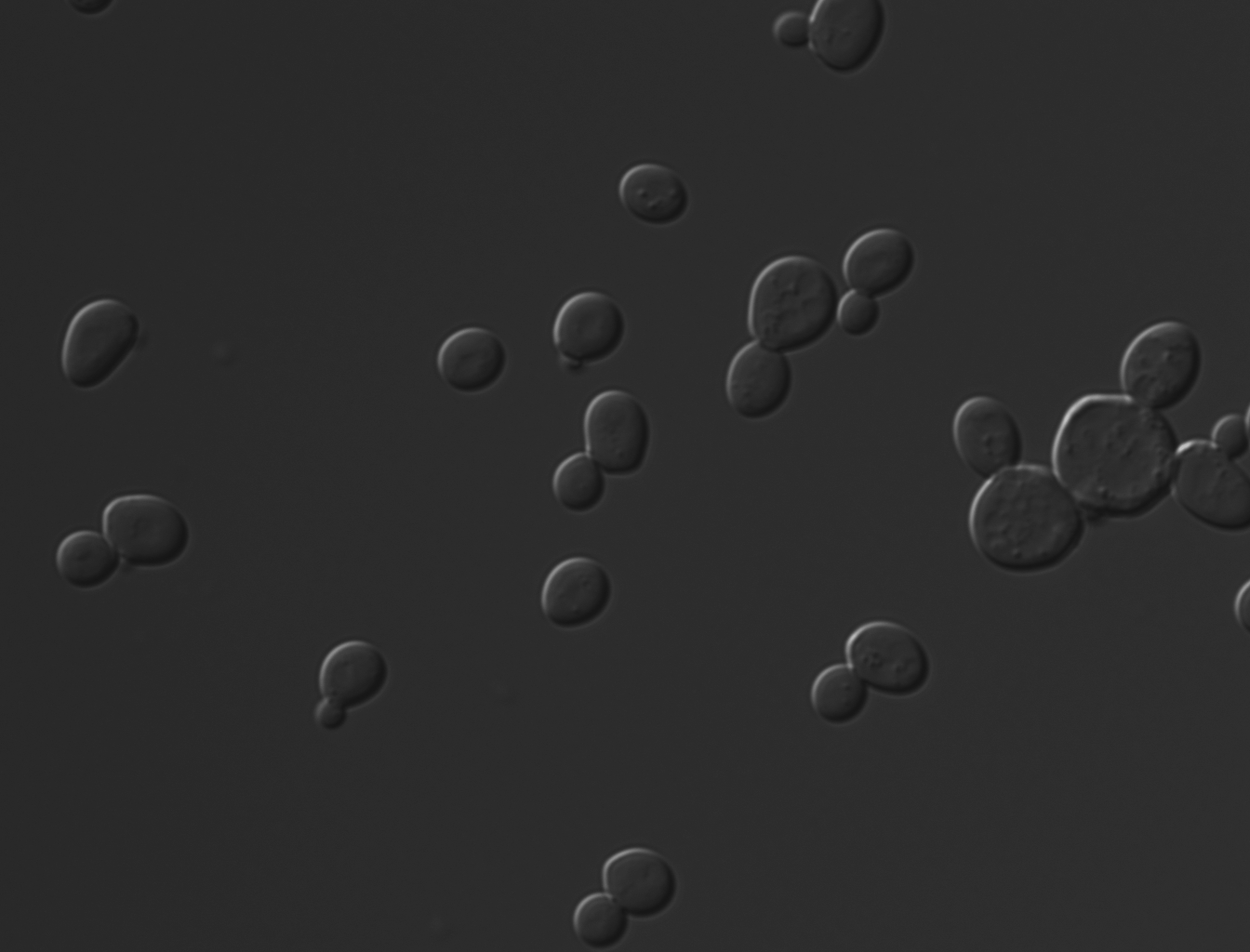

Supplement: Supplementary file 9 — Source data Fig. 1 [file 44319_2026_774_MOESM9_ESM.zip › Figure 1/1L/WT_DIC.TIF]

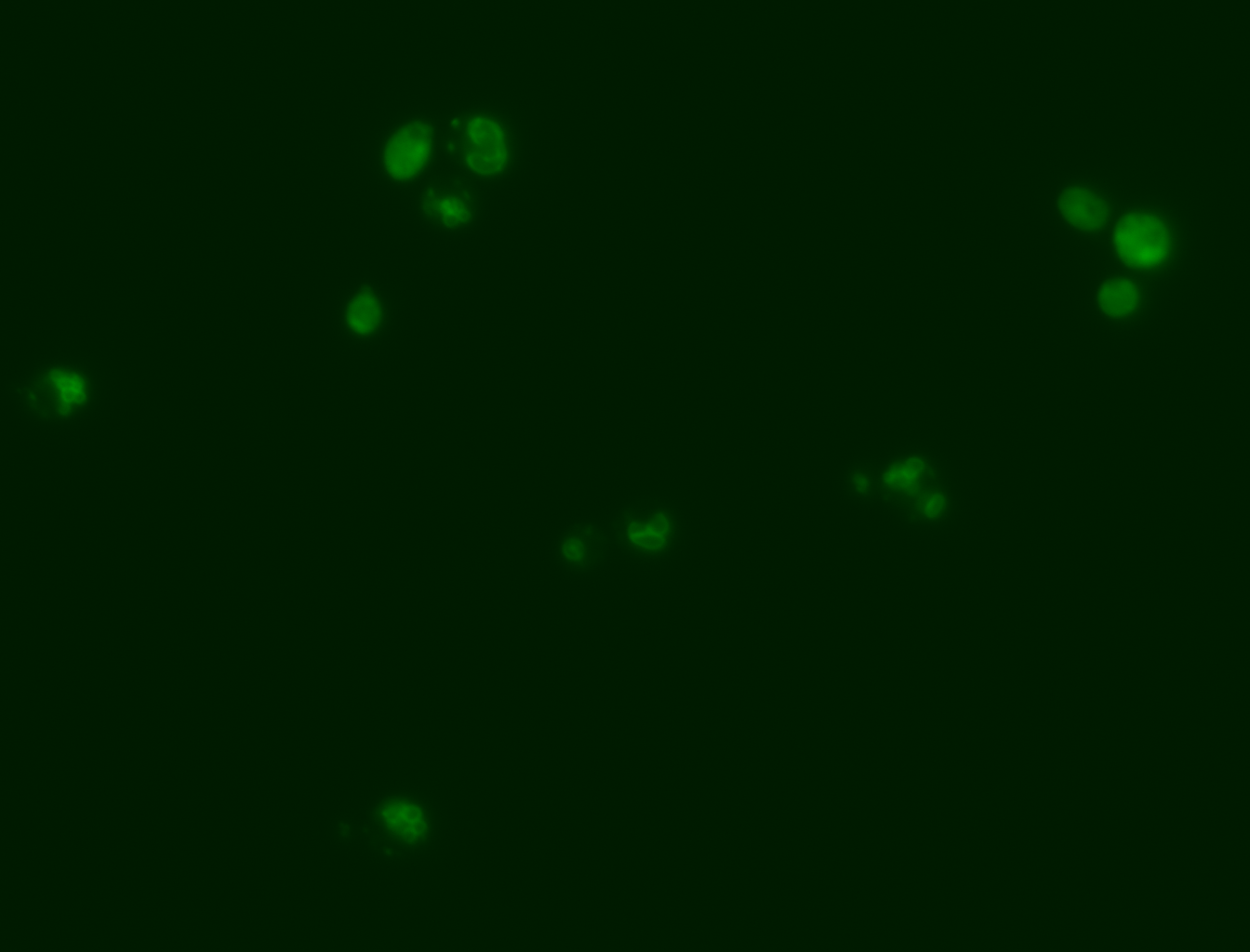

Supplement: Supplementary file 9 — Source data Fig. 1 [file 44319_2026_774_MOESM9_ESM.zip › Figure 1/1L/aac2(A128P)_GFP.TIF]

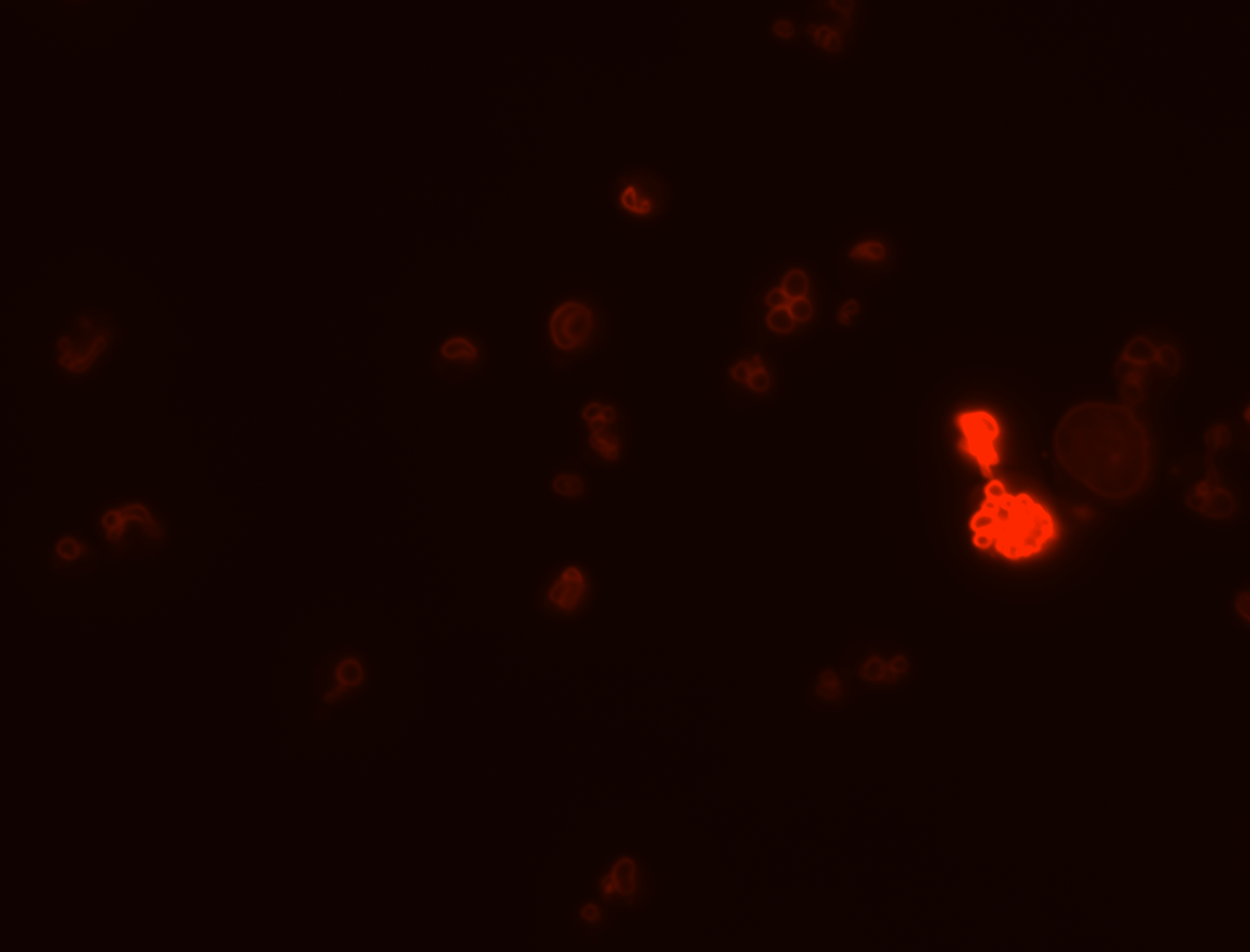

Supplement: Supplementary file 9 — Source data Fig. 1 [file 44319_2026_774_MOESM9_ESM.zip › Figure 1/1L/WT_FM4-64.TIF]

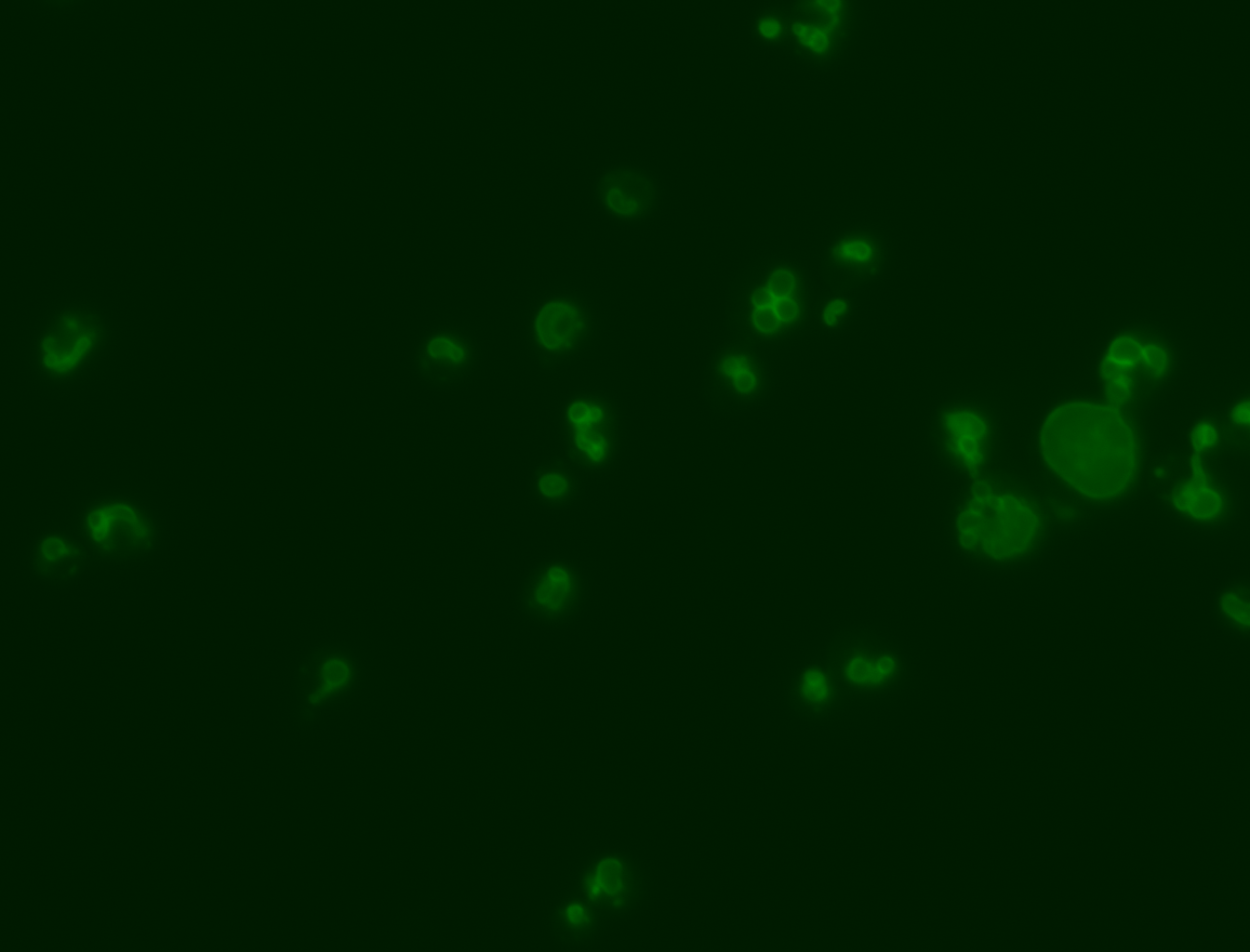

Supplement: Supplementary file 9 — Source data Fig. 1 [file 44319_2026_774_MOESM9_ESM.zip › Figure 1/1L/WT_GFP.TIF]

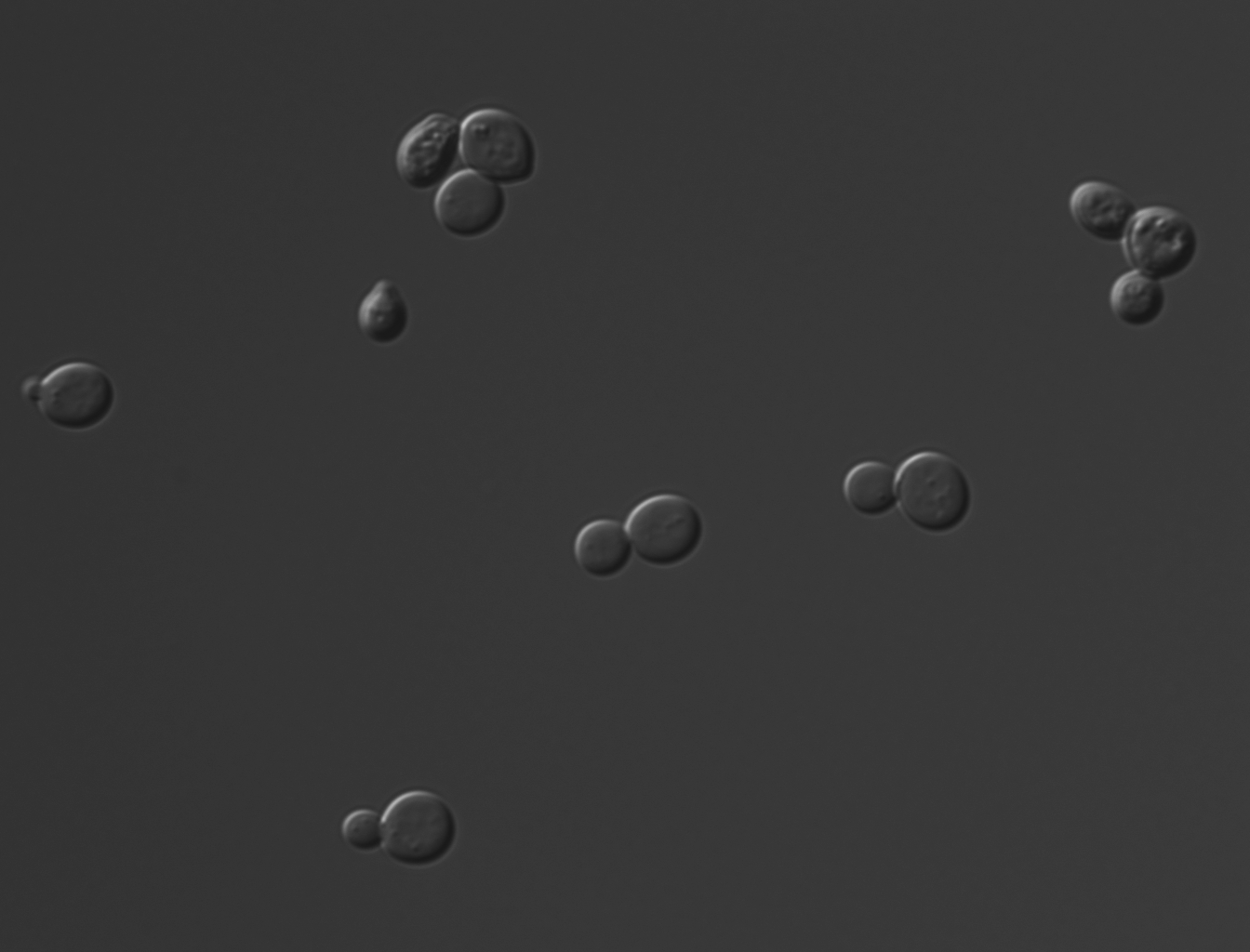

Supplement: Supplementary file 9 — Source data Fig. 1 [file 44319_2026_774_MOESM9_ESM.zip › Figure 1/1L/aac2(A128P)_DIC.TIF]

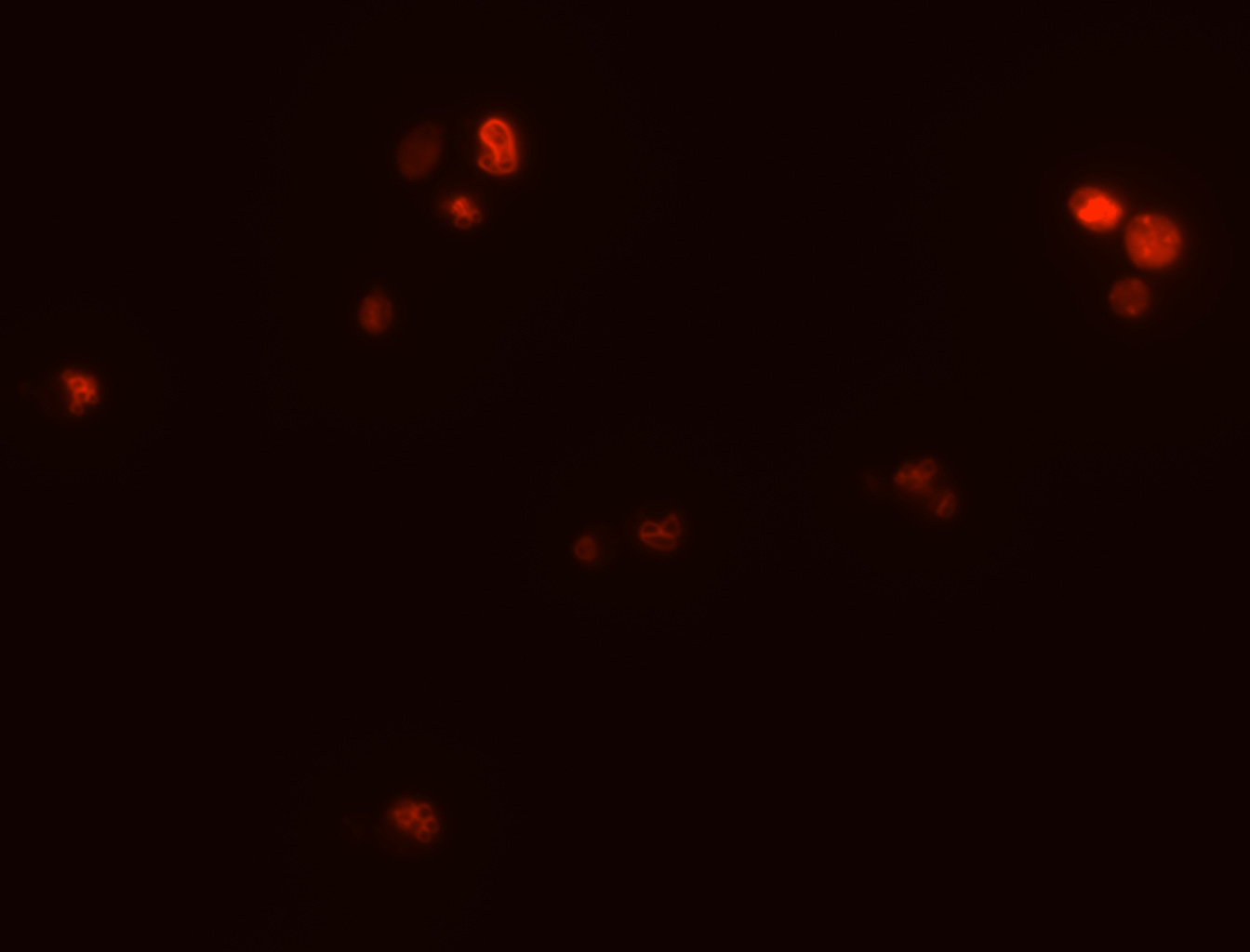

Supplement: Supplementary file 9 — Source data Fig. 1 [file 44319_2026_774_MOESM9_ESM.zip › Figure 1/1L/aac2(A128P)_FM4-64.TIF]

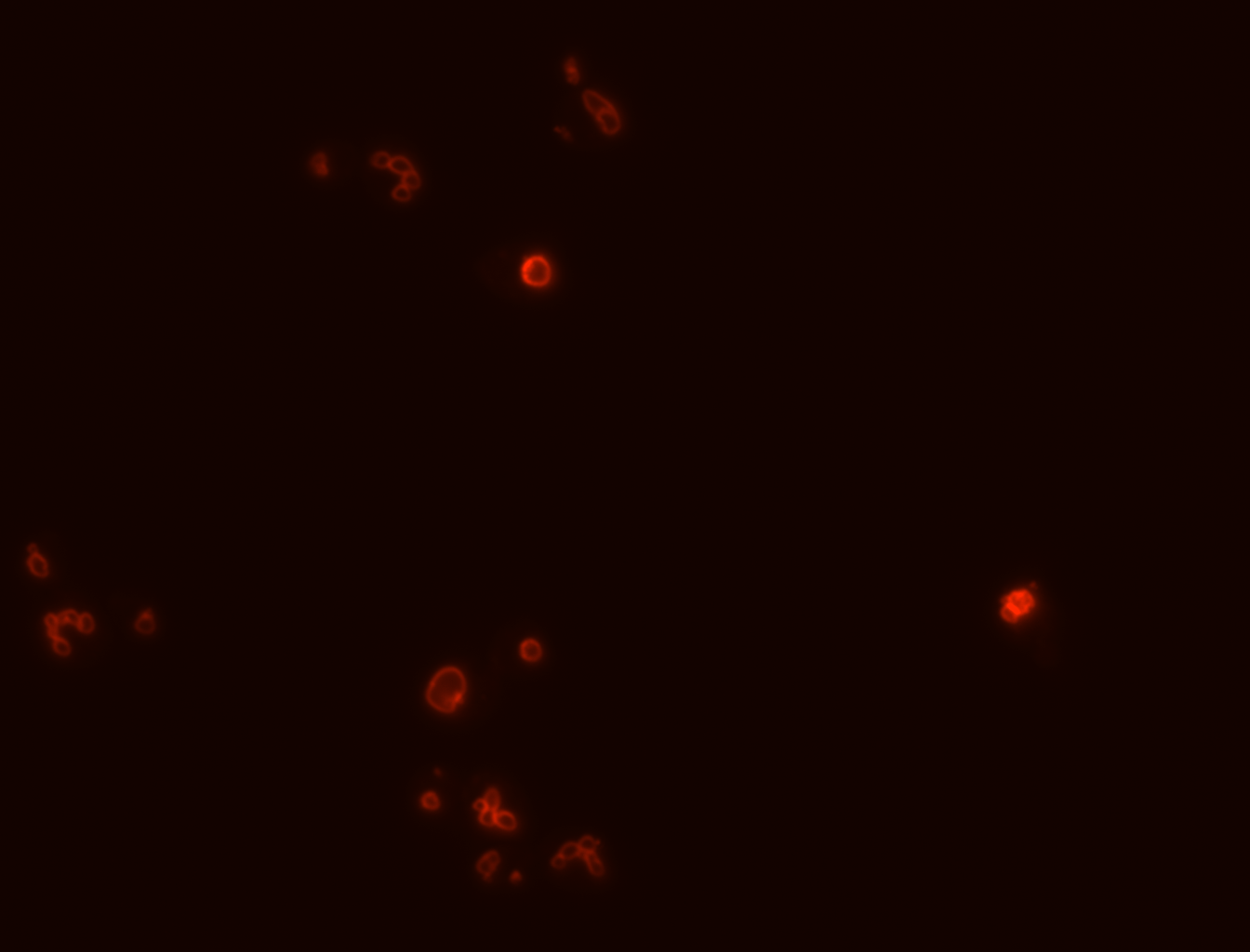

Supplement: Supplementary file 9 — Source data Fig. 1 [file 44319_2026_774_MOESM9_ESM.zip › Figure 1/1B/Wildtype FM4-64 vacuolar membrane image.TIF]

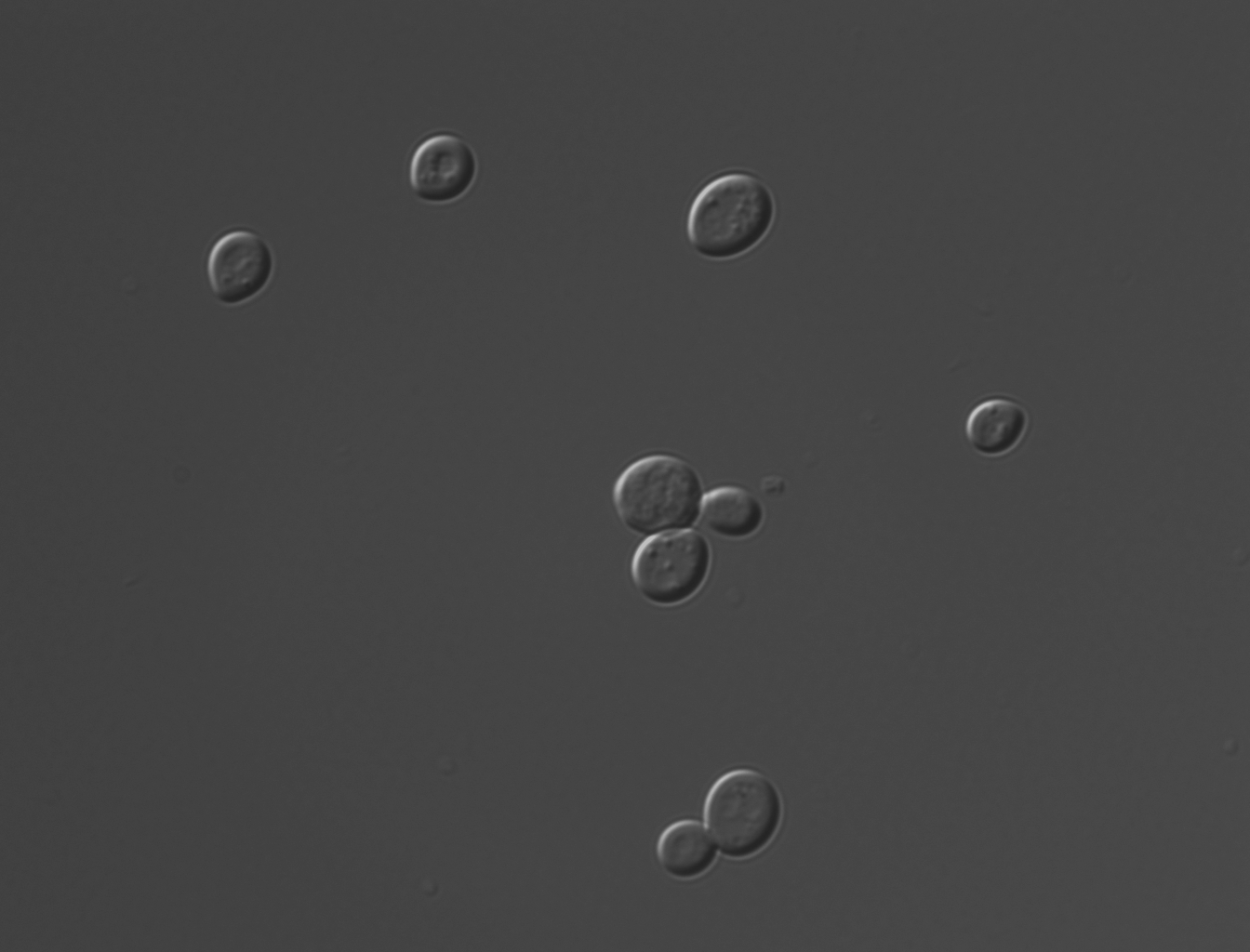

Supplement: Supplementary file 9 — Source data Fig. 1 [file 44319_2026_774_MOESM9_ESM.zip › Figure 1/1B/A128P DIC image.TIF]

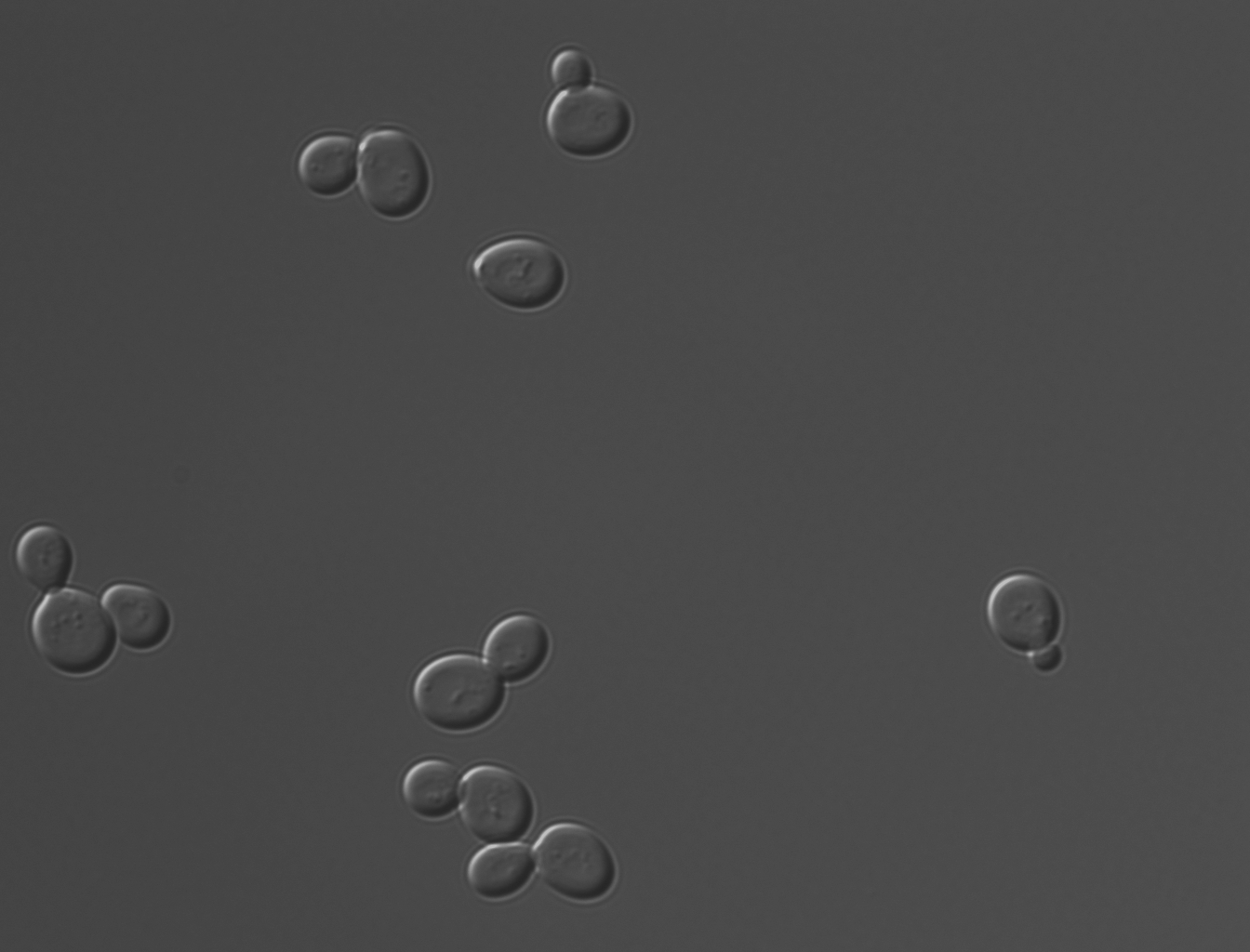

Supplement: Supplementary file 9 — Source data Fig. 1 [file 44319_2026_774_MOESM9_ESM.zip › Figure 1/1B/Wildtype DIC image.TIF]

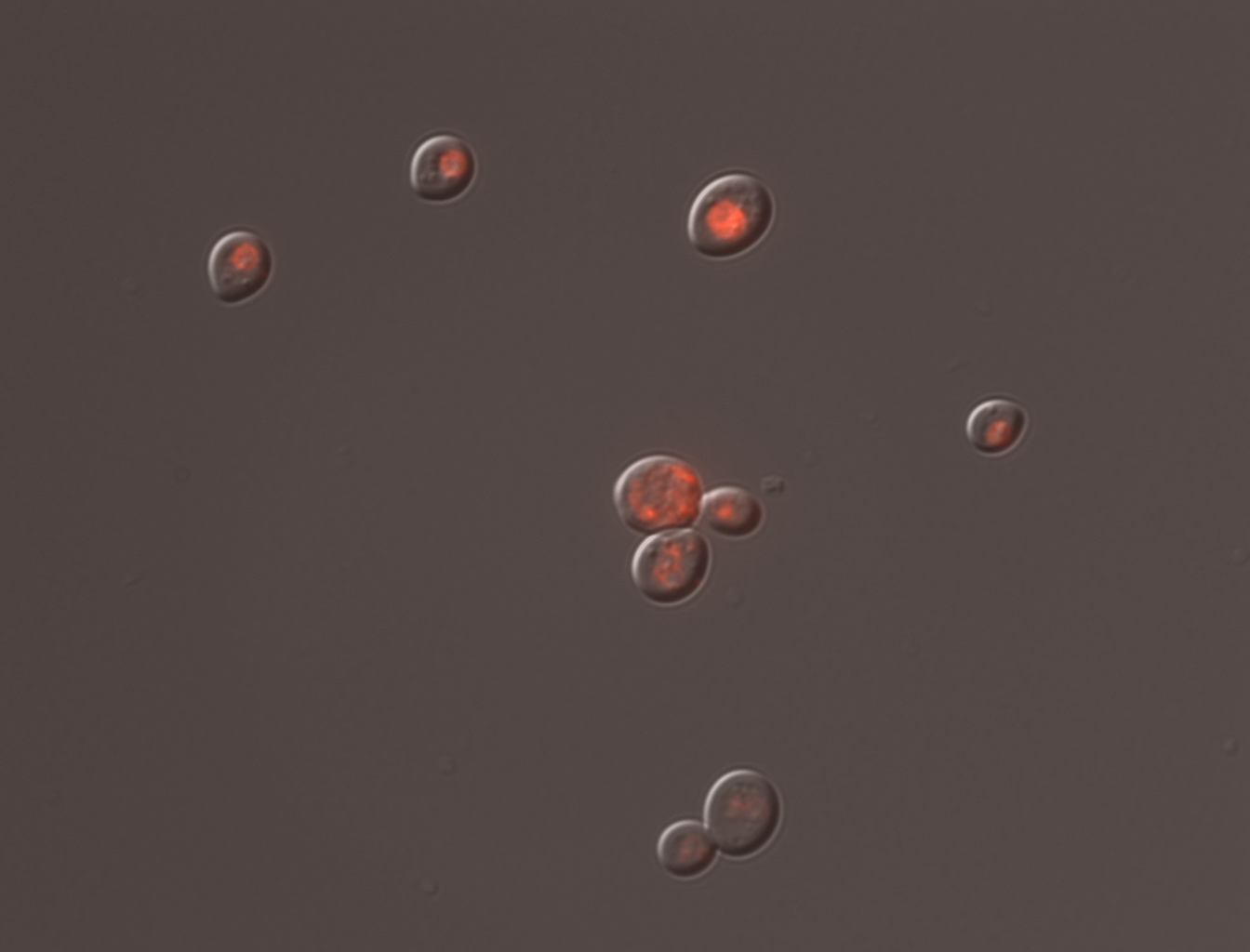

Supplement: Supplementary file 9 — Source data Fig. 1 [file 44319_2026_774_MOESM9_ESM.zip › Figure 1/1B/A128P overlay image.TIF]

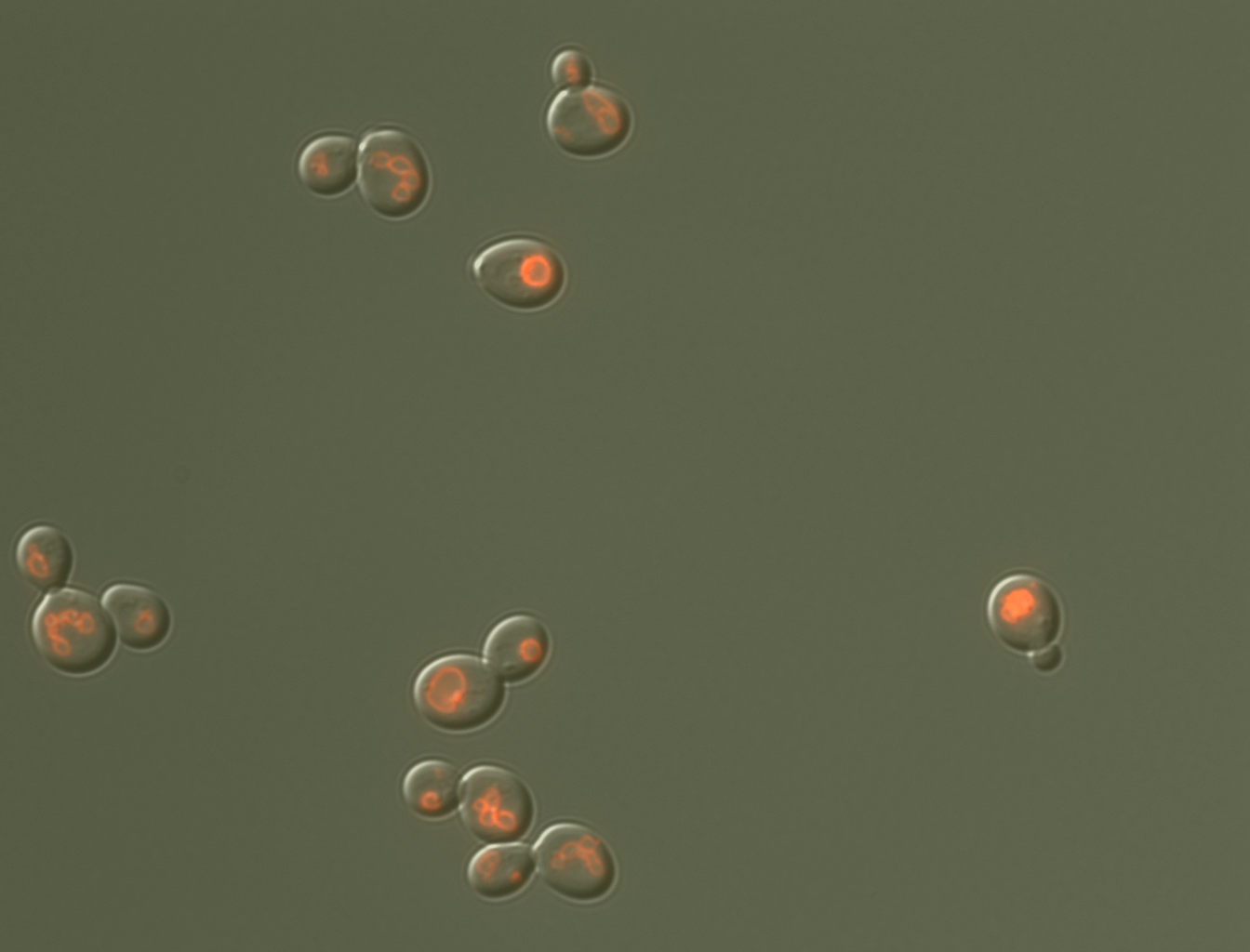

Supplement: Supplementary file 9 — Source data Fig. 1 [file 44319_2026_774_MOESM9_ESM.zip › Figure 1/1B/Wildype overlay image.TIF]

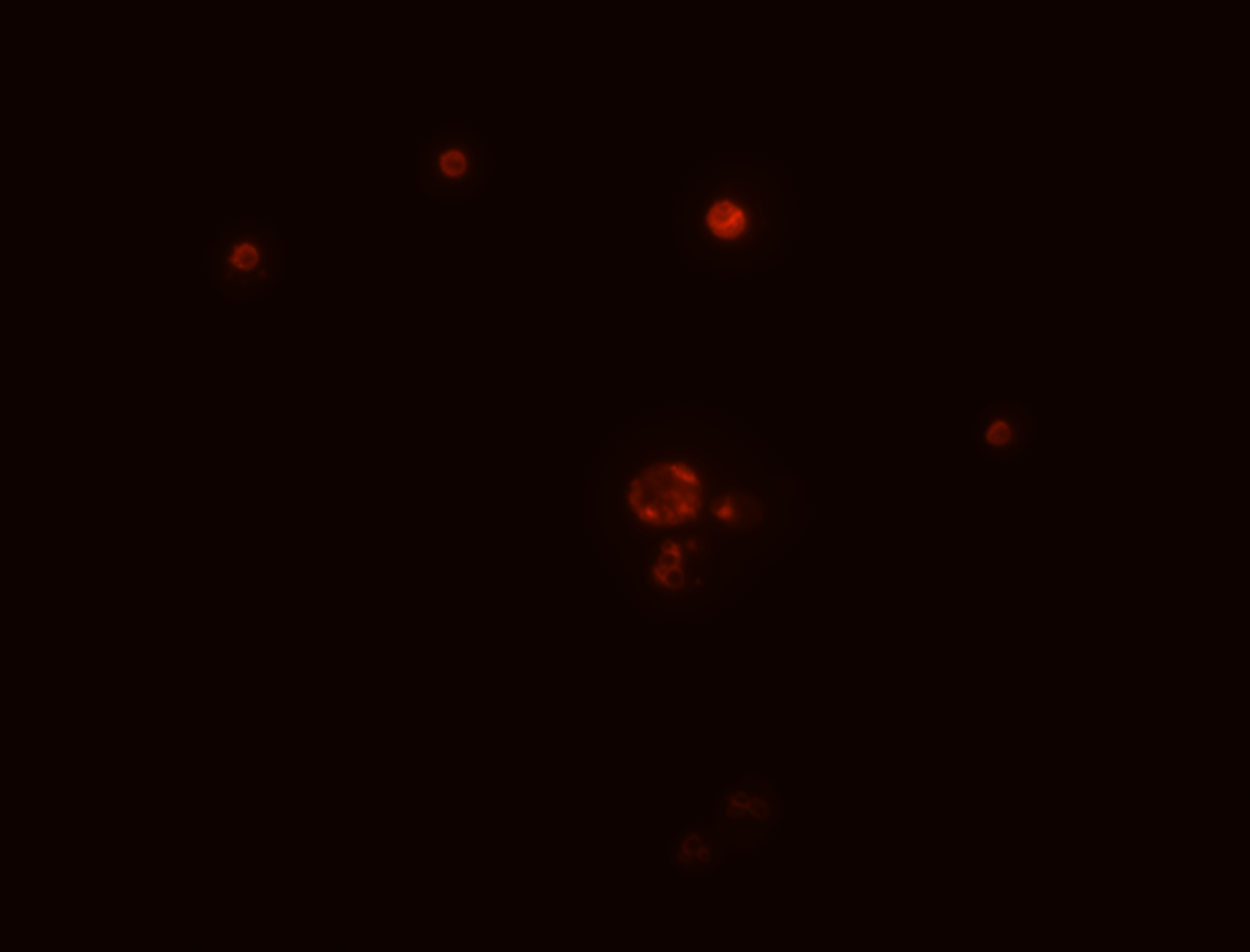

Supplement: Supplementary file 9 — Source data Fig. 1 [file 44319_2026_774_MOESM9_ESM.zip › Figure 1/1B/A128P FM4-64 vacuolar membrane image.TIF]

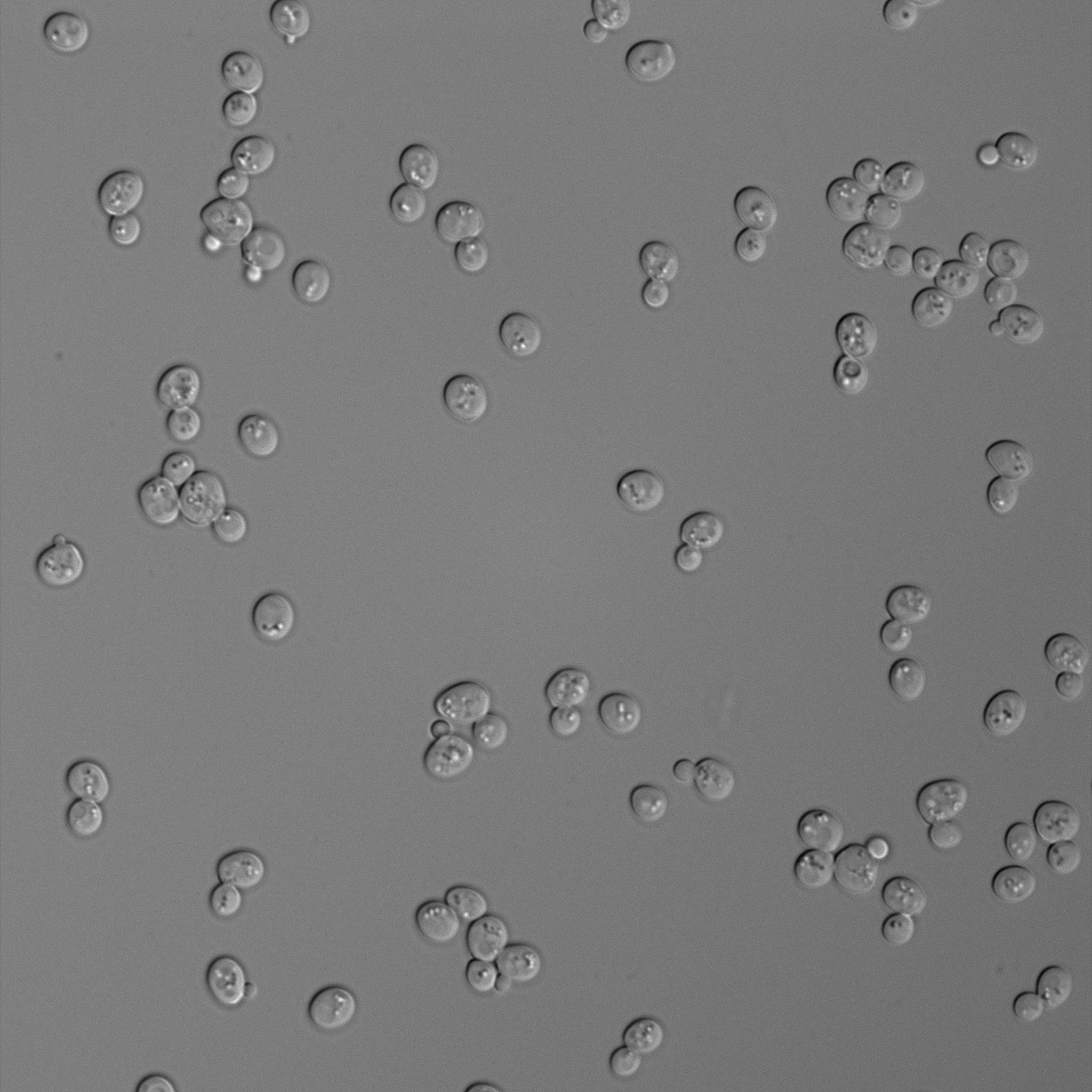

Supplement: Supplementary file 10 — Source data Fig. 2 [file 44319_2026_774_MOESM10_ESM.zip › Figure 2/2B/tom70_YPD_25C_Stationary phase_DIC.tif]

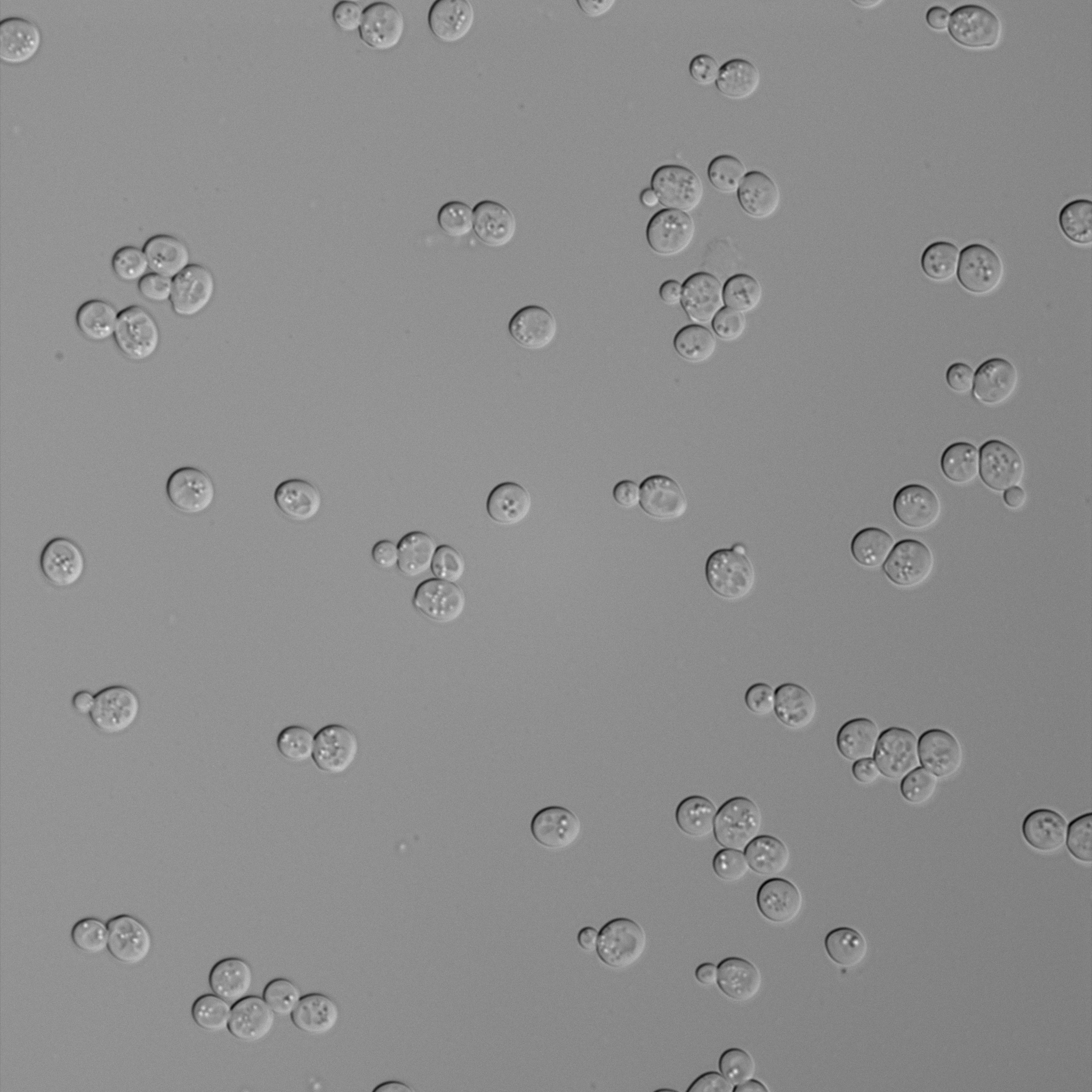

Supplement: Supplementary file 10 — Source data Fig. 2 [file 44319_2026_774_MOESM10_ESM.zip › Figure 2/2B/tom5_YPD_25C_Stationary phase_DIC.tif]

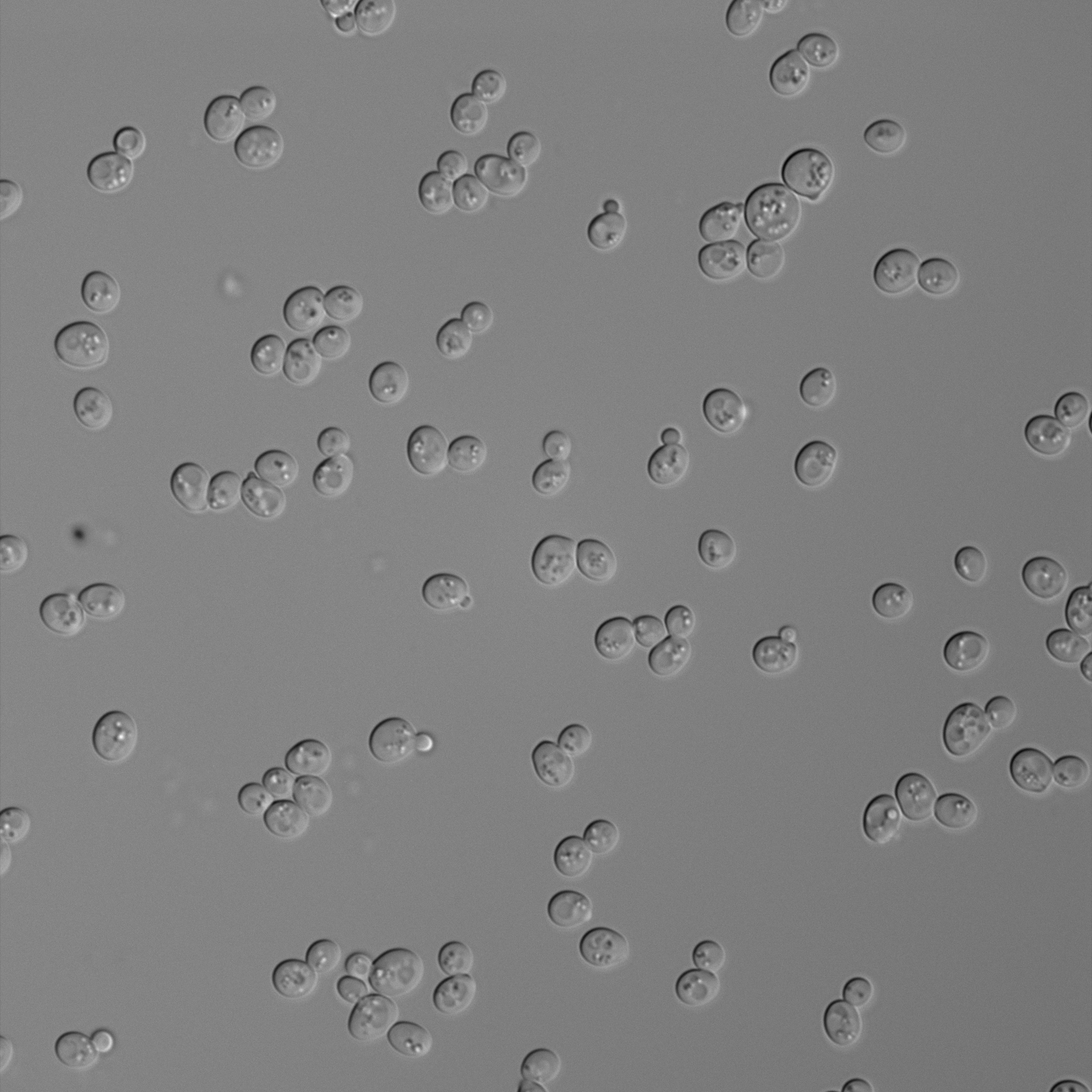

Supplement: Supplementary file 10 — Source data Fig. 2 [file 44319_2026_774_MOESM10_ESM.zip › Figure 2/2B/tom6_YPD_25C_Stationary phase_DIC.tif]

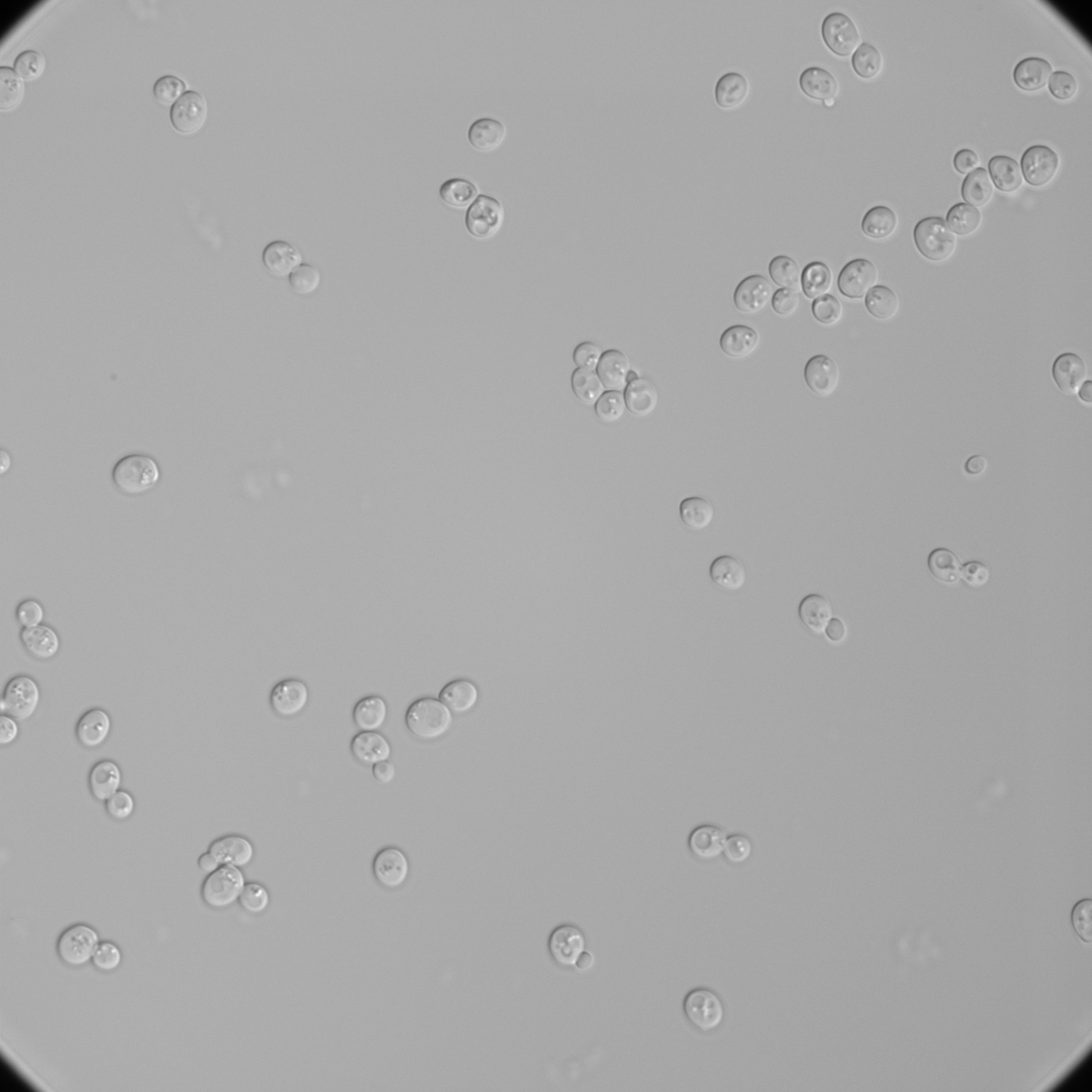

Supplement: Supplementary file 10 — Source data Fig. 2 [file 44319_2026_774_MOESM10_ESM.zip › Figure 2/2B/tim18_YPD_25C_Stationary phase_DIC.tif]

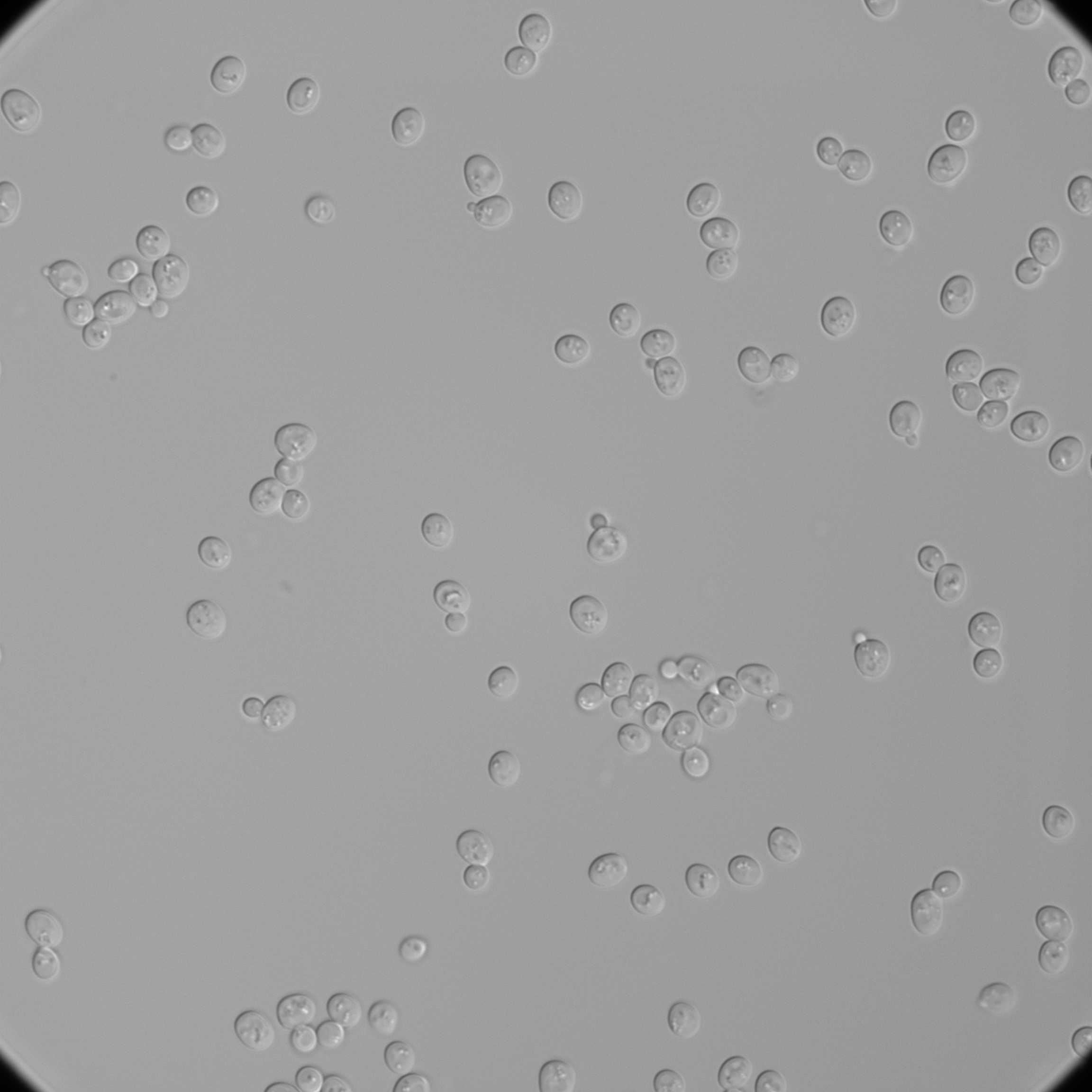

Supplement: Supplementary file 10 — Source data Fig. 2 [file 44319_2026_774_MOESM10_ESM.zip › Figure 2/2B/tom7_YPD_25C_Stationary phase_DIC.tif]

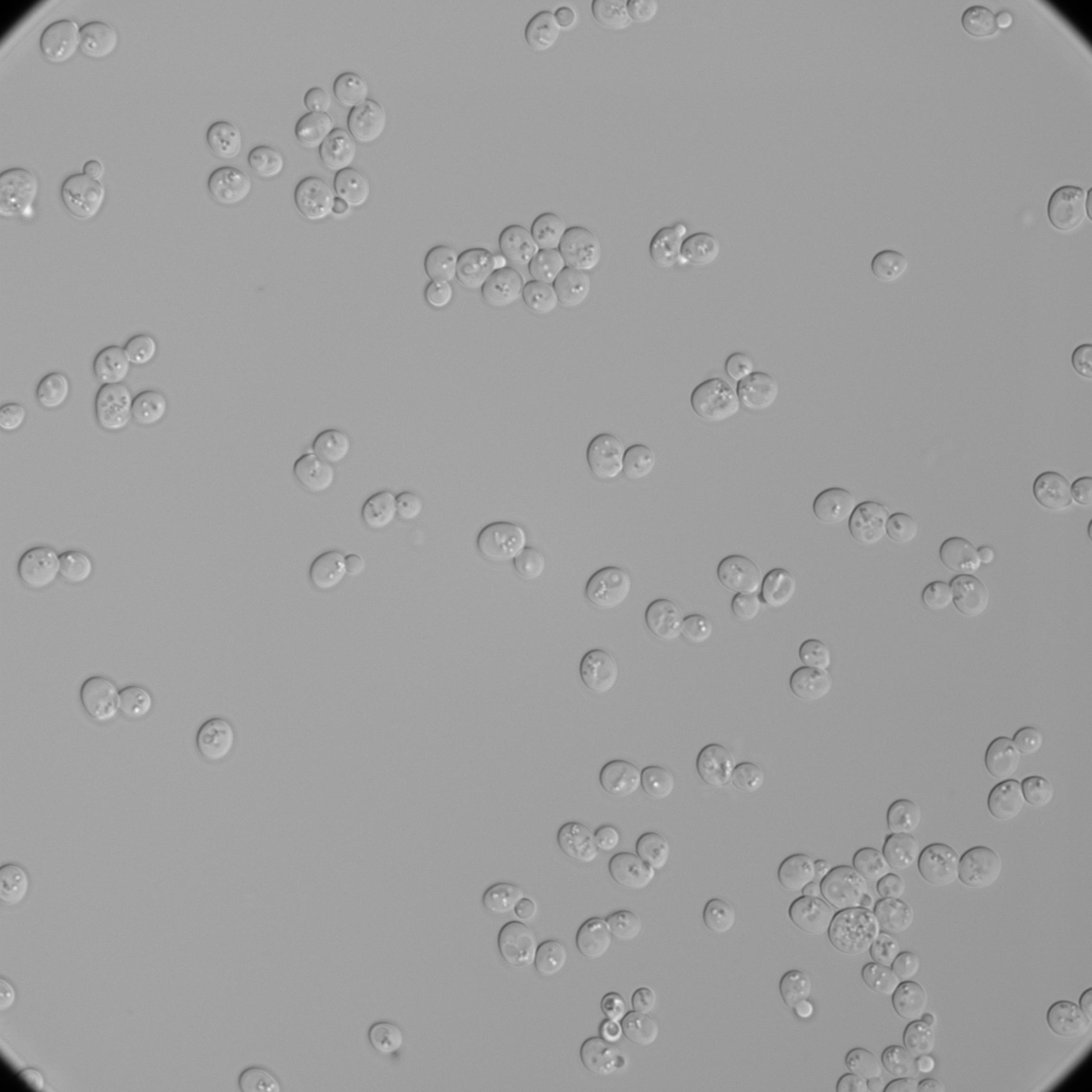

Supplement: Supplementary file 10 — Source data Fig. 2 [file 44319_2026_774_MOESM10_ESM.zip › Figure 2/2B/WT_YPD_25C_Stationary phase_DIC.tif]

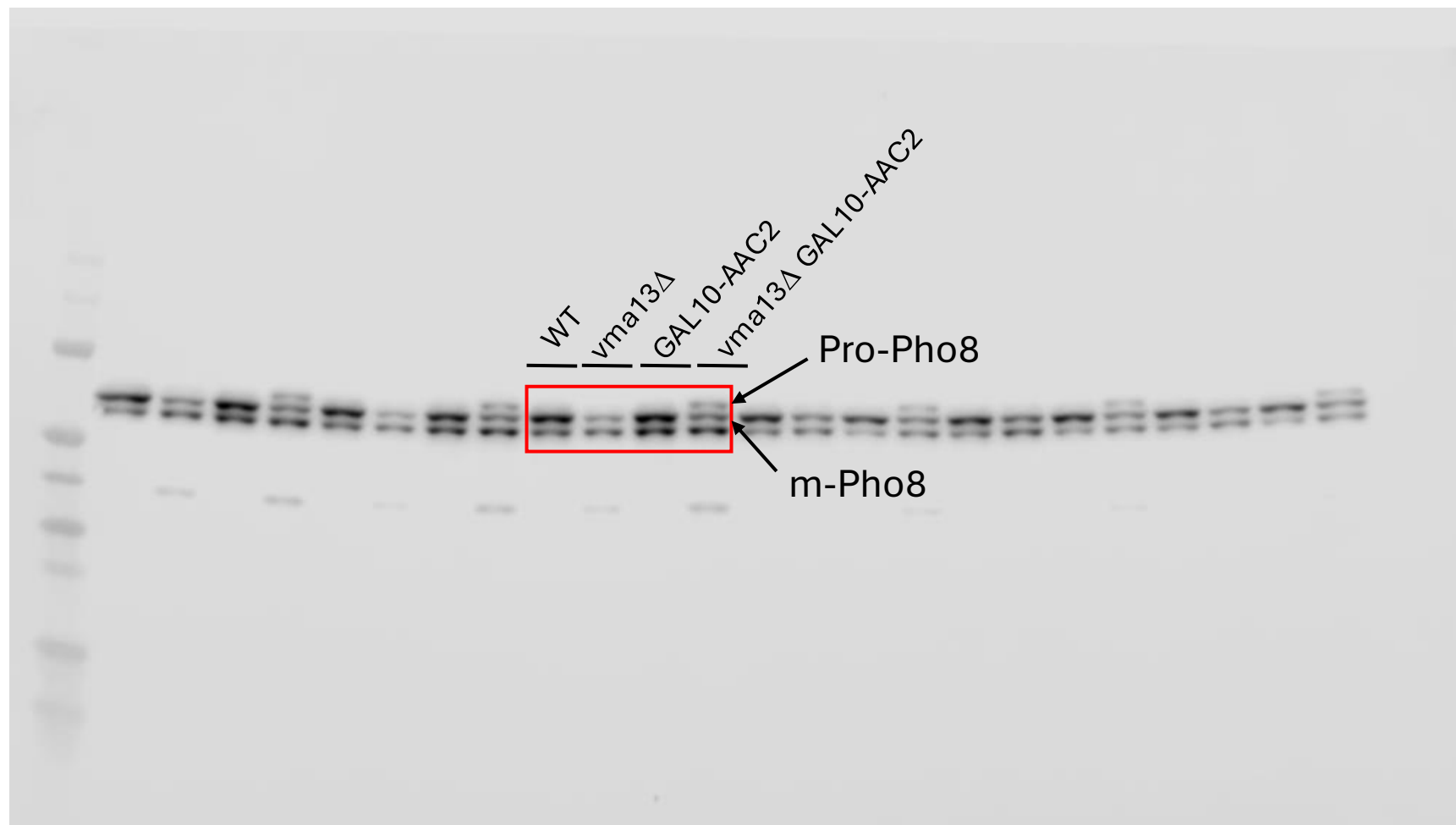

Supplement: Supplementary file 12 — Source data Fig. 4 [file 44319_2026_774_MOESM12_ESM.zip › Figure 4/4G/Pho8 western blot.pdf]

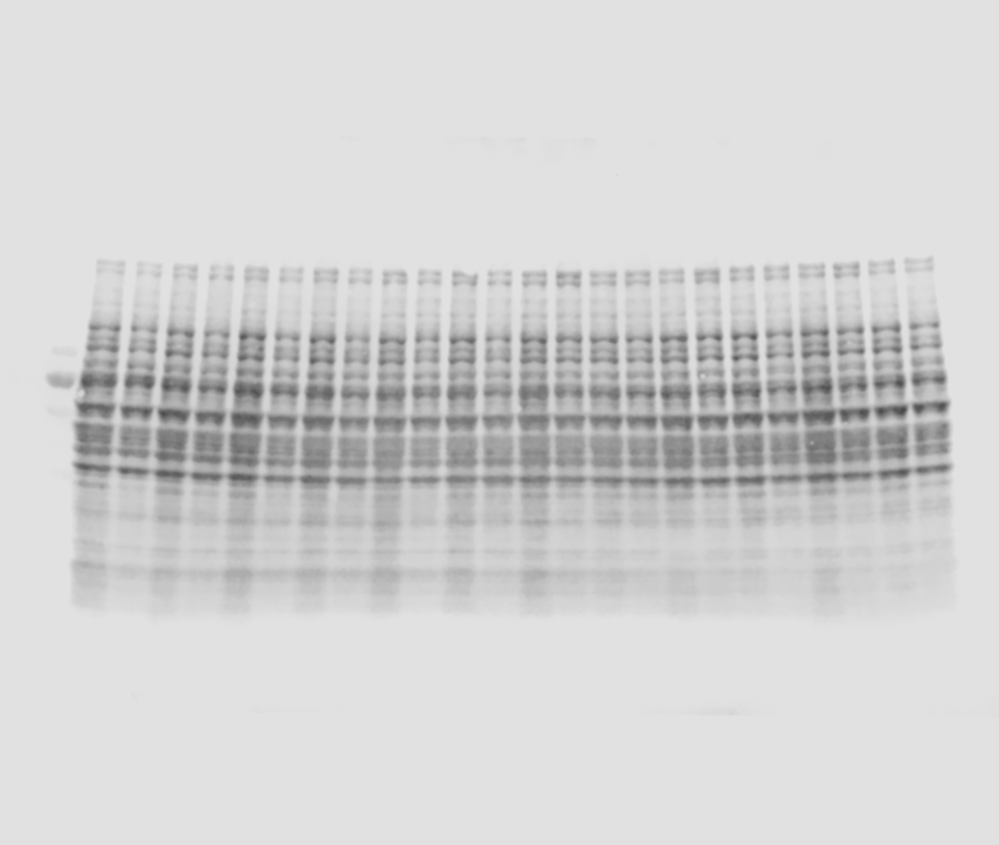

Supplement: Supplementary file 12 — Source data Fig. 4 [file 44319_2026_774_MOESM12_ESM.zip › Figure 4/4G/Total protein staining for Pho8 normalization.tif]

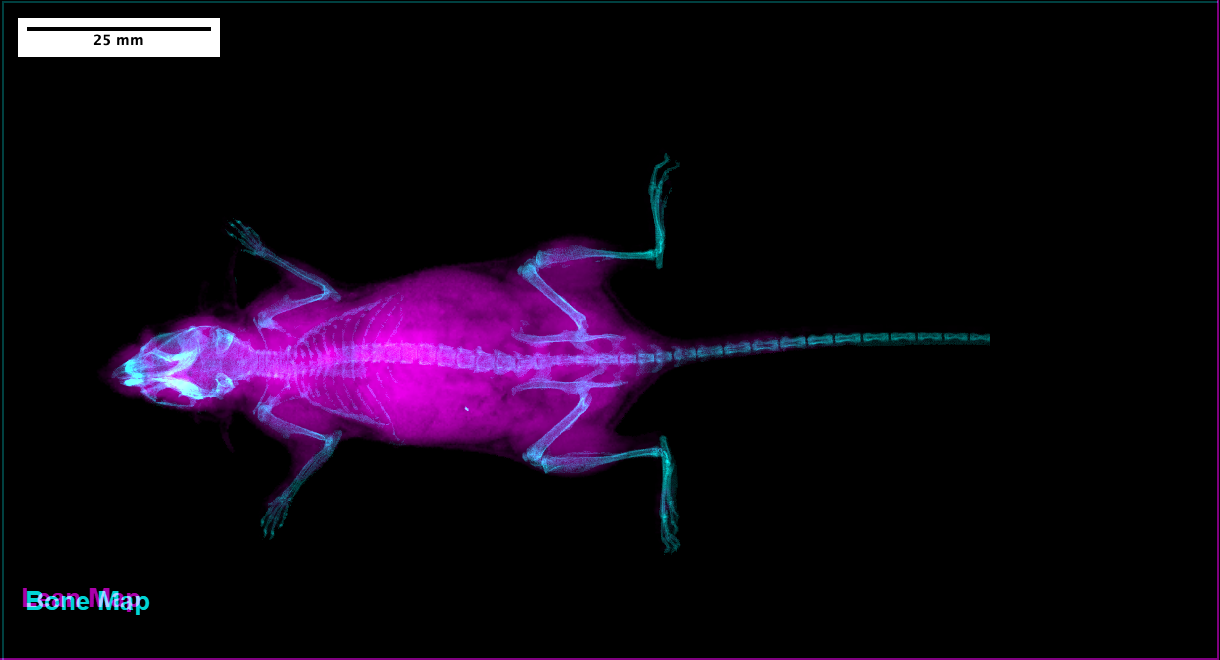

Supplement: Supplementary file 13 — Source data Fig. 5 [file 44319_2026_774_MOESM13_ESM.zip › Figure 5/5A/WT male.tiff]

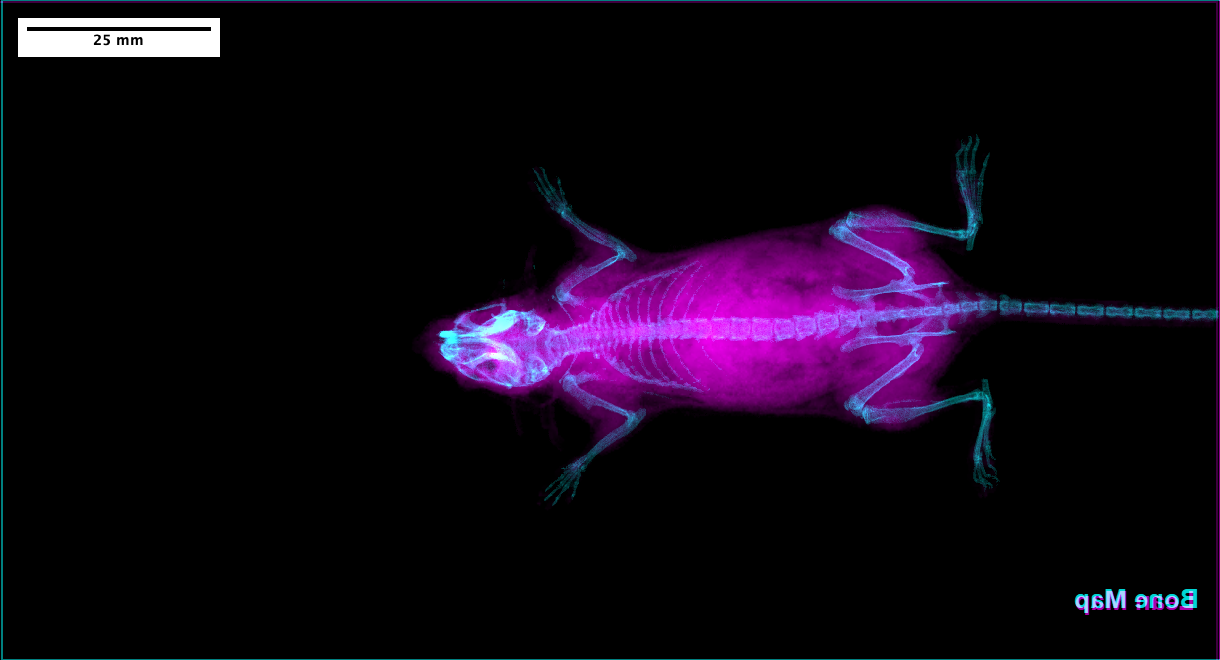

Supplement: Supplementary file 13 — Source data Fig. 5 [file 44319_2026_774_MOESM13_ESM.zip › Figure 5/5A/WT female.tiff]

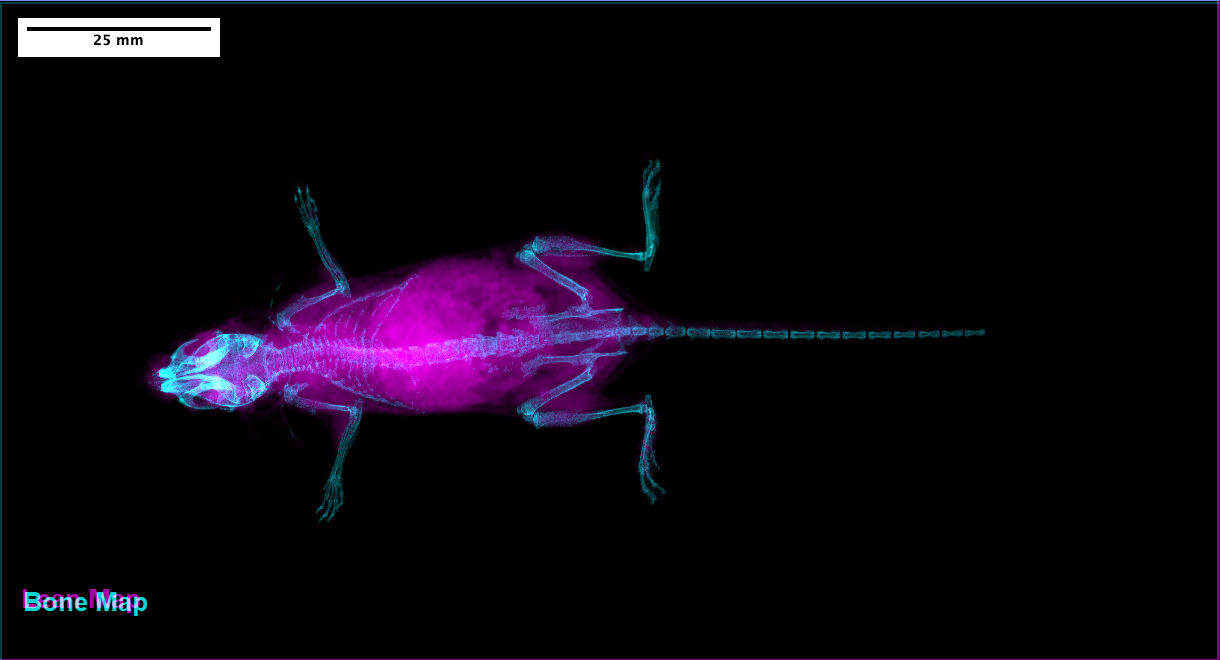

Supplement: Supplementary file 13 — Source data Fig. 5 [file 44319_2026_774_MOESM13_ESM.zip › Figure 5/5A/Tg Female.tiff]

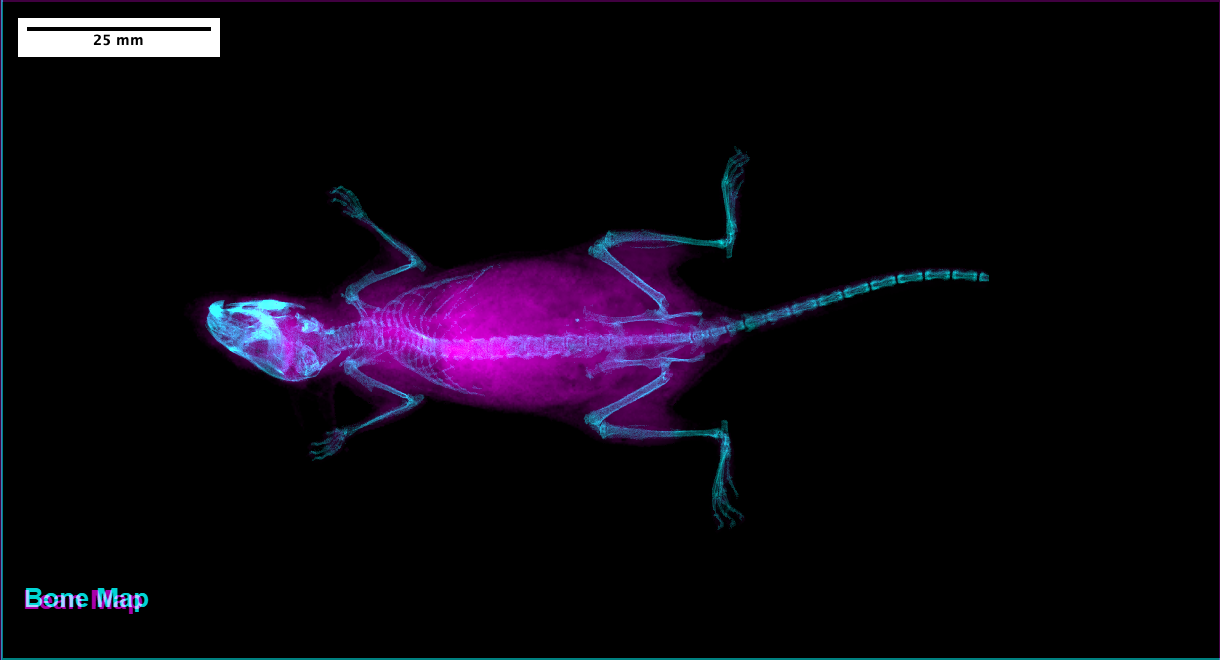

Supplement: Supplementary file 13 — Source data Fig. 5 [file 44319_2026_774_MOESM13_ESM.zip › Figure 5/5A/Tg male.tiff]

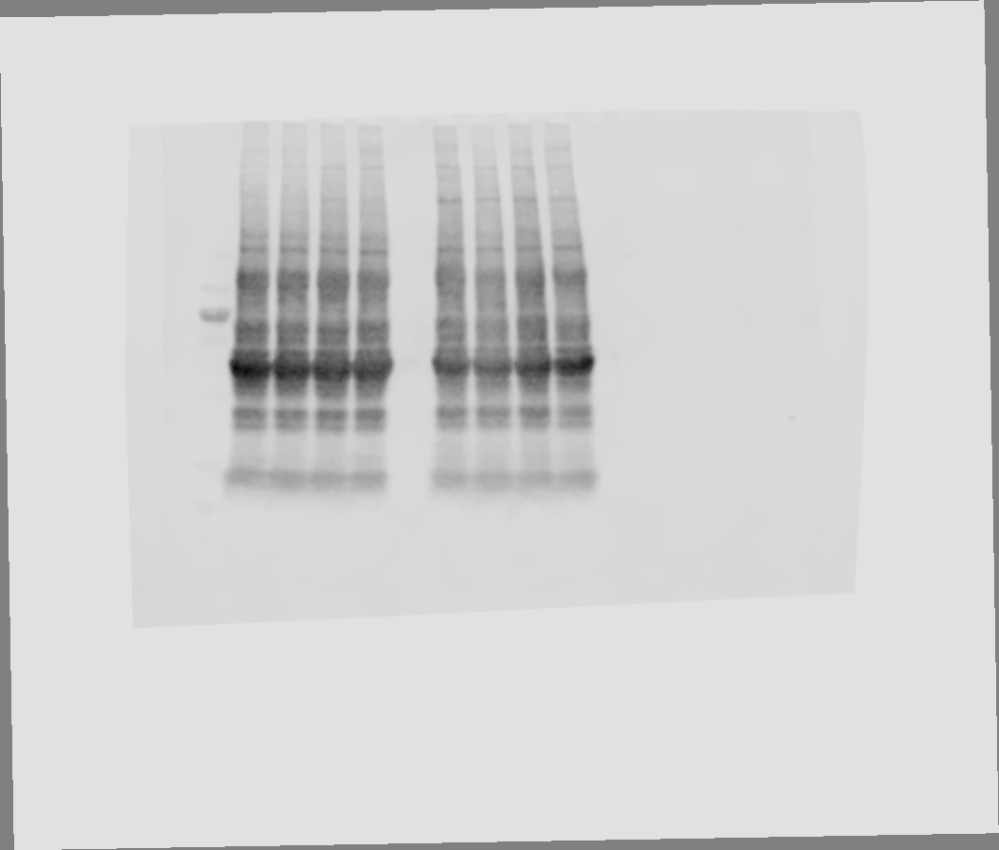

Supplement: Supplementary file 13 — Source data Fig. 5 [file 44319_2026_774_MOESM13_ESM.zip › Figure 5/5J/Total protein staining for HSPB7 normalization.tif]

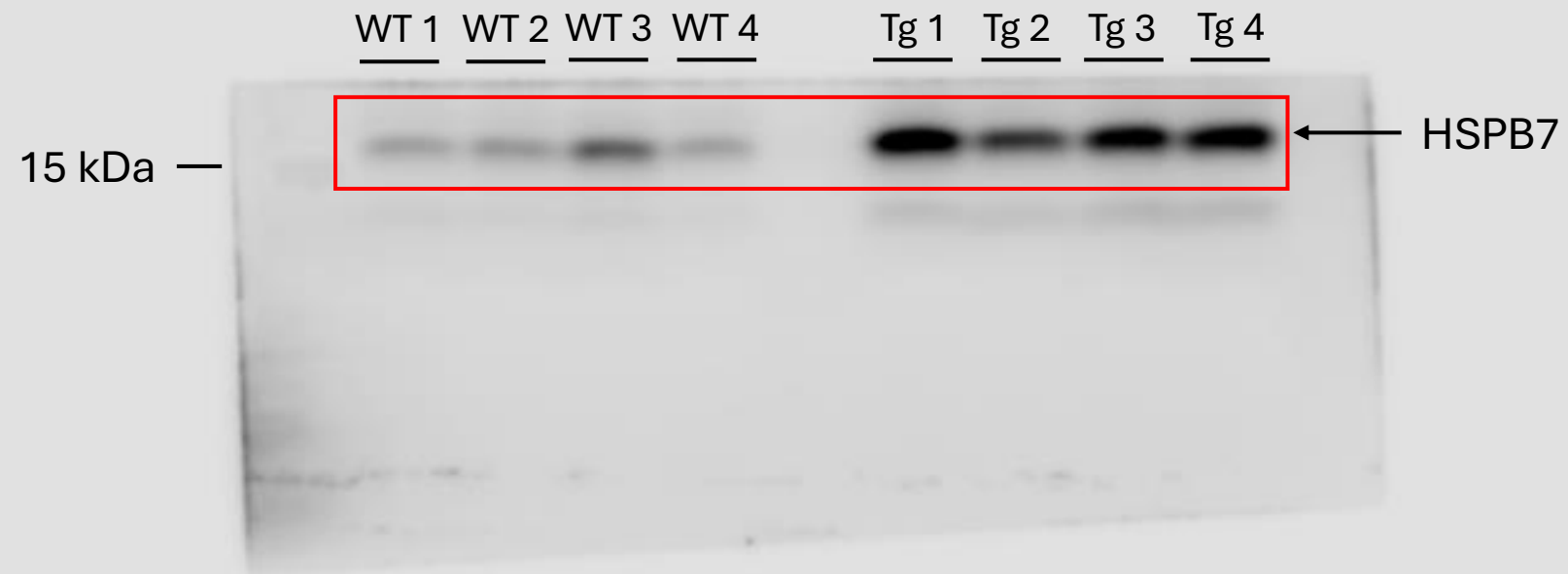

Supplement: Supplementary file 13 — Source data Fig. 5 [file 44319_2026_774_MOESM13_ESM.zip › Figure 5/5J/HSPB7 Western blot.pdf]

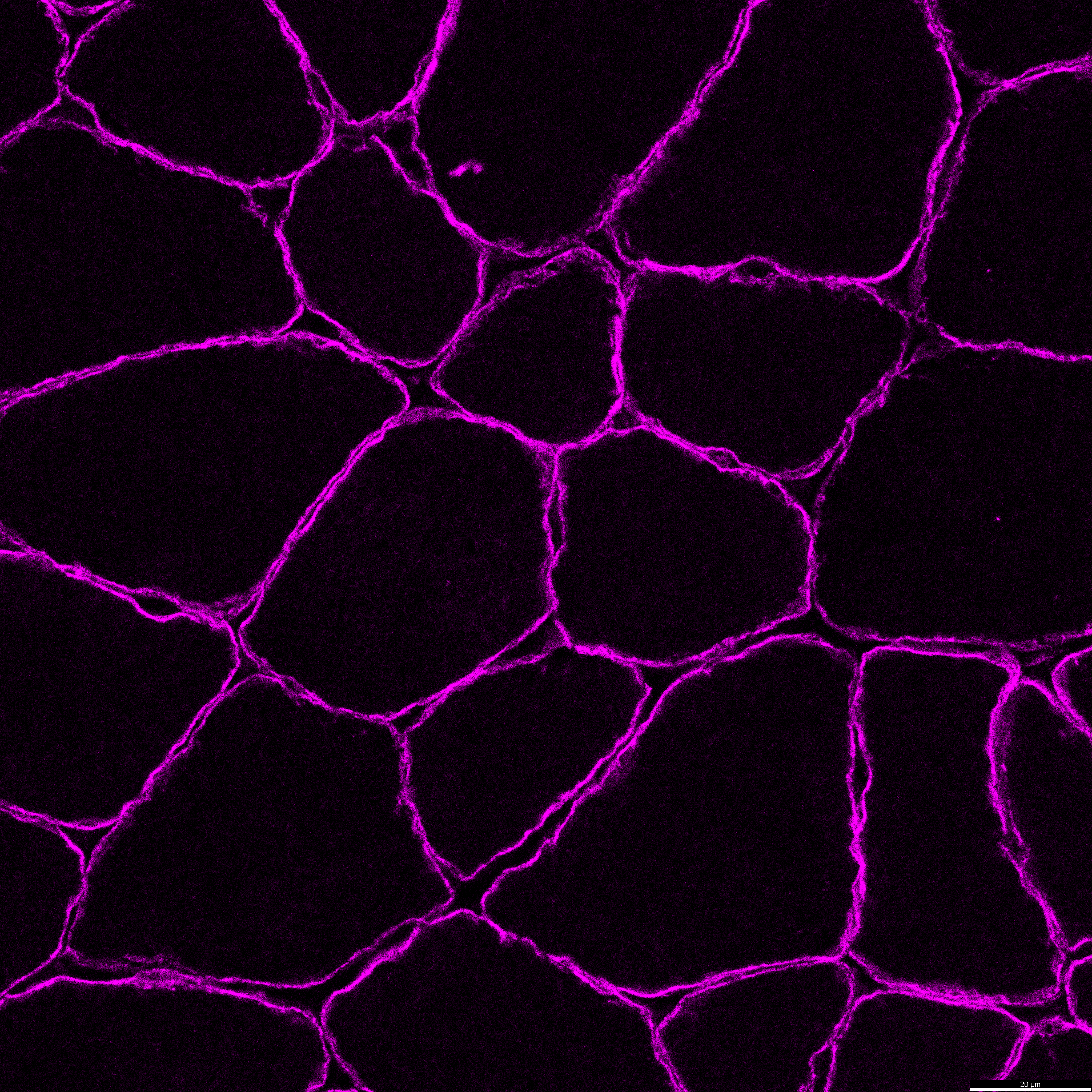

Supplement: Supplementary file 13 — Source data Fig. 5 [file 44319_2026_774_MOESM13_ESM.zip › Figure 5/5C/Wildtype dystrophin.tif]

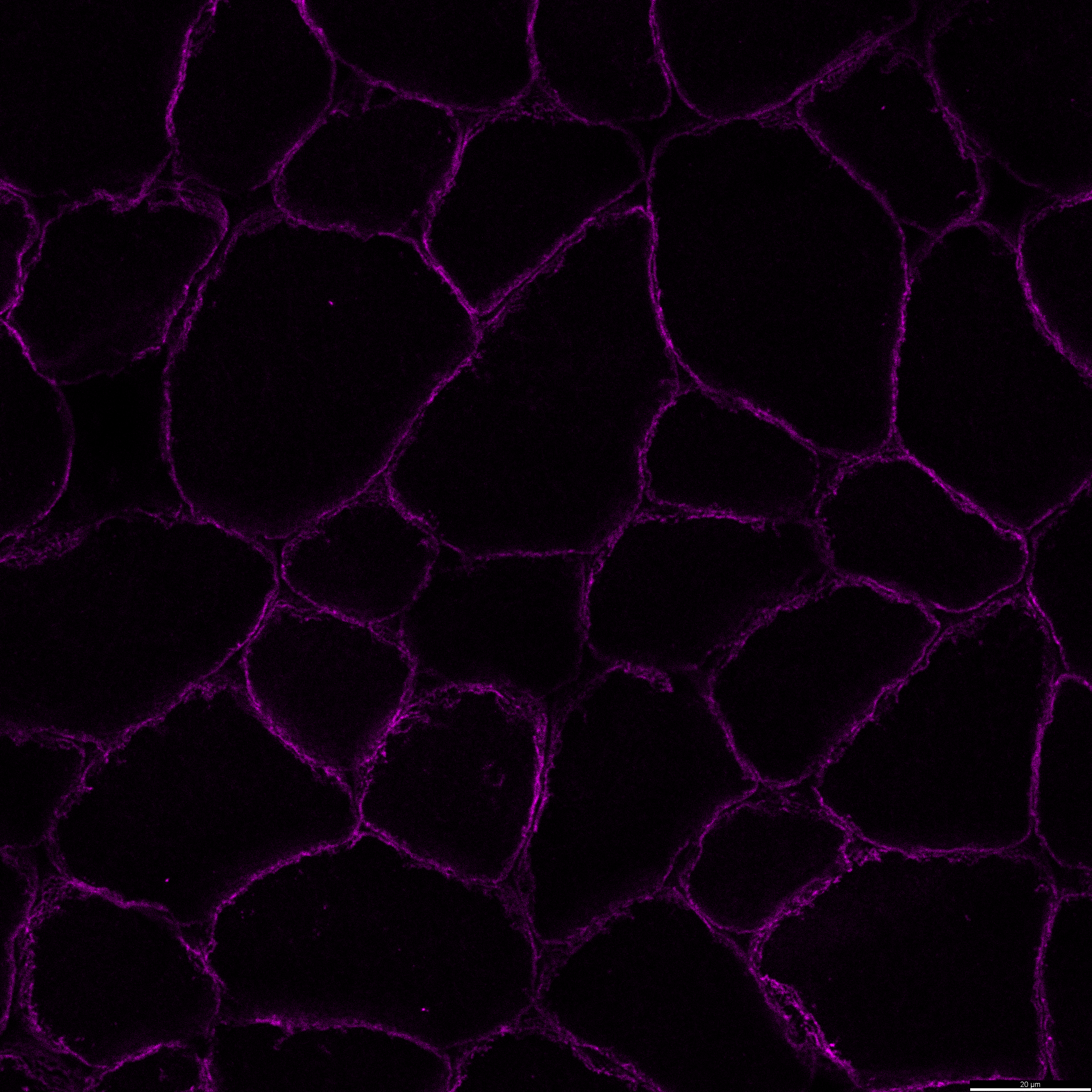

Supplement: Supplementary file 13 — Source data Fig. 5 [file 44319_2026_774_MOESM13_ESM.zip › Figure 5/5C/Transgenic dystrophin.tif]

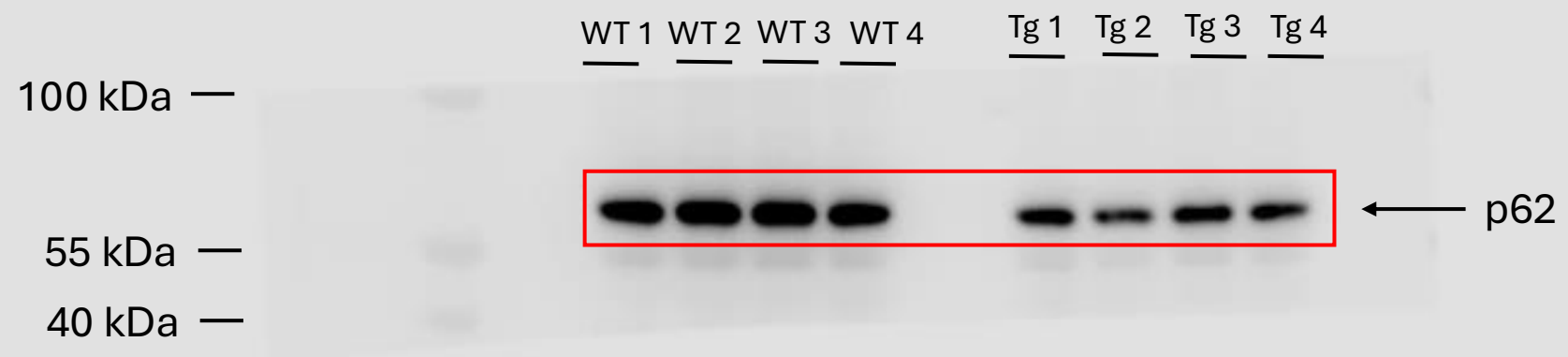

Supplement: Supplementary file 13 — Source data Fig. 5 [file 44319_2026_774_MOESM13_ESM.zip › Figure 5/5L/p62 Western blot.pdf]

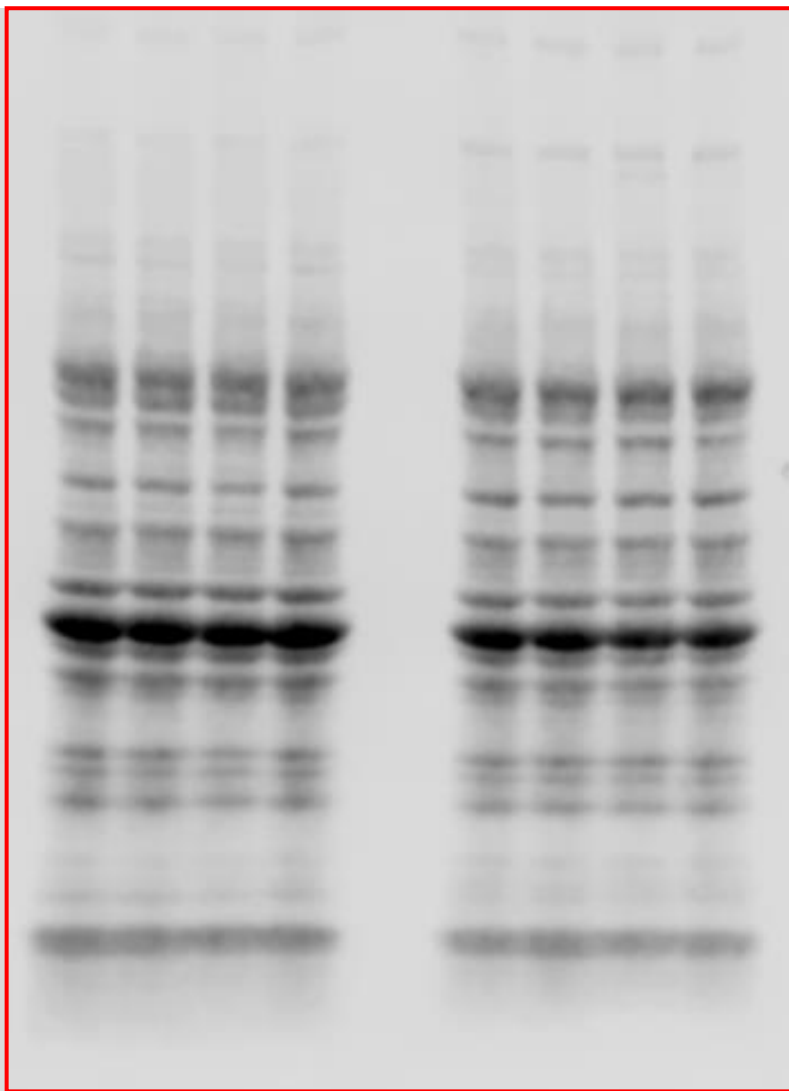

↑  
Used for p62 normalization

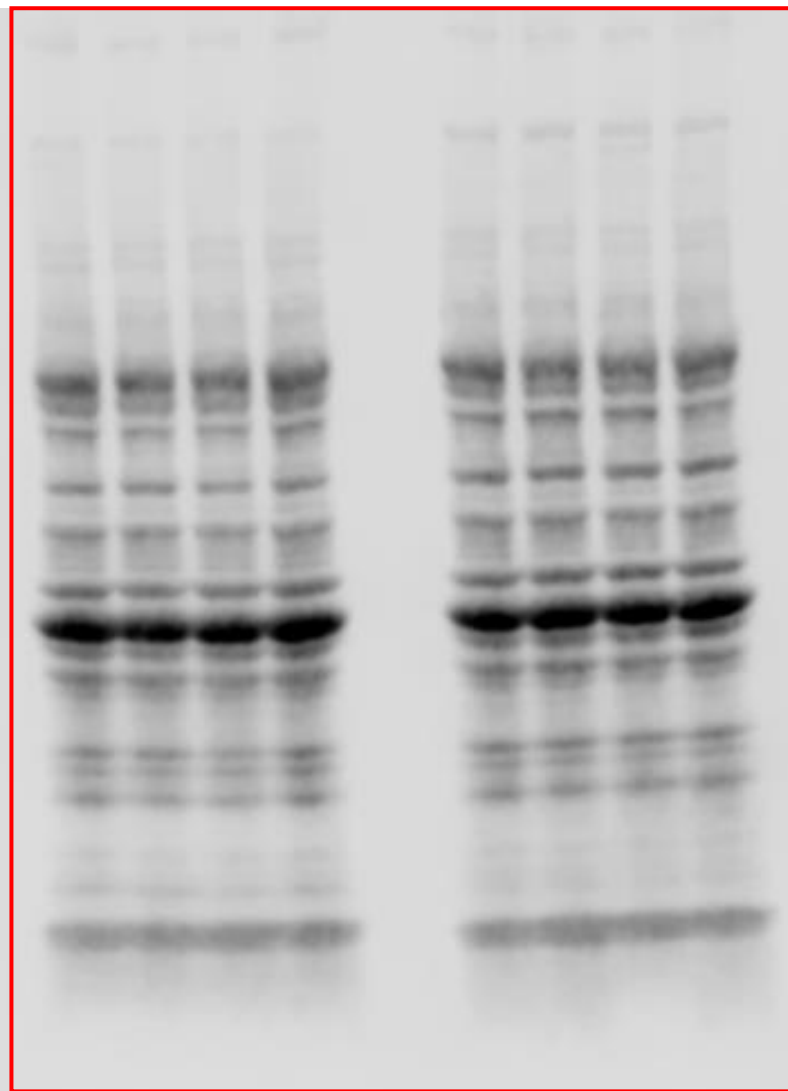

↑  
Used for Phos-p62 normalization

Supplement: Supplementary file 13 — Source data Fig. 5 [file 44319_2026_774_MOESM13_ESM.zip › Figure 5/5L/Total protein staining for p62 and phos-p62 normalization.pdf]

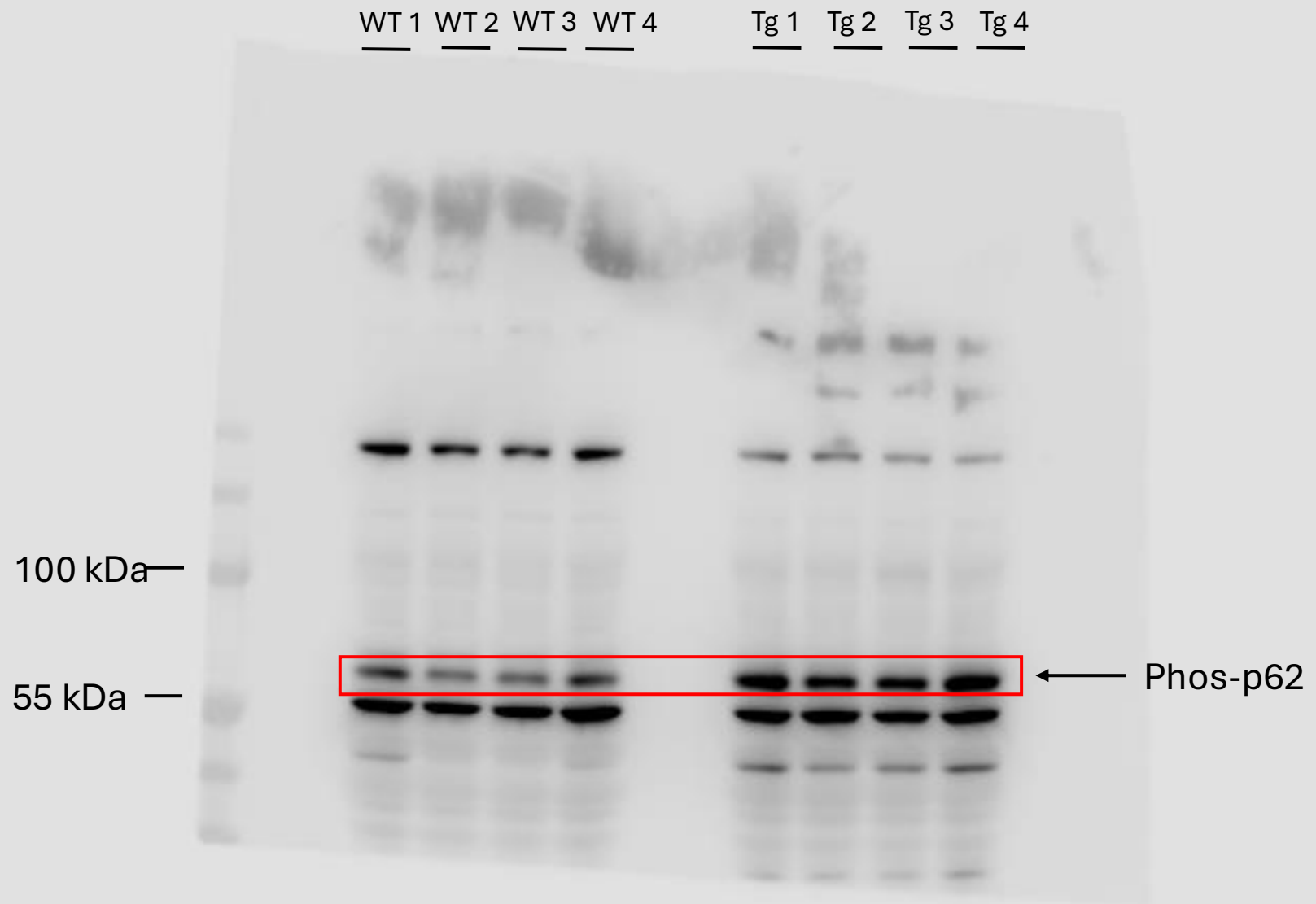

Supplement: Supplementary file 13 — Source data Fig. 5 [file 44319_2026_774_MOESM13_ESM.zip › Figure 5/5L/Phos-p62 western blot.pdf]

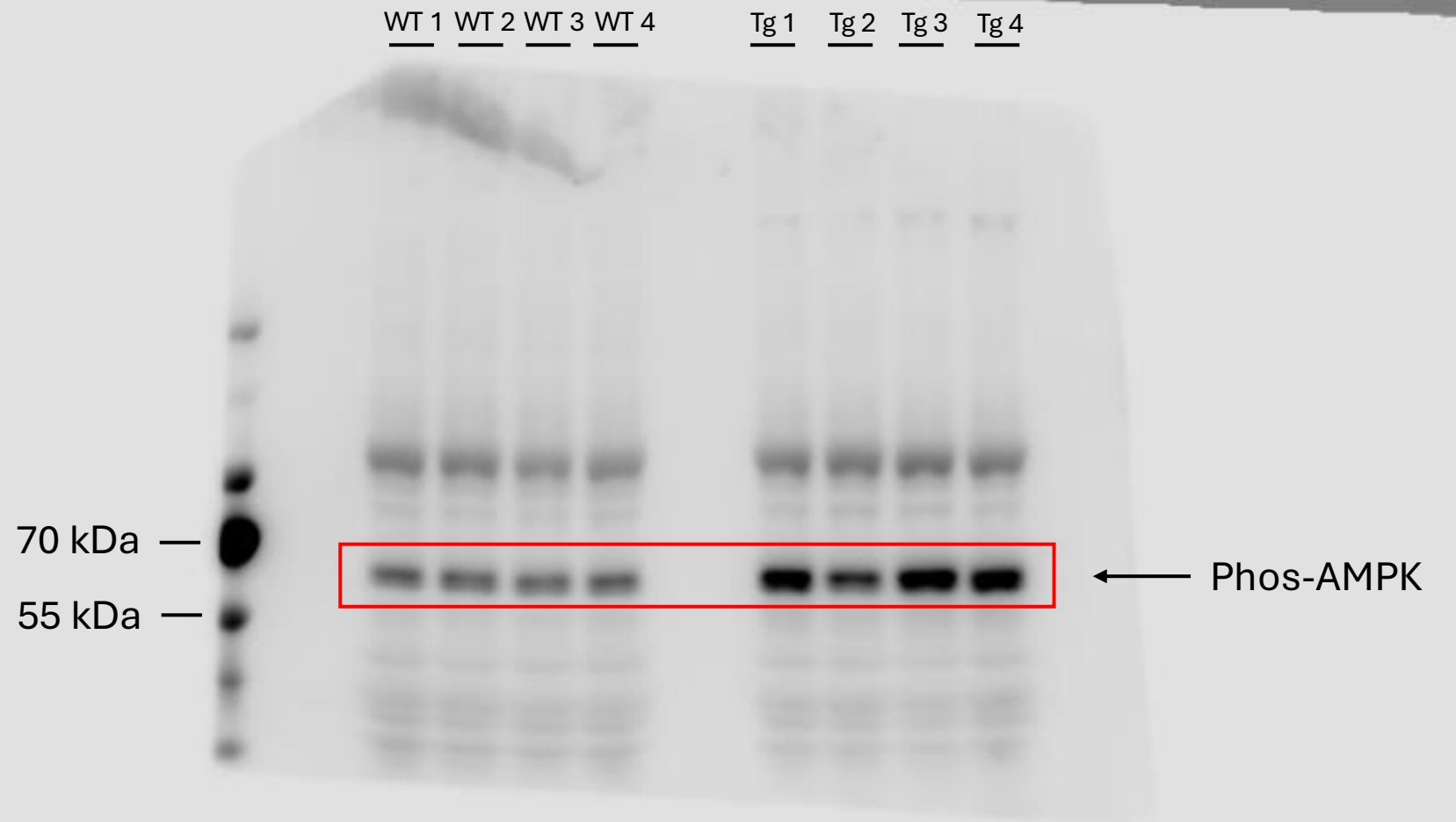

Supplement: Supplementary file 14 — Source data Fig. 6 [file 44319_2026_774_MOESM14_ESM.zip › Figure 6/6F/Phos-AMPK western blot.pdf]

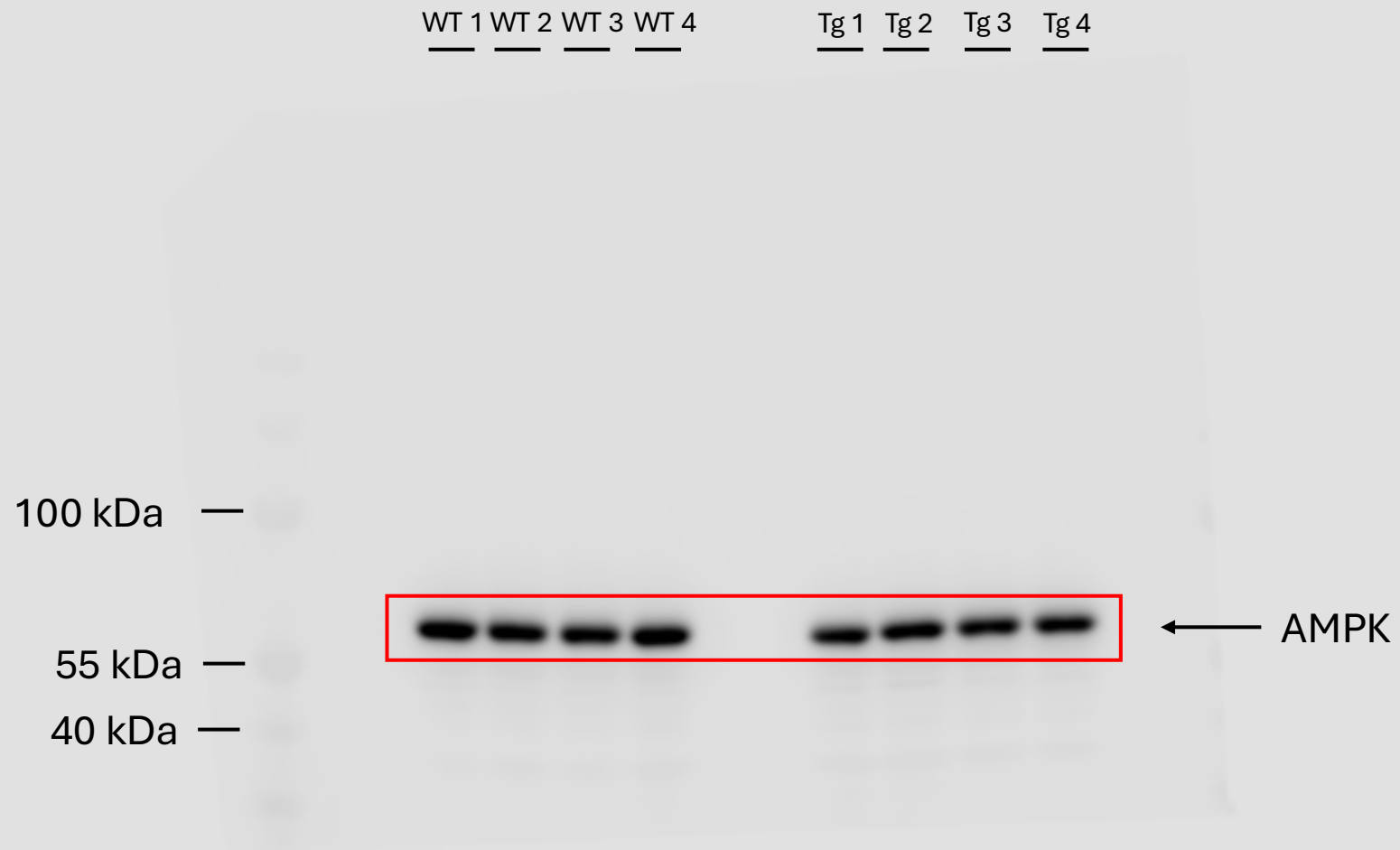

Supplement: Supplementary file 14 — Source data Fig. 6 [file 44319_2026_774_MOESM14_ESM.zip › Figure 6/6F/AMPK Western blot.pdf]

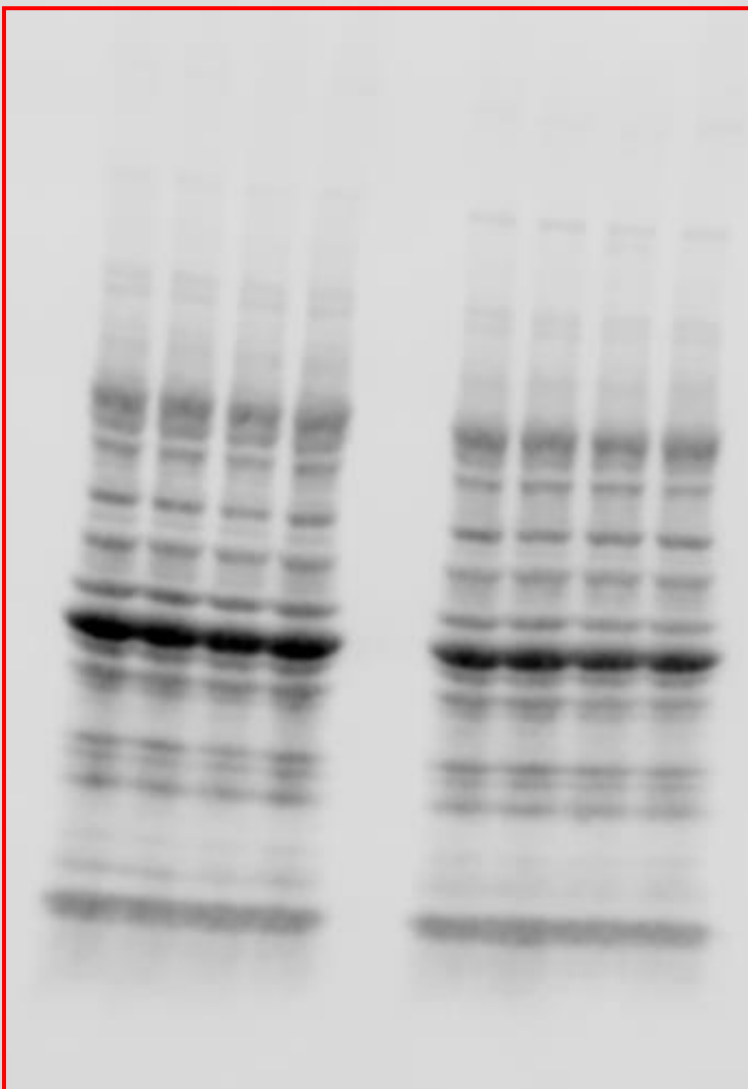

↑  
Used for AMPK normalization

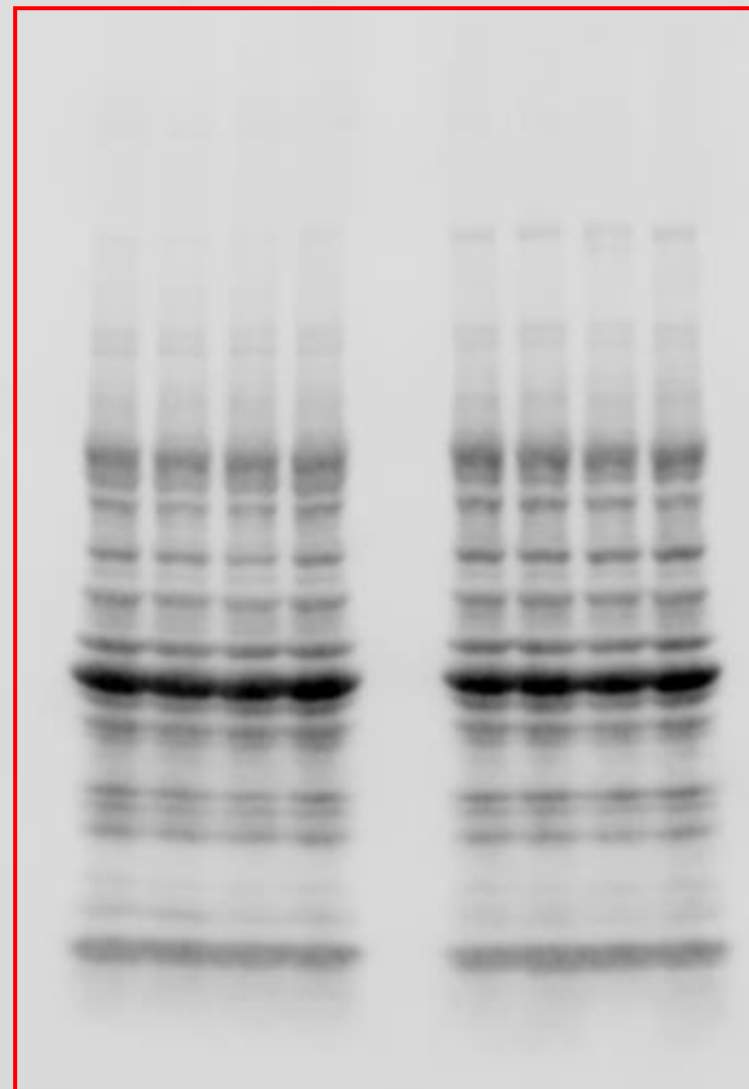

↑  
Used for Phos-AMPK normalization

Supplement: Supplementary file 14 — Source data Fig. 6 [file 44319_2026_774_MOESM14_ESM.zip › Figure 6/6F/TPS for AMPK and Phos-AMPK normalization.pdf]

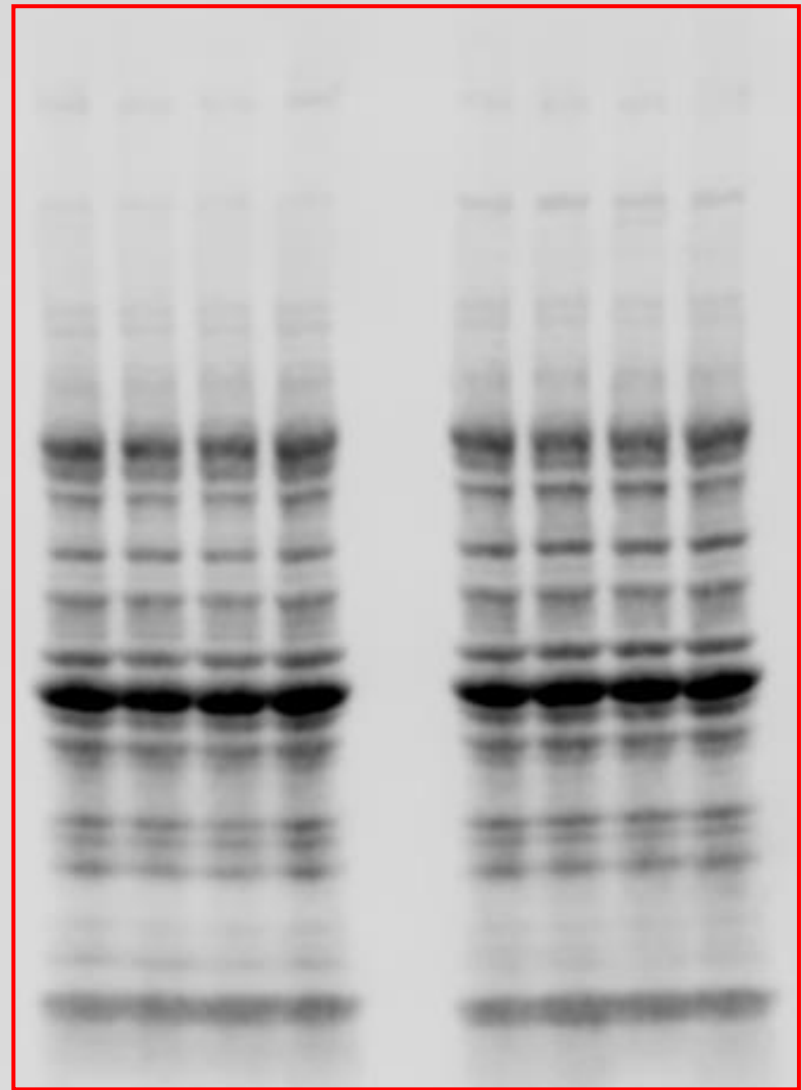

↑  
Used for LC3-II normalization

Supplement: Supplementary file 14 — Source data Fig. 6 [file 44319_2026_774_MOESM14_ESM.zip › Figure 6/6F/Total protein staining for LC3 normalization.pdf]

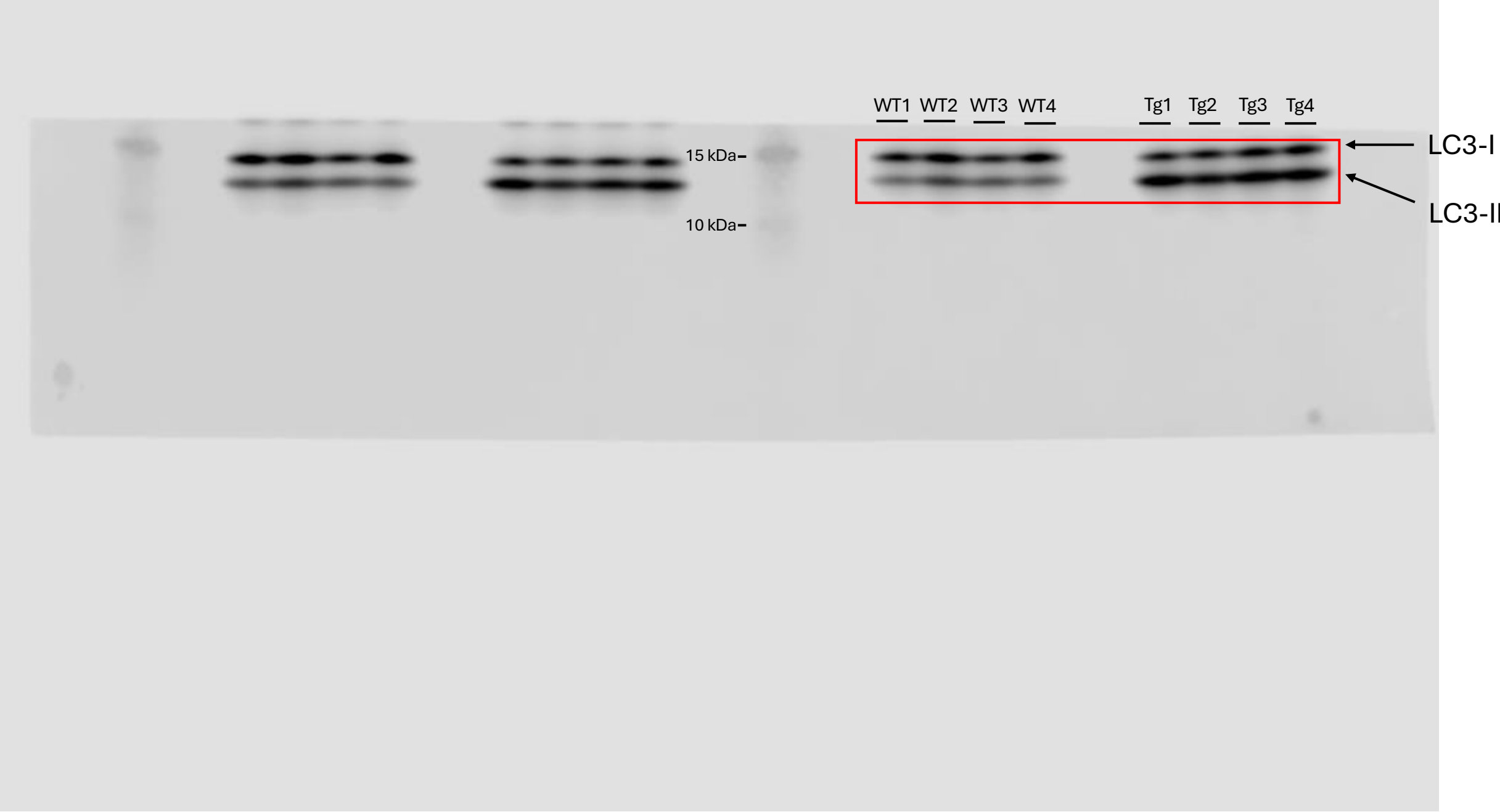

Supplement: Supplementary file 14 — Source data Fig. 6 [file 44319_2026_774_MOESM14_ESM.zip › Figure 6/6F/LC3 Western blot.pdf]

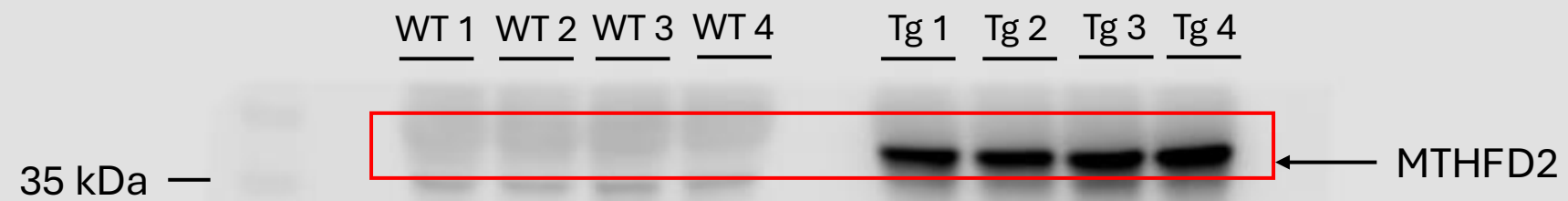

Supplement: Supplementary file 14 — Source data Fig. 6 [file 44319_2026_774_MOESM14_ESM.zip › Figure 6/6B/MTHFD2 western blot.pdf]

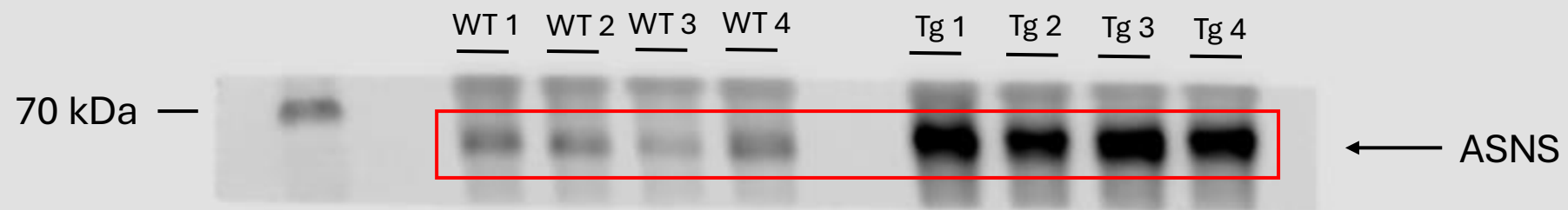

Supplement: Supplementary file 14 — Source data Fig. 6 [file 44319_2026_774_MOESM14_ESM.zip › Figure 6/6B/ASNS Western blot.pdf]

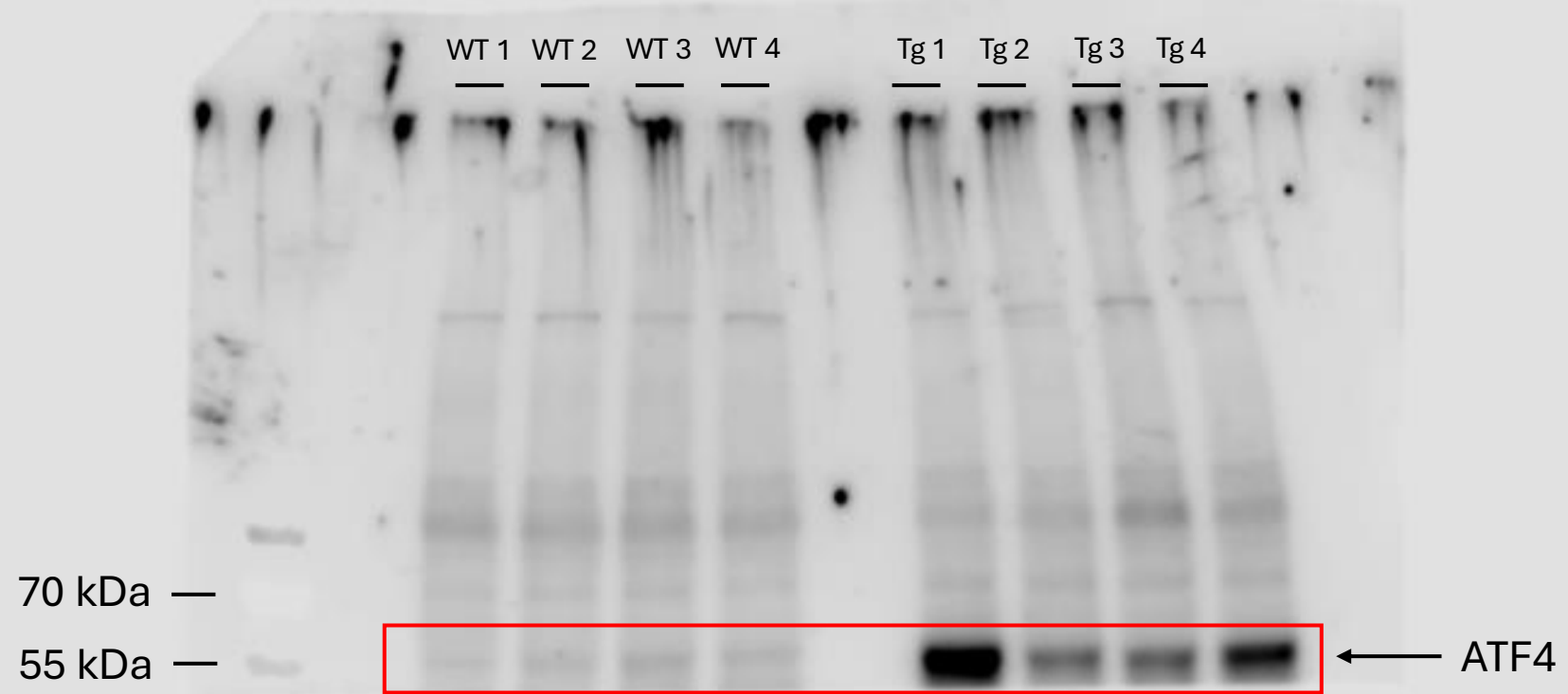

Supplement: Supplementary file 14 — Source data Fig. 6 [file 44319_2026_774_MOESM14_ESM.zip › Figure 6/6B/ATF4 western blot.pdf]

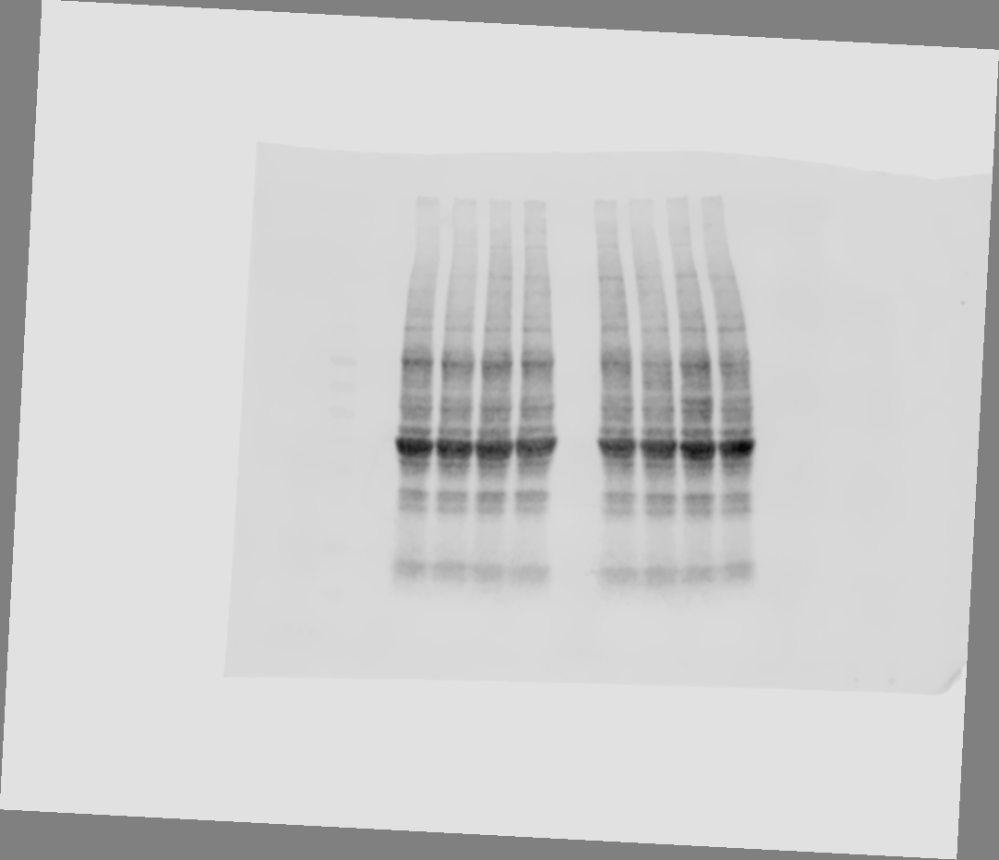

Supplement: Supplementary file 14 — Source data Fig. 6 [file 44319_2026_774_MOESM14_ESM.zip › Figure 6/6B/Total protein staining for ATF4 and MTHFD2 normalization.tif]

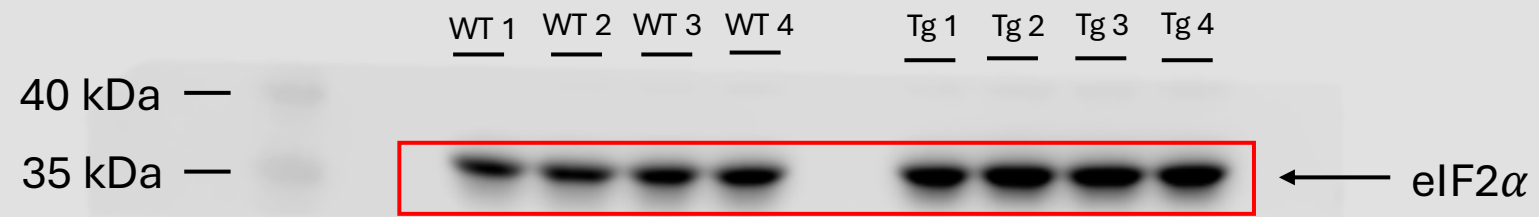

Supplement: Supplementary file 14 — Source data Fig. 6 [file 44319_2026_774_MOESM14_ESM.zip › Figure 6/6B/eIF2a western blot.pdf]

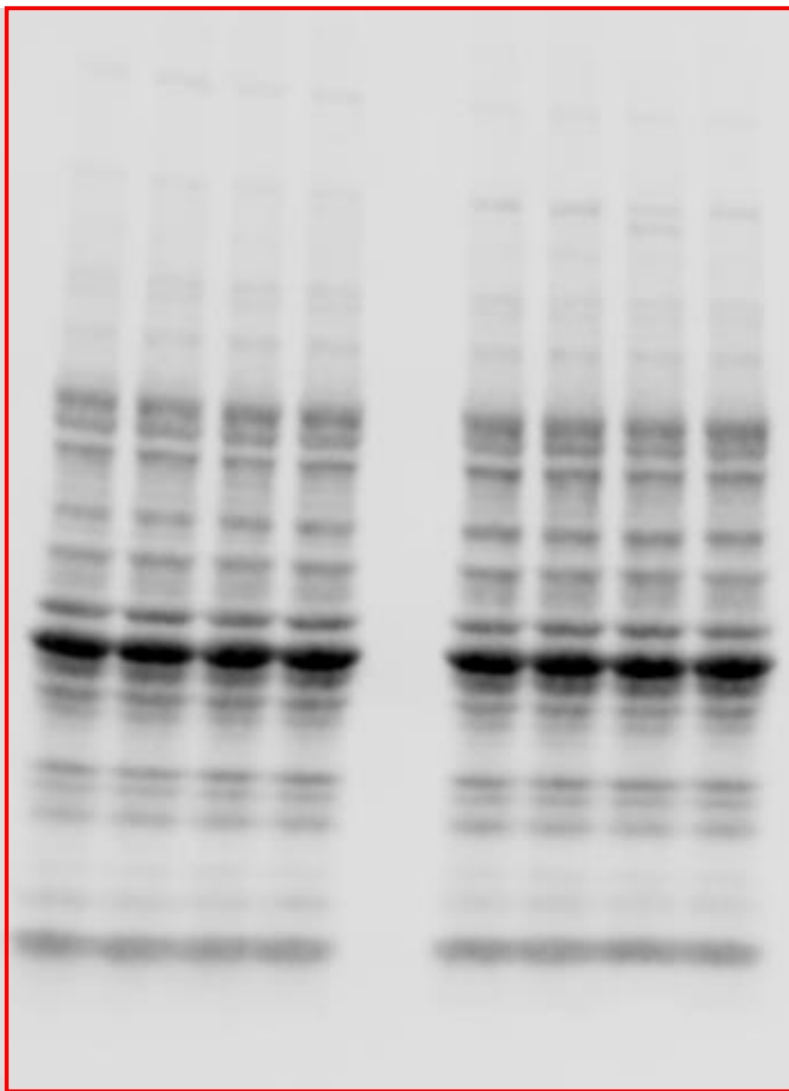

↑  
Used for eIF2 $\alpha$  normalization

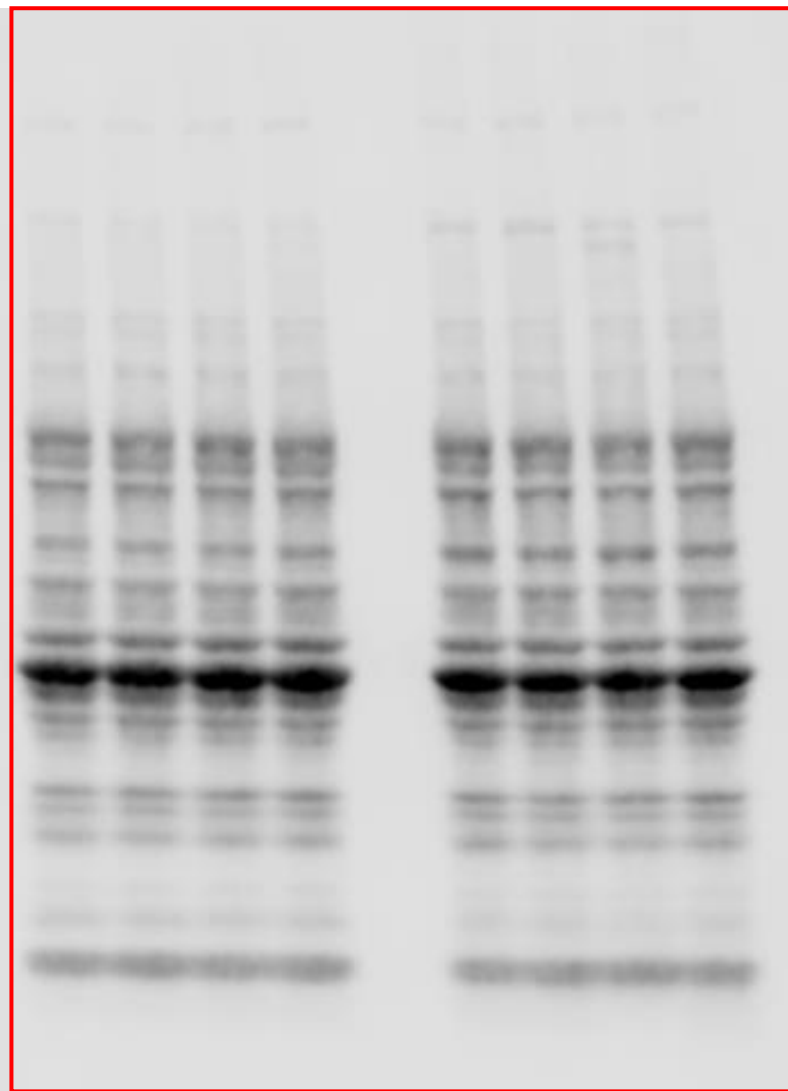

↑  
Used for Phos- eIF2 $\alpha$  normalization

Supplement: Supplementary file 14 — Source data Fig. 6 [file 44319_2026_774_MOESM14_ESM.zip › Figure 6/6B/TPS for eIF2Γì║ and phos-eIF2Γì║ normalization.pdf]

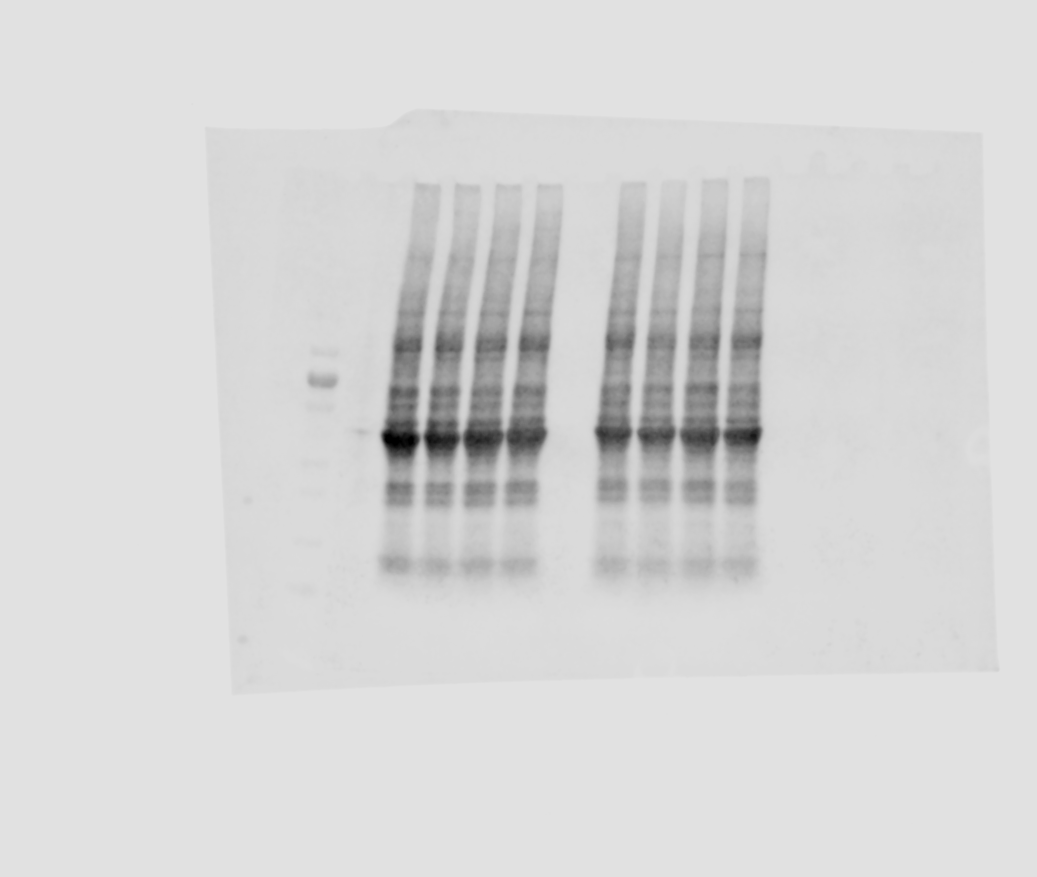

Supplement: Supplementary file 14 — Source data Fig. 6 [file 44319_2026_774_MOESM14_ESM.zip › Figure 6/6B/Total protein staining for ASNS normalization.tif]

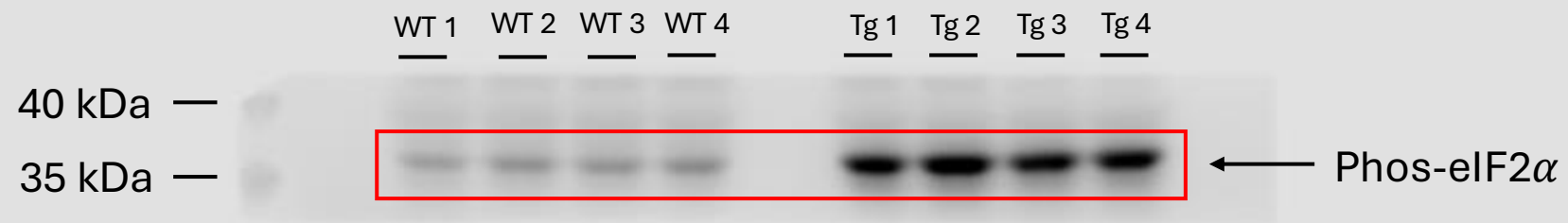

Supplement: Supplementary file 14 — Source data Fig. 6 [file 44319_2026_774_MOESM14_ESM.zip › Figure 6/6B/Phos-eIF2a western blot.pdf]

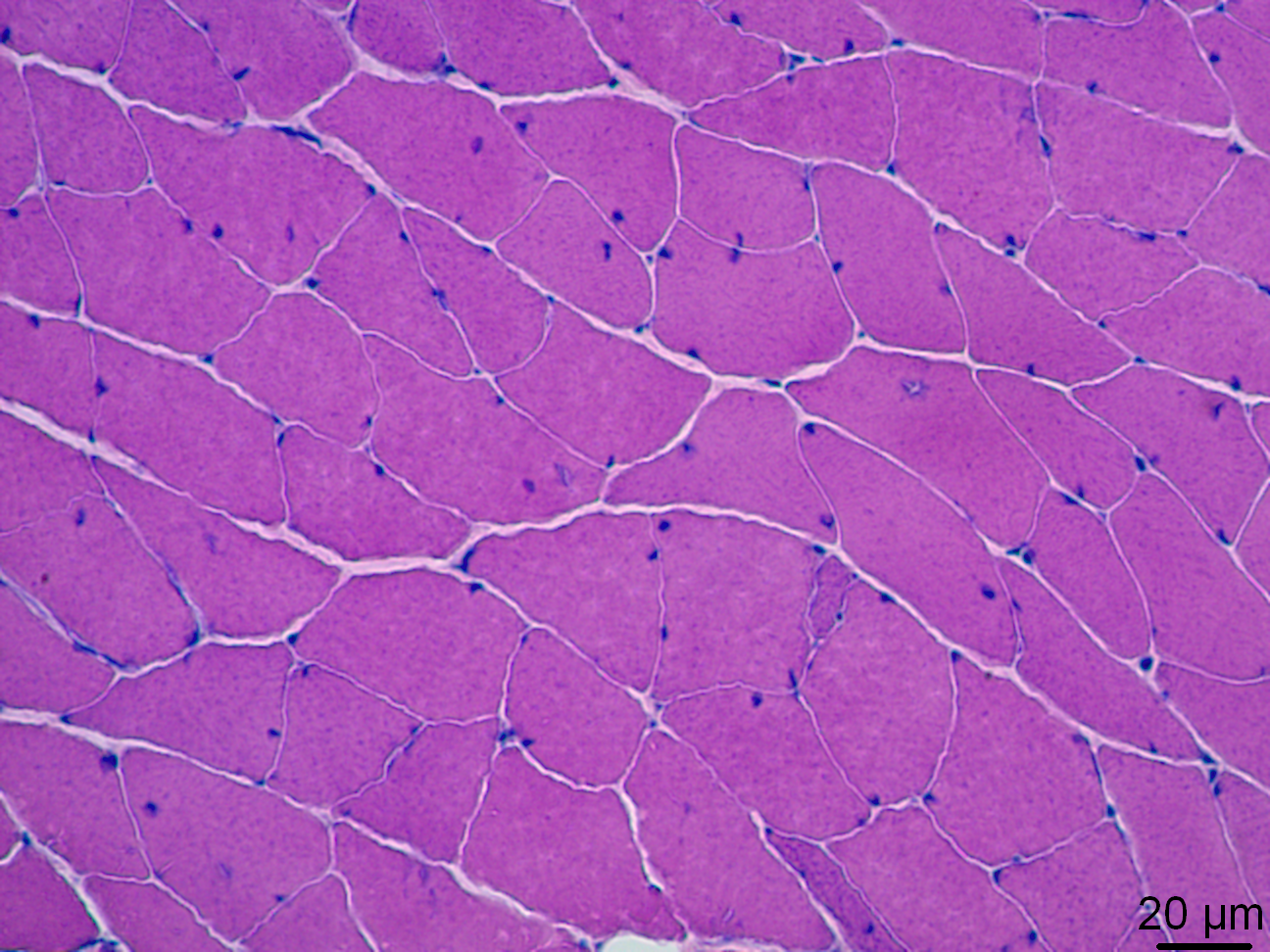

Supplement: Supplementary file 15 — Source data Fig. 7 [file 44319_2026_774_MOESM15_ESM.zip › Figure 7/7E/Wildtype 27-month-old quadricep H&E.tif]

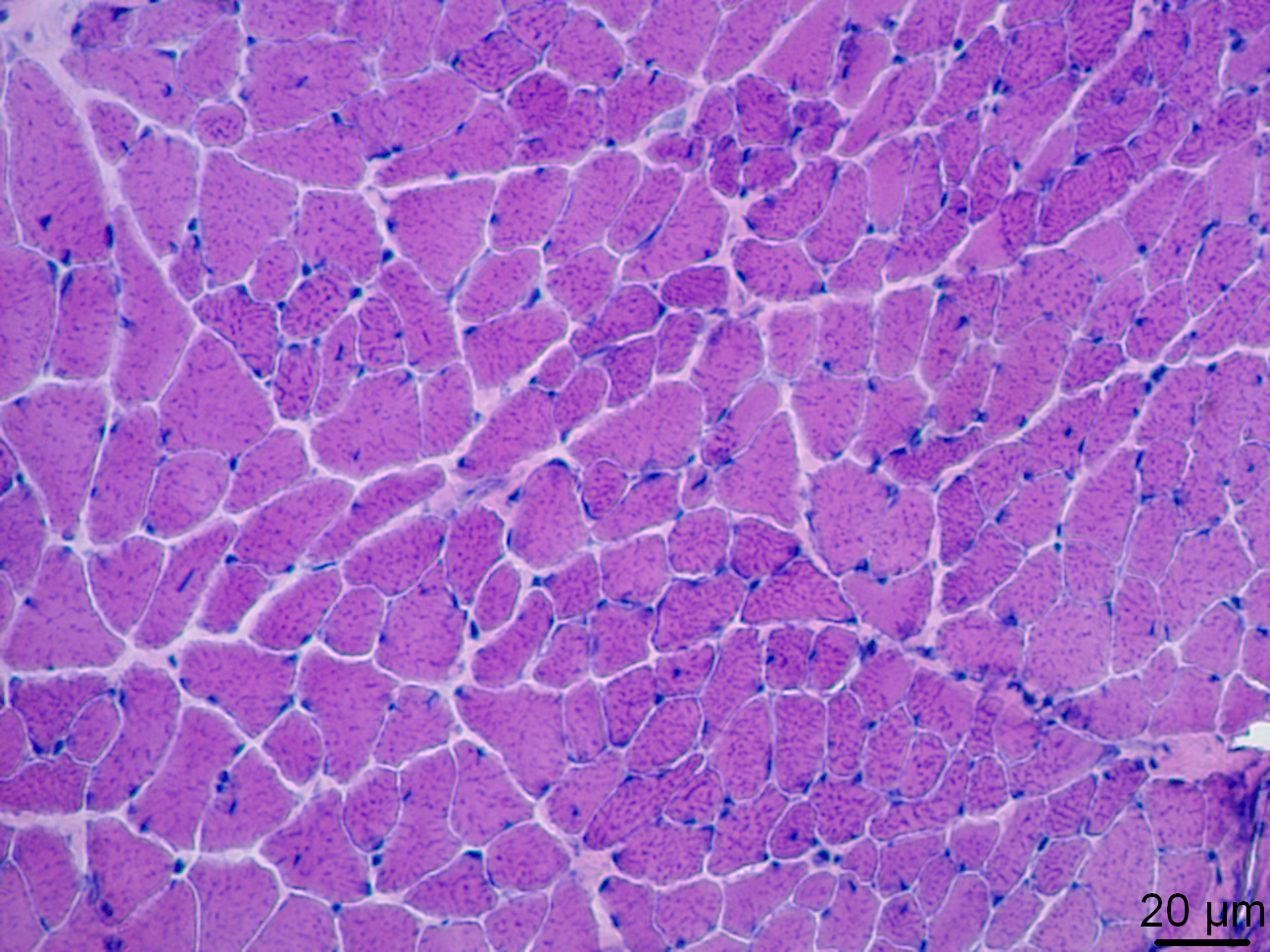

Supplement: Supplementary file 15 — Source data Fig. 7 [file 44319_2026_774_MOESM15_ESM.zip › Figure 7/7E/Transgenic 27-month-old quadricep H&E.tif]

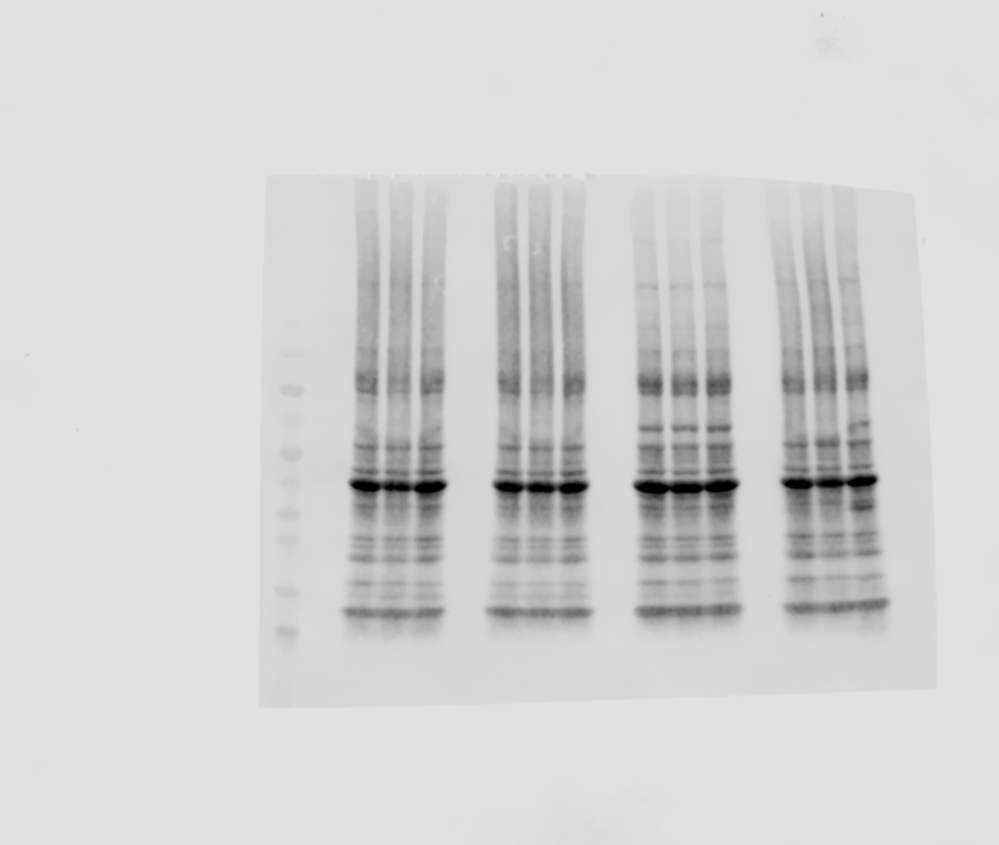

Supplement: Supplementary file 15 — Source data Fig. 7 [file 44319_2026_774_MOESM15_ESM.zip › Figure 7/7L/Total protein staining for LC3-II normalization.tif]

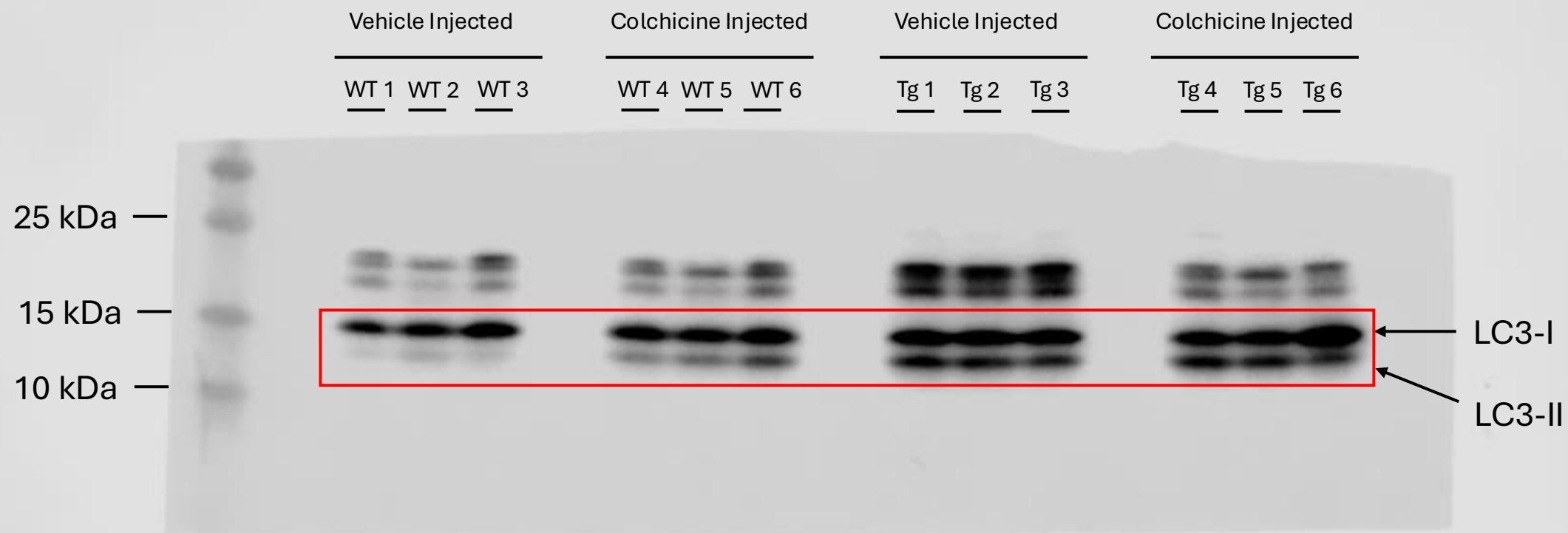

Supplement: Supplementary file 15 — Source data Fig. 7 [file 44319_2026_774_MOESM15_ESM.zip › Figure 7/7L/LC3 Western blot young colchicine mice.pdf]

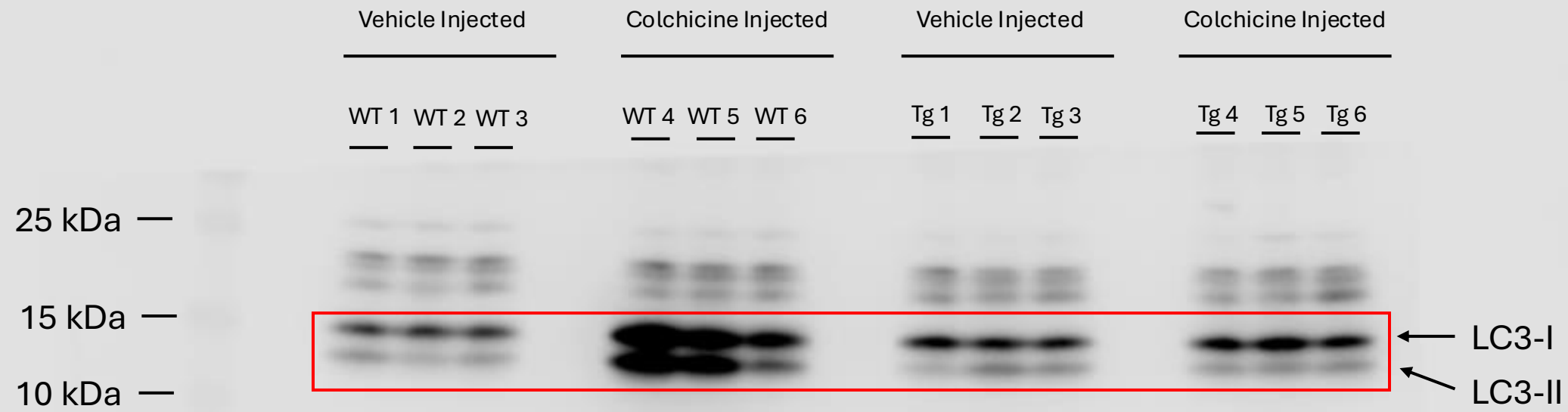

Supplement: Supplementary file 15 — Source data Fig. 7 [file 44319_2026_774_MOESM15_ESM.zip › Figure 7/7N/LC3 Western blot aged colchicine mice.pdf]

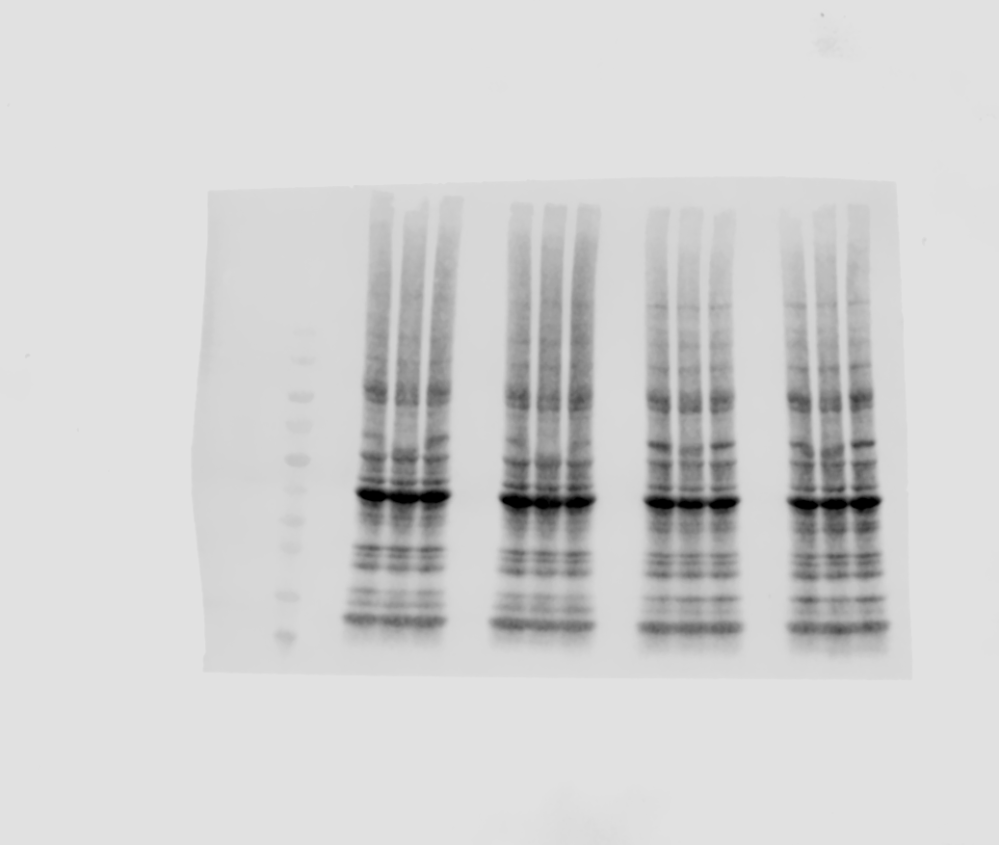

Supplement: Supplementary file 15 — Source data Fig. 7 [file 44319_2026_774_MOESM15_ESM.zip › Figure 7/7N/Total protein staining for LC3 normalization aged colchicine mice.tif]

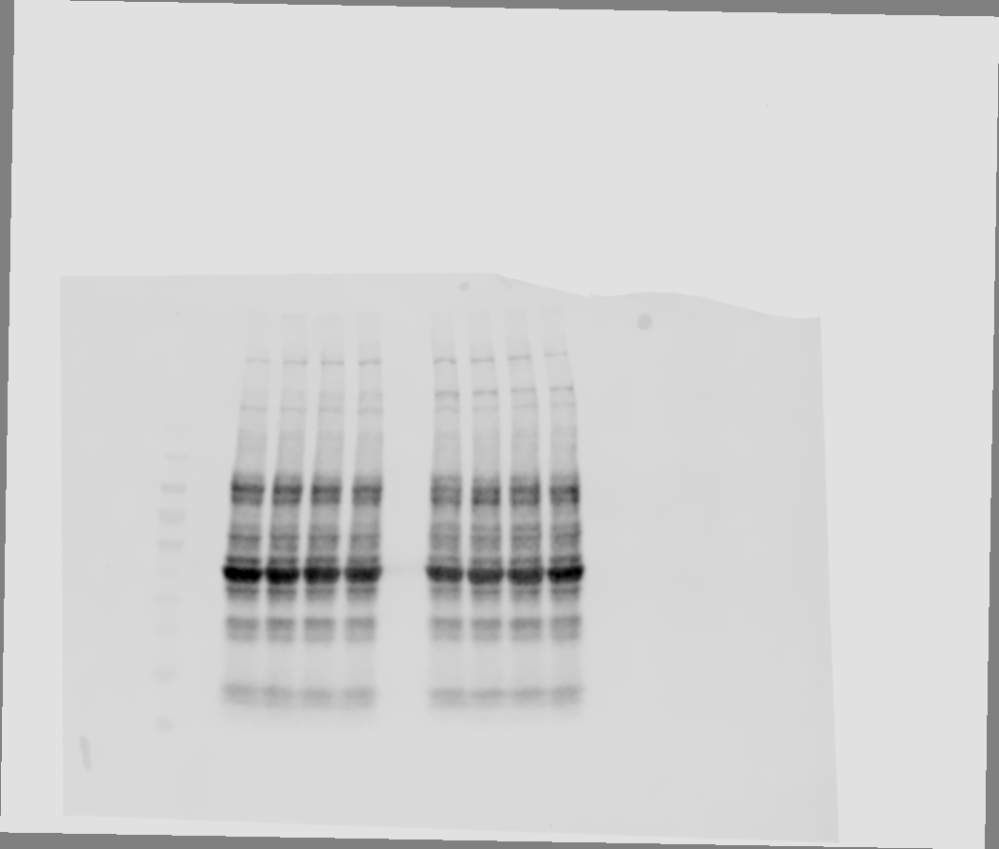

Supplement: Supplementary file 15 — Source data Fig. 7 [file 44319_2026_774_MOESM15_ESM.zip › Figure 7/7I/Total protein staining for SPG20 normalization.tif]

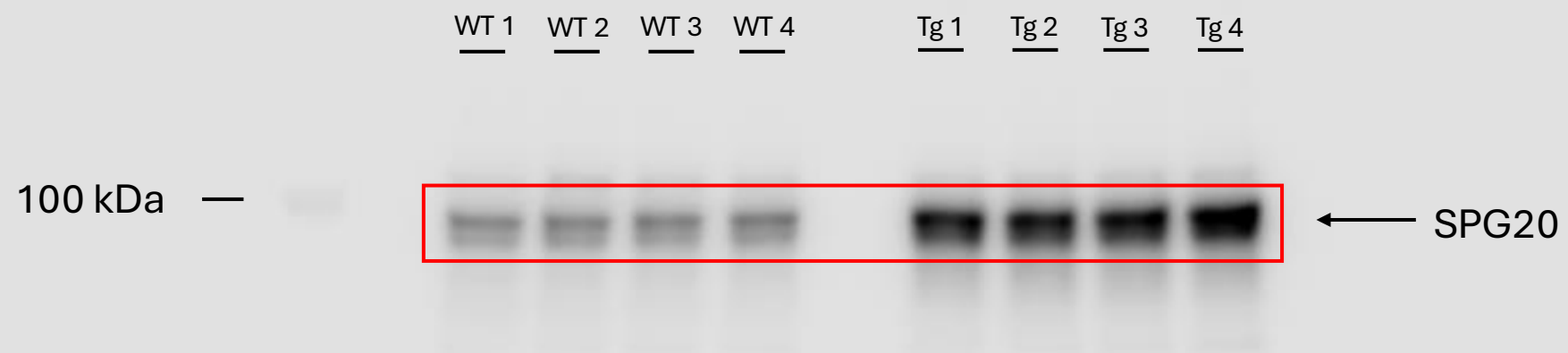

Supplement: Supplementary file 15 — Source data Fig. 7 [file 44319_2026_774_MOESM15_ESM.zip › Figure 7/7I/SPG20 Western blot.pdf]

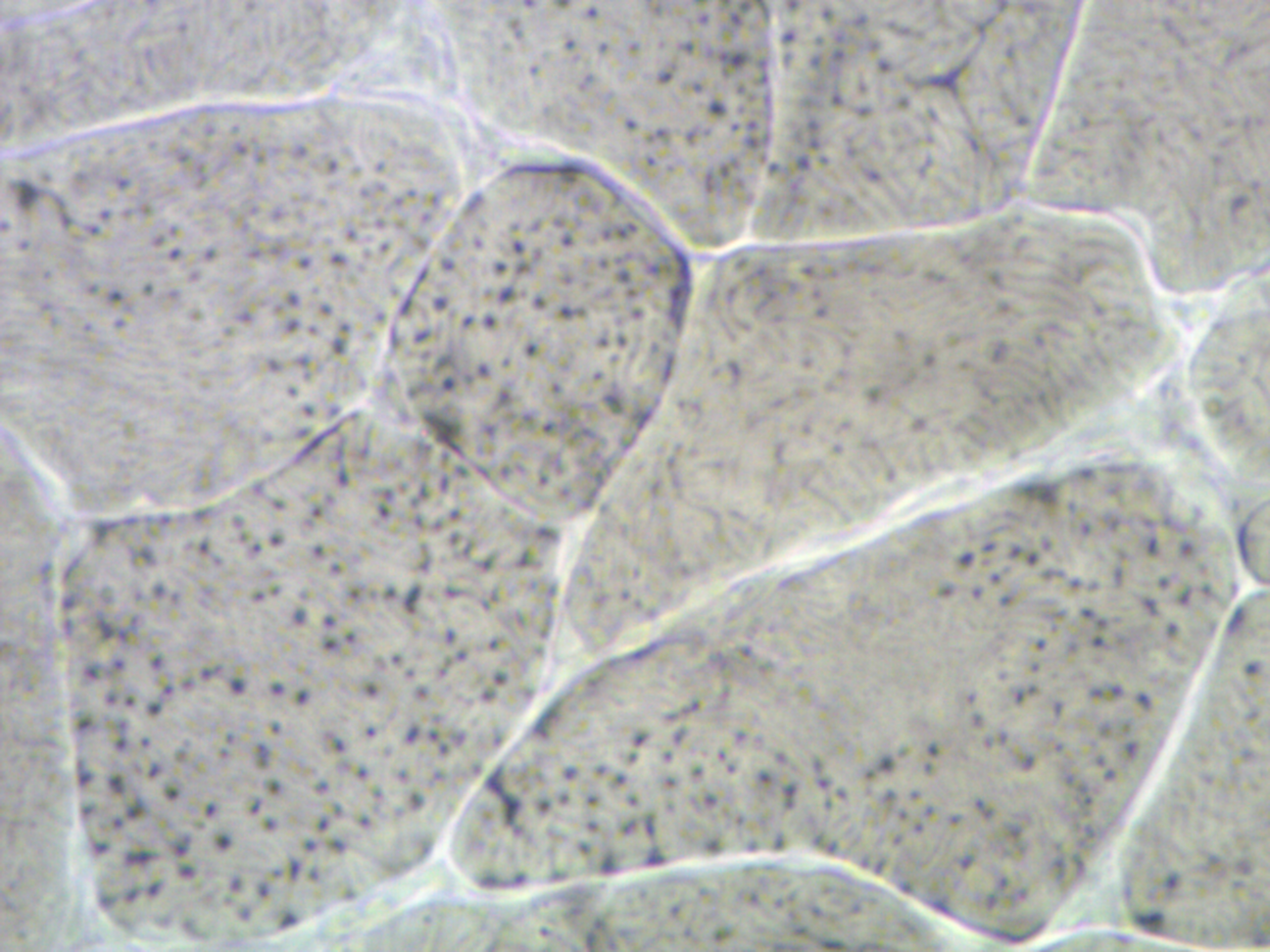

Supplement: Supplementary file 15 — Source data Fig. 7 [file 44319_2026_774_MOESM15_ESM.zip › Figure 7/7G/Transgenic aged Sudan Black B staining.tif]

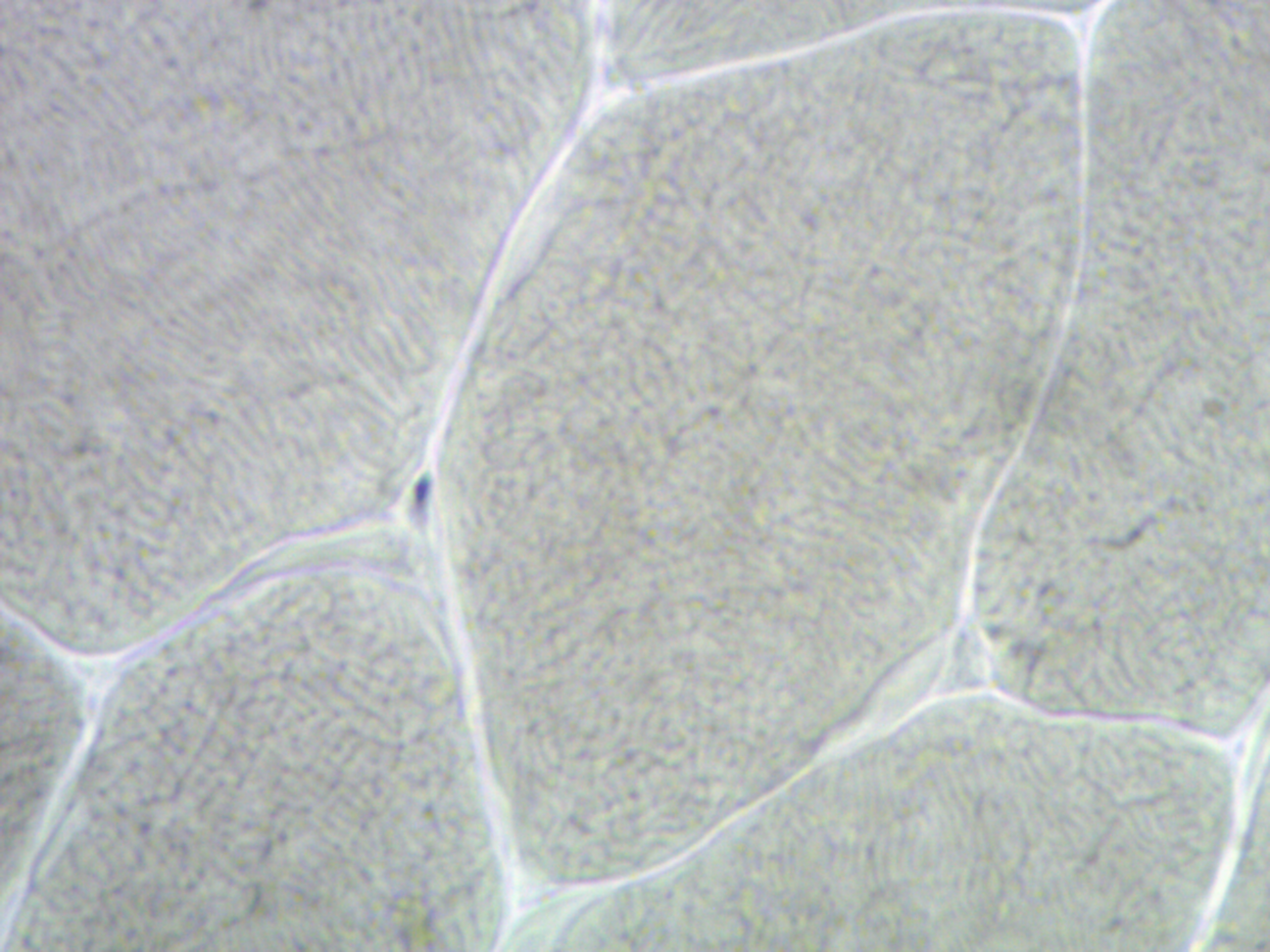

Supplement: Supplementary file 15 — Source data Fig. 7 [file 44319_2026_774_MOESM15_ESM.zip › Figure 7/7G/Wildtype aged Sudan Black B staining.tif]

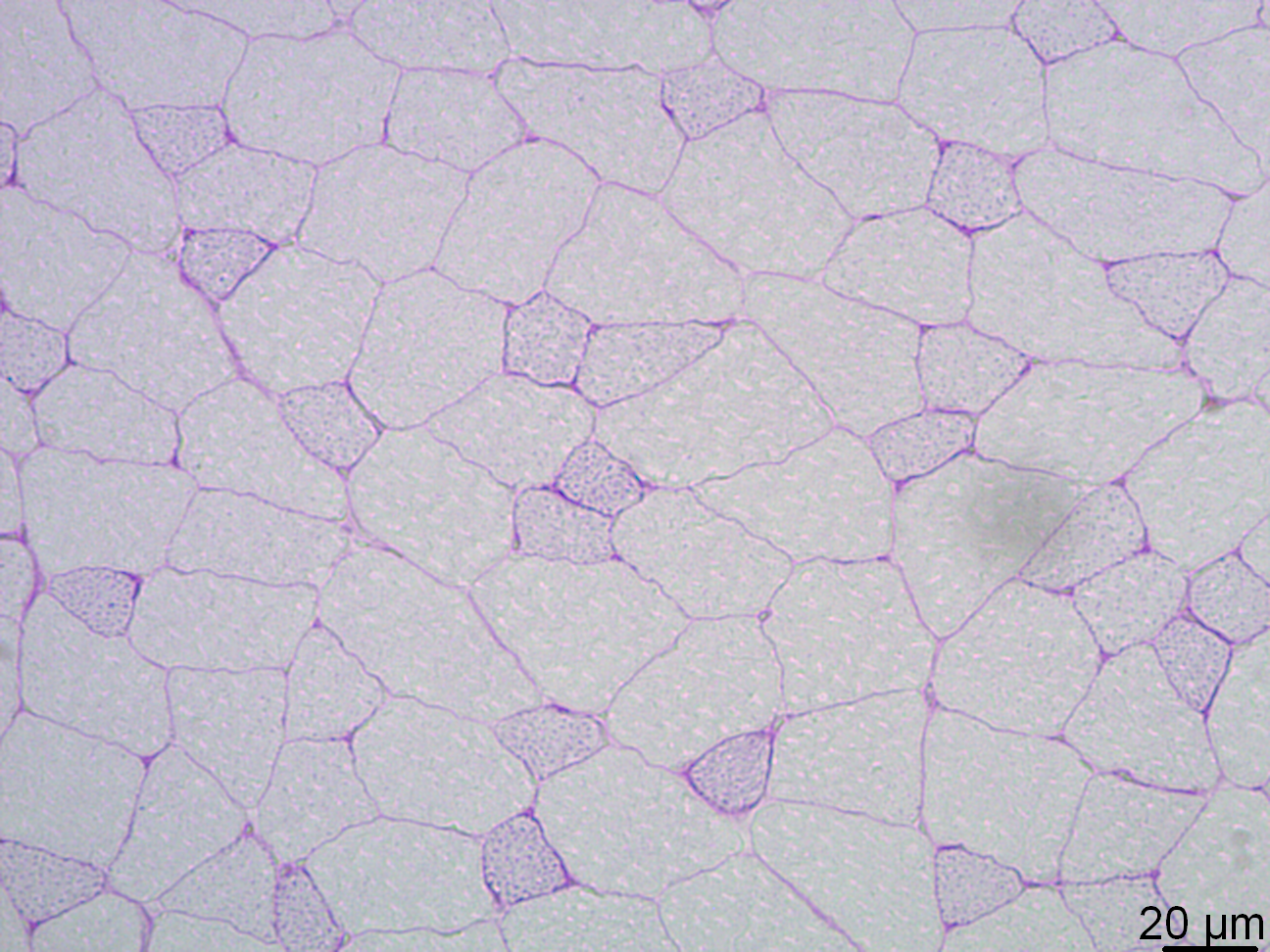

Supplement: Supplementary file 15 — Source data Fig. 7 [file 44319_2026_774_MOESM15_ESM.zip › Figure 7/7F/Transgenic 2-month-old Quadricep PAS staining.tif]

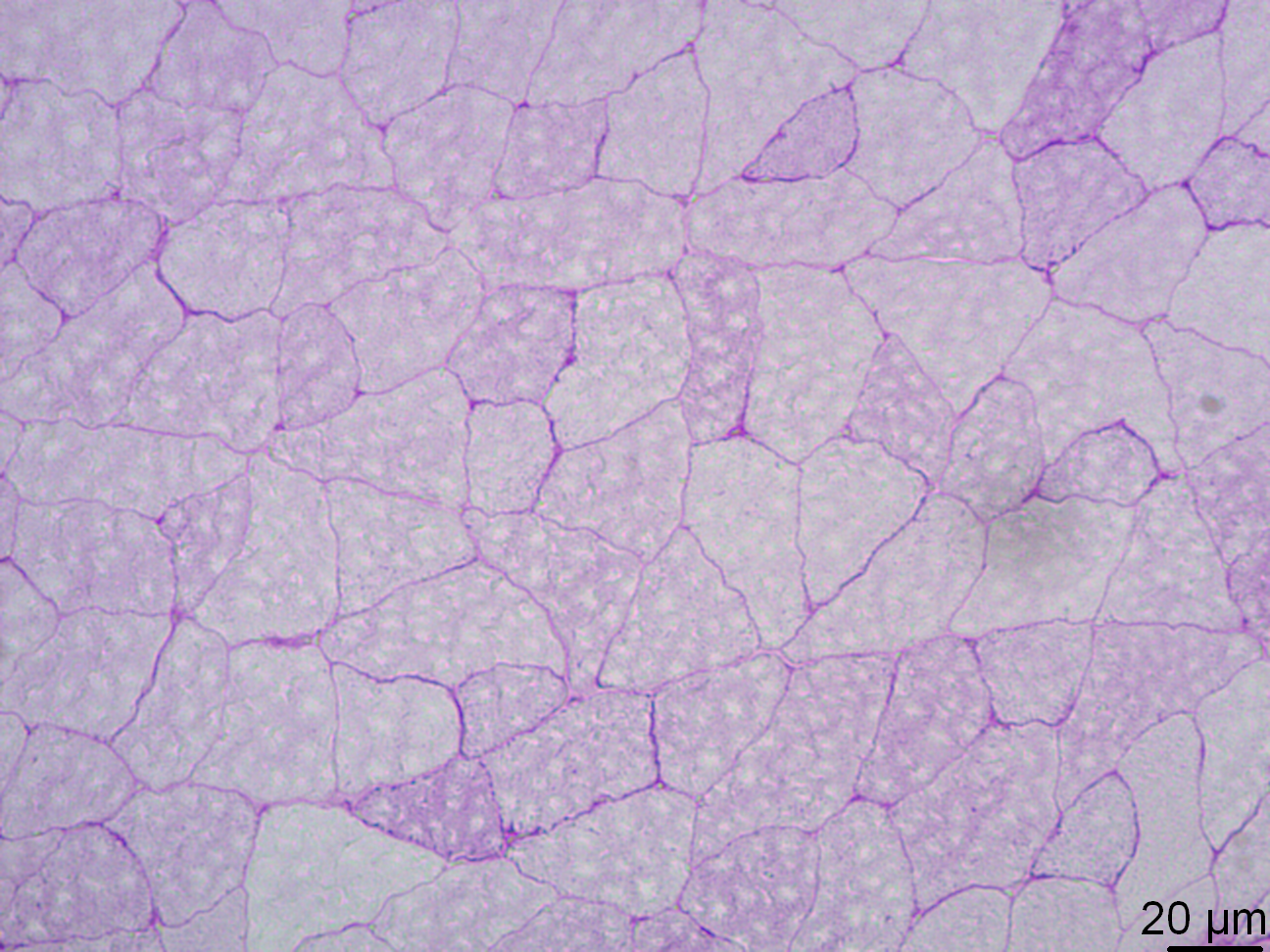

Supplement: Supplementary file 15 — Source data Fig. 7 [file 44319_2026_774_MOESM15_ESM.zip › Figure 7/7F/Wildtype 27-month-old Quadricep PAS staining.tif]

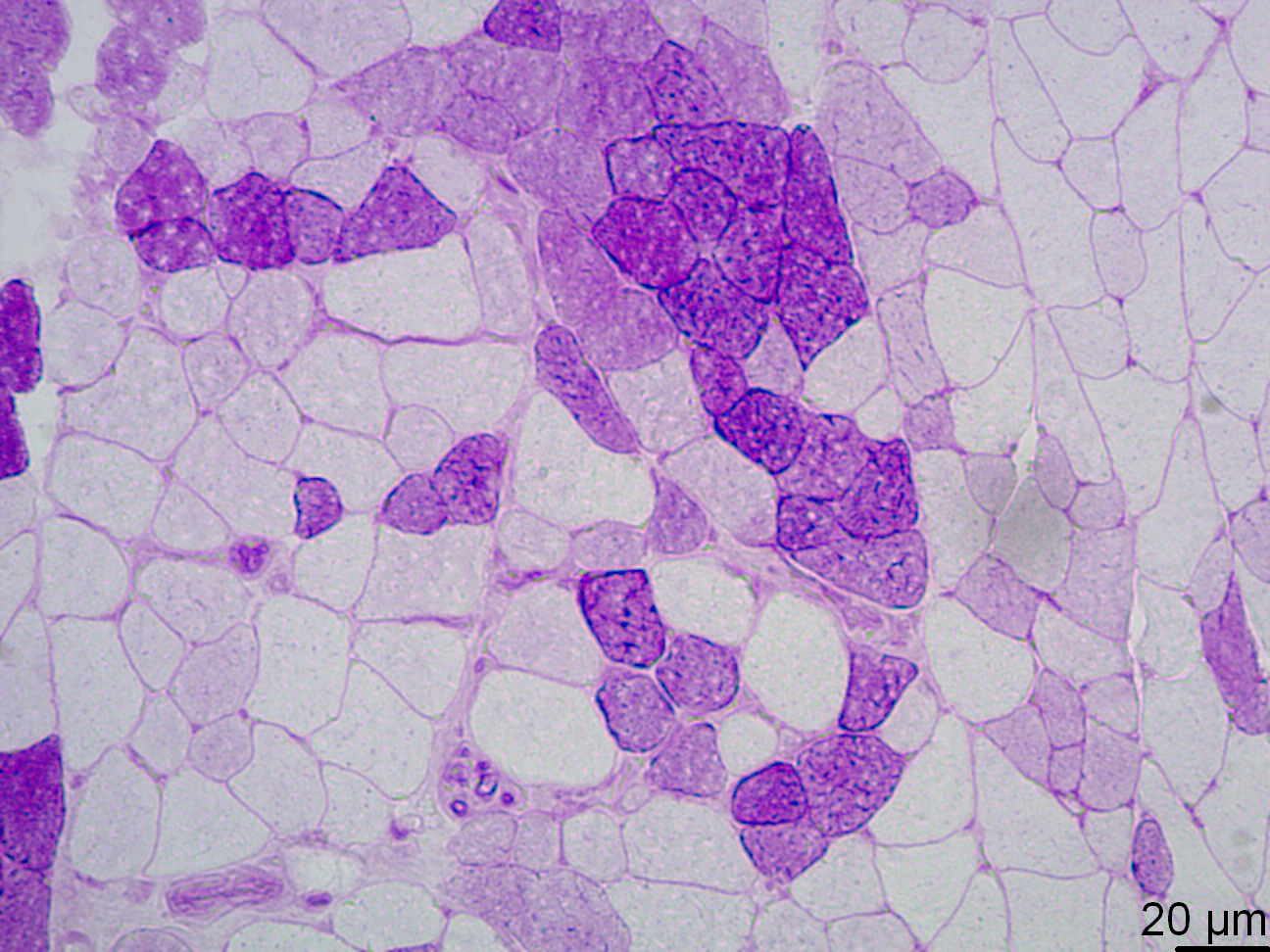

Supplement: Supplementary file 15 — Source data Fig. 7 [file 44319_2026_774_MOESM15_ESM.zip › Figure 7/7F/Transgenic 27-month-old Quadricep PAS staining.tif]

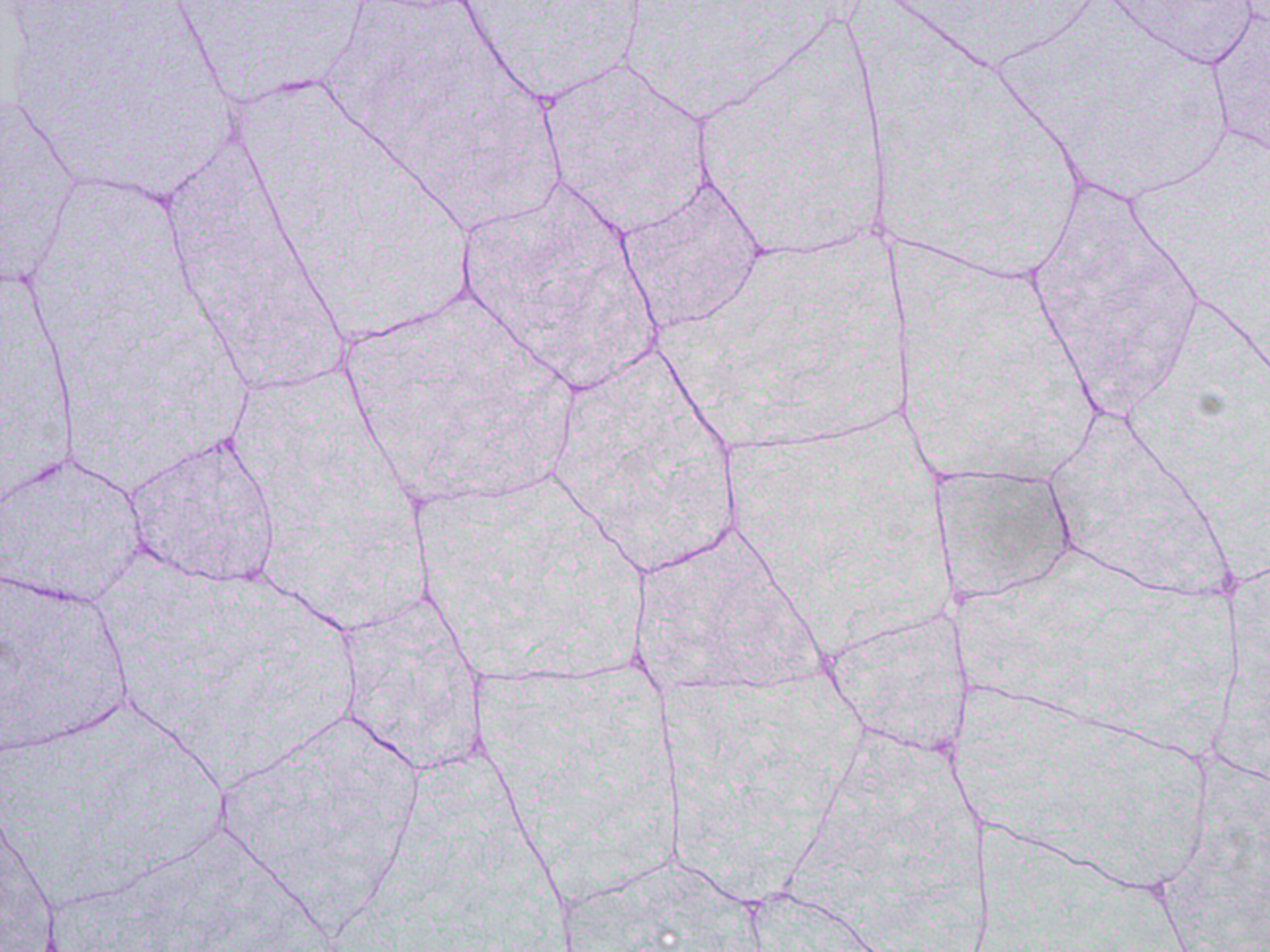

Supplement: Supplementary file 15 — Source data Fig. 7 [file 44319_2026_774_MOESM15_ESM.zip › Figure 7/7F/Wildtype 2-month-old Quadricep PAS staining.tif]

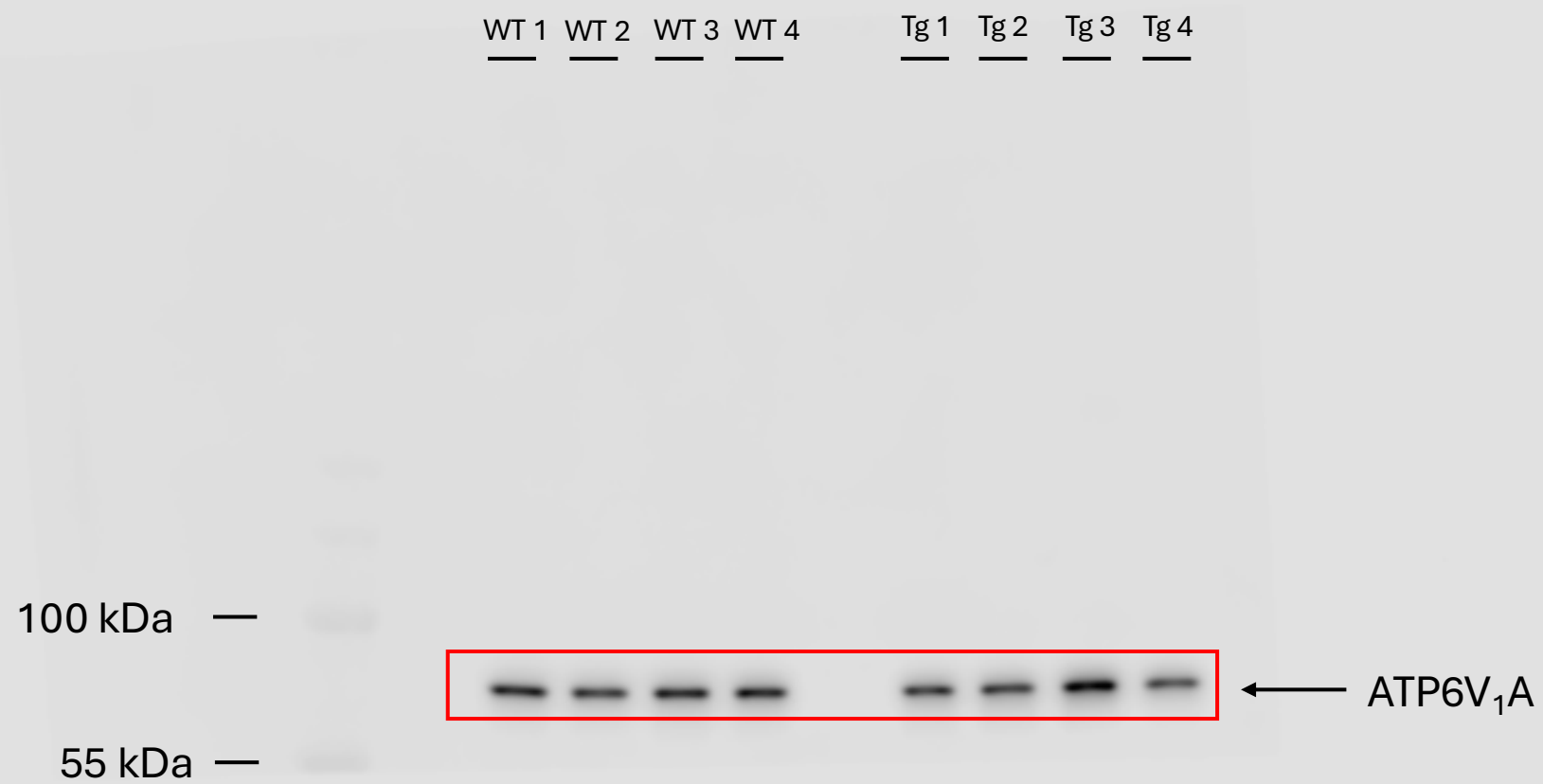

Supplement: Supplementary file 15 — Source data Fig. 7 [file 44319_2026_774_MOESM15_ESM.zip › Figure 7/7A/ATP6V1A Western blot.pdf]

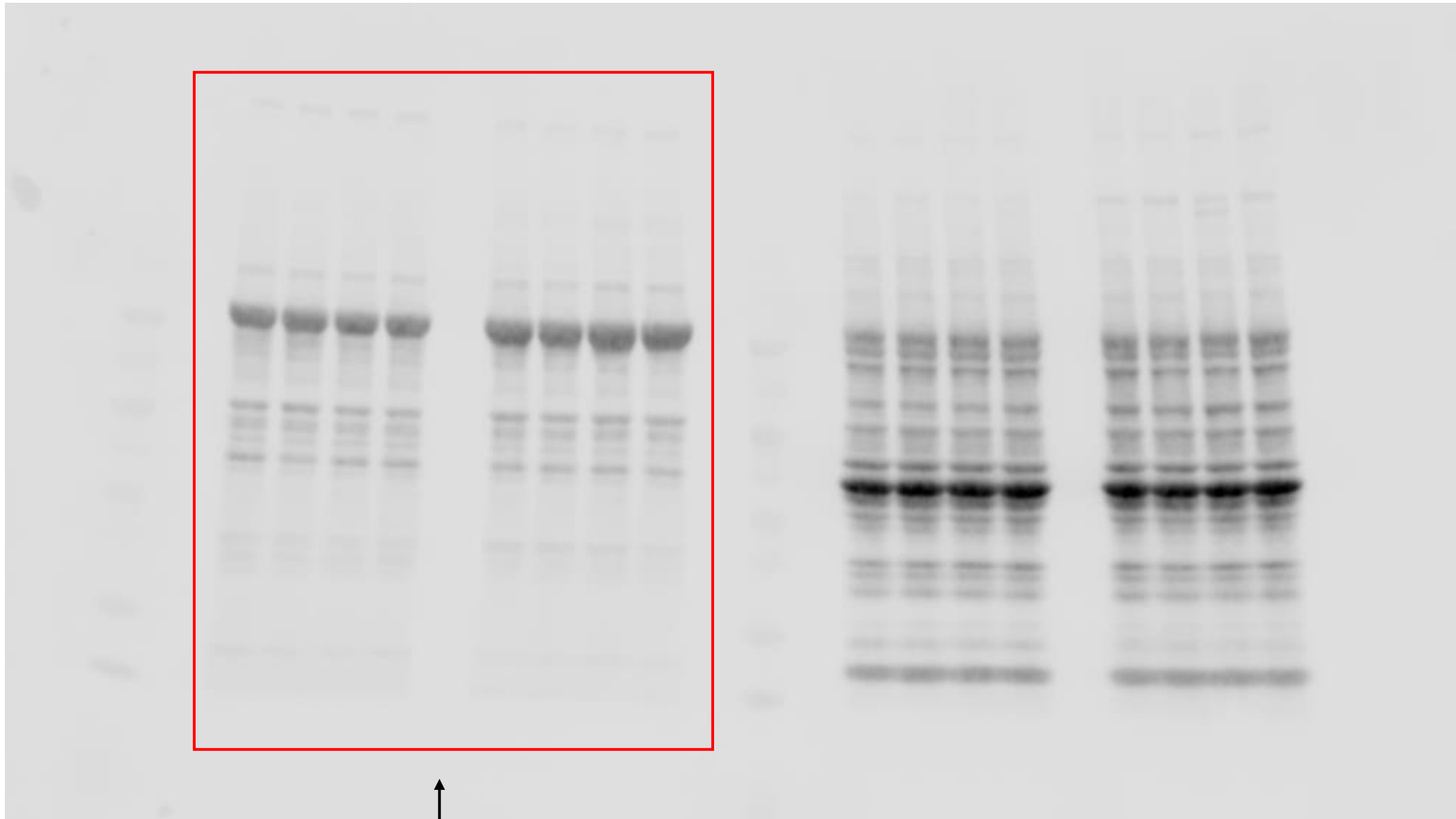

Used for ATP6V<sub>0</sub>D1 and ATP6V<sub>1</sub>A normalization

Supplement: Supplementary file 15 — Source data Fig. 7 [file 44319_2026_774_MOESM15_ESM.zip › Figure 7/7A/TPS for ATP6VoD1 and ATP6V1A normalization.pdf]

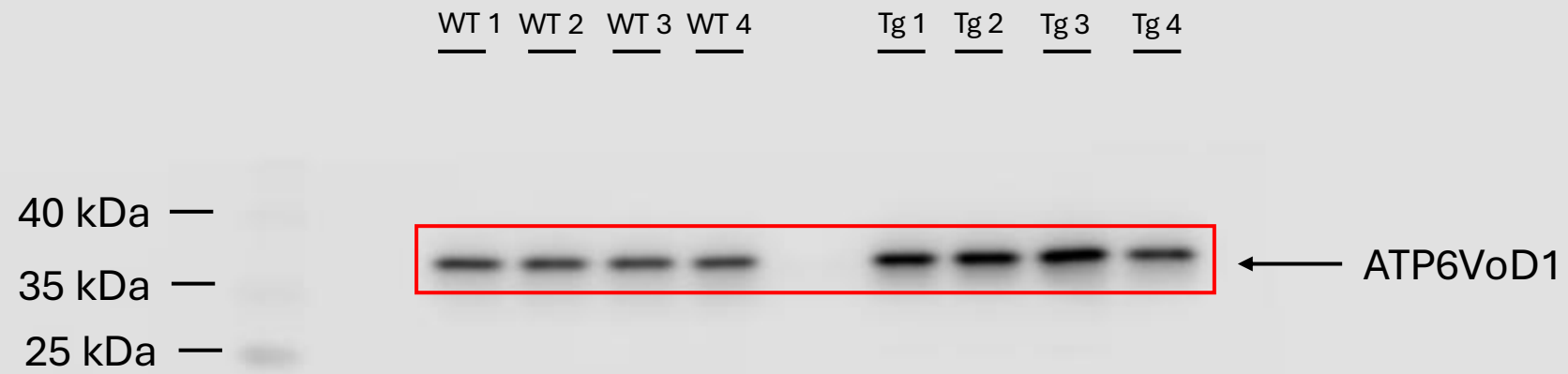

Supplement: Supplementary file 15 — Source data Fig. 7 [file 44319_2026_774_MOESM15_ESM.zip › Figure 7/7A/ATP6VoD1 Western blot.pdf]

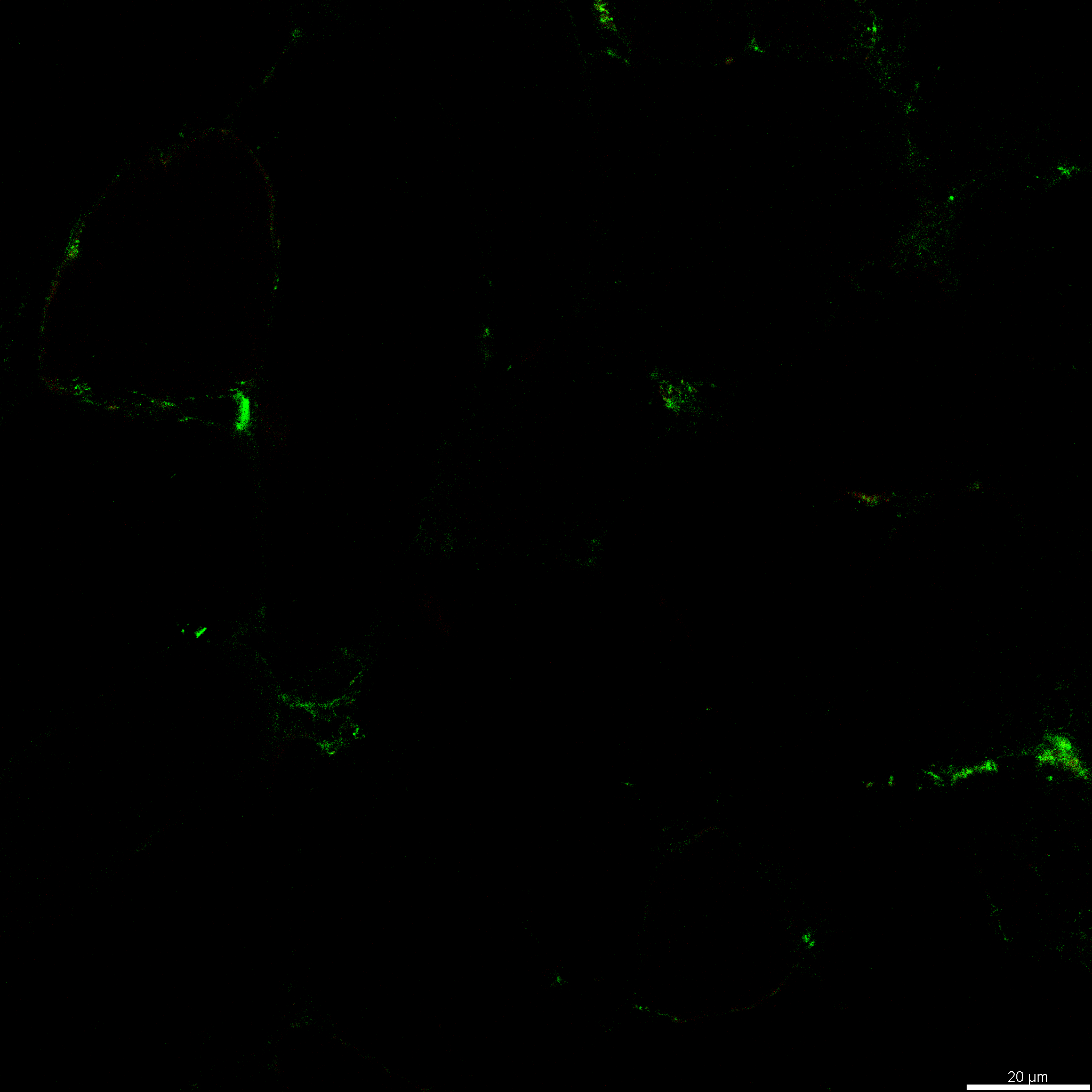

Supplement: Supplementary file 15 — Source data Fig. 7 [file 44319_2026_774_MOESM15_ESM.zip › Figure 7/7H/Wildtype LAMP2 and LGALS3 fluoresence overlap.tif]

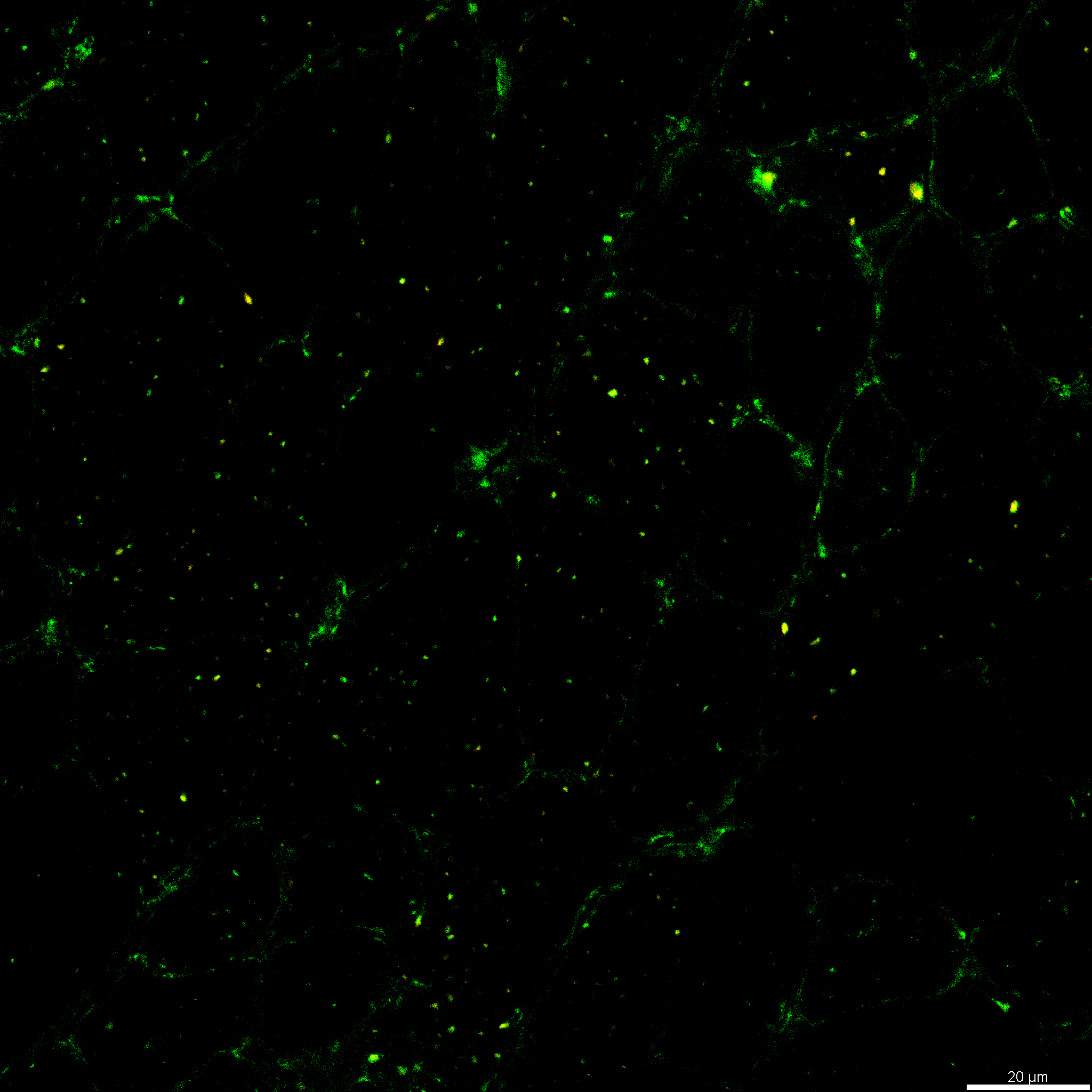

Supplement: Supplementary file 15 — Source data Fig. 7 [file 44319_2026_774_MOESM15_ESM.zip › Figure 7/7H/Transgenic LAMP2 and LGALS3 fluoresence overlap.tif]

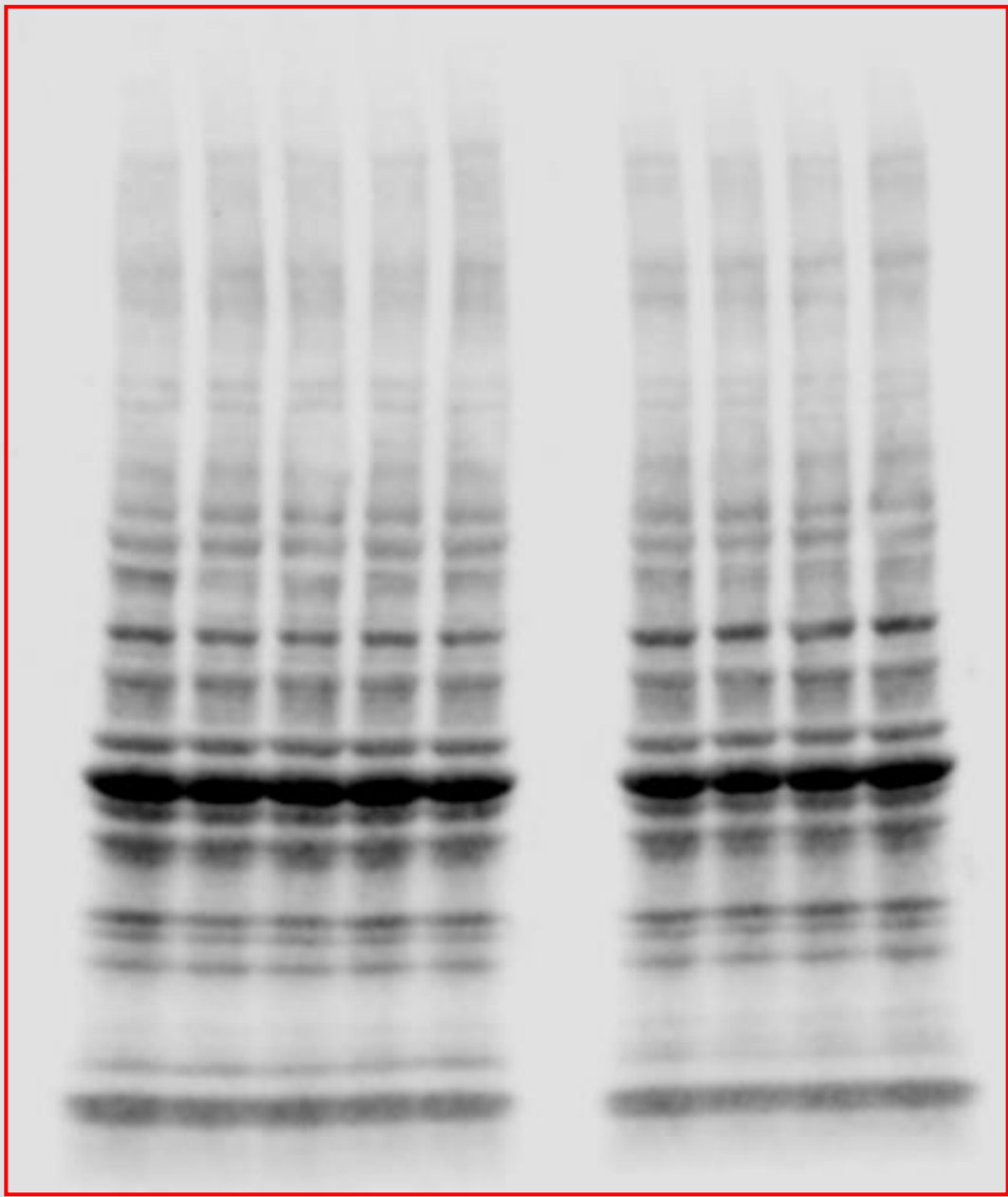

Supplement: Supplementary file 16 — Source data Fig. 8 [file 44319_2026_774_MOESM16_ESM.zip › Figure 8/8A/Total protein staining for puromycin normalization.pdf]
