# Supplementary material for: Mitochondrial protein import stress causes lysosomal damage and progressive tissue atrophy
Source: EMBO Rep. 2026 Apr 27;27(11):2973–3000. doi: 10.1038/s44319-026-00774-9 (PMC13260833; doi:10.1038/s44319-026-00774-9)
Supplement: Supplementary file 16 — Source data Fig. 8 [file 44319_2026_774_MOESM16_ESM.zip › Figure 8/8A/Puromycin western blot.pdf]

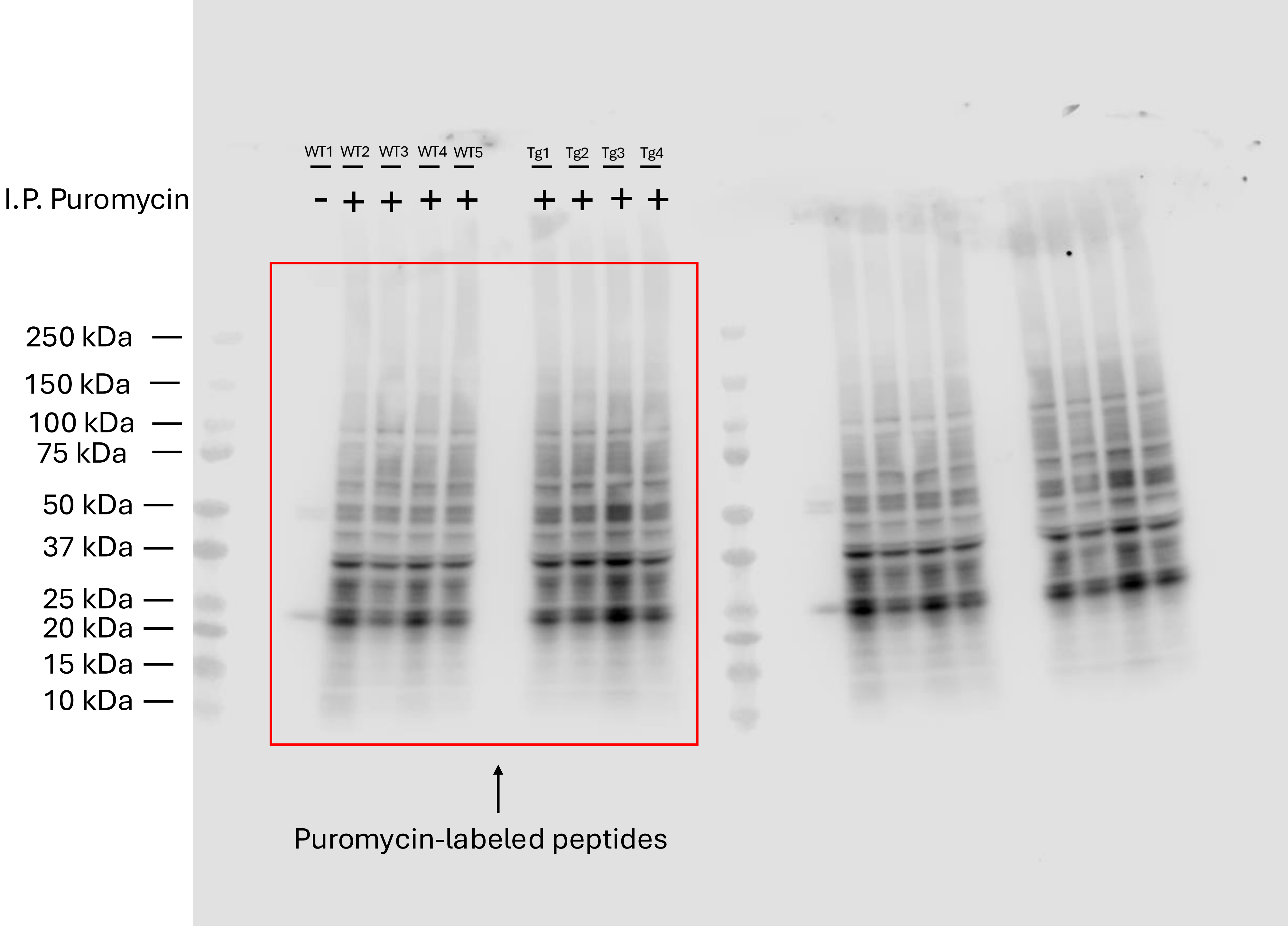
WT1 WT2 WT3 WT4 WT5 Tg1 Tg2 Tg3 Tg4
I.P. Puromycin
- + + + + + + + +
250 kDa
150 kDa
100 kDa
75 kDa
50 kDa
37 kDa
25 kDa
20 kDa
15 kDa
10 kDa
Puromycin-labeled peptides
