# Supplementary material for: Mitochondrial protein import stress causes lysosomal damage and progressive tissue atrophy
Source: EMBO Rep. 2026 Apr 27;27(11):2973–3000. doi: 10.1038/s44319-026-00774-9 (PMC13260833; doi:10.1038/s44319-026-00774-9)
Supplement: Supplementary file 17 — Expanded View Figures [file 44319_2026_774_MOESM17_ESM.pdf]

Expanded View Figures

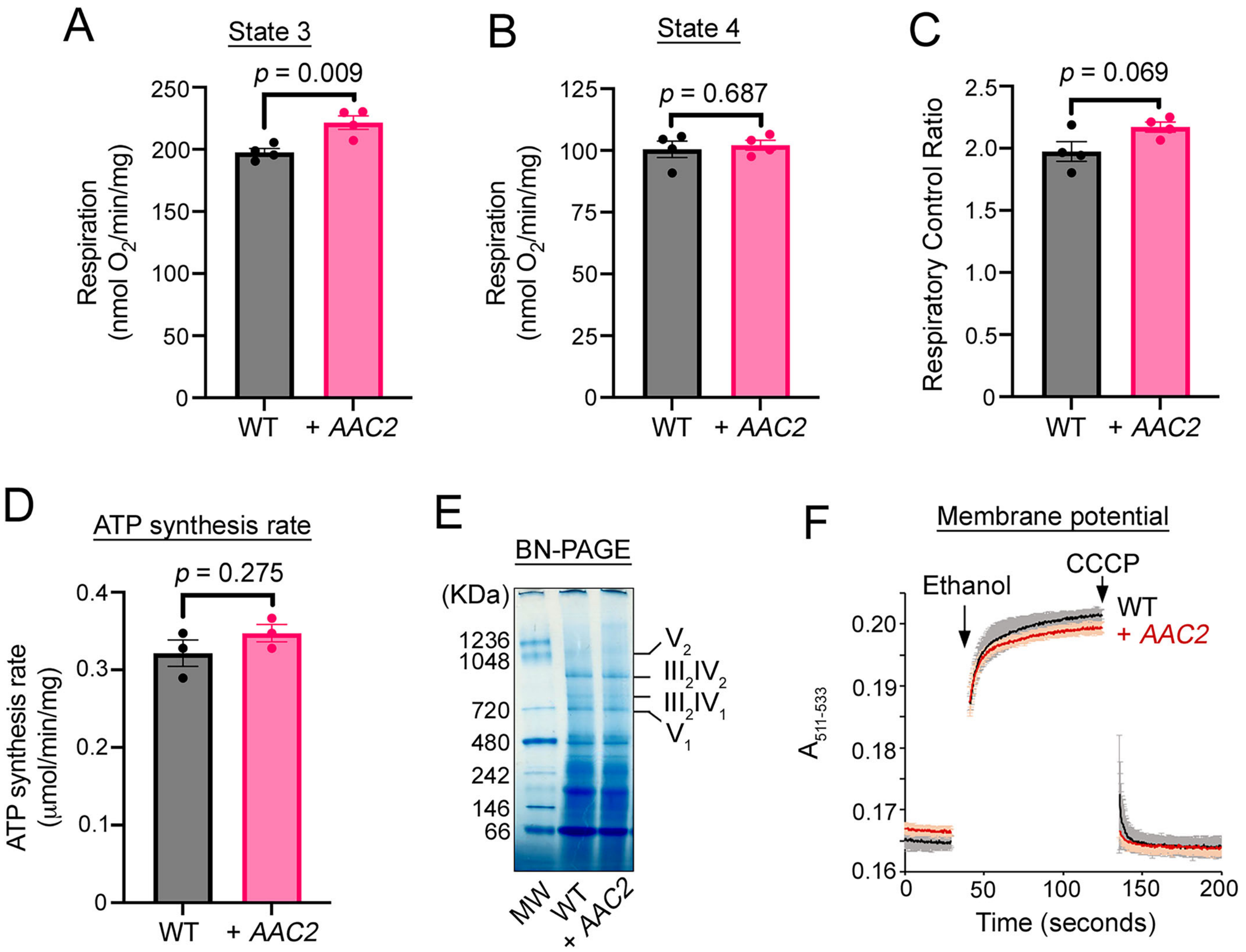

Figure EV1. Expression of an extra copy of *AAC2* integrated into the chromosome has little effect on mitochondrial function.

Mitochondria were isolated from BY4741/AN1 (+*AAC2*) (*α, lys2, his3, leu2, ura3, trp1Δ::AAC2-HIS3*) and the control strain BY4741 (WT) (*α, lys2, his3, leu2, ura3*) grown in complete galactose (YPGal) medium. (**A**, **B**) Ethanol-stimulated state 3 and state 4 respiration. (**C**) Respiratory control ratio. (**D**) ATP synthesis rate. (**E**) Respiratory complex assembly. V_1, complex V or ATP synthetase; V_2, dimer of complex V; III_2IV_2, supercomplex with the denoted stoichiometry; III_2IV_1, supercomplex with the denoted stoichiometry. (**F**) Membrane potential after the energization of mitochondria with ethanol. The error bars for three biological replicates are shown. For (**A–D**), data were means ± SEM of 3–4 biological replicates. *P* values were calculated by a two-tailed Student's *t*-test).

 © The Author(s)

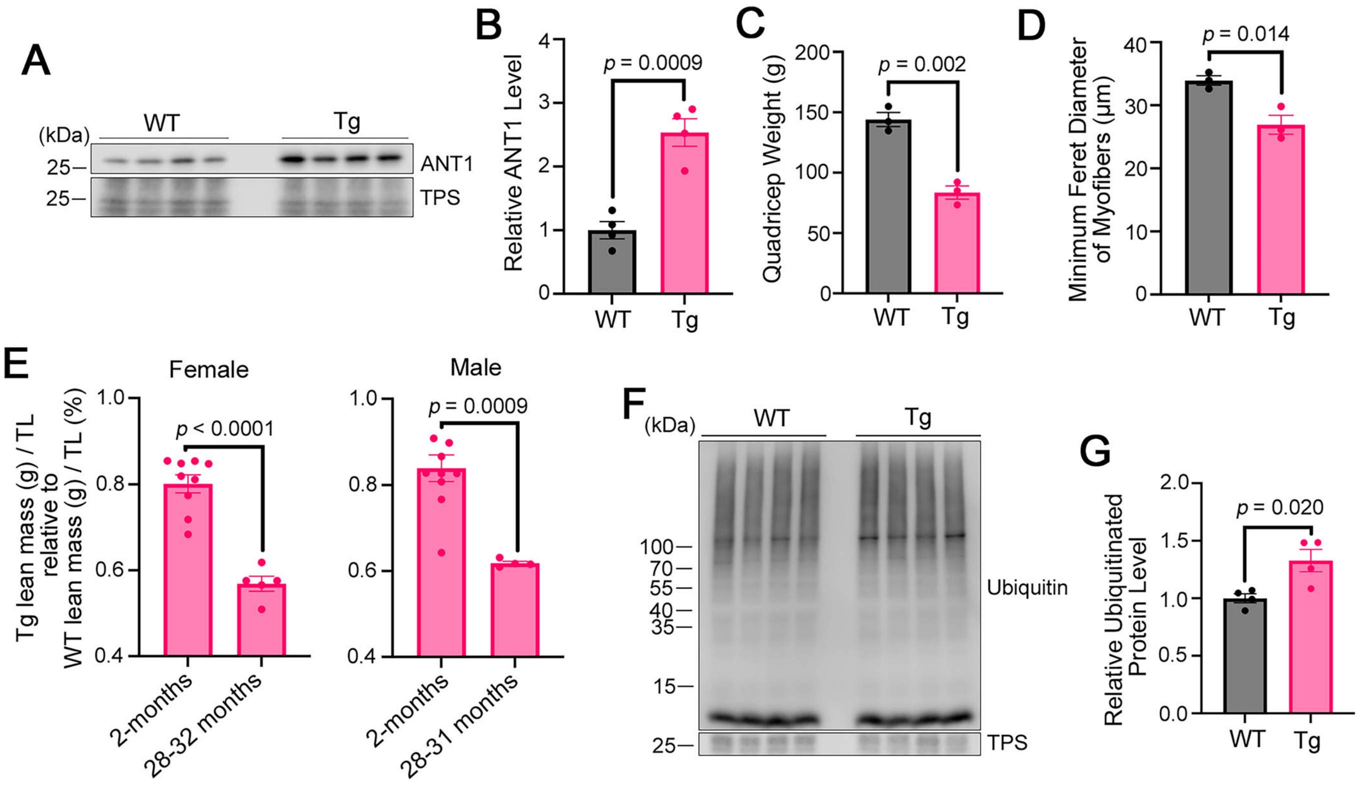

Figure EV2. ***Ant1* overexpression in *Ant1*^{Tg}/+ mice causes progressive muscle atrophy during aging and affects quadriceps muscle weight, myofiber size, and protein ubiquitination levels at 2 months of age.**

(**A**) Immunoblotting showing ANT1 protein levels in *Ant1*^{Tg}/+ (Tg) quadriceps relative to age-matched wild type controls (WT) at 2 months of age ($n = 4$/genotype; female). (**B**) Quantification of (**A**). (**C**) Quadriceps muscle weight at 2 months of age ($n = 3$/genotype; female). (**D**) Minimum Feret diameter of myofibers at 2 months of age ($n = 3$/genotype; female). (**E**) DXA scanning showing lean mass of *Ant1*^{Tg}/+ mice relative to wild-type controls at different ages ($n = 4$–10/genotype/sex) normalized by tibia length (TL). (**F**) Immunoblotting of total cell lysates using an anti-ubiquitin antibody ($n = 4$/genotype; female). (**G**) Quantification of (**F**). Data in (**A**–**D**, **G**) are means ± SEM. The "*n*" denotes the number of animals. *P* values were calculated by a two-tailed Student's *t*-test. TPS total protein stain.

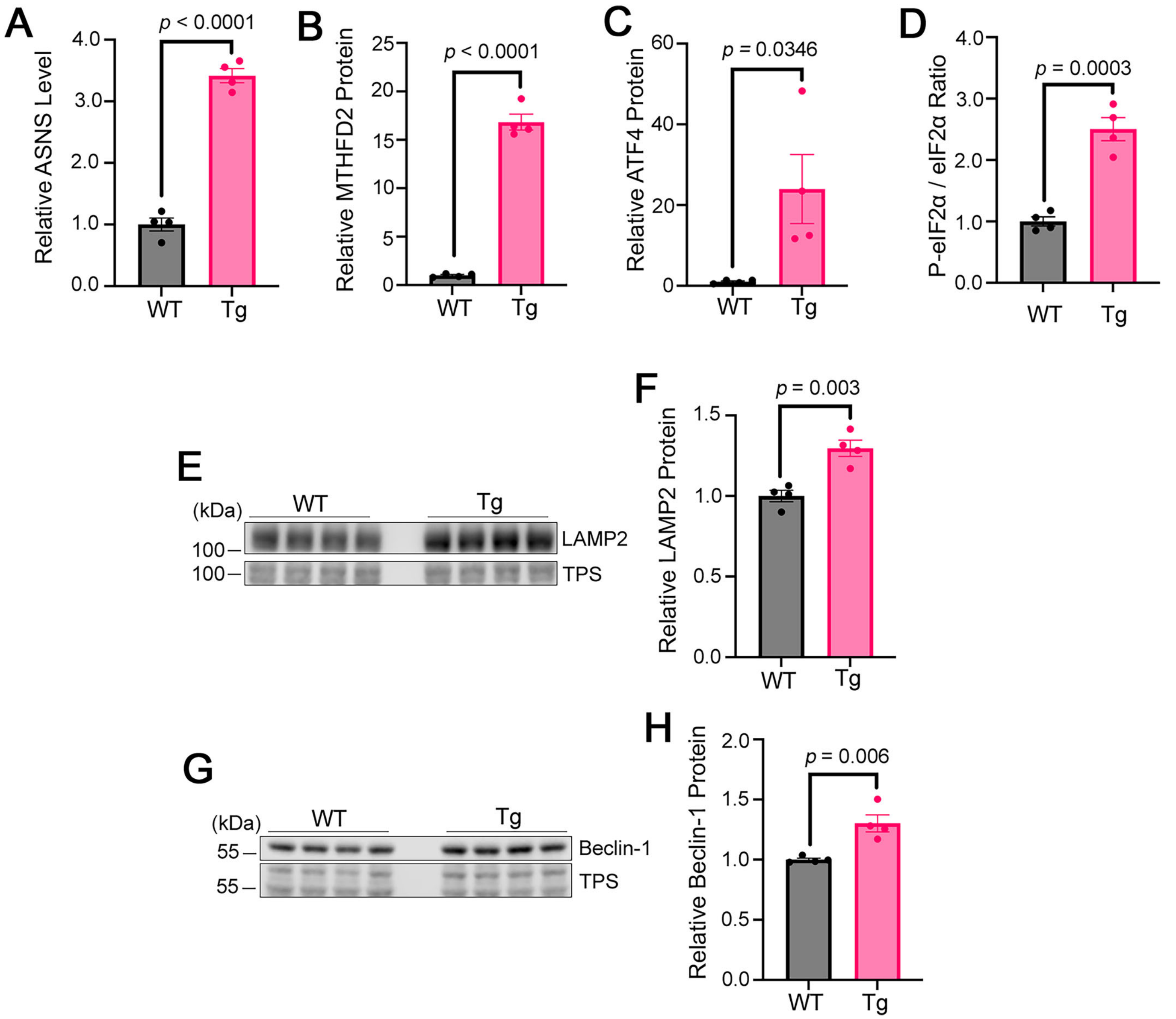

Figure EV3. Immunoblot analysis validating transcriptional activation of ISR and autophagy-related genes in 2-month-old *Ant1*^{Tg}/+ muscle.

(**A–C**) Quantification of relative protein levels of ASNS (**A**), MTHFD2 (**B**) and ATF4 (**C**) (Fig. 6B). (**D**) Quantification of the relative P-eIF2α/eIF2α ratio (Fig. 6B). (**E**) Immunoblotting of LAMP2 ($n = 4$/genotype; male). (**F**) Quantification of (**E**). (**G**) Immunoblotting of Beclin-1 ($n = 4$/genotype; male). (**H**) Quantification of (**G**). TPS, total protein stain. Error bars are means ± SEM. *P* values were calculated by a two-tailed Student's *t*-test. The "*n*" denotes the number of animals.

 © The Author(s)

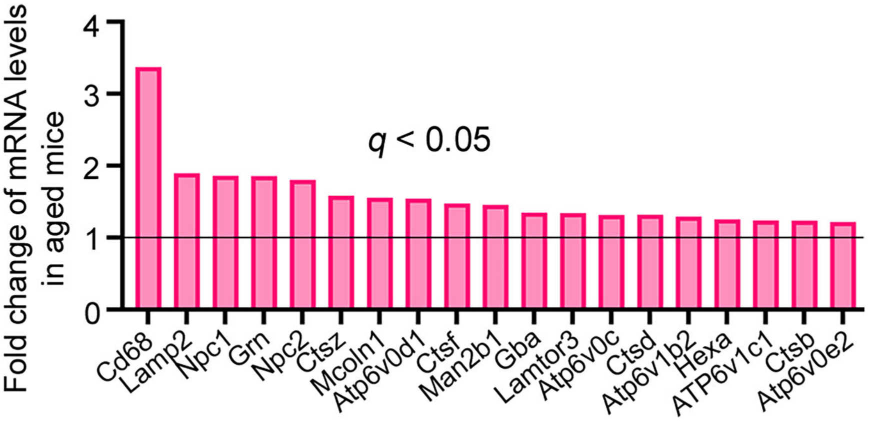

Figure EV4. RNA-seq data showing the genes involved in lysosomal and autophagic functions that are activated in aged (27–30 months) *Ant1*^{Tg}/+ quadriceps muscle relative to age-matched wild type controls ($n = 4$/genotype).

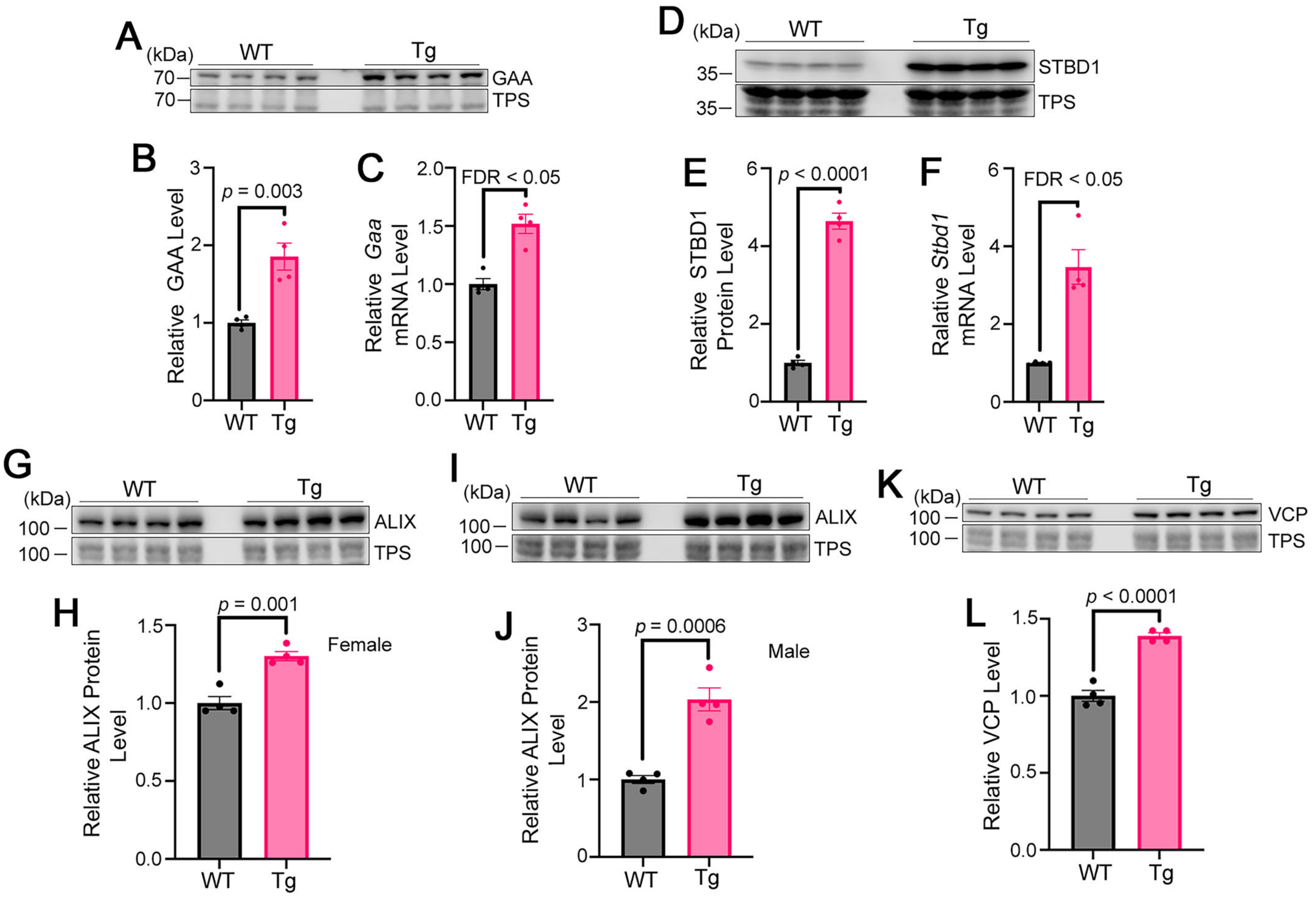

Figure EV5. Immunoblotting showing the increased levels of lysosomal repair-related proteins in 2-month-old *Ant1*^{Tg}/+ quadriceps muscle.

(**A**) Immunoblotting of GAA (acid α-glucosidase) ($n = 4$/genotype; male). (**B**) Quantification of (**A**). (**C**) RNA-seq analysis showing transcriptional activation of *Gaa* in *Ant1*^{Tg}/+ muscle ($n = 4$). (**D**) Immunoblotting of STBD1 ($n = 4$/genotype; male). (**E**) Quantification of (**D**). (**F**) RNA-seq analysis showing transcriptional activation of Stbd1 in *Ant1*^{Tg}/+ muscle. (**G**) Immunoblotting of ALIX in female mice ($n = 4$/genotype). (**H**) Quantification of (**G**). (**I**) Immunoblotting of ALIX in male mice ($n = 4$/genotype). (**J**) Quantification of (**I**). (**K**) Immunoblotting of VCP ($n = 4$/genotype). (**L**) Quantification of (K). TPS total protein stain. Error bars are means ± SEM. *P* values were calculated by a two-tailed Student's *t*-test. The "*n*" denotes the number of animals.

 © The Author(s)
