# Supplementary material for: Mitochondrial protein import stress causes lysosomal damage and progressive tissue atrophy
Source: EMBO Rep. 2026 Apr 27;27(11):2973–3000. doi: 10.1038/s44319-026-00774-9 (PMC13260833; doi:10.1038/s44319-026-00774-9)
Supplement: Supplementary file 15 — Source data Fig. 7 [file CTSL western blot.pdf › Figure 7/7C/CTSL western blot.pdf]

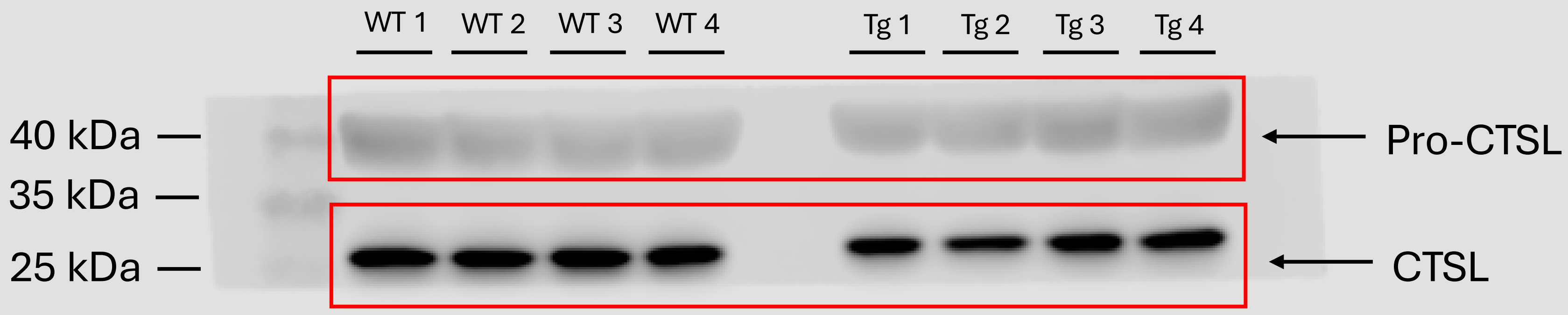
WT 1
WT 2
WT 3
WT 4
Tg 1
Tg 2
Tg 3
Tg 4
40 kDa
35 kDa
25 kDa
Pro-CTSL
CTSL
