# Supplementary material for: Mitochondrial protein import stress causes lysosomal damage and progressive tissue atrophy
Source: EMBO Rep. 2026 Apr 27;27(11):2973–3000. doi: 10.1038/s44319-026-00774-9 (PMC13260833; doi:10.1038/s44319-026-00774-9)
Supplement: Supplementary file 3 — Peer Review File [file 44319_2026_774_MOESM3_ESM.pdf]

EMBO reports

Mitochondrial Protein Import Stress Causes Lysosomal Damage and Progressive Tissue Atrophy

Xin Jie Chen, Nicholas Brennan, Xiaowen Wang, Arnav Rana, Sanaea Bhagwagar, Jason Horton, and Patricia Kane

Corresponding author(s): Xin Jie Chen (chenx@upstate.edu)

Review Timeline:

Transfer Date:	30th Sep 25
Editorial Decision:	21st Oct 25
Revision Received:	11th Jan 26
Editorial Decision:	19th Mar 26
Revision Received:	22nd Mar 26
Accepted:	27th Mar 26

Editor: Martina Rembold

Transaction Report: This manuscript was transferred to EMBO reports following peer review at The EMBO Journal.

(Note: With the exception of the correction of typographical or spelling errors that could be a source of ambiguity, letters and reports are not edited. Depending on transfer agreements, referee reports obtained elsewhere may or may not be included in this compilation. Referee reports are anonymous unless the Referee chooses to sign their reports.)

Referee #1:

In this study, the authors employ both yeast and mouse models in which adenosine nucleotide translocase (Ant1) is overexpressed to investigate the link between import stress at the mitochondria and lysosomal dysfunction. In yeast, they find negative genetic interaction between mild overexpression of AAC2 and vacuolar mutants, with an exacerbation of the vacuolar phenotypes at the morphological level and in terms of transcriptional response. They further characterize a transgenic mouse model where ANT1 is overexpressed (in the full body as far as can judge). These mice show muscular atrophy, and a transcriptome and proteome signature that suggests the activation of several stress responses, including lysosomal perturbations. This is not so surprising, given the overexpression of an inner mitochondrial membrane protein. Although the characterization of the mouse model is solid, the conclusion that mitochondrial protein import stress causes lysosomal damage with release of lysosomal enzymes, which in turn lead to excessive protein degradation causing muscular atrophy seems still rather speculative.

Major comments:

1) How many times have the yeast experiments been performed? There are some claims based on quantification of n=1. This is the case for example for the experiments in Figs 1 and 3.

2) One major concern is that the authors never show that there is actual import stress. Is overexpressed ACC2 or ANT1 in the muscle poorly imported? Can this be seen for instance at early time points? Is there accumulation of other mitochondrial precursors? I think that this is a crucial data to confirm the proposed model.

3)The authors infer a defect in V-ATPase assembly based on relative abundance of components in a western blot (Fig. 5C). This is not convincing, as the differences are minor and assembly is not directly evaluated. Native gels to look directly at assembly would be a better method.

4)The authors convincingly show that overexpression of ANT1 in the mouse causes muscular atrophy, triggers the integrated stress response, and other downstream pathways already associated with mitochondrial dysfunction. Moreover, several data are suggestive of possible lysosomal impairment. The conclusion that it is the release of lysosomal enzymes in the cytosol that leads to the muscular atrophy is however not so convincing. This conclusion is based on correlating three findings: unaffected protein synthesis,

increased cathepsin activity in the cytosol, and decrease total proteins in the muscle. These are interesting correlations, but do not really prove the causality of events. Can it simply be that enhanced autophagy leads to protein degradation? Can the authors show that there is an accelerated protein degradation? Can the authors exclude that the proteasome is responsible for the protein degradation? Proteasome is known to be hyperactive when autophagy is impaired.

Minor comments:
-Please specify what is trp1 in figure 1i
-Page 9: the sentence "mitochondria respiration is little unaffected" is confusing.

Referee #2:

The AAC2 carrier protein mutant variant and its pathological human variant cause clogging the mitochondrial protein import translocases when co/overexpressed. This highly interesting study sheds some light on the molecular mechanisms involved in pathology caused by the mitochondrial protein import stress caused by AAC2/human ANT-linked pathology. The authors find the vacuolar defects, insufficient deacidification as well as the changes in V-ATPase and propose that defective lysosomal function is the main cause of the carrier toxicity.
The main strength and novelty of this paper is a new connection between mitochondrial protein import stress and lysosomal dysfunction, mediated likely through defects in V-ATPase. Also the experiments in mice bring a very interesting insight into the pathology.
The weakness of this study are insufficient molecular mechanisms. The authors convincingly show the defects in vacuole/lysosome, however the underlying reason is unclear.
- Does AAC2 variant is a substrate for lysosomal degradation?
- What is the underlying cause of V-ATPase defects? Is this a direct interaction or interference with the V ATPase assembly - the mode of V ATPase deregulation is unclear and could in fact originate from yet unknown reason and not direct interference by AAC2.
- Is the proteasome activity, earlier reported to be upregulated during mitochondrial import stress, changed? Does this activity may indirectly affect V ATPase?
- Is the identified link typical only for AAC2/ANT pathology or other models of protein import stress also affect vacuolar/lysosomal pathway, via V ATPase?

Referee #3:

This study investigates the mechanistic link between mitochondrial protein import stress and lysosomal/vacuolar damage, using both yeast and a transgenic mouse model overexpressing the mitochondrial adenine nucleotide translocase (ANT1). The authors show that an unimported mitochondrial protein is trafficked to the vacuole/lysosome for degradation in cells overexpressing AAC2, and overloading this pathway disrupts V-ATPase assembly, vacuolar acidification, and lysosomal integrity. In yeast, this stress synergizes with V-ATPase mutations to impair growth. In mice, ANT1 overexpression triggers lysosomal dysfunction, protease leakage into the cytosol, and progressive muscle atrophy without bioenergetic deficits. Transcriptomic and proteomic analyses reveal conserved stress signatures across species, including activation of amino acid starvation responses, autophagy, and lysosomal repair pathways. The authors propose a model in which a conserved mitochondria-to-lysosome proteostatic relay contributes to tissue atrophy in aging and disease.

Overall, this study describes some very interesting phenotypes that occur in yeast and mammals with AAC/ANT mutations/expression changes, and raises an interesting and testable hypothesis/model that unimported mitochondrial proteins drive lysosomal failure in this context. While the manuscript contains many very intriguing phenotypes and striking changes in lysosome function, the overall hypothesis/conclusion is not well supported by the current data. As it currently stands, the data supporting the claim that unimported proteins are the driver of the vacuole/lysosome issues is not robust, and needs more investigation to rigorously test this model. It is also not entirely clear in the moue models whether the lysosome dysfunction downstream of import stress if the major driver of muscle atrophy, as rescue experiments are not included. These major limitations would need to be addressed by the authors prior to publication, in addition to the other points raised below.

Major Points:

1. Lack of support for overall model in the data: The authors' hypothesis and model that unimported proteins are driving vacuole and lysosome issues upon AAC/ANT perturbation. However, the evidence supporting this is rather weak. In yeast, the authors show one non-endogenous protein accumulating in the vacuole, and a more thorough examination of the extent of endogenous vacuole targeting of mitochondrial unimported precursors is not

included. Thus, it remains unclear how prominent this effect is. In the mouse model, proteomics of the cytosol show more mitochondrial proteins, but it's not clear which if any of these are targeted to the lysosome. Moreover, in both systems, how these unimported proteins could mechanistically cause the phenotypes described remains unclear. Why are the vacuoles and lysosome degradation pathways not capable of handling these proteins in this context? What is the route proteins use to get targeted to the vacuole/lysosome? The authors need some sort of mechanistic explanation here connecting these dots. Otherwise, it seems equally plausible that the vacuole/lysosome effects with AAC2/ANT are driven by another aspect of the toxic protein on cells.

2. Another issue is the conclusion that the synthetic phenotypes between loss of V-ATPase and AAC2 overexpression stems from unimported protein accumulation. While some investigation into mitochondrial ROS/OXPHOS is provided, it again seems very plausible given the vacuoles many impacts on the cells that the lethality may be linked to alterations in pH, iron, calcium ions, etc. in the double mutant. The authors should perform rescue experiments in the double mutants to test if doing things like adding iron to the media or altering the pH can suppress the growth defects. This may suggest that that lethality is caused through a slightly different mechanism than what is proposed here. Again, the data is robust that there are vacuole/lysosome defects, the mechanism underlying that connection is just not entirely clear right now.

3. Causality vs. correlation in in vivo findings: While the mouse model shows lysosomal damage concurrent with mitochondrial protein import stress, direct evidence that lysosomal dysfunction is the primary driver of muscle atrophy is not fully established (e.g., genetic or pharmacologic rescue experiments targeting lysosome function are missing).

4. Specificity of AAC2/ANT1: Do other cloggers cause the same effects? If the downstream driver is unimported proteins, then it seems likely that other cloggers should cause the same issue. Also, the switching between AAC2 overexpression and the mutant version throughout the first few figures is a bit confusing. It may be helpful to stick with one version or the other. If the effects are specific to one form or another, what does that suggest about the model?

Response to reviewers' comments – EMBOJ-2025-121713 Decision letter

Referee #1:

Major comments:

1) How many times have the yeast experiments been performed? There are some claims based on quantification of n=1. This is the case for example for the experiments in Figs 1 and 3.

Response – We thank the reviewer for this important observation. For genetic interactions, we always analyze 2-3 independent zygotes to avoid aberrations in genetic segregations that may skew the results. For the imaging experiments, we perform at least two independent experiments to insure reproducibility. For RNA-seq analysis, three and four biological replicates are included for yeast and mouse skeletal muscle samples respectively. For transparency, we added the information in either in the figure legends or in the Method sections.

2) One major concern is that the authors never show that there is actual import stress. Is overexpressed ACC2 or ANT1 in the muscle poorly imported? Can this be seen for instance at early time points? Is there accumulation of other mitochondrial precursors? I think that this is a crucial data to confirm the proposed model.

Response – Accumulation of ANT1 in the cytosolic fraction is increased by 2-fold, as revealed by mass spectrometry (Supplemental Table 4). Despite that unimported mitochondrial proteins are predicted to be unstable in the cytosol, we found that many mitochondrial proteins accumulate in the cytosol of young murine muscle cells at the age of two months (Figure 4i). Our previous studies have shown that many mitochondrial proteins, including Aac2, are retained in the cytosolic fraction in cells expressing GAL10-aac2(A128P) (X. Wang, X. J. Chen. Nature 524, 481-484 (2015).

3) The authors infer a defect in V-ATPase assembly based on relative abundance of components in a western blot (Fig. 5C). This is not convincing, as the differences are minor and assembly is not directly evaluated. Native gels to look directly at assembly would be a better method.

Response – We apologize for not providing the experimental details. Muscle samples were fractionated to obtain the lysosome-enriched fractions using differential centrifugation. In this way, we are examining the abundance of the intact V-ATPase complex as well as the membrane-associated Vo section. The dissociated V1 sector would not be present in this fraction. As such, the V1/Vo ratio in the pellet is used as a measure of V-ATPase assembly, which is commonly practiced in the field. In the revision, we have revised the text to more clearly state the rationale of this assay. For a long time, the V-ATPase investigators attempted to use native gels to study V-ATPase assembly like many investigators do with the F1Fo-ATP synthase. Unfortunately, there are no successful cases reported so far.

4) The authors convincingly show that overexpression of ANT1 in the mouse causes muscular atrophy, triggers the integrated stress response, and other downstream pathways already associated with mitochondrial dysfunction. Moreover, several data are suggestive of possible lysosomal impairment. The conclusion that it is the release of lysosomal enzymes in the cytosol that leads to the muscular atrophy is however not so convincing. This conclusion is based on correlating three findings: unaffected protein synthesis, increased cathepsin activity in the cytosol, and decrease total proteins in the muscle. These are interesting correlations, but do not really prove the causality of events. Can it simply be that enhanced autophagy leads to protein degradation? Can the authors show that there is an accelerated protein degradation? Can the authors exclude that the proteasome is responsible for the protein degradation? Proteasome is known to be hyperactive when autophagy is impaired.

Response – We agree that our data does not show that muscle atrophy is a direct cause of proteolytic enzyme release from the lysosome. A demonstration of causality would need to knockout multiple genes encoding cathepsin isoforms, which is technically challenging. Given the drastic increase of cathepsin activities in the cytosol, it would be expected that it may increase protein degradation.

As we showed in the manuscript, the data support increased autophagic activity (e.g., AMPK activation, p62 phosphorylation and increased LC3-II/I ratio). To implement reviewer's comments, we plan to measure the autophagic flux directly in vivo. In addition, we acknowledge the reviewer's point regarding the proteasome. Prior work from our group has shown that proteasomal activity can be upregulated under conditions of mitochondrial stress at the age of 6 months. We will extend the study to 2 month-old mice to be consistent with other data collected at this time point. Based on

the outcome of these new experiment, we will discuss the possibility of alternative proteolytic systems contributing to muscle atrophy in parallel.

Minor comments:

- Please specify what is trp1 in figure 1i.
  Response – Fixed

- Page 9: the sentence "mitochondria respiration is little unaffected" is confusing.
  Response – Fixed

Referee #2:

General comments:

The AAC2 carrier protein mutant variant and its pathological human variant cause clogging the mitochondrial protein import translocases when co/overexpressed. This highly interesting study sheds some light on the molecular mechanisms involved in pathology caused by the mitochondrial protein import stress caused by AAC2/human ANT-linked pathology. The authors find the vacuolar defects, insufficient deacidification as well as the changes in V-ATPase and propose that defective lysosomal function is the main cause of the carrier toxicity.

The main strength and novelty of this paper is a new connection between mitochondrial protein import stress and lysosomal dysfunction, mediated likely through defects in V-ATPase. Also the experiments in mice bring a very interesting insight into the pathology.

Concerns:

The weakness of this study are insufficient molecular mechanisms. The authors convincingly show the defects in vacuole/lysosome, however the underlying reason is unclear.
(1) Does AAC2 variant is a substrate for lysosomal degradation?
(2) What is the underlying cause of V-ATPase defects? Is this a direct interaction or interference with the V ATPase assembly - the mode of V ATPase deregulation is unclear and could in fact originate from yet unknown reason and not direct interference by AAC2.
(3) Is the proteasome activity, earlier reported to be upregulated during mitochondrial import stress, changed? Does this activity may indirectly affect V ATPase?

Response – We thank the reviewer's appreciation that we have convincingly showed the defects in vacuole/lysosome that results from mitochondrial protein import stress.

In fact, many mitochondrial proteins would be expected to be misfolded in extramitochondrial subcompartments due to the absence of assembly partners and/or the lack of proper maturation factors and environment (e.g., membrane composition and pH). At the current stage, the precise mechanism(s) of lysosomal damage is unclear. Lysosomal damage by accumulation of misfolded proteins has been extensively reported in the literature (refs. 50-52). Understanding the underlying biophysical principles is indeed exciting but is also very challenging in the field. In our opinion, demonstrating how and which mitochondrial protein(s) cause lysosomal membrane damage is beyond the scope of the current study. Even in classic lysosomal storage disease, the exact mechanisms by which lipid and glycogen accumulation cause lysosomal dysfunction and pathology remain poorly understood, despite several decades of investigation.

To implement reviewer's comments, we extended the Discussion section to propose several possibilities. In our view, it is possible that similar to classic lysosomal storage disease, overloading of the lysosome by misfolded mitochondrial precursor proteins overwhelms the degradative capacity, causing failure of the functional and structural maintenance of the organelle. Alternatively, specific proteins/aggregates may directly permeabilize the lysosomal membrane. We hope that these comments may prime the discussion in this area and help in generating testable hypotheses in the future.

(4) Is the identified link typical only for AAC2/ANT pathology or other models of protein import stress also affect vacuolar/lysosomal pathway, via V ATPase?

Response – Our data suggest that loss of general protein import machinery, such as Tim18, causes vacuolar damage (Fig. 1l-n). It is therefore likely that the effect is not limited to AAC2/ANT expression. It would be predicted that many aspects of vacuolar biology can be affected, including vacuolar protein maturation/biogenesis (see Fig. 3g), ectopic accumulation of undigested proteins, as well as defects in V-ATPase assembly and function.

Referee #3:

General comments:

Overall, this study describes some very interesting phenotypes that occur in yeast and mammals with AAC/ANT mutations/expression changes, and raises an interesting and testable hypothesis/model that unimported mitochondrial proteins drive lysosomal failure in this context.

While the manuscript contains many very intriguing phenotypes and striking changes in lysosome function, the overall hypothesis/conclusion is not well supported by the current data. As it currently stands, the data supporting the claim that unimported proteins are the driver of the vacuole/lysosome issues is not robust, and needs more investigation to rigorously test this model. It is also not entirely clear in the moue models whether the lysosome dysfunction downstream of import stress if (is) the major driver of muscle atrophy, as rescue experiments are not included. These major limitations would need to be addressed by the authors prior to publication, in addition to the other points raised below.

Response – As we mentioned above, the exact mechanism by which mitochondrial protein import stress causes vacuole/lysosome damage is a subject of future study. Multiple cathepsins are released from the lysosome (Fig. Figure 6a). Genetic and pharmacological rescues would indeed be exciting experiments, but likely not feasible and not within the scope of the current study. Rescuing mitochondria-induced muscle wasting is a long-term commitment in the field. As we mentioned in the response to reviewer 1, we agree that we should leave the possibility open that other pathways (e.g., increased autophagy and proteasomal activity) may also contribute to increased protein degradation and muscle atrophy. We will accordingly revise the discussion section to implement reviewer's comments.

Major Points:

(1) Lack of support for overall model in the data: The authors' hypothesis and model that unimported proteins are driving vacuole and lysosome issues upon AAC/ANT perturbation. However, the evidence supporting this is rather weak. In yeast, the authors show one non-endogenous protein accumulating in the vacuole, and a more thorough examination of the extent of endogenous vacuole targeting of mitochondrial unimported precursors is not included. Thus, it remains unclear how prominent this effect is. In the mouse model, proteomics of the cytosol show more mitochondrial proteins, but it's not clear which if any of these are targeted to the lysosome. Moreover, in both systems, how these unimported proteins could mechanistically cause the phenotypes described remains unclear. Why are the vacuoles and lysosome degradation pathways not capable of handling these proteins in this context? What is the route proteins use to get targeted to the vacuole/lysosome? The

authors need some sort of mechanistic explanation here connecting these dots. Otherwise, it seems equally plausible that the vacuole/lysosome effects with AAC2/ANT are driven by another aspect of the toxic protein on cells.

Response – There are many important questions here, including which mitochondrial proteins are trafficked to the lysosome, how these proteins are targeted to the lysosome, why are these proteins are not degraded, and how are these proteins causing lysosomal damage? Although Lyso-IP is now commonly used for analyzing the lysosomal proteome in cultured human cells, it unfortunately remains a significant challenge to implement this protocol in the study of mouse tissues and yeast cells, especially given the severely damaged lysosome/vacuole. We appreciate the reviewer's insightful comments.

2. Another issue is the conclusion that the synthetic phenotypes between loss of V-ATPase and AAC2 overexpression stems from unimported protein accumulation. While some investigation into mitochondrial ROS/OXPHOS is provided, it again seems very plausible given the vacuoles many impacts on the cells that the lethality may be linked to alterations in pH, iron, calcium ions, etc. in the double mutant. The authors should perform rescue experiments in the double mutants to test if doing things like adding iron to the media or altering the pH can suppress the growth defects. This may suggest that that lethality is caused through a slightly different mechanism than what is proposed here. Again, the data is robust that there are vacuole/lysosome defects, the mechanism underlying that connection is just not entirely clear right now.

Response – We showed that low pH can rescue the synthetic lethality phenotype (Fig. 1i and 1k). Especially in Fig. 1k, rescue was successful under the conditions where OXPHOS is little affected (fig. s1). These data are consistent with heightened vacuolar damage, which the reviewer clearly agrees. To implement the reviewer's comments, we rephrased the text to leave other types of cellular damage open (page 6).

3. Causality vs. correlation in in vivo findings: While the mouse model shows lysosomal damage concurrent with mitochondrial protein import stress, direct evidence that lysosomal dysfunction is the primary driver of muscle atrophy is not fully established (e.g., genetic or pharmacologic rescue experiments targeting lysosome function are missing).

See response to "General comments" above.

4. Specificity of AAC2/ANT1: Do other cloggers cause the same effects? If the downstream driver is unimported proteins, then it seems likely that other cloggers should cause the

same issue. Also, the switching between AAC2 overexpression and the mutant version throughout the first few figures is a bit confusing. It may be helpful to stick with one version or the other. If the effects are specific to one form or another, what does that suggest about the model?

Response – As responded to reviewer 2, our data suggest that a general defect in protein import can lead to vacuolar damage. This is supported by the finding that loss of a general protein import protein such as Tim18 also causes vacuolar damage (Fig. 1l-n). Therefore, the effect is unlikely to be limited to AAC2/ANT expression. We chose to use the overexpression system because it minimally affects mitochondrial function. This was critical, as it allowed us to demonstrate that severe vacuolar damage, rather than defective OXPHOS, is the primary factor inhibiting cell growth. In contrast, the clogging system induces a more severe defect in protein import, which would be expected to affect OXPHOS as well. Importantly, this system not only allowed us to capture more severe phenotypes such as vacuolar fragmentation, but also provided a clinically relevant model for mitochondria-induced vacuolar damage. In our opinion, these two systems are complementary and together strengthen our conclusion.

1st Editorial Decision 21st Oct 2025

Dear Xin Jie,

Thank you for the transfer of your manuscript to EMBO reports and for the feedback and discussions. Based on these, I would like to invite you to revise your study for potential publication in EMBO reports. As discussed, please add a further analysis of autophagy and proteolysis in mice, an extended analysis of the tim18 mutant yeast strains and discuss alternative explanations and reasons for the observed phenotypes.

Please address all referee concerns in a complete point-by-point response. Acceptance of the manuscript will depend on a positive outcome of a second round of review. It is EMBO Reports policy to allow a single round of revision only and acceptance or rejection of the manuscript will therefore depend on the completeness of your responses included in the next, final version of the manuscript.

We realize that it is difficult to revise to a specific deadline. In the interest of protecting the conceptual advance provided by the work, we recommend a revision within 3 months (January 21, 2026).

================
IMPORTANT NOTE:

We perform an initial quality control of all revised manuscripts before re-review. Your manuscript will FAIL this control and the handling will be delayed IN CASE the following APPLIES:

1) A data availability section providing access to data deposited in public databases is missing. If you have not deposited any data, please add a sentence to the data availability section that explains that.

2) Your manuscript contains statistics and error bars based on n=2. Please use scatter blots in these cases. No statistics should be calculated if n=2.

When submitting your revised manuscript, please carefully review the instructions that follow below. Failure to include requested items will delay the evaluation of your revision.
================

When submitting your revised manuscript, we will require:

1) a .docx formatted version of the manuscript text (including legends for main figures, EV figures and tables). Please make sure that the changes are highlighted to be clearly visible.

2) individual production quality figure files as .eps, .tif, .jpg (one file per figure).
Please download our Figure Preparation Guidelines (figure preparation pdf) from our Author Guidelines pages https://www.embopress.org/page/journal/14693178/authorguide for more info on how to prepare your figures.

3) a .docx formatted letter INCLUDING the reviewers' reports and your detailed point-by-point responses to their comments. As part of the EMBO Press transparent editorial process, the point-by-point response is part of the Review Process File (RPF), which will be published alongside your paper.

4) a complete author checklist, which you can download from our author guidelines (<https://www.embopress.org/page/journal/14693178/authorguide>). Please insert information in the checklist that is also reflected in the manuscript. The completed author checklist will also be part of the RPF.

5) Please note that all corresponding authors are required to supply an ORCID ID for their name upon submission of a revised manuscript (<https://orcid.org/>). Please find instructions on how to link your ORCID ID to your account in our manuscript tracking system in our Author guidelines (<https://www.embopress.org/page/journal/14693178/authorguide#authorshipguidelines>)

6) We replaced Supplementary Information with Expanded View (EV) Figures and Tables that are collapsible/expandable online. A maximum of 5 EV Figures can be typeset. EV Figures should be cited as 'Figure EV1, Figure EV2" etc... in the text and their respective legends should be included in the main text after the legends of regular figures.

- For the figures that you do NOT wish to display as Expanded View figures, they should be bundled together with their legends in a single PDF file called *Appendix*, which should start with a short Table of Content. Appendix figures should be referred to in the main text as: "Appendix Figure S1, Appendix Figure S2" etc. See detailed instructions regarding expanded view here: <https://www.embopress.org/page/journal/14693178/authorguide#expandedview>

- Additional Tables/Datasets should be labeled and referred to as Table EV1, Dataset EV1, etc. Legends have to be provided in a separate tab in case of .xls files. Alternatively, the legend can be supplied as a separate text file (README) and zipped together with the Table/Dataset file.

7) Before submitting your revision, primary datasets (and computer code, where appropriate) produced in this study need to be deposited in an appropriate public database (see <
https://www.embopress.org/page/journal/14693178/authorguide#dataavailability>).
Specifically, we would kindly ask you to provide public access to the following datasets:
- RNAseq
- mass spectrometry
- metabolomics, if possible

Please remember to provide a reviewer password if the datasets are not yet public.

The accession numbers and database should be listed in a formal "Data Availability " section (placed after Materials & Method) that follows the model below (see also < https://www.embopress.org/page/journal/14693178/authorguide#dataavailability>). Please note that the Data Availability Section is restricted to new primary data that are part of this study.

Data availability

The datasets (and computer code) produced in this study are available in the following databases:

- RNA-Seq data: Gene Expression Omnibus GSE46843 (https://www.ncbi.nlm.nih.gov/geo/query/acc.cgi?acc=GSE46843)
- [data type]: [name of the resource] [accession number/identifier/doi] ([URL or identifiers.org/DATABASE:ACCESSION])

*** Note - All links should resolve to a page where the data can be accessed. ***

8) At EMBO Press we ask authors to provide source data for the main manuscript figures. You will receive a separate email with instructions for providing source data with your revised manuscript, including how to upload and organize the files.

Additional information on source data and instruction on how to label the files are available
<https://www.embopress.org/page/journal/14693178/authorguide#sourcedata>

9) The journal requires a statement specifying whether or not authors have competing interests (defined as all potential or actual interests that could be perceived to influence the presentation or interpretation of an article). In case of competing interests, this must be specified in your disclosure statement. Further information: https://www.embopress.org/competing-interests

10) Figure legends and data quantification:
The following points must be specified in each figure legend:

- the name of the statistical test used to generate error bars and P values,
- the EXACT p-values,
- the number (n) of independent experiments (please specify technical or biological replicates) underlying each data point,
- the nature of the bars and error bars (s.d., s.e.m.)

- If the data are obtained from n {less than or equal to} 5, show the individual data points in addition to the SD or SEM.
- If the data are obtained from n {less than or equal to} 2, use scatter blots showing the individual data points.

Discussion of statistical methodology can be reported in the materials and methods section, but figure legends should contain a basic description of n, P and the test applied.

See also the guidelines for figure legend preparation:
https://www.embopress.org/page/journal/14693178/authorguide#figureformat

- Please also include scale bars in all microscopy images and define their size in the legend, not the image.

11) Our journal encourages inclusion of *data citations in the reference list* to directly cite datasets that were re-used and obtained from public databases. Data citations in the article text are distinct from normal bibliographical citations and should directly link to the database records from which the data can be accessed. In the main text, data citations are formatted as follows: "Data ref: Smith et al, 2001" or "Data ref: NCBI Sequence Read Archive PRJNA342805, 2017". In the Reference list, data citations must be labeled with "[DATASET]". A data reference must provide the database name, accession number/identifiers and a resolvable link to the landing page from which the data can be accessed at the end of the reference.

Further instructions are available at <https://www.embopress.org/page/journal/14693178/authorguide#referencesformat>.

12) All Materials and Methods need to be described in the main text using our 'Structured Methods' format. According to this format, the Methods section includes a Reagents and Tools Table (listing key reagents, experimental models, software and relevant equipment and including their sources and relevant identifiers) followed by a Methods and Protocols section describing the methods, ideally using a step-by-step protocol format. The aim is to facilitate adoption of the methodologies across labs. Please download and fill our Reagents and Tools Table template (.docx), which you can find in our author guidelines: https://www.embopress.org/page/journal/14693178/authorguide#structuredmethods.
When submitting your revised manuscript, please do not include the Reagents and Tools Table in the Methods section of the manuscript but upload it as a separate file choosing the file type "Reagent Table".
An example of a Method paper with Structured Methods can be found here: https://www.embopress.org/doi/10.15252/msb.20178071.

13) As part of the EMBO publication's Transparent Editorial Process, EMBO Reports publishes online a Review Process File to accompany accepted manuscripts. This File will be published in conjunction with your paper and will include the referee reports, your point-by-point response and all pertinent correspondence relating to the manuscript.

You are able to opt out of this by letting the editorial office know (emboreports@embo.org). If you do opt out, the Review Process File link will point to the following statement: "No Review Process File is available with this article, as the authors have chosen not to make the review process public in this case."

We would also welcome the submission of cover suggestions, or motifs to be used by our Graphics Illustrator in designing a cover.

I look forward to seeing a revised form of your manuscript when it is ready and please let me know if you have questions or comments regarding the revision.

Kind regards,

Martina

Martina Rembold, PhD
Senior Editor
EMBO reports

Response to reviewers' comments – EMBOR-2025-62864-T

Summary – We would like first to thank the reviewers and editors for taking time to evaluate our work, and for the opportunity to transfer the manuscript to *EMBO Reports*. We appreciate the reviewers' endorsement regarding the conceptual novelty of our study, revealing a connection between mitochondrial protein import stress and lysosomal dysfunction, as well as its relevance to muscle pathology. To our reading, the major concerns from the reviewers are focused on the following:

(1) The assumption that the release of lysosomal enzymes is the driver of muscle atrophy is speculative. Although the data showing unaffected protein synthesis, increased cathepsin activity in the cytosol and decreased total protein content are strong, possible contributions from other proteolytic activities including enhanced autophagy and proteasomal activation are not considered.
(2) The mitochondrial protein import model is mainly based on AAC2/ANT1 overexpression. A more direct link between defects in protein import and the observed phenotypes needs to be provided.
(3) The authors' hypothesis and model that unimported proteins are driving vacuole and lysosome issues upon AAC/ANT perturbation. However, the evidence supporting this is rather weak. "….a more thorough examination of the extent of endogenous vacuole targeting of mitochondrial unimported precursors is not included".
(4) A mechanistic explanation is lacking as to how unimported mitochondrial proteins cause lysosomal damage.

To address the reviewers' comments and the feedback from the editor, we revised the manuscript as outlined below.

(1) We measured *in vivo* autophagic flux in both young and aged Ant1^{Tg}/+ mice compared to wild type.
(2) We determined proteasomal activity in both young and aged *Ant1^{Tg}*/+ muscle.
(3) We expanded our analysis to other viable mitochondrial protein import mutants, including *tom5, 6, 7 and 70* (in addition to *tim18* in the initial submission). We found that all these mutants have a *vma*-like phenotype.
(4) We also found that all the mitochondrial protein import mutants are defective in V-ATPase assembly.
(5) We determined the V-ATPase independent effect of *tom7* and *tim18* on vacuolar pH control in *vma13* cells.
(6) We showed that a representative mitochondrial matrix protein, ACO1, is targeted to the vacuole under condition of mitochondrial protein import stress.

Overall, we are excited by the new data that strengthen our conclusion that a mitochondria-to-lysosome proteostatic signaling pathway exists. The data also provided additional insights into the mechanism of mitochondria-induced muscle atrophy.

(7) Finally, we revised the Discussion section and proposed possible mechanisms by which unimported mitochondrial proteins could lead to vacuolar/lysosomal damage, with the aim of stimulating future studies in the field. We also highlighted several aspects of novelties that our study may contribute to the field at the current stage. These include the uncovering of a mitochondria-to-lysosome stress signaling pathway, the identification of a novel mechanism of muscle atrophy, and a bioenergetics-independent mechanism of ISR activation and metabolic remodeling.

Below are point-to-point responses to reviewer's concerns.

Referee #1:

Major comments:

1) How many times have the yeast experiments been performed? There are some claims based on quantification of n=1. This is the case for example for the experiments in Figs 1 and 3.

<u>Response</u> – We thank the reviewer for this important observation. For genetic interactions, we always analyze 2-3 independent zygotes to avoid aberrations in genetic segregations that may skew the results. For the imaging experiments, we perform at least two independent experiments to insure reproducibility. For RNA-seq analysis, three and four biological replicates are included for yeast and mouse skeletal muscle samples, respectively. For transparency, we added this information either in the figure legends or in the Method section.

2) One major concern is that the authors never show that there is actual import stress. Is overexpressed AAC2 or ANT1 in the muscle poorly imported? Can this be seen for instance at early time points? Is there accumulation of other mitochondrial precursors? I think that this is a crucial data to confirm the proposed model.

<u>Response</u> – Agreed. Unimported mitochondrial proteins have variable stability in the cytosol under different stress conditions. In our opinion, the most effective way to document protein import stress within the cell is a mass spectrometry-based study of the cytosolic proteome. This may help in capturing the proteins that are not rapidly turned over when not imported. Using this technique, we found that ANT1 level in the cytosolic fraction is indeed increased by 2-fold in young transgenic mice at the age of 2 months (see dataset EV4). In these mice, we also found that 191 mitochondrial proteins accumulate in the cytosol (see Figure 5i). Our previous studies have shown that mitochondrial proteins are retained and form aggregates in the cytosol of HEK293T cells that transiently express *Ant1* (Mol Biol Cell. 2019, 30:1272-1284), and in yeast cells expressing GAL10-aac2(A128P) (Nature 2015, 524:481-484). On the other hand, we also used *tim18* and several other protein import mutants (*tom5, 6, 7* and *70*) as stressors (see below). These genes directly affect protein import. These mutants exhibit a typical *vma* phenotype, which is also seen in *AAC2*-overexpressing cells. We hope that the cumulative collection of these data strengthens our conclusion supporting a link between mitochondrial protein import stress and vacuolar dysfunction.

3) The authors infer a defect in V-ATPase assembly based on relative abundance of components in a western blot (Fig. 7a-b). This is not convincing, as the differences are minor and assembly is not directly evaluated. Native gels to look directly at assembly would be a better method.

<u>Response</u> – We apologize for not providing sufficient details regarding the rationale and the experimental protocols. The V1 sector is reversibly assembled in both yeast and mammalian cells. Dissociated V1 will not be detected in the membrane fractions where Vo is embedded. In this experiment, we fractionated muscle samples via differential centrifugation to obtain lysosome-enriched fractions. This approach allows us to assess the abundance of the intact V-ATPase complex as well as the membrane-associated Vo sector. Importantly, dissociated V1 is excluded from this fraction and remains in the cytosol. Accordingly, the V1/Vo ratio measured in the pellet serves as a readout of V-ATPase assembly, an approach that is commonly practiced in the field. In the revision, we have revised the text to more clearly state the rationale of this assay. A paragraph is added to the Methods section describing the experimental details. We agree that ANT1 overloading has only a moderate effect on the assembly of V-ATPase. This supports both V-ATPase dependent and independent damage by the unimported mitochondrial proteins, like in yeast. Historically, investigators in the V-ATPase field have explored the use native gel electrophoresis to study V-ATPase assembly, similar to approaches used for the F_1F_o-ATP synthase. However, to our knowledge, this approach has not yielded successful or reproducible results. We thank the reviewer for raising this point, as it highlights a technical challenge in the field. Native gel-based approaches may become informative in the future should new tools become available.

4) The authors convincingly show that overexpression of ANT1 in the mouse causes muscular atrophy, triggers the integrated stress response, and other downstream pathways already associated with mitochondrial dysfunction. Moreover, several data are suggestive of possible lysosomal impairment. The conclusion that it is the release of lysosomal enzymes in the cytosol that leads to the muscular atrophy is however not so convincing. This conclusion is based on correlating three findings: unaffected protein synthesis, increased cathepsin activity in the cytosol, and decrease total proteins in the muscle. These are interesting correlations, but do not really prove the causality of events. Can it simply be that enhanced autophagy leads to protein degradation? Can the authors show that there is an accelerated protein degradation? Can the authors exclude that the proteasome is responsible for the protein degradation? Proteasome is known to be hyperactive when autophagy is impaired.

Response – We showed that increased cathepsin abundance and activity are increased in the cytosol of transgenic muscle, which would be expected to increase protein degradation over time. However, the reviewer raised excellent questions regarding whether increased autophagy and proteasomal activity can also cause excessive protein degradation and muscle atrophy. Accordingly, we measured *in vivo* autophagic flux and proteasomal activity in tissue lysates.

Interestingly, we found autophagic flux is completely stalled in both young and aged Ant1-transgenic muscles (See new data presented in Fig. 7l-7o). This is consistent with severe lysosomal damage, despite AMPK activation. The data led to our conclusion that autophagy does not play a key role in the loss of protein content in the transgenic myofibers.

Secondly, we measured the three proteolytic activities associated with the proteasome in both young and aged *Ant1*-transgenic mice compared to the wild type. We found that at the age of 2 months, the trypsin-like activity is very marginally increased, whereas the chymotrypsin-like and caspase-like activities were not increased in the $Ant1^{Tg}$/+ muscle compared to wild type (Fig. 8f). At 27-28 months of age, all three proteasome-associated protease activities were significantly increased in $Ant1^{Tg}$/+ muscle compared to wild type controls (Fig. 8g). It is therefore possible that enhanced proteasomal activity may also contribute to reduced protein content and muscle atrophy, especially in aged $Ant1^{Tg}$/+ mice.

We are grateful for the reviewer's insightful comment, which prompted us to refine the model of muscle atrophy based on the new data (Fig. 8h).

Minor comments:

- Please specify what is trp1 in figure 1i

  Response – Fixed

- Page 9: the sentence "mitochondria respiration is little unaffected" is confusing.

  Response – Fixed

Referee #2:

- General comments: The AAC2 carrier protein mutant variant and its pathological human variant cause clogging of the mitochondrial protein import translocases when co/overexpressed. This highly interesting study sheds some light on the molecular mechanisms involved in pathology caused by the mitochondrial protein import stress caused by AAC2/human ANT-linked pathology. The authors find the vacuolar defects, insufficient deacidification as well as the changes in V-ATPase and propose that defective lysosomal function is the main cause of the carrier toxicity.

- The main strength and novelty of this paper is a new connection between mitochondrial protein import stress and lysosomal dysfunction, mediated likely through defects in V-ATPase. Also the experiments in mice bring a very interesting insight into the pathology.

Concerns: The weakness of this study are insufficient molecular mechanisms. The authors convincingly show the defects in vacuole/lysosome, however the underlying reason is unclear.

(1) Does AAC2 variant is a substrate for lysosomal degradation?
(2) What is the underlying cause of V-ATPase defects? Is this a direct interaction or interference with the V ATPase assembly - the mode of V ATPase deregulation is unclear and could in fact originate from yet unknown reason and not direct interference by AAC2.
(3) Is the proteasome activity, earlier reported to be upregulated during mitochondrial import stress, changed? Does this activity may indirectly affect V ATPase?

Response – We thank the reviewer for appreciating that we have convincingly demonstrated defects in vacuole/lysosome that results from mitochondrial protein import stress

In fact, we predict that the effect on lysosomal function is not limited to Aac2/Ant1, as many mitochondrial proteins are expected to be misfolded in extramitochondrial subcompartments due to the absence of assembly partners and/or appropriate maturation factors and environments (e.g., membrane composition and pH). Using mass spectrometry, we detected 191 mitochondrial proteins that are retained in the cytosol of Ant1-transgenic muscle. As we know, many mitochondrial proteins are very hydrophobic and therefore not readily detected by mass spectrometry. The investigation of how lysosomes are damaged by the accumulation of misfolded proteins (e.g., alpha-synuclein, tau, etc) has been extensively studied in other contexts (refs. Kakuda *et al*, 2024; Rose *et al*, 2024; Zoncu & Perera, 2022). Understanding the underlying biophysical principles is indeed exciting but also remains highly challenging in the field. In our view, addressing these questions is beyond the scope of the current study. Notably, even in classic lysosomal storage disease, the precise mechanisms by which lipid and glycogen accumulation induce lysosomal dysfunction and pathology remain poorly understood, despite several decades of investigation. Regarding the V-ATPase, it is expected that perturbations in lysosomal biogenesis, assembly/disassembly, protein quality control, membrane properties or proteasomal activation, as suggested by the reviewer, could all influence V-ATPase function. In the yeast system, our data clearly support the notion that unimported mitochondrial proteins can impair vacuolar function through both V-ATPase dependent and independent mechanism, which we believe is an important contribution.

To address the reviewer's comments, we expanded the Discussion section to propose several potential mechanisms. In our view, similar to classic lysosomal storage diseases, overloading of the lysosome with misfolded mitochondrial precursor proteins may overwhelm its degradative capacity, leading to impaired functional and structural maintenance of the organelle. Alternatively, specific proteins/aggregates may directly permeabilize the lysosomal membrane, as proposed by others in the field of degenerative disease. We hope that these considerations help frame the discussion in this area and stimulate the development of testable hypotheses in the future.

(4) Is the identified link typical only for AAC2/ANT pathology or do other models of protein import stress also affect vacuolar/lysosomal pathway, via V ATPase?

Response – This is an excellent question. As such, we extended our analysis to include *tom5, 6, 7* and *70*, in addition to *tim18* that was presented in the initial submission. These mutants are known to directly affect mitochondrial protein import, but these cells can still respire. Our new data showed that these mutants (1) all have a typical *vma* phenotype (i.e., sensitivity to high environmental pH and calcium) (Fig. 2a); (2) partially affect V-ATPase assembly (Fig. 2b-2c); and (3) deacidify the vacuole in

vma13 cells (Fig. 2e-2f). These new data show that the effect is not limited to AAC2/ANT expression. Therefore, intrinsic defects in the mitochondrial protein import pathway would be expected to cause lysosomal stress. Consequently, multiple aspects of vacuolar biology may be affected, including vacuolar protein maturation/biogenesis, ectopic accumulation of undigested proteins, as well as defects in V-ATPase assembly and function.

Referee #3:

General comments - Overall, this study describes some very interesting phenotypes that occur in yeast and mammals with AAC/ANT mutations/expression changes, and raises an interesting and testable hypothesis/model that unimported mitochondrial proteins drive lysosomal failure in this context.

While the manuscript contains many very intriguing phenotypes and striking changes in lysosome function, the overall hypothesis/conclusion is not well supported by the current data. As it currently stands, the data supporting the claim that unimported proteins are the driver of the vacuole/lysosome issues is not robust, and needs more investigation to rigorously test this model. It is also not entirely clear in the moue models whether the lysosome dysfunction downstream of import stress if (is) the major driver of muscle atrophy, as rescue experiments are not included. These major limitations would need to be addressed by the authors prior to publication, in addition to the other points raised below.

Major Points:

(1) Lack of support for overall model in the data: The authors' hypothesis and model that unimported proteins are driving vacuole and lysosome issues upon AAC/ANT perturbation. However, the evidence supporting this is rather weak. In yeast, the authors show one non-endogenous protein accumulating in the vacuole, and a more thorough examination of the extent of endogenous vacuole targeting of mitochondrial unimported precursors is not included. Thus, it remains unclear how prominent this effect is. In the mouse model, proteomics of the cytosol show more mitochondrial proteins, but it's not clear which if any of these are targeted to the lysosome. Moreover, in both systems, how these unimported proteins could mechanistically cause the phenotypes described remains unclear. Why are the vacuoles and lysosome degradation pathways not capable of handling these proteins in this context? What is the route proteins use to get targeted to the vacuole/lysosome? The authors need some sort of mechanistic explanation here connecting these dots. Otherwise, it seems equally plausible that the vacuole/lysosome effects with AAC2/ANT are driven by another aspect of the toxic protein on cells.

<u>*Response*</u> – Undoubtedly, these are all interesting and important questions, including why these proteins are not degraded, how endogenous mitochondrial proteins are trafficked to the vacuole/lysosome for degradation, and how such proteins ultimately cause lysosomal damage.

The lysosomal/vacuolar system is known to be vulnerable to over-accumulation of various substrates, including lipids, glycogen and other complex sugars. A well-established example of sensitivity to protein accumulation is the use of GFP-ATG8 to monitor autophagic flux, as GFP is tightly packed and poorly processed in the yeast vacuole. More broadly, impaired degradation of incoming substrates is a hallmark of many lysosomal storage diseases. In our study, we showed that under conditions of mitochondrial protein import stress, the yeast vacuole becomes enlarged and poorly processed multivesicular bodies accumulate in skeletal muscle, consistent with vacuolar/lysosomal stress due to increased substrate loading. At the current stage, we proposed that autophagy may play a role in the delivery of unimported mitochondrial proteins to the vacuole, as mutation in a specific autophagic gene is synthetically lethal with mitochondrial protein import stress (our unpublished observation). We now discussed this in the revised Discussion section of the manuscript.

At the current stage, we do not yet know precisely how the accumulation of unimported mitochondrial proteins mechanistically causes vacuolar/lysosomal damage. As described above and in our response to reviewer 2, protein overloading may overwhelm degradative capacity and cause functional failure of the vacuole/lysosome. As further discussed in the revised Discussion section, specific proteins/aggregates may directly permeabilize the lysosomal membrane, as proposed by others in the degenerative disease field. Determining which classes of proteins drive vacuolar/lysosomal damage will require further investigation and lies beyond the scope of the current study. We appreciate the reviewer's insightful questions.

An informative approach would be to determine the lysosomal proteome in Ant1-transgenic muscle. However, this remains a significant technical challenge in the field, even though Lyso-IP is now increasingly used to analyze the lysosomal proteome in cultured human cells. To address the reviewer's comments, we instead examined whether mitochondrial protein import stress promotes vacuolar targeting of a representative mitochondria endogenous protein, Aco1, in *vma1* cells with impaired degradative capacity. Indeed, we found that Aco1 is targeted to the vacuole under these conditions (Fig. 1i-j).

2. Another issue is the conclusion that the synthetic phenotypes between loss of V-ATPase and AAC2 overexpression stems from unimported protein accumulation. While some investigation into mitochondrial ROS/OXPHOS is provided, it again seems very plausible given the vacuoles many impacts on the cells that the lethality may be linked to alterations in pH, iron, calcium ions, etc. in the double mutant. The authors should perform rescue experiments in the double mutants to test if doing things like adding iron to the media or altering the pH can suppress the growth defects. This may suggest that that lethality is caused through a slightly different mechanism than what is proposed here. Again, the data is robust that there are vacuole/lysosome defects, the mechanism underlying that connection is just not entirely clear right now.

<u>*Response*</u> – This question was the central motivation for the design of our study and was the main reason for using the AAC2 overexpression model, in which mitochondrial bioenergetic function is minimally affected. In yeast cells expressing one extra copy of AAC2, we did not see any detectable mitochondrial damage. Nevertheless, this is sufficient to produce a synthetic lethal phenotype with *vma13*. We also showed that *AAC2* overexpression alone deacidifies the vacuole (Fig. 3a-3b). These data support our conclusion that severe vacuolar damage underlies the synthetic lethality. Given that vacuolar damage is known to cause mitochondrial damage, we agree that mitochondrial function can be further reduced in the double mutant. However, the synthetic lethality was assessed on raffinose- or glucose containing medium, which supports fermentative growth. Under these conditions, additional impairment of OXPHOS would not be expected to inhibit cell growth. Consistent with this finding, RNA-seq analysis of the double mutant cells showed that many activated genes are involved in pH homeostasis, nutrient homeostasis, vacuolar function and cytosolic proteostasis, rather than those involved in oxidative phosphorylation and anti-oxidant defense. Together, these data support the conclusion that vacuolar dysfunction and loss of proteostasis, rather than primary defects in mitochondrial bioenergetics, are likely the cause of the synthetic lethality phenotype.

3. Causality vs. correlation in in vivo findings: While the mouse model shows lysosomal damage concurrent with mitochondrial protein import stress, direct evidence that lysosomal dysfunction is the primary driver of muscle atrophy is not fully established (e.g., genetic or pharmacologic rescue experiments targeting lysosome function are missing).

<u>*Response*</u> – We agree that establishing direct causality through genetic or pharmacological rescue of lysosomal dysfunction would be highly informative and potentially of broad translational relevance, including for lysosomal storage diseases, Parkinson's and Alzheimer's disease. However, effective

rescue of lysosomal dysfunction in vivo remains challenging, particularly in the context of mitochondrial protein important stress, where lysosomal impairment is accompanied by release of multiple cathepsins, activation of proteasome-associated proteolytic activities and disruption of proteostasis. We believe our findings provide a compelling framework that positions mitochondrial protein import stress as a key contributor to lysosomal dysfunction and subsequent muscle atrophy, which will stimulate future studies aimed at directly testing causality and therapeutic rescue.

4. Specificity of AAC2/ANT1: Do other cloggers cause the same effects? If the downstream driver is unimported proteins, then it seems likely that other cloggers should cause the same issue. Also, the switching between AAC2 overexpression and the mutant version throughout the first few figures is a bit confusing. It may be helpful to stick with one version or the other. If the effects are specific to one form or another, what does that suggest about the model?

Response – As noted in our response to reviewer 2, the most relevant approach to address this question is to examine additional perturbations that directly impair the mitochondrial protein import machinery. We have now done so. In addition to Tim18, we show that loss of Tom5, 6, 7 and 70 each results in a characteristic *vma* phenotype, including reduced V-ATPase assembly and severe defects in vacuolar acidification in the *vma13* background (see Fig. 2). These data support the conclusion that unimported mitochondrial proteins, rather than AAC2 specifically, are likely responsible for inducing vacuolar dysfunction.

Throughout the manuscript, we intentionally employ both the *AAC2* overexpression model and clogger-based import defect model because they provide complementary insights. The *AAC2* overexpression model enabled us to learn that severe vacuolar damage, rather than defective OXPHOS, is likely the primary factor inhibiting cell growth. It also provided a system analogous to the Ant1 overexpression model in mice. In contrast, the clogging system induces a more severe defect in protein import, which would be expected to affect OXPHOS as well. Importantly, this system not only allowed us to capture more severe phenotypes such as vacuolar fragmentation, but it also provided a clinically relevant model for mitochondria-induced vacuolar damage. Finally, in the revised manuscript, we expanded our study to include not only *tim18 but also tom5, tom6, tom7* and *tom70* that intrinsically affect protein import. This comprehensive further strengthens the idea that different forms of defects in protein import can converge to compromise vacuolar function. In our view, these three types of stressors are complementary and when considered together, substantially strengthen our conclusions and the clinical relevance of our study. We appreciate reviewer's comments.

1st Revision - Editorial Decision 19th Mar 2026

Dear Xin Jie,

Thank you once more for the submission of your revised manuscript to EMBO reports. I have already forwarded you the positive referee comments and follow herewith up with the formal decision letter.

Before we can formally accept your manuscript for publication in EMBO reports, I need you to address the following points:

- The manuscript sections should be in the following order: Title page - Abstract & Keywords - Introduction - Results - Discussion - Methods - Data Availability - Acknowledgments - Disclosure Statement & Competing Interests - References - Figure Legends - (Main Tables with legends if applicable) - Expanded View Figure Legends.

- Please cite Takaine & Morita, 2023 like this in the text: (preprint: Takaine & Morita, 2023).

- "Data and materials availability" should be renamed to "Data Availability". Moreover, this section should exclusively refer to the datasets depositid in public repositories. Therefore, please remove: "All remaining data is available in the Article and Supplementary Data files. Additional inquiries can be directed to the corresponding author."

- Regarding the Author Contributions, we now use CRediT to specify the contributions of each author in the journal submission system. CRediT replaces the author contribution section, which therefore needs to be removed from the manuscript text. You can use the free text box in our system if you wish to provide more detailed descriptions. See also guide to authors https://www.embopress.org/page/journal/14693178/authorguide#authorshipguidelines.

- The funding information needs to be part of Acknowledgments in the manuscript, so the 'Funding' subheading is not needed.

- Table S4 on page 10 is a wrong callout. Please check and correct the callout.

- Datasets EV1-5 need a legend each in a separate tab of the .xls file. The legend needs to carry/include the title "Dataset EV#".

- Appendix Figure S1b, please add a scale bar and define it in the legend. Please note that the figure panels should be labeled with capital letters (A instead of a) in all figures.

- Please list each figure with its page number on the title page of the Appendix (the table of content).

- Mouse experiments: please state the reference approval number in addition to the authority granting approval in the methods section.

- In Figure 5L, the two TPS blots shown look very similar to each other, also in the source data file. Could you please double-check and confirm that both TPS blots are uniqute?

- Figure panels should be labeled with capital letters.

- Please address the following points in the figure legends:
1) indicate the statistical test used for data analysis in the legends of figures 1H, 4A, 5B, D, F, H, K, M
2) provide the information related to n in the legends of figures 6D, E, G, H; EV5 C, S2 B
3) define the error bars in the legends of figures 5B, D, F, H, K, M
4) add a scale bar and its definition for figure S1 B.

- I introduced a few minor changes in the abstract. Could you please review the modified text copied at the end of this e-mail? Thank you.

- Finally, EMBO Reports papers are accompanied online by
A) a short (1-2 sentences) summary of the findings and their significance,
B) 2-3 bullet points highlighting key results and
C) a schematic summary figure that provides a sketch of the major findings (not a data image).
Please provide the summary figure as a separate file in PNG or JPG format at a size of 550x300-600 pixels (width x height). Please note that the size is rather small and that text needs to be readable at the final size. Please send us this information along with the revised manuscript.

We look forward to seeing a final version of your manuscript as soon as possible.

With kind regards,

Martina

Martina Rembold, PhD
Senior Editor
EMBO reports

==================

Referee #1:

The revised version of the manuscript is greatly improved and addresses lots of previous concerns. Although the mechanistic insights are not revealed, the phenomenon is now comprehensively demonstrated.
Given a speculative level concerning mechanisms, I suggest to make the discussion more concise. Whether the phenomenon is related to hydrophobic proteins or all mitochondrial proteins is also still unclear- this should be briefly mentioned in the discussion.

Referee #2:

The authors have done a very good job addressing the concerns raised in the initial review. In particular, they have added new experiments examining autophagic flux and proteasomal activity in the mouse model, expanded the analysis to additional mitochondrial protein import mutants in yeast, and strengthened the evidence linking mitochondrial protein import stress with vacuolar/lysosomal dysfunction. The authors also provide additional support for cytosolic accumulation of mitochondrial proteins and include new data suggesting that endogenous mitochondrial proteins can be targeted to the vacuole under import stress.

While the precise mechanism connecting mitochondrial protein import stress to lysosomal dysfunction remains to be fully defined, the authors have clarified the limitations of the current data and moderated the interpretation accordingly. Overall, this is a nice study, and the authors have done a great job addressing the concerns raised previously. The manuscript is now appropriate for publication in EMBO Reports.

Referee #3:

The authors have addressed several of the previous concerns. Importantly, they now show that there is autophagy stalling, as well as increased proteasomal activity in the muscle of ANT1-overexpressing mice. They also extended their model to other import mutants, at least in yeast. Overall, the revision makes the manuscript stronger, while still leaving several mechanistic questions unresolved (see comments from reviewer 3). The authors have now revised the discussion to clarify the missing points in their model. However, I think that the report of this link between mitochondrial import stress and lysosomal dysfunction is interesting and worth being reported. It will stimulate further research in the field.

===============

Proposed abstract:

Mitochondrial and lysosomal abnormalities co-occur in aging-related diseases with progressive tissue atrophy. It remains unclear whether these two pathogenic pathways affect tissue homeostasis independently, convergently or epistatically. We show that mitochondrial protein import stress causes vacuolar damage in yeast, manifested by V-ATPase disassembly, and vacuolar deacidification and fragmentation. In a mouse model of mitochondrial protein import stress induced by overloading of the nuclear-encoded ANT1 protein, we observe progressive muscle atrophy independent of bioenergetic defects. Like in yeast mutants with severe vacuolar damage, genes involved in amino acid uptake/biosynthesis, one-carbon metabolism, lysosomal biogenesis and iron homeostasis are activated in the skeletal muscle of Ant1-transgenic mice. The affected muscles accumulate glycogen, lipofuscin and poorly processed multivesicular bodies. Despite activation of lysosomal repair and lysophagic pathways, autophagic flux is severely stalled. During aging, various proteolytic cathepsins are increasingly released from the

lysosomal lumen into the cytosol. Together with proteasomal activation, this may contribute to unbalanced proteostasis, reduced myofiber size and skeletal muscle atrophy. Our study therefore discovered an evolutionarily conserved mitochondria-to-lysosome proteotoxic axis that affects tissue mass homeostasis during aging.

All minor editorial requests have been addressed by the authors.

Prof. Xin Jie Chen
SUNY Upstate Medical University
Department of Biochemistry and Molecular Biology
750 East Adams Street
Syracuse, NY 13210
United States

Dear Xin Jie,

I am very pleased to accept your manuscript for publication in the next available issue of EMBO reports. Thank you for your contribution to our journal.

Your manuscript will be processed for publication by EMBO Press. It will be copy edited and you will receive page proofs prior to publication. Please note that you will be contacted by Springer Nature Author Services to complete licensing and payment information.

You may qualify for financial assistance for your publication charges - either via a Springer Nature fully open access agreement or an EMBO initiative. Check your eligibility: https://link.springer.com/journal/44319/how-to-publish-with-us

Should you be planning a Press Release on your article, please get in contact with embo_production@springernature.com as early as possible in order to coordinate publication and release dates.

If you have any questions, please do not hesitate to contact the Editorial Office. Thank you for your contribution to EMBO Reports.

Kind regards,

Martina

Martina Rembold, PhD
Senior Editor
EMBO reports

>>> Please note that it is EMBO Reports policy for the transcript of the editorial process (containing referee reports and your response letter) to be published as an online supplement to each paper. If you do NOT want this, you will need to inform the Editorial Office via email immediately. More information is available here: https://link.springer.com/partners/embo-press/editorial-policies#Peer%20review

EMBO Press Author Checklist

Corresponding Author Name: Xin Jie Chen

Journal Submitted to: EMBO Reports
Manuscript Number: EMBOR-2025-62864-T

USEFUL LINKS FOR COMPLETING THIS FORM
The EMBO Journal - Author Guidelines
EMBO Reports - Author Guidelines
Molecular Systems Biology - Author Guidelines
EMBO Molecular Medicine - Author Guidelines

Reporting Checklist for Life Science Articles (updated January
This checklist is adapted from Materials Design Analysis Reporting (MDAR) Checklist for Authors. MDAR establishes a minimum set of requirements in transparent reporting in the life sciences (see Statement of Task: 10.31222/osf.io/9sm4x). Please follow the journal's guidelines in preparing your
Please note that a copy of this checklist will be published alongside your article.

Abridged guidelines for figures
1. Data
The data shown in figures should satisfy the following conditions:
- the data were obtained and processed according to the field's best practice and are presented to reflect the results of the experiments in an accurate and unbiased manner.
- ideally, figure panels should include only measurements that are directly comparable to each other and obtained with the same assay.
- plots include clearly labeled error bars for independent experiments and sample sizes. Unless justified, error bars should not be shown for technical
- if n<5, the individual data points from each experiment should be plotted. Any statistical test employed should be justified.
- Source Data should be included to report the data underlying figures according to the guidelines set out in the authorship guidelines on Data

2. Captions
Each figure caption should contain the following information, for each panel where they are relevant:
- a specification of the experimental system investigated (eg cell line, species name).
- the assay(s) and method(s) used to carry out the reported observations and measurements.
- an explicit mention of the biological and chemical entity(ies) that are being measured.
- an explicit mention of the biological and chemical entity(ies) that are altered/varied/perturbed in a controlled manner.
- the exact sample size (n) for each experimental group/condition, given as a number, not a range;
- a description of the sample collection allowing the reader to understand whether the samples represent technical or biological replicates (including how many animals, litters, cultures, etc.).
- a statement of how many times the experiment shown was independently replicated in the laboratory.
- definitions of statistical methods and measures:

  n es s s as es ease s e f e er a re s n a re s e es s n an ann ne es s an e
  unambiguously identified by name only, but more complex techniques should be described in the methods section;
  - are tests one-sided or two-sided?
  - are there adjustments for multiple comparisons?
  - exact statistical test results, e.g., P values = x but not P values < x;
  - definition of 'center values' as median or average;
  - definition of error bars as s.d. or s.e.m.

Please complete ALL of the questions below.
Select "Not Applicable" only when the requested information is not relevant for your study.

Materials

Newly Created Materials	Information included in the manuscript?	In which section is the information available? (Reagents and Tools Table, Materials and Methods, Figures, Data Availability Section)
New materials and reagents need to be available; do any restrictions apply?	Yes	Reagents and Tools Table, Materials and Methods

Antibodies	Information included in the manuscript?	In which section is the information available? (Reagents and Tools Table, Materials and Methods, Figures, Data Availability Section)
For **antibodies** provide the following information:<br>- Commercial antibodies: RRID (if possible) or supplier name, catalogue number and or/clone number<br>- Non-commercial: RRID or citation	Yes	Reagents and Tools Table, Materials and Methods

DNA and RNA sequences	Information included in the manuscript?	In which section is the information available? (Reagents and Tools Table, Materials and Methods, Figures, Data Availability Section)
**Short novel DNA or RNA including primers, probes**: provide the sequences.	Not Applicable	

Cell materials	Information included in the manuscript?	In which section is the information available? (Reagents and Tools Table, Materials and Methods, Figures, Data Availability Section)
**Cell lines:** Provide species information, strain. Provide accession number in repository **OR** supplier name, catalog number, clone number, and/**OR** RRID.	Not Applicable	
**Primary cultures:** Provide species, strain, sex of origin, genetic modification status.	Not Applicable	
Report if the cell lines were recently **authenticated** (e.g., by STR profiling) and tested for mycoplasma contamination.	Not Applicable	

Experimental animals	Information included in the manuscript?	In which section is the information available? (Reagents and Tools Table, Materials and Methods, Figures, Data Availability Section)
**Laboratory animals or Model organisms:** Provide species, strain, sex, age, genetic modification status. Provide accession number in repository **OR** supplier name, catalog number, clone number, **OR** RRID.	Yes	Reagents and Tools Table, Materials and Methods
**Animal observed in or captured from the field:** Provide species, sex, and age where possible.	Not Applicable	
Please detail **housing and husbandry conditions**.	Yes	Materials and Methods

Plants and microbes	Information included in the manuscript?	In which section is the information available? (Reagents and Tools Table, Materials and Methods, Figures, Data Availability Section)
**Plants:** provide species and strain, ecotype and cultivar where relevant, unique accession number if available, and source (including location for collected wild specimens).	Not Applicable	
**Microbes:** provide species and strain, unique accession number if available, and source.	Not Applicable	

Human research participants	Information included in the manuscript?	In which section is the information available? (Reagents and Tools Table, Materials and Methods, Figures, Data Availability Section)
If collected and within the bounds of privacy constraints report on age, sex and gender or ethnicity for all study participants.	Not Applicable	

Core facilities	Information included in the manuscript?	In which section is the information available? (Reagents and Tools Table, Materials and Methods, Figures, Data Availability Section)
If your work benefited from core facilities, was their service mentioned in the acknowledgments section?	Yes	Materials and Methods

Design

Study protocol	Information included in the manuscript?	In which section is the information available? (Reagents and Tools Table, Materials and Methods, Figures, Data Availability Section)
If study protocol has been **pre-registered, provide DOI in the manuscript**. For clinical trials, provide the trial registration number **OR** cite DOI.	Not Applicable	
Report the **clinical trial registration number** (at ClinicalTrials.gov or equivalent), where applicable.	Not Applicable	

Laboratory protocol	Information included in the manuscript?	In which section is the information available? (Reagents and Tools Table, Materials and Methods, Figures, Data Availability Section)
Provide DOI OR other citation details if **external detailed step-by-step protocols** are available.	Not Applicable	

Experimental study design and statistics	Information included in the manuscript?	In which section is the information available? (Reagents and Tools Table, Materials and Methods, Figures, Data Availability Section)
Include a statement about **sample size** estimate even if no statistical methods were used.	Yes	Figures, Materials and Methods
ere an s e s a en n e e effe s f s e e as en allocating animals/samples to treatment (e.g. **randomization procedure**)? If yes, have they been described?	Not Applicable	
Include a statement about **blinding** even if no blinding was done.	Not Applicable	
Describe **inclusion/exclusion criteria** if samples or animals were e e fr e ana s s ere e r er a re es a s e<br><br>If sample or data points were omitted from analysis, report if this was due to attrition or intentional exclusion and provide justification.	Not Applicable	
For every figure, are **statistical tests** justified as appropriate? Do the data meet the assumptions of the tests (e.g., normal distribution)? Describe any methods used to assess it. Is there an estimate of variation within each group of data? Is the variance similar between the groups that are being statistically compared?	Yes	Figures

Sample definition and in-laboratory replication	Information included in the manuscript?	In which section is the information available? (Reagents and Tools Table, Materials and Methods, Figures, Data Availability Section)
In the figure legends: state number of times the experiment was **replicated** in laboratory.	Yes	Figures
In the figure legends: define whether data describe **technical or biological replicates**.	Yes	Figures

Ethics

Ethics	Information included in the manuscript?	In which section is the information available? (Reagents and Tools Table, Materials and Methods, Figures, Data Availability Section)
Studies involving **human participants**: State details of **authority granting ethics approval** (IRB or equivalent committee(s), provide reference number for approval.	Not Applicable	
Studies involving **human participants**: Include a statement confirming that **informed consent** was obtained from all subjects and that the experiments nf r e e r n es se n e e ara n f e s n an e e ar en f ea an an er es e n e r	Not Applicable	
Studies involving **human participants:** For publication of **patient photos**, include a statement confirming that consent to publish was obtained.	Not Applicable	
Studies involving experimental **animals**: State details of **authority granting ethics approval** (IRB or equivalent committee(s), provide reference number for approval. Include a statement of compliance with ethical regulations.	Yes	Materials and Methods
Studies involving **specimen and field samples:** State if relevant **permits** obtained, provide details of authority approving study; if none were required, explain why.	Not Applicable	

Dual Use Research of Concern (DURC)	Information included in the manuscript?	In which section is the information available? (Reagents and Tools Table, Materials and Methods, Figures, Data Availability Section)
Could your study fall under dual use research restrictions? Please check biosecurity documents and list of **select agents and toxins** (CDC): https://www.selectagents.gov/sat/list.htm	Not Applicable	
If you used a select agent, is the security level of the lab appropriate and reported in the manuscript?	Not Applicable	
If a study is subject to dual use research of concern regulations, is the name of the **authority granting approval and reference number** for the regulatory approval provided in the manuscript?	Not Applicable	

Reporting

The MDAR framework recommends adoption of discipline-specific guidelines, established and endorsed through community initiatives. Journals have their own policy about requiring specific guidelines and recommendations to complement MDAR.

Adherence to community standards	Information included in the manuscript?	In which section is the information available? (Reagents and Tools Table, Materials and Methods, Figures, Data Availability Section)
State if relevant guidelines or checklists (e.g., **ICMJE, MIBBI, ARRIVE, PRISMA**) have been followed or provided.	Not Applicable	
For **tumor marker prognostic studies,** we recommend that you follow the **REMARK** reporting guidelines (see link list at top right). See author guidelines, under 'Reporting Guidelines'. Please confirm you have followed these guidelines.	Not Applicable	
For **phase II and III randomized controlled trials**, please refer to the **CONSORT** flow diagram (see link list at top right) and submit the CONSORT checklist (see link list at top right) with your submission. See author guidelines, under 'Reporting Guidelines'. Please confirm you have submitted this list.	Not Applicable	

Data Availability

Data availability	Information included in the manuscript?	In which section is the information available? (Reagents and Tools Table, Materials and Methods, Figures, Data Availability Section)
a e **primary datasets** been deposited according to the journal's guidelines (see 'Data Deposition' section) and the respective accession numbers provided in the Data Availability Section?	Yes	Materials and Methods
ere **human clinical and genomic datasets** deposited in a public access-controlled repository in accordance to ethical obligations to the patients and to the applicable consent agreement?	Not Applicable	
Are **computational models** that are central and integral to a study a a a e res r ns n a a ne rea a e f r ere e relevant accession numbers or links provided?	Not Applicable	
If publicly available data were reused, provide the respective **data citations in the reference list**.	Not Applicable	
